# Born This Way: Individuality Is Seeded Before Birth and Robust to Ecological Stress

**DOI:** 10.1111/ele.70454

**Published:** 2026-09-14

**Authors:** James H. Gallagher, Ammon D. Perkes, Chia‐Chen Chang, Emma S. Chirila, Karen Kacevas, Kate L. Laskowski

**Affiliations:** ^1^ Department of Evolution and Ecology University of California Davis Davis California USA; ^2^ Department of Biological Sciences National University of Singapore Singapore

**Keywords:** animal personality, development, developmental plasticity, individual variation, maternal effects, predation, variance partitioning

## Abstract

Behavioural individuality is widespread, yet its developmental origins are unclear. We subjected clonal individuals to a critical ecological challenge—the threat of predation—to disentangle the developmental mechanisms of individuality. Predation may erode or amplify among‐individual differences, demonstrating that individuality is developmentally plastic. If individuality is instead determined before birth, it should resist environmental influences. We tracked behaviours of genetically identical fish (Amazon mollies), reared with or without predation stress, continuously from birth for 4 weeks. While predation shifted mean‐level behaviours, the magnitude of individuality was unaffected, indicating that individuality crystallizes robustly over time under stress and without genetic variation. Surprisingly, maternal identity strongly shaped swimming velocity but not refuge use, suggesting distinct developmental mechanisms for different ecologically relevant behaviours. We show that individuality persists despite ecological stress and is seeded before birth through non‐genetic factors. Even in the face of a shared environmental challenge, the behavioural trajectories of individuals are unique.

## Graphical abstract

We reared and recorded behaviours of clonal Amazon mollies in standardized environments, with and without predation stress, from birth through week four of life, to understand how the developmental environment influences the emergence of behavioural individuality. We found that while stress affected mean‐level behaviours, it did not influence the magnitude of behavioural individuality, suggesting that individuality was seeded before birth through non‐genetic factors.

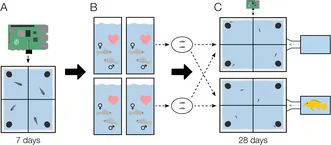

## 1 Introduction

Understanding what shapes individuality has been a long‐standing, fundamental question in the field of biology that has permeated many aspects of culture, from philosophy to science fiction. Behavioural individuality—consistent differences in behaviour among individuals—is ubiquitous across the animal kingdom (Bell et al. 2009; de Bivort 2025; Dall et al. 2012; Sih, Bell, and Johnson 2004; Sih, Bell, Johnson, and Ziemba 2004), can have important fitness implications for individuals (Dall et al. 2004; Dingemanse and Réale 2005; Li et al. 2024; Moiron et al. 2020; Scherer et al. 2023; Smith and Blumstein 2008), and can shape the evolutionary trajectories of populations (Duckworth 2009; Laskowski, Chang, et al. 2022; Wagner and Altenberg 1996), even driving speciation (Ingley and Johnson 2014; Wolf and Weissing 2012). Where do individual differences in behaviour come from? While this question has been historically posed as the ‘nature versus nurture’ debate, this framing is now widely regarded as a false dichotomy, as both genetic and environmental variation will almost always interact to influence an individual's behaviour (Lewkowicz 2011; Saltz et al. 2018). Considerable effort has been spent controlling for these two factors in order to pinpoint the drivers of behavioural individuality. Nevertheless, there is a growing body of work indicating that even when genetic and environmental variation is minimized, individuals still exhibit consistent differences in their behaviours (Freund et al. 2013; Kain et al. 2015; Schuett et al. 2011; Vogt et al. 2008), even at birth (Bierbach et al. 2017; Laskowski, Bierbach, et al. 2022). Some of this variation may be due to variation in pre‐birth influences, such as maternal effects or developmental stochasticity, which may play an underappreciated role in generating variation among individuals (de Bivort 2025; Johnson et al. 2025; Mousseau and Fox 1998; Mulder et al. 2002; Oates 2011; Richards 2006; Vogt 2015; Vogt et al. 2008). This type of non‐genetic variation may help set the seed around which further behavioural individuality crystallizes. Regardless of where such initial differences arise, the continued persistence of individuality despite our best efforts to minimize it suggests that we still lack a full understanding of the key mechanisms underlying its emergence. To identify these mechanisms, we must consider not only *what* shapes behaviour, but also *how* behavioural differences emerge over the course of life (Laskowski, Chang, et al. 2022; Stamps and Krishnan 2014).

Development is an iterative, path‐dependent process, where early experiences may lead to lasting behavioural differences (Monaghan 2007; Zipple et al. 2025). Thus, tracking how individuals respond to environmental challenges during development can reveal the processes that shape their behaviours. One predominant environmental feature that individuals must contend with is that of risk, most notably in the form of predation. Because most animals are subject to predation risk, their behavioural responses to this risk can heavily impact their fitness. Across taxa, predation stress during development has been shown to have profound effects on individual behaviour, brain function, and personality (Bell and Sih 2007; Broder and Angeloni 2014; Bucklaew and Dochtermann 2021; Edenbrow and Croft 2013; Ehlman et al. 2019; Krama et al. 2023; Płaskonka et al. 2024; Scherer et al. 2024; Sommer‐Trembo et al. 2016; Tariel et al. 2020; Toscano et al. 2014; Urszán et al. 2018), suggesting that predation may play a key role in shaping behavioural individuality. However, we still do not understand how predation shapes the *emergence* of individuality from birth throughout ontogeny. This is an important knowledge gap because exposure to predation stress may impact patterns of individual behavioural variation in different ways. On one hand, predation stress may collapse variation attributed to among‐individual differences, making individuals behave more similar to each other if there is an optimal behavioural strategy for survival in a predator‐rich environment (Dingemanse et al. 2009). Alternatively, predation stress could instead expand among‐individual differences (Urszán et al. 2018), as there may be many strategies to mitigate risk in the face of predation. Indeed, life history theory, such as the pace‐of‐life syndrome hypothesis, suggests that predation risk could strengthen trade‐offs between alternative behavioural strategies, thereby potentially expanding among‐individual variation (Réale et al. 2010; Dammhahn et al. 2018). A final possibility is that individual differences may not be impacted by predation stress during development, implying a predominant role for pre‐birth factors in driving behavioural individuality. Disentangling these possibilities requires closely tracking individual behaviour from birth throughout development, across different developmental environments.

Studying the emergence of individuality over development can be very challenging due to the presence of confounding genetic variation in animals, as well as an inability to reliably measure the behaviour of individuals from birth in standardized conditions throughout ontogeny, either due to features of the study species (e.g., some species require parental care, introducing uncontrolled variation in experience among offspring) or technological limitations in following individuals at such early points in their lives. To fully understand the processes that shape behavioural variation, we must be able to solve these challenges and intimately track the details of the timing and rate at which behaviours emerge in individuals (Dupont et al. 2024).

**FIGURE 1:**
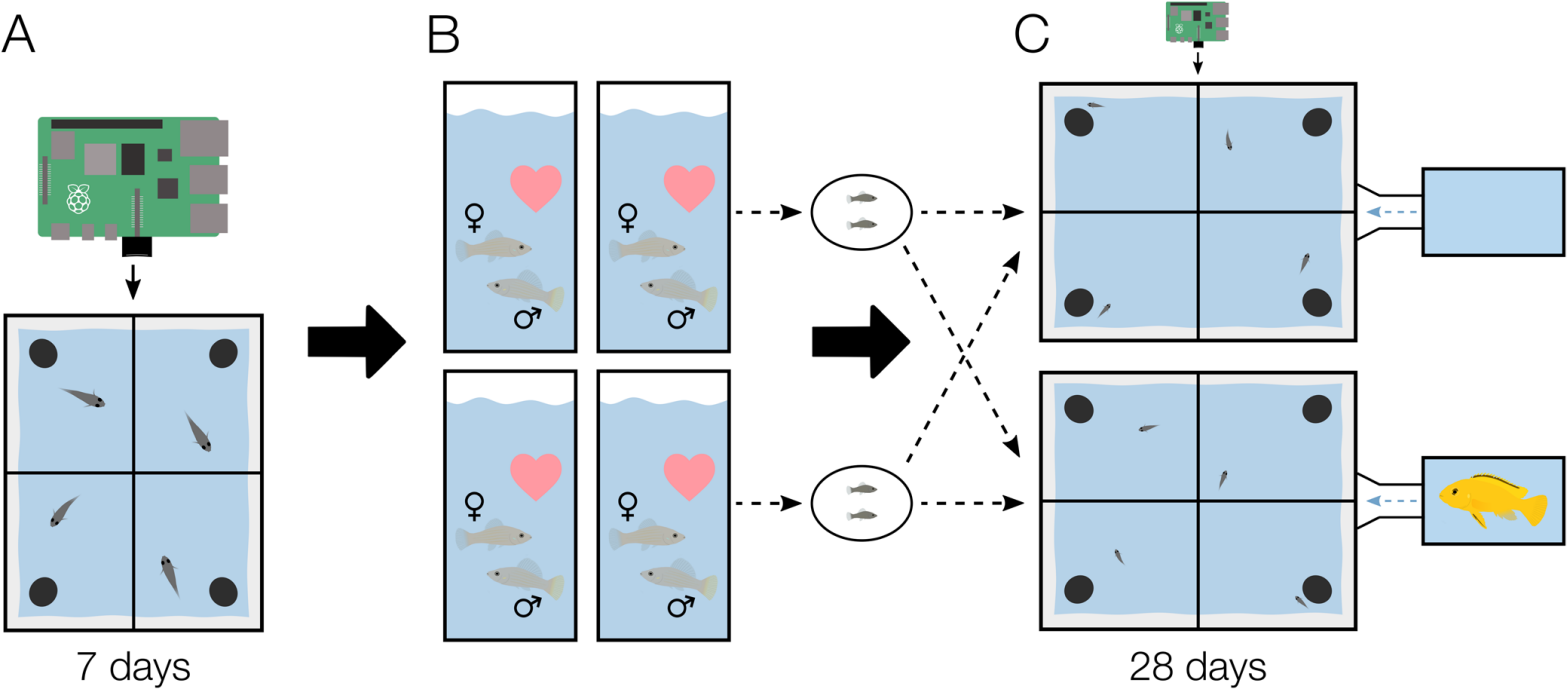
Experimental design. (A) Adult Amazon mollies were each placed in identical individual tanks in our custom tracking system and recorded using cameras attached to Raspberry Pi computers for 12 h each day over 1 week. (B) Fish were then placed in individual tanks with a male Atlantic molly to initiate reproduction. (C) Offspring born from the breeding tanks were split evenly by brood and placed into individual tanks in one of two closed‐water tracking systems—with and without water containing predator cues—and continuously recorded for 4 weeks.

Here, we used an ideal animal study system in conjunction with high‐resolution tracking to bypass historical experimental limitations and deeply study the emergence of behavioural individuality over ontogeny. The Amazon molly ( *Poecilia formosa* ) is an all‐female freshwater fish that reproduces via gynogenesis, resulting in individuals that are genetically identical to each other and their mothers (Lampert and Schartl 2008; Schartl et al. 1995; Schlupp et al. 2007; Schlupp and Plath 2005), allowing us to minimize the effects of confounding (among‐individual) genetic variation, analogous to twin studies in humans (Polderman et al. 2015). They are also live‐bearing without any parental care, so individuals can be isolated from birth with minimal consequence to standardize life experience from day one. We combined this optimal study species with custom tracking technology (using cameras connected to individual Raspberry Pi computers; Jolles 2021; Laskowski, Bierbach, et al. 2022) to record and extract behaviours from individuals (*N* = 107) at high temporal resolution (1 s resolution for 12 h every day) over the first 28 days of their lives. Amazons can reach sexual maturity by ~70 days of age (Scherer et al. 2023), so our observation period represents a complete picture of the early stages of development. These fish were placed in one of two environmental conditions—with or without exposure to predator cues—to determine how stress shapes behaviour and the magnitude of individuality across development. We also standardized the environments that these individuals' mothers experienced in the week prior to breeding (with none of them facing predation stress at any time during their lives) and used the same tracking approach to measure the behaviour of all mothers before they gave birth (*N* = 13, for 7 days) to test whether maternal behaviour and/or maternal identity explain offspring behaviour (Figure 1). This resulted in over 30,000 h of data‐rich recordings (> 100 million data points) that we then quantified using a convolutional neural network‐based pipeline to track the position of each individual all day, every day. Using these data, we extracted ecologically relevant behavioural metrics, such as swimming velocity and time spent near a refuge in the tank, that are likely to be affected by predation threat (Brown 2003; Heczko and Seghers 1981; Lawrence and Smith 1989; Lehtiniemi 2005; Mathis et al. 1993). To date, this work is the first to continuously track how individuals respond to predation stress with such high temporal resolution from the very beginning of life.

In our study, we asked a series of questions to disentangle the developmental drivers of behavioural variation. First, we asked how average behaviour shifts over ontogeny in the face of a significant environmental challenge (threat of predation). Predator‐exposed fish may adjust fitness‐relevant behaviours, such as swimming velocity and time spent near their shelter, and/or show different behavioural developmental trajectories from control fish. Second, we investigated which factors (developmental environment, maternal effects, and remaining non‐genetic among‐individual variation) best explain behavioural variance. By determining what shapes behavioural variation, we will also learn whether different behaviours share the same developmental mechanisms. Finally, and of prime interest here, we tested whether and how predation alters the magnitude of individuality—quantified as the relative proportion of among‐individual variance—across developmental time compared to animals that were reared under benign conditions. This will reveal whether individuality itself is developmentally plastic or determined before birth through non‐genetic factors.

## 2 Methods

### 2.1 Maternal Behaviour and Breeding

To track the behaviour of mothers of experimental fish, we placed adult Amazon mollies in individual observation tanks and monitored them for 1 week (Figure 1A). All adult fish were of similar age and reared in the same conditions. We used fish from the same clonal lineage to minimize among‐individual genetic variation as much as possible. Other studies on behavioural individuality in the Amazon molly found similar results using this and other clonal lineages (Bierbach et al. 2017; Laskowski, Bierbach, et al. 2022), suggesting that the persistence of individuality in Amazons is not lineage‐specific. Above each set of four tanks was a mounted camera attached to a Raspberry Pi 4B computer that consistently recorded individual behaviours.

After 1 week, we transferred each adult fish (*N* = 13) to an individual 18.93‐L (5‐gal) glass breeding tank containing an artificial plant for shelter and a male Atlantic molly ( *Poecilia mexicana* ) to initiate breeding (Figure 1B). Amazon mollies are gynogenetic, meaning they require mating with a related species to trigger embryogenesis, but offspring are genetic clones of the mother (Schartl et al. 1995; Schlupp et al. 2007). One week later, we removed males from the tanks. Throughout the breeding period, all fish experienced the same conditions (e.g., temperature, light cycle, food access).

### 2.2 Offspring Experimental Setup

When fish were born in the breeding tanks, we transferred them that day (in a standardized manner) to their observation tanks (Figure 1C). We first herded them by net to a small container of water, then moved them to their respective individual tanks, limiting air exposure. We took individuals from each brood and distributed them evenly between observation tanks that were part of two closed circulating water systems: one containing a live electric yellow lab cichlid ( *Labidochromis caeruleus* ), and one without (control). Each closed water system was connected to a sump tank below the observation tanks; in the predator system, this sump tank contained the cichlid, which was fed whole Tetra‐Min flake food, blood worms, and brine shrimp daily. All tanks in a given filtration system shared water, so fish in the predator system were consistently receiving chemical (but not visual) cues from the cichlid—this species is not a sympatric predator of Amazon mollies, but is still recognized by Amazons as a predator, as their chemical cues elicit anti‐predator behaviour in adult Amazons (Aguiñaga et al. 2024). We used a single individual to generate predator cues, as Amazons respond similarly to cues of different individuals of this cichlid species (Aguiñaga et al. 2024). To deliver alarm cues to experimental fish, which are released from the skin following mechanical damage (Wisenden et al. 2009; Wisenden 2014, 2019; Chivers and Smith 1998), we fed the cichlid an anaesthetised small black molly ( *Poecilia sphenops* ) twice per week—once between Monday and Wednesday, and once between Thursday and Friday—on random days during those two windows and at random times of day to reduce habituation of fish to alarm cues. Experimental conditions for offspring were identical to those used for tracking the behaviour of the mothers, except fish were fed powdered food. Tanks contained aquarium gravel along the walls to help promote natural foraging behaviours, and each group of tanks was surrounded by curtains to reduce disturbance. There were always multiple individuals in a given shared water system, so fish did not perceive complete social isolation (Bierbach et al. 2017; Laskowski, Bierbach, et al. 2022), which can lead to altered behaviours in some fish (Franck et al. 1985). We allowed fish to develop in their observation tanks undisturbed for 4 weeks, resulting in data from 107 individuals. We tracked fish using the machine learning framework SLEAP (Pereira et al. 2022) (see Supporting Information: Methods for details).

### 2.3 Statistical Analysis

All statistical analysis was conducted using R accessed through R Studio (R Studio version 2023.12.1, R version 4.3.3). We selected which behaviours we would investigate by eliminating highly correlated behaviours (> 0.8). This resulted in two behaviours, median velocity (swimming speed) and proportion of time spent near the standpipe (refuge use), that we used for analysis. In order to understand which factors shape mean‐level behaviours and behavioural variance, we first ran Bayesian bivariate mixed models using the MCMCglmm package (version 2.36; Hadfield 2010), separately for each behaviour (velocity and refuge use) where our bivariate response was the same behaviour in each treatment. We used a bivariate model first to determine whether or not variance attributed to individual identity and maternal identity should be estimated independently by treatment. All models included treatment (with or without predator exposure), day of life, hour, and the interaction between treatment and day as fixed effects, and individual and maternal ID as random effects because these were both of primary interest to our research question. We first conducted model selection to determine any additional random effects structure of our models (i.e., inclusion of random intercepts and slopes over time for individual and/or mother). For all model selection in this study, we chose the least complex model within 2 of the lowest DIC value. To account for differences in behaviour due to time of year, we next compared our models with and without a time of year variable as a fixed effect. We performed hierarchical clustering using the stats package (version 4.3.3) to translate the time of year variable into batches; this revealed four primary clusters, which we used for our time of year batch variable. The best supported models for both behaviours included random effects (individual and maternal IDs) pooled between treatments, as opposed to independent random effects that were estimated uniquely for each treatment (i.e., estimating variance separately by treatment did not reduce DIC by more than 2 relative to the simpler model with a single variance estimate across treatments). This suggested that variance components did not differ by treatment, and therefore a univariate model was most appropriate to answer our questions moving forward.

We used the same model selection process above to determine the best structure for the univariate model. We also compared our models to one that allowed for heterogeneous residual variance over time, as this could impact repeatability calculations and reveal cryptic yet relevant information about the effects of the developmental environment on behavioural variability. For both behaviours, heterogeneous residual variance improved the models' fit. Thus, the final model for velocity included treatment, day, their interaction, hour, and time of year as fixed effects, random intercepts for individual and maternal ID, and daily heterogeneous residual variation (family = gaussian, iterations = 500,000, burn‐in = 50,000, thinning = 100). For refuge use, the final model included treatment, day, their interaction, and hour as fixed effects (time of year did not improve the model fit), random intercepts for individual and maternal ID, and daily heterogeneous residual variation (family = gaussian, iterations = 500,000, burn‐in = 50,000, thinning = 100). All models were validated by ensuring variables of interest had low autocorrelation values (< 0.1) and high effective sample sizes (> 1500), and by visually inspecting trace and density plots. To aid with statistical interpretation of differences between treatment groups (shown in Supporting Information), we calculated probability of direction (*p*_d_) using the package bayestestR (a *p*_d_ value > 0.975 is equivalent to a frequentist *p*‐value of < 0.05, version 0.16.0; Makowski, Ben‐Shachar, and Lüdecke 2019; Makowski, Ben‐Shachar, Chen, and Lüdecke 2019).

To test for the effects of body size on behaviour, we ran the full models described above with and without the inclusion of individual standard length and width as fixed effects (family = gaussian, iterations = 50,000, burn‐in = 5000, thinning = 100). Because body size data were collected at the end of the experiment (day 27), any individuals that were not recorded through the end of the experiment could not have their body size measured, so we ran the models on a subset of the data that only included individuals with body size measurements (*N* = 90 individuals). See Supporting Information for body size measurement methods.

To test for differences in mean behaviours between treatments on day one of life, we ran the same models on a subset of the data (day one) but excluded day as a fixed effect. We again conducted model selection to determine which other fixed effects should be included for the day one comparison (time of year and hour). Our final model for velocity included treatment and hour as fixed effects, and random intercepts for individual and maternal ID, and for refuge use included treatment as a fixed effect and random intercepts for individual and maternal ID (for both behaviours: family = gaussian, iterations = 500,000, burn‐in = 50,000, thinning = 100).

To estimate the proportion of variance explained overall (i.e., over the entire observation period and across both treatments) by different sources, we calculated marginalized repeatability (i.e., intraclass correlation) by dividing the variance attributed to each variable (individual ID, maternal ID, and residual) by the total variance (Nakagawa et al. 2026; Schielzeth and Nakagawa 2022). For example, to calculate maternal ICC, we used the following equation: maternal ID variance / (maternal ID variance + individual ID variance + residual variance; see Supporting Information for further details). To determine whether maternal behaviour predicted offspring behaviour, we ran our full mixed model (as above), this time with the inclusion of maternal behaviour as a fixed effect (separately for velocity and refuge use) on a subset of individuals that had data for their own and their mother's behaviour (*N* = 86 individuals).

To more deeply investigate how exposure to predation may alter patterns of individual behaviour specifically, we ran separate univariate mixed models for each treatment (control, predator) and each behaviour, which allowed us to partition variance components (among‐ vs. within‐individual variance) and estimate behavioural repeatability, our proxy for individuality, within each treatment. We ran models separately by treatment because even though variance averaged over development did not differ by treatment, our aim here was to understand how multiple variance components, including within‐individual variation, changed throughout development, which our pooled treatment model above cannot estimate. As for the previous models, we also conducted model selection to determine the random and fixed effects structure. Because our goal was to compare control and predator models directly, we chose the most complex model structure that was the best fit for either treatment group, then used this structure for both models to ensure that important sources of variation were not excluded. The best supported model structure for velocity included developmental day, hour, and time of year as fixed effects, random intercepts and slopes for individual ID (across days), random intercepts and slopes for maternal ID (across days), and daily heterogeneous residual variances. For refuge use, the best supported models included developmental day and hour as fixed effects, random intercepts and slopes for individual ID, random intercepts for maternal ID, and daily heterogeneous residual variances (for both behaviours: family = gaussian, iterations = 1,500,000, burn‐in = 150,000, thinning = 200). Models were again validated by ensuring variables of interest had low autocorrelation and high effective sample sizes.

Estimating variance components and repeatability across time series data is challenging. When random slopes are present, meaning individuals are changing rank‐order over time, conditional repeatability can be used to estimate the magnitude of among‐individual variance at any given time point (Nakagawa et al. 2026; Schielzeth and Nakagawa 2022); however, in these models, residual (i.e., within‐individual) variance is typically described by a single component regardless of time. Given growing evidence of the importance of within‐individual behavioural variance (Alonzo 2015; Westneat et al. 2013, 2015), we chose to use heterogeneous residual models that can estimate within‐individual variance at a given time point. Indeed, in our study, the best supported models (as described above) included both random intercepts, slopes and heterogeneous residual variance. Therefore, we expanded the calculations of conditional repeatability proposed by Schielzeth and Nakagawa (2022) and Nakagawa et al. (2026) to also account for heterogeneous residual variance, estimated separately for each day. Our final repeatability equation included the variance attributable to individual intercepts ($\sigma_{\alpha 0}^{2}$), slopes ($\sigma_{\alpha 1}^{2}{\left(x_{1}^{*}\right)}^{2}$), their covariance ($2\sigma_{\alpha 0\alpha 1}x_{1}^{*}$), and time‐specific heterogeneous residual variance ($\sigma_{\epsilon_{x^{*}}}^{2}$):

$${R_{c}}_{x^{*}}=\frac{\sigma_{\alpha 0}^{2}+\sigma_{\alpha 1}^{2}{\left(x_{1}^{*}\right)}^{2}+2\sigma_{\alpha 0\alpha 1}x_{1}^{*}}{\sigma_{\alpha 0}^{2}+\sigma_{\alpha 1}^{2}{\left(x_{1}^{*}\right)}^{2}+2\sigma_{\alpha 0\alpha 1}x_{1}^{*}+\sigma_{\epsilon_{x^{*}}}^{2}}$$

For all the models above, we used parameter‐expanded priors from similar studies (Dingemanse and Dochtermann 2013; Laskowski, Bierbach, et al. 2022); we also verified that these expanded priors were not biasing our interpretation (using MCMCglmm default priors did not change results). To investigate the effects of treatment and maternal identity on offspring body size, we used mixed models (separately for length and width) with body size as our dependent variable, treatment and time of year as fixed effects, and maternal ID as a random effect (family = gaussian).

## 3 Results

### 3.1 Predation Stress During Development Impacts Mean‐Level Behaviour

**FIGURE 2:**
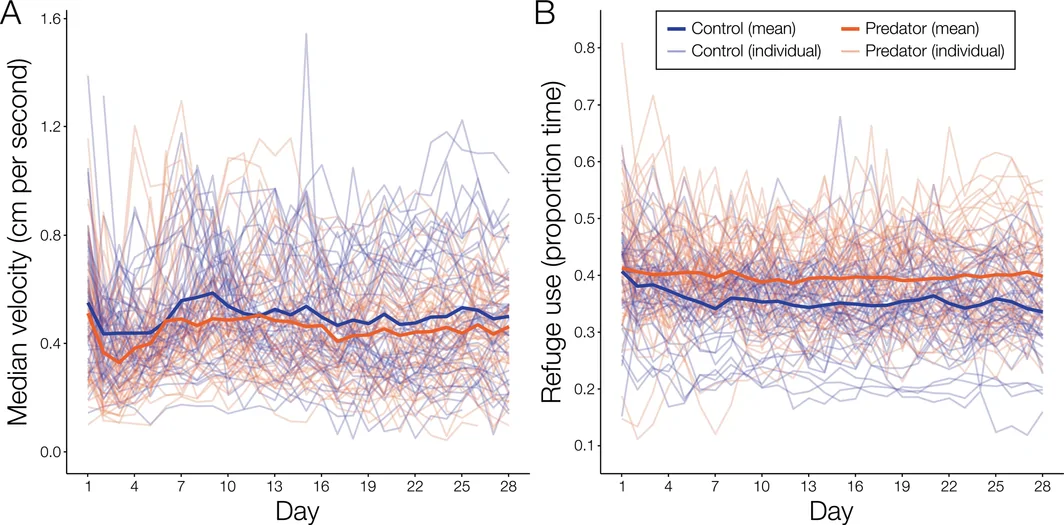
Average‐level behaviours (velocity (A), shown in cm per second, and refuge use (B), shown in proportion of time near the refuge) of individuals over developmental time, reared with (orange) and without (blue) predation threat. Thin lines represent the behaviours of individuals over time, while thick lines represent mean behaviours of individuals in a given developmental treatment. Note that for median velocity (A), a single individual's day one data is not shown (but is included in analysis) due to its unusually high value that would hinder visual clarity of the plot if included.

Our first question was to assess how mean‐level behaviours shifted as a result of experiencing predation stress during early life. We did not find evidence for significant treatment‐by‐time interactions in either swimming velocity (treatment × time: post.mean = 0.002 [95% CI: −0.01, 0.01]; Figure 2A) or refuge use (treatment × time: post.mean = 0.01 [95% CI: −0.004, 0.02]; Figure 2B), indicating that behaviours did not change differently over ontogeny based on environment. However, we did find significant main effects of treatment for both swimming velocity (post.mean = 0.19 scaled pixels per second [95% CI: −0.34, −0.02]) and refuge use (post.mean = 0.24 scaled proportion [95% CI: 0.03, 0.43]), with predator‐reared fish moving more slowly and spending more time near their refuge (Figure 2). For velocity, these differences between the two treatments appeared small, but were present on the first day of life (post.mean = 1.21 scaled pps [95% CI: 0.74, 1.75]). Unlike velocity, there were no significant differences in refuge use on day one (post.mean = 0.22 scaled proportion [95% CI: −0.01, 0.48]). Neither velocity nor refuge use were predicted by body size (Table S1), and body size was not predicted by rearing environment (length: post.mean = 0.06 [95% CI: −1.71, 1.67]; width: post.mean = −0.16 [95% CI: −0.45, 0.10]).

### 3.2 Different Behaviours Have Different Sources of Variance

**FIGURE 3:**
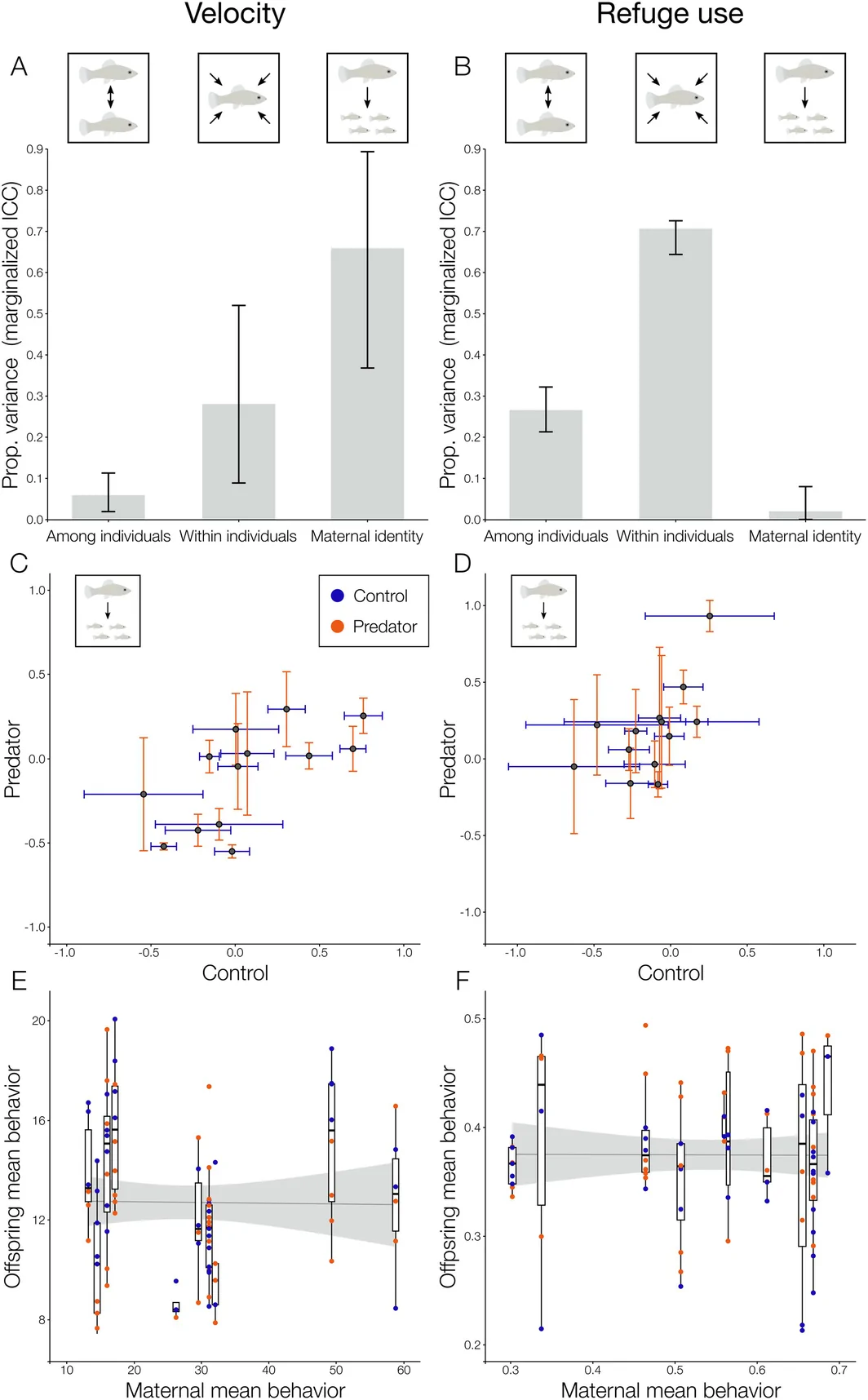
Sources of variation across behaviours (left = velocity, right = refuge use). A and B show intraclass correlations averaged across development (marginalized ICC), with error bars indicating 95% credible intervals. For velocity (A), most of the variance was explained by maternal identity, providing strong evidence of maternal effects. For refuge use (B), maternal identity contributed minimally to variance, with variation among individuals explaining a much larger share, and variation within individuals (i.e., residual) explaining the largest amount. (C, D) The relationship between control and predator siblings from each mother (units = scaled behaviour). Grey dots indicate the mean behavioural values of all offspring from a given mother. Error bars show the standard error of means for control (blue) and predator (orange) offspring of that mother. Maternal identity is a much stronger predictor of velocity than refuge use, as indicated by the lack of overlap between offspring behaviour from different mothers (error bars). (E, F) The lack of association between maternal behaviour and offspring behaviour, for both velocity (E) and refuge use (F). Boxplots represent individual mothers, while blue and orange dots show mean behaviours over developmental time for each individual offspring of that mother, reared with (orange) or without (blue) exposure to predator cues.

We used a combination of model selection and variance partitioning to determine how developmental environment (treatment), among‐individual differences, and/or maternal identity explained behavioural variance. Using model selection, we found that shared variance components across treatments were best supported for both velocity and refuge use, indicating that there were not significant differences in the magnitude of among‐individual and among‐mother variance between the treatments (see Supporting Information). For velocity, maternal identity explained the majority of the total variance across all of development (ICC = 0.659 [0.368, 0.893]), while differences among individuals explained a much lower amount (ICC = 0.059 [0.020, 0.113]; Figure 3A). However, for refuge use, patterns were reversed, with among‐individual differences (ICC = 0.267 [0.214, 0.323]) explaining much more of the variance than maternal identity (ICC = 0.021 [< 0.001, 0.081]), which was minimal (Figure 3B).

Because maternal identity strongly contributed to variation in velocity (Figure 3C; but not refuge use, Figure 3D), we tested whether these effects could be explained by maternal behaviour. For both velocity and refuge use, maternal behaviour did not predict offspring behaviour (velocity: post.mean = 0.005 [95% CI: −0.02, 0.03]; refuge use: post.mean = 0.02 [95% CI: −0.128, 1.41]), nor did maternal identity predict body size (length: post.mean = 0.546 [95% CI: < 0.001, 3.926]; width: post.mean = 0.004 [95% CI: < 0.001, 0.021]; Figure 3E,F).

### 3.3 Individuality Persists Despite Predation Stress During Development

**FIGURE 4:**
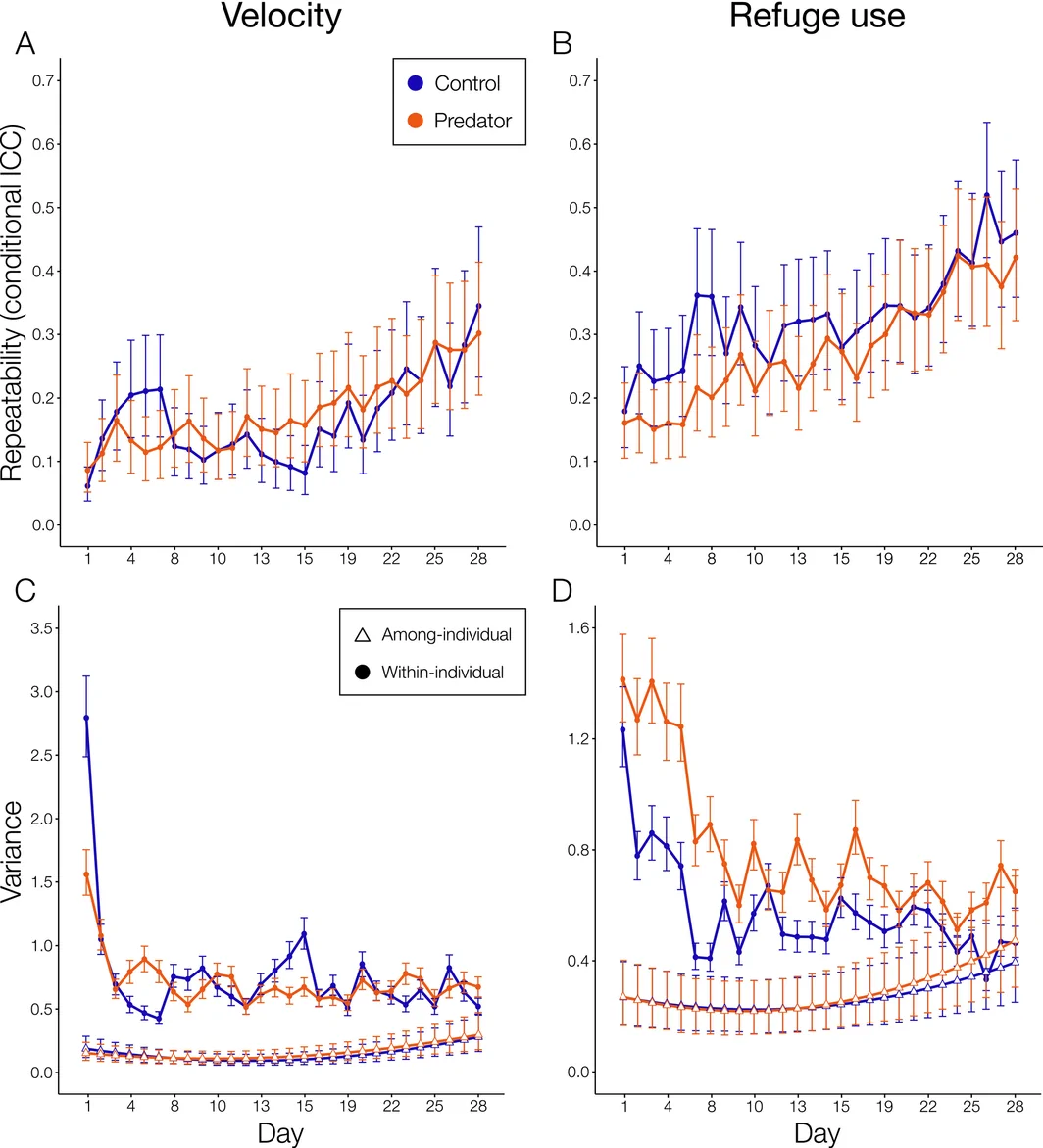
Extracted variance components over developmental time, coloured by treatment. Repeatability—the proportion of variance attributed to variation between individuals (the metric of individuality)—increased over the first month of life for velocity (A) and refuge use (B), and this pattern did not differ between treatments, indicating the persistence of individuality in the face of predation stress. (C, D) Variance components as posterior medians, separated into among‐ and within‐individual components. For velocity (C), both among‐ and within‐individual variance did not change over time or between treatments, aside from day one of life, where within‐individual variance was much higher, particularly in the control group. For refuge use (D), among‐individual variance also did not differ by treatment. Within‐individual variance was highest during the first week of life, followed by a sharp decrease and plateau for the rest of development. However, within‐individual variance was consistently greater in predator‐reared fish over developmental time. Error bars on all plots indicate 95% credible intervals.

We used generalized linear mixed models to explore patterns of individual behavioural variation, estimating variance components at each day (among‐ and within‐individual) to then calculate repeatability of behaviour over developmental time, our proxy for individuality, for each treatment separately, while controlling for among‐mother variance. We found that for both velocity and refuge use, individuality persisted, regardless of environmental treatment (Figure 4A,B). In both treatments, individuals began life with low repeatability, which gradually increased over the next 4 weeks, resulting in individuals with fairly repeatable behaviours by the time they were 1 month old. Refuge use was consistently more repeatable than velocity. Notably, repeatability and among‐individual variance did not differ by developmental treatment, as indicated by overlapping credible intervals. However, for refuge use, within‐individual variance was greater in predator‐reared individuals (Figure 4D), indicating that predator‐reared fish are less predictable in this behaviour, within any given day compared to control‐reared fish.

## 4 Discussion

We found that both developmental environment (Figure 2) and maternal effects (Figure 3) are sufficient to generate behavioural variation, even in the absence of genetic differences. However, the relative contribution of each source differed dramatically between behaviours, indicating distinct developmental mechanisms in response to ecological stress. Perhaps surprisingly, ecological stress did not disrupt the emergence of behavioural individuality across development, suggesting that individuality is seeded before birth rather than developmentally plastic in response to the environment (Figure 4A,B). However, individuals reared under stress did show greater variability (within‐individual variation) in their refuge use behaviour, indicating that stress does increase intraindividual variability or disrupt developmental canalization (Figure 4D). Here, we discuss the implications of how developmental environment shapes behaviour and individuality, the role of other factors—notably maternal effects—in explaining this variation, and the contrasting mechanisms underlying different behaviours. Finally, we discuss possible limitations, place our findings in the context of other recent studies, and propose directions for future research.

Exposure to predation during development impacted mean‐level behaviour, with predator‐reared individuals moving more slowly and spending more time near their refuges, showing that our animals responded to the cues of predation stress as we expected (Figure 2)—although for velocity these differences were relatively modest. This aligns with work in various non‐clonal species showing that exposure to predation during development can significantly shape behaviour (Bell and Sih 2007; Bucklaew and Dochtermann 2021; Płaskonka et al. 2024; Scherer et al. 2024; Tariel et al. 2020; Urszán et al. 2018). Indeed, species across taxa have been shown to decrease activity and increase refuge in response to predation, as these behavioural changes may reduce potential detection (Endler 1986). Despite this logic, work in Trinidadian guppies (congeneric with Amazon mollies) found that bolder and more active individuals in fact survived longer when faced with a predator (Smith and Blumstein 2010), so whether the behavioural shifts that we saw here are truly adaptive remains unclear.

In both environmental conditions, repeatability increased over ontogeny, suggesting that individuality crystallizes over time potentially through self‐reinforcing behavioural patterns or internal feedback loops (Sih et al. 2015; Laskowski, Bierbach, et al. 2022; Dall et al. 2004). Perhaps surprisingly, this strengthening of individuality over time was not impacted by predation, with repeatability increasing similarly over development in both treatments (Figure 4A,B). This indicates that the seeds for individuality may be set before birth, and suggests that there may be many optimal behavioural strategies to overcome environmental challenges. While our results are contrary to other studies where exposure to predation either suppressed or expanded among‐individual variance (e.g., Ehlman et al. 2019; Scherer et al. 2024; Toscano et al. 2014), our work measured behaviour continuously over development, rather than at set intervals which can only provide snapshots of behaviour. This approach allowed us to estimate individual variance components daily, improving the accuracy of our repeatability calculations and revealing additional insights into how behavioural variation emerges throughout ontogeny (Dupont et al. 2024). One example of this can be seen in our estimates of behavioural predictability (i.e., within‐individual variance or intraindividual variability; Biro and Adriaenssens 2013; Stamps et al. 2012). Although among‐individual variance did not differ between treatments, we did uncover temporal and treatment‐level changes in intraindividual variability by allowing residual variance to fluctuate over time in our model (i.e., heterogeneous residual variance). This revealed that while individuals in both treatments behaved more predictably as they aged, predator‐reared fish showed lower predictability (i.e., greater intraindividual variability) in refuge use within a given day throughout development (Figure 4D). Lower predictability in predator‐reared individuals may reflect either an adaptive increase in flexible use of the refuge throughout the day as individuals assess immediate risk, or a disruption of developmental canalization in response to risk. We encourage others to account for heterogeneous residual variance over time when supported by the data, as this not only provides useful insights into the often underappreciated yet important within‐individual variation (Alonzo 2015; Biro and Adriaenssens 2013; Dingemanse and Dochtermann 2013; Mitchell et al. 2021; Westneat et al. 2015), but also directly impacts the calculation of repeatability (which is dependent on correctly estimating residual variance at a given time point).

If individuality remains robust throughout environmental stress, what then drives it? We found that despite attempting to minimize maternal effects, for velocity, maternal identity predicted offspring behaviour more so than any other component (Figure 3A). This study was not designed to test for the effects of differential maternal experience on offspring behaviour because mothers used in this experiment were reared in similar conditions. Nevertheless, our results suggest that maternal effects play a notable role in shaping behaviour and may be a key candidate for setting the seeds of individuality before birth. Despite this strong evidence of maternal effects, we did not see any association between maternal and offspring behaviour (Figure 3E,F), indicating that the influence of mothers was not mediated by what they did, but rather by who they were (although maternal behaviour was collected when fish were adults rather than during early development). This points to maternal effects with a non‐behavioural basis, such as maternal provisioning, physiological state, and/or inherited epigenetic variation through changes in DNA methylation or maternal hormone transfer (Ensminger et al. 2018; Laskowski, Bierbach, et al. 2022; Mousseau et al. 2009; Mousseau and Fox 1998; Richards 2006), as mechanisms through which behavioural variation emerges, even in the absence of genetic or environmental variation. Individuality may begin with cryptic differences among mothers that bias developmental trajectories from the very start of life.

Interestingly, our two representative behaviours, velocity and refuge use, emerged differently over ontogeny and appeared driven by different factors, suggesting contrasting underlying mechanisms. Variation in velocity was overwhelmingly explained by maternal identity (Figure 3A), while variation in refuge use was not explained by maternal identity at all (Figure 3B). As for developmental environment, differences between treatments were present on day one of life for velocity, but not for refuge use, which emerged over the first week of life. Additionally, predation stress increased behavioural variability (within‐individual variance) for refuge use, but not velocity. These differences suggest that important ecologically relevant behaviours can have different mechanisms of emergence. Behaviours relating to space or refuge use may be far more context‐dependent than swimming speed. For example, while individual humans have highly consistent walking speeds (Veilleux et al. 2011), decisions about where and when to move, such as crossing a street, vary much more within individuals based on context (e.g., how much traffic is present). Given that refuge use differed more clearly among treatments than velocity (Figure 2A), it's possible that behaviours relating to space use may be more direct indicators of anti‐predator responses than activity and movement related behaviours. This is supported by work in other Poecilid fish showing change in space use, but not movement latency, in response to conspecific alarm cues (Crane et al. 2022). Even fully understanding the developmental processes underlying one suite of behaviours may not inform others, and we should be careful to not generalize across traits when studying the mechanisms of behaviours.

Although we attempted to control for as many variables as possible, there remain potential limitations. Our study focused on isolating the effects of the environment on behavioural development, so we minimized genetic variation and carefully controlled experimental environments. Nevertheless, small genetic differences between individuals could arise even within a clonal lineage (Lushai et al. 1998), and experiences are impossible to fully standardize from the first second of life.

We end this by placing our results in the context of other studies and proposing future research. First, our repeatability estimates differed from those in other work on Amazon mollies. Repeatability increased over ontogeny, with refuge use reaching higher levels after 4 weeks (> 0.5). However, for velocity, repeatability remained fairly low (under 0.2) until after week three. Such values differ from previous work in this species, where repeatability of velocity was > 0.4 throughout the first month of development (measured only in control conditions; Laskowski, Bierbach, et al. 2022), and instead more closely matched levels found in other taxa (e.g., crickets) when using inbred lines (Sweep et al. 2025). One likely reason for the discrepancy is that here we had a larger number of individuals and mothers compared to other related studies in Amazons, allowing us to more accurately account for variance that was attributed to differences between mothers (Figure 3A). While repeatability remained relatively low for velocity over the first 3 weeks, there was a greater rate of increase over the final week of the experiment, a pattern that mirrors results from the aforementioned work on Amazons (Laskowski, Bierbach, et al. 2022). If that trajectory were to continue, repeatability in our fish may have reached much higher levels over time (as in Laskowski, Bierbach, et al. 2022).

The persistence of among‐individual variation, despite a lack of genetic differences, raises additional questions about the role that allelic variation plays in generating individuality. One hallmark of sexual reproduction is its ability to generate variation among individuals. Yet here (and in other recent work; Bierbach et al. 2017; Laskowski, Bierbach, et al. 2022) we found a striking degree of among‐individual variation even when genetic differences were minimized. Future work could reintroduce genetic variation in a standardized environment to better understand the contribution of allelic variation (or lack thereof) to the onset of individuality (e.g., comparing Amazon mollies to their parental species, which naturally should contain more among‐individual genetic variation via sexual reproduction). For example, a recent study found reduced repeatability in some inbred lines of crickets (Sweep et al. 2025). Whether this pattern holds true in other taxa—and in clonal species like the Amazon molly—would make for interesting future work. Other ecologically important environmental factors, such as temperature (Briffa et al. 2013; Hageter et al. 2021; Nakayama et al. 2016) or resource availability (Honegger et al. 2020), could also be systematically manipulated to further assess their impact on individuality. Given our evidence for maternal effects, an important next step is to explore how maternal environment shapes offspring behaviour using a multigenerational design testing the impacts of maternal experience on mechanisms that canalize individual behavioural trajectories.

In this study, we disentangled the developmental drivers of individuality to understand the mechanisms that make individuals unique. Despite facing a strong environmental stressor that affected mean‐level behaviours, individuality persisted, suggesting that development does not result in a single dominant phenotype, but rather an acceptable range. Even though mean values may be shifted up or down, the magnitude of variation among individuals does not change—behavioural individuality appears to be a robust and intrinsic feature of animal life.

## Author Contributions

Conceptualization: Kate L. Laskowski, Chia‐Chen Chang, James H. Gallagher and Ammon D. Perkes. Methodology: James H. Gallagher, Ammon D. Perkes, Kate L. Laskowski and Chia‐Chen Chang. Validation: James H. Gallagher and Ammon D. Perkes. Formal analysis: James H. Gallagher, Ammon D. Perkes and Kate L. Laskowski. Investigation: James H. Gallagher, Emma S. Chirila and Karen Kacevas. Data curation: James H. Gallagher. Writing – original draft: James H. Gallagher. Writing – reviewing and editing: James H. Gallagher, Ammon D. Perkes, Chia‐Chen Chang, Emma S. Chirila, Karen Kacevas and Kate L. Laskowski. Visualization: James H. Gallagher. Supervision: Kate L. Laskowski. Project administration: James H. Gallagher and Kate L. Laskowski. Funding acquisition: Kate L. Laskowski.

## Funding

This work was supported by the National Science Foundation (IOS2100625) and Alfred P. Sloan Foundation.

## Conflicts of Interest

The authors declare no conflicts of interest.

## Supporting information


**Table S1:** Outputs for the mixed models showing that body size components did not predict behaviours (velocity, refuge use).

**Data S1:** Supplemental code for reproducing results.

## Data Availability

Data and code are available in the public data sharing repository, Dryad: https://doi.org/10.5061/dryad.5mkkwh7jx.
