# Supplementary material for: Born This Way: Individuality Is Seeded Before Birth and Robust to Ecological Stress
Source: Ecol Lett. 2026 Sep 14;29(9):e70454. doi: 10.1111/ele.70454 (PMC13574246; doi:10.1111/ele.70454)
Supplement: Supplementary file 1 [file ELE-29-0-s002.pdf]

Supplemental code for 'Born this way: individuality is seeded before birth and robust to ecological stress'

James H. Gallagher

2026-06-26

Results were produced using R version 4.3.3

Important metadata for data file

FishId: The unique identifier of the individual fish.

MomId: The unique identifier of the individual's mother.

PredatorCue: Whether or not the fish was reared with predator cues. 0 = no, 1 = yes.

Hour: The hour of the day that data was collected for that fish on that date.

ExpDay: Day of the fish's life, starting at 0.

YearDay: Calendar year day, used to test for time of year effects in the models.

MedianVel: Median velocity (swimming speed).

MeanAct: Proportion of time that the fish was active, meaning she was moving.

MeanCorner: Proportion of time that the fish spent in the area that has the refuge (standpipe).

MedianActiveVel: Median velocity of fish when it was moving.

MeanVis: The proportion of time the fish was visible for tracking. Used for tracking validation.

Any metric with an underscore (_) at the end of its name represents hourly data; without underscore represents daily means. Most analyses use hourly data, with daily means used more for visualizing across days.

Initial setup

Make sure you have downloaded the necessary model (.rds) and data (.csv) files and put them in your working directory in order to run this script.

Load required libraries

```
library(Matrix)
library(lme4)
library(lmerTest)
```

```
##
## Attaching package: 'lmerTest'

## The following object is masked from 'package:lme4':
##
##     lmer

## The following object is masked from 'package:stats':
##
##     step
```

```r
library(carData)
library(car)
library(ggplot2)
library(dplyr)
```

```
##
## Attaching package: 'dplyr'
## The following object is masked from 'package:car':
##
##     recode
## The following objects are masked from 'package:stats':
##
##     filter, lag
## The following objects are masked from 'package:base':
##
##     intersect, setdiff, setequal, union
```

```r
library(stats)
library(MCMCglmm)
```

```
## Loading required package: coda
## Loading required package: ape
##
## Attaching package: 'ape'
## The following object is masked from 'package:dplyr':
##
##     where
```

```r
library(brms)
```

```
## Loading required package: Rcpp
## Loading 'brms' package (version 2.22.0). Useful instructions
## can be found by typing help('brms'). A more detailed introduction
## to the package is available through vignette('brms_overview').
##
## Attaching package: 'brms'
## The following object is masked from 'package:MCMCglmm':
##
##     me
## The following object is masked from 'package:lme4':
##
##     ngrps
## The following object is masked from 'package:stats':
##
##     ar
```

```r
library(performance)
library(emmeans)
```

```
## Welcome to emmeans.
## Caution: You lose important information if you filter this package's results.
## See '? untidy'
```

```r
library(coda)
library(DHARMa)
```

```
## This is DHARMa 0.4.7. For overview type '?DHARMa'. For recent changes, type news(package = 'DHARMa')
```

```r
library(bayestestR)
library(tidyr)
```

```
##
## Attaching package: 'tidyr'

## The following objects are masked from 'package:Matrix':
##
##     expand, pack, unpack
```

```r
library(styler)
library(tinytex)
library(formatR)
```

Load offspring data

```r
### Data are available for download on Dryad
hourly_data <- read.csv("../Submission items/data_for_gallagher_et_al_2026.csv")
```

Data curation

Make sure ExpDay is numeric:

```r
hourly_data$ExpDay <- as.numeric(hourly_data$ExpDay)
```

Make batches (YearBatch) by YearDay using hierarchical clustering

```r
year_days <- sort(unique(hourly_data$YearDay))
year_days_matrix <- as.matrix(year_days)
cluster <- hclust(dist(year_days_matrix))
plot(cluster)
```

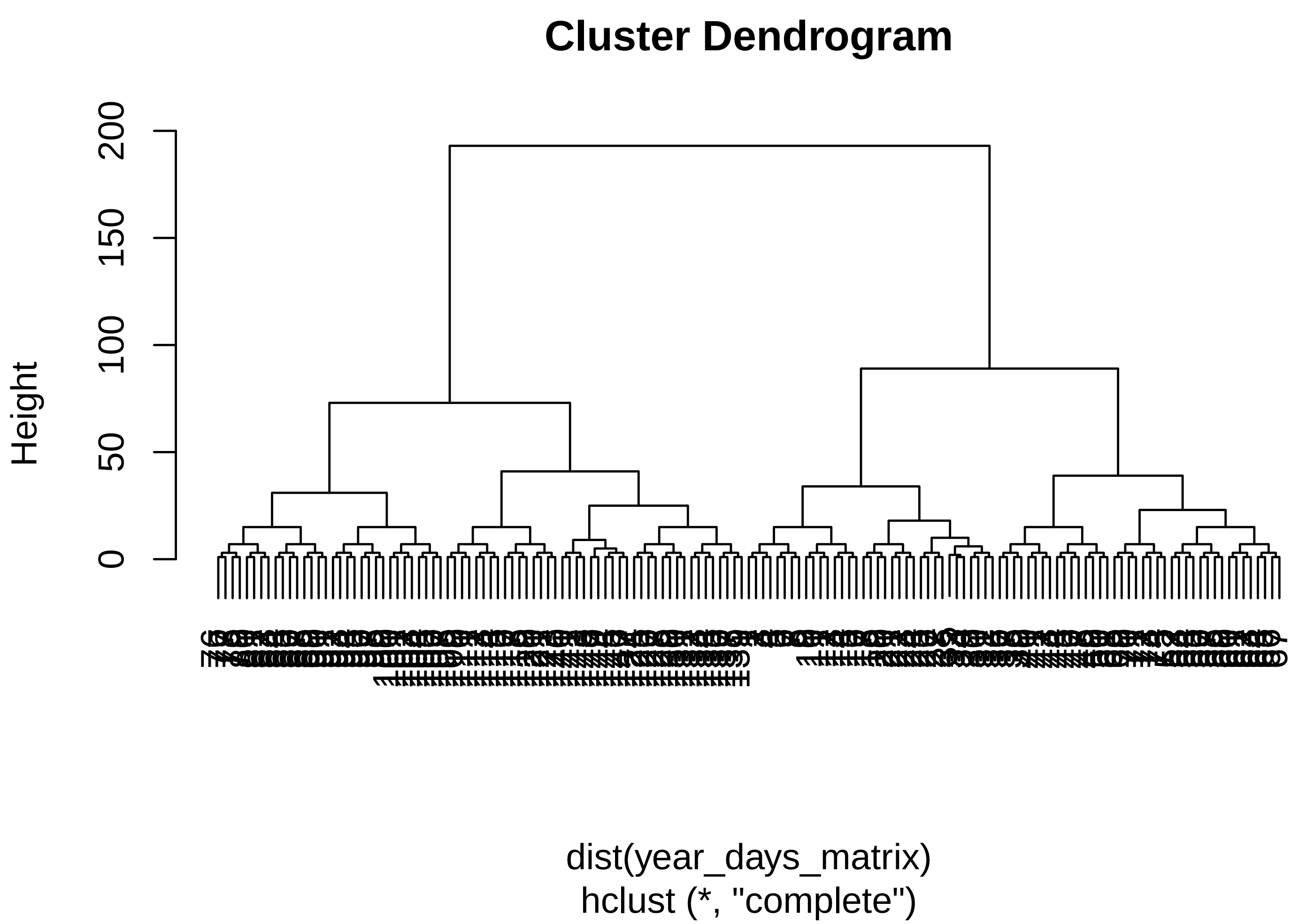


```
k <- 4  # 4 batches looks most appropriate
batches <- cutree(cluster, k)
hourly_data$YearBatch <- batches[match(hourly_data$YearDay, year_days)]
```

Make additional subsets for analysis

Scale hourly data:

```
hourly_data$MedianVel_hourly_scaled <- scale(hourly_data$MedianVel_)
hourly_data$MeanCorner_hourly_scaled <- scale(hourly_data$MeanCorner_)
```

Make daily data set:

```
day_only_data <- subset(hourly_data, Hour == 0)
day_only_data <- day_only_data %>%
    distinct(FishId, ExpDay, .keep_all = TRUE)
```

Scale daily (non-hourly) data:

```
day_only_data$MedianVel_scaled <- scale(day_only_data$MedianVel)
day_only_data$MeanCorner_scaled <- scale(day_only_data$MeanCorner)
```

Make data sheet that only includes fish with bodysize data:

```
bodysize_only_data <- subset(day_only_data, !is.na(avg_width))
```

Make data sheet that only includes fish with mom behavior data:

```
mombehavior_only_data_hourly <- subset(hourly_data, !is.na(Avg_MedVel_mom))
```

Check sample sizes

```
number_of_babies <- length(unique(hourly_data$FishId))
number_of_babies
```

```
## [1] 106
```

```
number_of_control_babies <- length(unique(day_only_data$FishId[day_only_data$PredatorCue ==
    0]))
number_of_predator_babies <- length(unique(day_only_data$FishId[day_only_data$PredatorCue ==
    1]))
number_of_control_babies
```

```
## [1] 51
```

```
number_of_predator_babies
```

```
## [1] 55
```

```
number_of_moms <- length(unique(hourly_data$MomId))
number_of_moms
```

```
## [1] 13
```

Preliminary analysis

Daily behavioral correlations

```
pairs(day_only_data[, c("MedianVel", "MeanAct", "MeanCorner",
    "MedianActiveVel")])
```

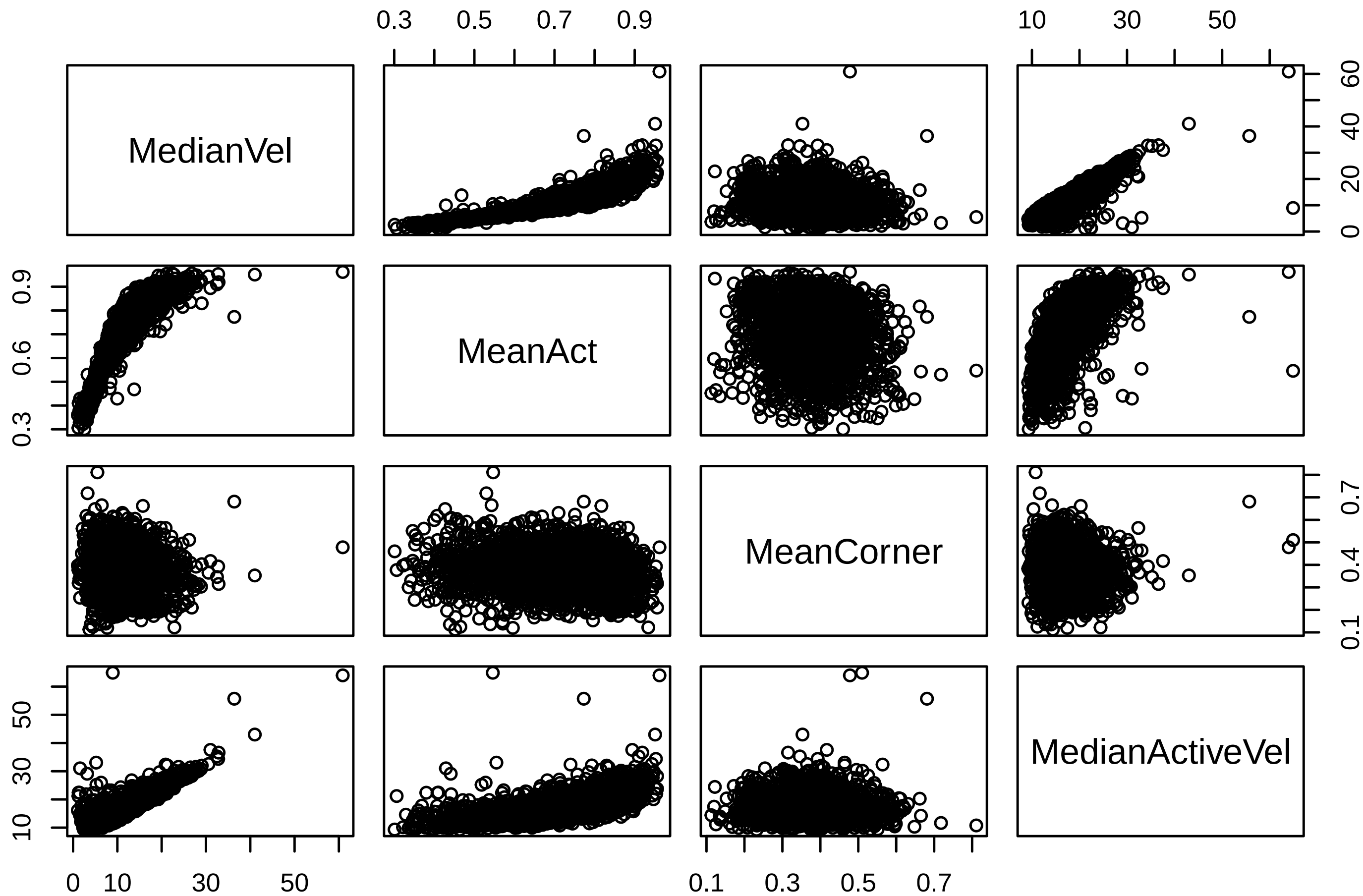

```r
cor(day_only_data[, c("MedianVel", "MeanAct", "MeanCorner", "MedianActiveVel")])
```

```
##                   MedianVel    MeanAct  MeanCorner MedianActiveVel
## MedianVel        1.00000000  0.8845265 -0.08292711      0.85265989
## MeanAct          0.88452653  1.0000000 -0.11617562      0.62883705
## MeanCorner      -0.08292711 -0.1161756  1.00000000     -0.01758919
## MedianActiveVel  0.85265989  0.6288370 -0.01758919      1.00000000
```

Mean activity and median active velocity are highly correlated with median velocity, so we will only analyze median velocity and refuge use (MeanCorner).

Analysis

Analysis pipeline overview for each behavior:

1. Mean-level differences in behavior 1a. Data preparation for bivariate model. 1b. Model selection. 1c. Model output. 1d. Plotting velocity over time by treatment.
2. Maternal effects 2a. Model output linking maternal and offspring behavior. 2b. Plotting maternal by offspring behavior. 2c. Plotting the range of offspring behavior by treatment per mother ID. 2d. Calculate proportion of variance attributed to maternal ID.
3. Estimating variance components over time 3a. Data preparation for univariate models. 3b. Model selection. 3c. Estimating conditional repeatability, among-, and within-individual variance components. 4d. Plotting variance components over time for each treatment.
4. Effect of maternal ID and treatment on body size
5. Effect of semi-weekly alarm cues on behavioral means and variance

Median velocity

Mean effect of treatment

Prepare data for bivariate model

We need to create columns for control and predator treatments:

```r
hourly_data$MedianVel_control <- ifelse(hourly_data$PredatorCue ==
    0, hourly_data$MedianVel_hourly_scaled, NA)
hourly_data$MedianVel_predator <- ifelse(hourly_data$PredatorCue ==
    1, hourly_data$MedianVel_hourly_scaled, NA)
```

Model comparison to determine model structure

We will conduct model comparison to determine the best fit random effect structure. First we will set priors.

Set priors

We need to set expanded priors for all of our models:

```r
# Null
prior.null <- list(R = list(V = diag(2), nu = 0.002))

# FishId
prior.FishId <- list(R = list(V = diag(2), nu = 0.002), G = list(G1 = list(V = diag(2),
    nu = 0.002, alpha.mu = rep(0, 2), alpha.V = diag(25^2, 2))))

# MomId
prior.MomId <- list(R = list(V = diag(2), nu = 0.002), G = list(G1 = list(V = 1,
    nu = 0.002, alpha.mu = 0, alpha.V = 25^2)))

# FishId + MomId
prior.FishId_MomId <- list(R = list(V = diag(2), nu = 0.002),
    G = list(G1 = list(V = diag(2), nu = 0.002), G2 = list(V = 1,
```

```r
        nu = 0.002)))

# FishId + MomId independent variance across treatments (no
# covariance)
prior.FishId_MomId_idh <- list(R = list(V = diag(2), nu = 0.002),
    G = list(G1 = list(V = diag(2), nu = 0.002), G2 = list(V = diag(2),
        nu = 0.002)))

# FishId + MomId unstructured (covariance)
prior.FishId_MomId_unstructured_mom <- list(R = list(V = diag(2),
    nu = 0.002), G = list(G1 = list(V = diag(2), nu = 0.002),
    G2 = list(V = diag(2), nu = 2)))

# FishId shared variance across treatments + MomId
prior.FishId_MomId_NOidh <- list(R = list(V = diag(2), nu = 0.002),
    G = list(G1 = list(V = 1, nu = 0.002), G2 = list(V = 1, nu = 0.002)))
```

Model comparison to determine included random effects

```r
# Null
model_null <- readRDS("model_MedianVel_null_bivariate_8_12_25.rds")
# model_null <- MCMCglmm(cbind(MedianVel_control,
# MedianVel_predator) ~ trait*ExpDay - 1 + YearBatch +
# Hour, rcov = ~idh(trait):units, family = c('gaussian',
# 'gaussian'), data = hourly_data, prior = prior.null, nitt
# = 200000, burnin = 20000, thin = 100, verbose = TRUE)
# saveRDS(model_null, file =
# 'model_MedianVel_null_bivariate_8_12_25.rds')
summary(model_null)
```

```
##
##  Iterations = 20001:199901
##  Thinning interval  = 100
##  Sample size  = 1800
##
##  DIC: 91773.9
##
##  R-structure:  ~idh(trait):units
##
##                                post.mean l-95% CI u-95% CI eff.samp
## traitMedianVel_control.units      1.0303   1.0070   1.0518     1800
## traitMedianVel_predator.units     0.9316   0.9121   0.9507     1923
##
##  Location effects: cbind(MedianVel_control, MedianVel_predator) ~ trait * ExpDay - 1 + YearBatch + Hour
##
##                                  post.mean   l-95% CI   u-95% CI eff.samp
## traitMedianVel_control           0.3133274  0.2733718  0.3536112     1800
## traitMedianVel_predator          0.0896844  0.0495568  0.1283541     1800
## ExpDay                          -0.0015236 -0.0035257  0.0003254     1800
## YearBatch                       -0.0067291 -0.0161758  0.0035770     1800
## Hour                            -0.0342316 -0.0375199 -0.0312931     1800
## traitMedianVel_predator:ExpDay   0.0040905  0.0015243  0.0066202     1800
##                                   pMCMC
## traitMedianVel_control          < 6e-04 ***
## traitMedianVel_predator         < 6e-04 ***
## ExpDay                          0.13778
## YearBatch                       0.19000
## Hour                            < 6e-04 ***
## traitMedianVel_predator:ExpDay 0.00333 **
## ---
## Signif. codes:  0 '***' 0.001 '**' 0.01 '*' 0.05 '.' 0.1 ' ' 1
```

```r
# FishId
model_id <- readRDS("model_MedianVel_id_bivariate_8_26_25.rds")
# model_id <- MCMCglmm(cbind(MedianVel_control,
# MedianVel_predator) ~ trait*ExpDay - 1 + YearBatch +
# Hour, random = ~idh(trait):FishId, rcov =
# ~idh(trait):units, family = c('gaussian', 'gaussian'),
# data = hourly_data, prior = prior.FishId, nitt = 200000,
# burnin = 20000, thin = 100, verbose = TRUE)
# saveRDS(model_id, file =
# 'model_MedianVel_id_bivariate_8_26_25.rds')
summary(model_id)
```

```
##
##  Iterations = 20001:199901
##  Thinning interval  = 100
##  Sample size  = 1800
##
##  DIC: 85528.49
##
##  G-structure:  ~idh(trait):FishId
##
##                                 post.mean l-95% CI u-95% CI eff.samp
## traitMedianVel_control.FishId      0.2037   0.1255   0.2854     1204
## traitMedianVel_predator.FishId     0.2106   0.1353   0.3058     1160
##
##  R-structure:  ~idh(trait):units
##
##                                post.mean l-95% CI u-95% CI eff.samp
## traitMedianVel_control.units      0.8326   0.8144   0.8521     1941
## traitMedianVel_predator.units     0.7800   0.7634   0.7972     1800
##
##  Location effects: cbind(MedianVel_control, MedianVel_predator) ~ trait * ExpDay - 1 + YearBatch + Hour
##
##                                  post.mean   l-95% CI   u-95% CI eff.samp  pMCMC
## traitMedianVel_control           0.6224631  0.4006650  0.8573835    813.8 <6e-04
## traitMedianVel_predator          0.4278803  0.2082326  0.6756425    690.3 <6e-04
## ExpDay                          -0.0013650 -0.0033252  0.0002864   2151.3  0.151
## YearBatch                       -0.1381021 -0.2223082 -0.0581732    769.3 <6e-04
## Hour                            -0.0344081 -0.0374541 -0.0317383   1800.0 <6e-04
## traitMedianVel_predator:ExpDay  0.0045399  0.0019917  0.0069413   1998.0 <6e-04
##
## traitMedianVel_control          ***
## traitMedianVel_predator         ***
## ExpDay
## YearBatch                       ***
## Hour                            ***
## traitMedianVel_predator:ExpDay ***
## ---
## Signif. codes:  0 '***' 0.001 '**' 0.01 '*' 0.05 '.' 0.1 ' ' 1
```

```r
# MomId
model_mom <- readRDS("model_MedianVel_mom_bivariate_8_26_25.rds")
# model_mom <- MCMCglmm(cbind(MedianVel_control,
# MedianVel_predator) ~ trait*ExpDay - 1 + YearBatch +
# Hour, random = ~MomId, rcov = ~idh(trait):units, family =
# c('gaussian', 'gaussian'), data = hourly_data, prior =
# prior.MomId, nitt = 200000, burnin = 20000, thin = 100,
# verbose = TRUE) saveRDS(model_mom, file =
# 'model_MedianVel_mom_bivariate_8_26_25.rds')
summary(model_mom)
```

```
##
```

```
##  Iterations = 20001:199901
##  Thinning interval  = 100
##  Sample size  = 1800
##
##  DIC: 88719.36
##
##  G-structure:  ~MomId
##
##       post.mean l-95% CI u-95% CI eff.samp
## MomId     1.223   0.3296    2.578     1800
##
##  R-structure:  ~idh(trait):units
##
##                                post.mean l-95% CI u-95% CI eff.samp
## traitMedianVel_control.units     0.9089   0.8880   0.9282     1791
## traitMedianVel_predator.units    0.8714   0.8532   0.8899     1800
##
##  Location effects: cbind(MedianVel_control, MedianVel_predator) ~ trait * ExpDay - 1 + YearBatch + Hour
##
##                                  post.mean   l-95% CI   u-95% CI eff.samp  pMCMC
## traitMedianVel_control           1.9655578  1.1839728  2.7572783     2439 <6e-04
## traitMedianVel_predator          1.7318562  0.9473263  2.5243084     2472 <6e-04
## ExpDay                          -0.0020195 -0.0039680 -0.0002245     1800 0.0367
## YearBatch                       -0.7659864 -1.0254033 -0.5434844     2060 <6e-04
## Hour                            -0.0344964 -0.0374030 -0.0314431     2596 <6e-04
## traitMedianVel_predator:ExpDay  0.0045174  0.0018900  0.0068888     1800 <6e-04
##
## traitMedianVel_control          ***
## traitMedianVel_predator         ***
## ExpDay                          *
## YearBatch                       ***
## Hour                            ***
## traitMedianVel_predator:ExpDay ***
## ---
## Signif. codes:  0 '***' 0.001 '**' 0.01 '*' 0.05 '.' 0.1 ' ' 1
```

```r
# FishId + MomId
model_id_mom <- readRDS("model_MedianVel_id_mom_bivariate_hour_8_11_25.rds")
# model_id_mom <- MCMCglmm(cbind(MedianVel_control,
# MedianVel_predator) ~ trait*ExpDay - 1 + YearBatch +
# Hour, random = ~idh(trait):FishId + MomId, rcov =
# ~idh(trait):units, family = c('gaussian', 'gaussian'),
# data = hourly_data, prior = prior.FishId_MomId, nitt =
# 1500000, burnin = 150000, thin = 400, verbose = TRUE)
# saveRDS(model_id_mom, file =
# 'model_MedianVel_id_mom_bivariate_hour_8_11_25.rds')
summary(model_id_mom)
```

```
##
##  Iterations = 150001:1499601
##  Thinning interval  = 400
##  Sample size  = 3375
##
##  DIC: 85473.25
##
##  G-structure:  ~idh(trait):FishId
##
##                                  post.mean l-95% CI u-95% CI eff.samp
## traitMedianVel_control.FishId     0.09003  0.05196   0.1355     3375
## traitMedianVel_predator.FishId    0.13377  0.07783   0.1940     3375
##
##                ~MomId
```

```
##
##       post.mean l-95% CI u-95% CI eff.samp
## MomId     1.279   0.2434     2.75     3375
##
##  R-structure:  ~idh(trait):units
##
##                               post.mean l-95% CI u-95% CI eff.samp
## traitMedianVel_control.units     0.8295   0.8122   0.8482     3375
## traitMedianVel_predator.units    0.7795   0.7632   0.7961     3375
##
##  Location effects: cbind(MedianVel_control, MedianVel_predator) ~ trait * ExpDay - 1 + YearBatch + Hour
##
##                                 post.mean   l-95% CI   u-95% CI eff.samp  pMCMC
## traitMedianVel_control          2.0542426  1.2653524  2.8243354     3375 <3e-04
## traitMedianVel_predator         1.8435364  1.0676376  2.6386976     3375 <3e-04
## ExpDay                         -0.0010726 -0.0029163  0.0006747     3375  0.245
## YearBatch                      -0.8028568 -1.0528943 -0.5972976     3375 <3e-04
## Hour                           -0.0343877 -0.0372379 -0.0316226     3790 <3e-04
## traitMedianVel_predator:ExpDay  0.0044915  0.0020934  0.0068283     3375 <3e-04
##
## traitMedianVel_control         ***
## traitMedianVel_predator        ***
## ExpDay
## YearBatch                      ***
## Hour                           ***
## traitMedianVel_predator:ExpDay ***
## ---
## Signif. codes:  0 '***' 0.001 '**' 0.01 '*' 0.05 '.' 0.1 ' ' 1
```

```
# FishId + MomId independence across treatments (but no
# covariance)
model_id_mom_idh <- readRDS("model_MedianVel_id_mom_slopes_bivariate_8_12_25.rds")
# model_id_mom_idh <- MCMCglmm(cbind(MedianVel_control,
# MedianVel_predator) ~ trait*ExpDay - 1 + YearBatch +
# Hour, random = ~idh(trait):FishId + idh(trait):MomId,
# rcov = ~idh(trait):units, family = c('gaussian',
# 'gaussian'), data = hourly_data, prior =
# prior.FishId_MomId_idh, nitt = 1000000, burnin = 100000,
# thin = 100, verbose = TRUE) saveRDS(model_id_mom_idh,
# file =
# 'model_MedianVel_id_mom_slopes_bivariate_8_12_25.rds')
summary(model_id_mom_idh)
```

```
##
##  Iterations = 100001:999901
##  Thinning interval  = 100
##  Sample size  = 9000
##
##  DIC: 85486.69
##
##  G-structure:  ~idh(trait):FishId
##
##                                post.mean l-95% CI u-95% CI eff.samp
## traitMedianVel_control.FishId    0.08228  0.04651   0.1224     6533
## traitMedianVel_predator.FishId   0.11707  0.06826   0.1756     5973
##
##                ~idh(trait):MomId
##
##                               post.mean l-95% CI u-95% CI eff.samp
## traitMedianVel_control.MomId     0.6413  0.07773    1.447     8732
## traitMedianVel_predator.MomId    0.7246  0.08041    1.681     8659
##
```

```
##  R-structure:  ~idh(trait):units
##
##                                post.mean l-95% CI u-95% CI eff.samp
## traitMedianVel_control.units     0.8303   0.8125   0.8498     9000
## traitMedianVel_predator.units    0.7794   0.7635   0.7961     9000
##
##  Location effects: cbind(MedianVel_control, MedianVel_predator) ~ trait * ExpDay - 1 + YearBatch + Hour
##
##                                  post.mean   l-95% CI   u-95% CI eff.samp
## traitMedianVel_control           1.5042548  0.7669327  2.2468553     9000
## traitMedianVel_predator          1.2862074  0.5610381  2.0680595     9000
## ExpDay                          -0.0012448 -0.0029879  0.0006027     9728
## YearBatch                       -0.5532199 -0.8195866 -0.2773300     9000
## Hour                            -0.0344335 -0.0371875 -0.0315745    10027
## traitMedianVel_predator:ExpDay   0.0045543  0.0020824  0.0069079     9284
##                                   pMCMC
## traitMedianVel_control           < 1e-04 ***
## traitMedianVel_predator          < 1e-04 ***
## ExpDay                          0.170444
## YearBatch                        < 1e-04 ***
## Hour                             < 1e-04 ***
## traitMedianVel_predator:ExpDay 0.000222 ***
## ---
## Signif. codes:  0 '***' 0.001 '**' 0.01 '*' 0.05 '.' 0.1 ' ' 1
```

```r
# FishId + MomId unstructured (covariance)
model_id_mom_us <- readRDS("model_MedianVel_id_mom_us_bivariate_hour_9_8_25.rds")
# model_id_mom_us <- MCMCglmm(cbind(MedianVel_control,
# MedianVel_predator) ~ trait*ExpDay - 1 + YearBatch +
# Hour, random = ~idh(trait):FishId + us(trait):MomId, rcov
# = ~idh(trait):units, family = c('gaussian', 'gaussian'),
# data = hourly_data, prior =
# prior.FishId_MomId_unstructured_mom, nitt = 1500000,
# burnin = 150000, thin = 400) saveRDS(model_id_mom_us,
# file =
# 'model_MedianVel_id_mom_us_bivariate_hour_9_8_25.rds')
summary(model_id_mom_us)
```

```
##
##  Iterations = 150001:1499601
##  Thinning interval  = 400
##  Sample size  = 3375
##
##  DIC: 85473.69
##
##  G-structure:  ~idh(trait):FishId
##
##                                 post.mean l-95% CI u-95% CI eff.samp
## traitMedianVel_control.FishId     0.08082  0.04920   0.1223     3375
## traitMedianVel_predator.FishId    0.11428  0.07031   0.1700     3190
##
##                ~us(trait):MomId
##
##                                                       post.mean l-95% CI
## traitMedianVel_control:traitMedianVel_control.MomId       1.253   0.3681
## traitMedianVel_predator:traitMedianVel_control.MomId      1.083   0.1976
## traitMedianVel_control:traitMedianVel_predator.MomId      1.083   0.1976
## traitMedianVel_predator:traitMedianVel_predator.MomId     1.410   0.3556
##                                                       u-95% CI eff.samp
## traitMedianVel_control:traitMedianVel_control.MomId      2.574     3375
## traitMedianVel_predator:traitMedianVel_control.MomId     2.356     3375
## traitMedianVel_control:traitMedianVel_predator.MomId     2.356     3375
```

```
## traitMedianVel_predator:traitMedianVel_predator.MomId    2.854     3567
##
##  R-structure:  ~idh(trait):units
##
##                                 post.mean l-95% CI u-95% CI eff.samp
## traitMedianVel_control.units     0.8296   0.8110   0.8483     3160
## traitMedianVel_predator.units    0.7800   0.7639   0.7974     3375
##
##  Location effects: cbind(MedianVel_control, MedianVel_predator) ~ trait * ExpDay - 1 + YearBatch + Hour
##
##                                   post.mean   l-95% CI   u-95% CI eff.samp  pMCMC
## traitMedianVel_control           2.0263163  1.1747667  2.7915776     3375 <3e-04
## traitMedianVel_predator          1.8058591  1.0123735  2.6740390     3375 <3e-04
## ExpDay                          -0.0011344 -0.0029541  0.0006395     3497   0.22
## YearBatch                       -0.7899249 -1.0142963 -0.5602700     3375 <3e-04
## Hour                            -0.0344214 -0.0370899 -0.0315681     3174 <3e-04
## traitMedianVel_predator:ExpDay  0.0045573  0.0020169  0.0069639     3612 <3e-04
##
## traitMedianVel_control         ***
## traitMedianVel_predator        ***
## ExpDay
## YearBatch                      ***
## Hour                           ***
## traitMedianVel_predator:ExpDay ***
## ---
## Signif. codes:  0 '***' 0.001 '**' 0.01 '*' 0.05 '.' 0.1 ' ' 1
```

```
# FishId + MomId with shared variance for FishId or MomId
# across treatments
model_id_mom_noidh <- readRDS("model_MedianVel_id_mom_bivariate_noidh_9_10_25.rds")
# model_id_mom_noidh <- MCMCglmm(cbind(MedianVel_control,
# MedianVel_predator) ~ trait*ExpDay - 1 + YearBatch +
# Hour, random = ~FishId + MomId, rcov = ~idh(trait):units,
# family = c('gaussian', 'gaussian'), data = hourly_data,
# prior = prior.FishId_MomId_NOidh, nitt = 1500000, burnin
# = 15000, thin = 400) saveRDS(model_id_mom_noidh, file =
# 'model_MedianVel_id_mom_bivariate_noidh_9_10_25.rds')
summary(model_id_mom_noidh)  # Best fit model!
```

```
##
##  Iterations = 15001:1499801
##  Thinning interval  = 400
##  Sample size  = 3713
##
##  DIC: 85473.41
##
##  G-structure:  ~FishId
##
##        post.mean l-95% CI u-95% CI eff.samp
## FishId    0.1095  0.07863   0.1433     3727
##
##                ~MomId
##
##       post.mean l-95% CI u-95% CI eff.samp
## MomId     1.276   0.2777    2.722     3713
##
##  R-structure:  ~idh(trait):units
##
##                                 post.mean l-95% CI u-95% CI eff.samp
## traitMedianVel_control.units     0.8294   0.8105   0.8475     3713
## traitMedianVel_predator.units    0.7794   0.7637   0.7964     3713
##
```

```
##  Location effects: cbind(MedianVel_control, MedianVel_predator) ~ trait * ExpDay - 1 + YearBatch + Hour
##
##                                post.mean   l-95% CI   u-95% CI eff.samp  pMCMC
## traitMedianVel_control          2.0452933  1.2658688  2.8348601     3713 <3e-04
## traitMedianVel_predator         1.8370353  1.1044635  2.6864748     3713 <3e-04
## ExpDay                         -0.0010912 -0.0029303  0.0006184     3713   0.25
## YearBatch                      -0.7990211 -1.0489107 -0.5753489     3713 <3e-04
## Hour                           -0.0344622 -0.0372016 -0.0316881     3713 <3e-04
## traitMedianVel_predator:ExpDay  0.0044777  0.0020685  0.0070326     3906 <3e-04
##
## traitMedianVel_control         ***
## traitMedianVel_predator        ***
## ExpDay
## YearBatch                      ***
## Hour                           ***
## traitMedianVel_predator:ExpDay ***
## ---
## Signif. codes:  0 '***' 0.001 '**' 0.01 '*' 0.05 '.' 0.1 ' ' 1
```

```r
autocorr.diag(model_id_mom_noidh$VCV)
```

```
##                  FishId        MomId traitMedianVel_control.units
## Lag 0      1.0000000000  1.000000000                  1.000000000
## Lag 400    0.0079088961  0.003048656                  0.003498184
## Lag 2000   0.0269468153 -0.014647230                  0.010540594
## Lag 4000  -0.0083564639 -0.004131898                 -0.021290614
## Lag 20000 -0.0006784796  0.001826491                  0.005460874
##           traitMedianVel_predator.units
## Lag 0                        1.00000000
## Lag 400                      0.01125644
## Lag 2000                    -0.01780600
## Lag 4000                     0.02978434
## Lag 20000                    0.01181099
```

```r
autocorr.diag(model_id_mom_noidh$Sol)
```

```
##           traitMedianVel_control traitMedianVel_predator       ExpDay
## Lag 0                 1.00000000             1.000000000  1.000000000
## Lag 400               0.01224312             0.008680018 -0.008515712
## Lag 2000             -0.02527170            -0.022240055  0.025940828
## Lag 4000             -0.01294598            -0.016054746  0.004785492
## Lag 20000            -0.01741125            -0.014670969 -0.025515481
##             YearBatch         Hour traitMedianVel_predator:ExpDay
## Lag 0      1.00000000  1.000000000                    1.000000000
## Lag 400    0.01410212 -0.007155110                   -0.025451310
## Lag 2000  -0.02999481 -0.005951867                    0.028094785
## Lag 4000  -0.01349952 -0.001840148                    0.007588493
## Lag 20000 -0.01704351 -0.000561398                   -0.016393268
```

```r
effectiveSize(model_id_mom_noidh$VCV)
```

```
##                       FishId                        MomId
##                     3726.644                     3713.000
##  traitMedianVel_control.units traitMedianVel_predator.units
##                     3713.000                     3713.000
```

```r
effectiveSize(model_id_mom_noidh$Sol)
```

```
##         traitMedianVel_control        traitMedianVel_predator
##                       3713.000                       3713.000
##                         ExpDay                      YearBatch
##                       3713.000                       3713.000
##                           Hour traitMedianVel_predator:ExpDay
##                       3713.000                       3905.885
```

Now that we have determined the random effects structure (FishId + MomId), we will test the contribution of the fixed effects Hour and YearBatch.

```
# Without YearBatch
model_id_mom_noYearBatch <- readRDS("model_MedianVel_id_mom_noYearBatch_bivariate_9_10_25.rds")
# model_id_mom_noYearBatch <-
# MCMCglmm(cbind(MedianVel_control, MedianVel_predator) ~
# trait*ExpDay - 1 + Hour, random = ~FishId + MomId, rcov =
# ~idh(trait):units, family = c('gaussian', 'gaussian'),
# data = hourly_data, prior = prior.FishId_MomId_NOidh,
# nitt = 500000, burnin = 50000, thin = 400, verbose =
# TRUE) saveRDS(model_id_mom_noYearBatch, file =
# 'model_MedianVel_id_mom_noYearBatch_bivariate_9_10_25.rds')
summary(model_id_mom_noYearBatch)  # Worse fit without
```

```
##
##  Iterations = 50001:499601
##  Thinning interval  = 400
##  Sample size  = 1125
##
##  DIC: 85548.91
##
##  G-structure:  ~FishId
##
##        post.mean l-95% CI u-95% CI eff.samp
## FishId    0.1087  0.07981   0.1455    947.5
##
##                ~MomId
##
##       post.mean l-95% CI u-95% CI eff.samp
## MomId   0.08943  0.02331   0.1846     1125
##
##  R-structure:  ~idh(trait):units
##
##                                post.mean l-95% CI u-95% CI eff.samp
## traitMedianVel_control.units     0.8326   0.8150   0.8502     1253
## traitMedianVel_predator.units    0.7799   0.7643   0.7974     1262
##
##  Location effects: cbind(MedianVel_control, MedianVel_predator) ~ trait * ExpDay - 1 + Hour
##
##                                 post.mean  l-95% CI  u-95% CI eff.samp  pMCMC
## traitMedianVel_control           0.269936  0.078531  0.452486     1125 0.0142
## traitMedianVel_predator          0.058457 -0.138011  0.231037     1125 0.5476
## ExpDay                          -0.001450 -0.003393  0.000152     1125 0.1173
## Hour                            -0.034337 -0.037144 -0.031570     1125 <9e-04
## traitMedianVel_predator:ExpDay  0.004526  0.002267  0.006914     1125 <9e-04
##
## traitMedianVel_control         *
## traitMedianVel_predator
## ExpDay
## Hour                           ***
## traitMedianVel_predator:ExpDay ***
## ---
## Signif. codes:  0 '***' 0.001 '**' 0.01 '*' 0.05 '.' 0.1 ' ' 1
```

```
# Without Hour
model_id_mom_noHour <- readRDS("model_MedianVel_id_mom_noHour_bivariate_9_10_25.rds")
# model_id_mom_noHour <- MCMCglmm(cbind(MedianVel_control,
# MedianVel_predator) ~ trait*ExpDay - 1 + YearBatch,
# random = ~FishId + MomId, rcov = ~idh(trait):units,
# family = c('gaussian', 'gaussian'), data = hourly_data,
# prior = prior.FishId_MomId_NOidh, nitt = 500000, burnin =
# 50000, thin = 400, verbose = TRUE)
```

```r
# saveRDS(model_id_mom_noHour, file =
# 'model_MedianVel_id_mom_noHour_bivariate_9_10_25.rds')
summary(model_id_mom_noHour)  # Worse fit without
```

```
##
##  Iterations = 50001:499601
##  Thinning interval  = 400
##  Sample size  = 1125
##
##  DIC: 86040.81
##
##  G-structure:  ~FishId
##
##        post.mean l-95% CI u-95% CI eff.samp
## FishId    0.1094  0.07866   0.1414     1125
##
##                ~MomId
##
##       post.mean l-95% CI u-95% CI eff.samp
## MomId     1.228   0.3308    2.608     1125
##
##  R-structure:  ~idh(trait):units
##
##                                 post.mean l-95% CI u-95% CI eff.samp
## traitMedianVel_control.units     0.8516   0.8317   0.8694     1125
## traitMedianVel_predator.units    0.7866   0.7702   0.8020     1171
##
##  Location effects: cbind(MedianVel_control, MedianVel_predator) ~ trait * ExpDay - 1 + YearBatch
##
##                                   post.mean   l-95% CI   u-95% CI eff.samp
## traitMedianVel_control            1.8456143  1.1250126  2.7098454     1125
## traitMedianVel_predator           1.6348431  0.8779732  2.4728878     1125
## ExpDay                           -0.0010909 -0.0030037  0.0005641     1125
## YearBatch                        -0.7960465 -1.0469524 -0.5718647     1125
## traitMedianVel_predator:ExpDay  0.0044334  0.0019864  0.0068359     1125
##                                   pMCMC
## traitMedianVel_control           0.00178 **
## traitMedianVel_predator          0.00178 **
## ExpDay                           0.23467
## YearBatch                        < 9e-04 ***
## traitMedianVel_predator:ExpDay 0.00178 **
## ---
## Signif. codes:  0 '***' 0.001 '**' 0.01 '*' 0.05 '.' 0.1 ' ' 1
```

Including YearBatch and Hour do improve the fit of the model, so we will include them in our final model.

Because the best supported model includes a single variable for both FishId and MomId (not separate by treatment), this suggests that variance does not differ by treatment–we would use pooled FishId and MomId variables across treatments. Therefore, we can and should run a univariate instead of bivariate model to partition overall sources of variance.

Univariate model for estimating overall variance components

```r
# Set priors:

# Null
prior.univariate.null <- list(R = list(V = 1, nu = 0.002))

# FishId
prior.univariate.FishId <- list(R = list(V = 1, nu = 0.002),
```

```r
    G = list(G1 = list(V = 1, nu = 0.002, alpha.mu = 0, alpha.V = 25^2)))

# MomId
prior.univariate.MomId <- list(R = list(V = 1, nu = 0.002), G = list(G1 = list(V = 1,
    nu = 0.002, alpha.mu = 0, alpha.V = 25^2)))

# FishId + MomId
prior.univariate.FishId.MomId <- list(R = list(V = 1, nu = 0.002),
    G = list(G1 = list(V = 1, nu = 0.002, alpha.mu = 0, alpha.V = 25^2),
        G2 = list(V = 1, nu = 0.002, alpha.mu = 0, alpha.V = 25^2)))

# FishId slopes + MomId
prior.univariate.FishId_slopes.MomId <- list(R = list(V = 1,
    nu = 0.002), G = list(G1 = list(V = diag(2), nu = 0.002,
    alpha.mu = rep(0, 2), alpha.V = diag(25^2, 2)), G2 = list(V = 1,
    nu = 0.002, alpha.mu = 0, alpha.V = 25^2)))

# FishId slopes + MomId slopes
prior.univariate.FishId_slopes.MomId_slopes <- list(R = list(V = 1,
    nu = 0.002), G = list(G1 = list(V = diag(2), nu = 0.002,
    alpha.mu = rep(0, 2), alpha.V = diag(25^2, 2)), G2 = list(V = diag(2),
    nu = 0.002, alpha.mu = rep(0, 2), alpha.V = diag(25^2, 2))))

# FishId slopes + MomId slopes with heterogeneous residual
# variance
hourly_data$ExpDay_factor <- as.factor(hourly_data$ExpDay)
num_days <- nlevels(hourly_data$ExpDay_factor)
R_prior <- list(V = diag(num_days) * 0.5, nu = num_days + 0.002)
prior.univariate.FishId_slopes.MomId_slopes.heteroResidual <- list(R = R_prior,
    G = list(G1 = list(V = diag(2), nu = 0.002, alpha.mu = rep(0,
        2), alpha.V = diag(25^2, 2)), G2 = list(V = diag(2),
        nu = 0.002, alpha.mu = rep(0, 2), alpha.V = diag(25^2,
            2))))

# FishId slopes + MomId slopes with heterogeneous residual
# variance MORE INFORMED PRIORS
prior.univariate.FishId_slopes.MomId_slopes.heteroResidual_informed <- list(R = R_prior,
    G = list(G1 = list(V = diag(2) * 0.5, nu = 2.002, alpha.mu = c(0,
        0), alpha.V = diag(2) * 25^2), G2 = list(V = diag(2) *
        0.5, nu = 2.002, alpha.mu = c(0, 0), alpha.V = diag(2) *
        25^2)))


### Model comparison

# Null
model_MedianVel_big_univariate_null <- readRDS("model_MedianVel_big_univariate_null_9_18_25.rds")
# model_MedianVel_big_univariate_null <-
# MCMCglmm(MedianVel_hourly_scaled ~ PredatorCue*ExpDay +
# YearBatch + Hour, rcov = ~units, family = 'gaussian',
# data = hourly_data, prior = prior.univariate.null, nitt =
# 500000, burnin = 50000, thin = 100)
# saveRDS(model_MedianVel_big_univariate_null, file =
# 'model_MedianVel_big_univariate_null_9_18_25.rds')
summary(model_MedianVel_big_univariate_null)
```

```
##
##  Iterations = 50001:499901
##  Thinning interval  = 100
##  Sample size  = 4500
##
```

```
##  DIC: 91812.99
##
##  R-structure:  ~units
##
##       post.mean l-95% CI u-95% CI eff.samp
## units    0.9783   0.9644   0.9936     4500
##
##  Location effects: MedianVel_hourly_scaled ~ PredatorCue * ExpDay + YearBatch + Hour
##
##                     post.mean   l-95% CI   u-95% CI eff.samp  pMCMC
## (Intercept)         0.3233259  0.2802893  0.3649543     4500 <2e-04 ***
## PredatorCue        -0.2235279 -0.2663359 -0.1841743     4500 <2e-04 ***
## ExpDay             -0.0015011 -0.0034380  0.0004059     4500 0.1231
## YearBatch          -0.0101246 -0.0204356 -0.0012992     4500 0.0382 *
## Hour               -0.0346480 -0.0377440 -0.0316450     4871 <2e-04 ***
## PredatorCue:ExpDay  0.0040987  0.0015098  0.0068288     4500 0.0040 **
## ---
## Signif. codes:  0 '***' 0.001 '**' 0.01 '*' 0.05 '.' 0.1 ' ' 1
```

```
# FishId
model_MedianVel_big_univariate_id <- readRDS("model_MedianVel_big_univariate_id_9_21_25.rds")
# model_MedianVel_big_univariate_id <-
# MCMCglmm(MedianVel_hourly_scaled ~ PredatorCue*ExpDay +
# YearBatch + Hour, random = ~FishId, rcov = ~units, family
# = 'gaussian', data = hourly_data, prior =
# prior.univariate.FishId, nitt = 500000, burnin = 50000,
# thin = 100) saveRDS(model_MedianVel_big_univariate_id,
# file = 'model_MedianVel_big_univariate_id_9_21_25.rds')
summary(model_MedianVel_big_univariate_id)
```

```
##
##  Iterations = 50001:499901
##  Thinning interval  = 100
##  Sample size  = 4500
##
##  DIC: 85543.86
##
##  G-structure:  ~FishId
##
##        post.mean l-95% CI u-95% CI eff.samp
## FishId    0.1988   0.1395   0.2582     4500
##
##  R-structure:  ~units
##
##       post.mean l-95% CI u-95% CI eff.samp
## units    0.8046   0.7924   0.8175     4500
##
##  Location effects: MedianVel_hourly_scaled ~ PredatorCue * ExpDay + YearBatch + Hour
##
##                     post.mean   l-95% CI   u-95% CI eff.samp  pMCMC
## (Intercept)         0.6173019  0.3922994  0.8485578     4500 <2e-04 ***
## PredatorCue        -0.1955829 -0.3647249 -0.0145597     4500 0.0271 *
## ExpDay             -0.0013385 -0.0031254  0.0004297     4285 0.1364
## YearBatch          -0.1359287 -0.2141548 -0.0592784     4500 <2e-04 ***
## Hour               -0.0346813 -0.0375957 -0.0318805     4500 <2e-04 ***
## PredatorCue:ExpDay  0.0045153  0.0021398  0.0070855     4500 <2e-04 ***
## ---
## Signif. codes:  0 '***' 0.001 '**' 0.01 '*' 0.05 '.' 0.1 ' ' 1
```

```
# MomId
model_MedianVel_big_univariate_mom <- readRDS("model_MedianVel_big_univariate_mom_9_21_25.rds")
# model_MedianVel_big_univariate_mom <-
# MCMCglmm(MedianVel_hourly_scaled ~ PredatorCue*ExpDay +
```

```r
# YearBatch + Hour, random = ~MomId, rcov = ~units, family
# = 'gaussian', data = hourly_data, prior =
# prior.univariate.MomId, nitt = 500000, burnin = 50000,
# thin = 100) saveRDS(model_MedianVel_big_univariate_mom,
# file = 'model_MedianVel_big_univariate_mom_9_21_25.rds')
summary(model_MedianVel_big_univariate_mom)
```

```
##
##  Iterations = 50001:499901
##  Thinning interval  = 100
##  Sample size  = 4500
##
##  DIC: 88723.83
##
##  G-structure:  ~MomId
##
##       post.mean l-95% CI u-95% CI eff.samp
## MomId     1.364   0.3204    2.823     2410
##
##  R-structure:  ~units
##
##       post.mean l-95% CI u-95% CI eff.samp
## units    0.8897   0.8765   0.9043     4500
##
##  Location effects: MedianVel_hourly_scaled ~ PredatorCue * ExpDay + YearBatch + Hour
##
##                     post.mean   l-95% CI   u-95% CI eff.samp    pMCMC
## (Intercept)         2.0166093  1.2138413  2.8739824     4234  < 2e-04 ***
## PredatorCue        -0.2337918 -0.2725883 -0.1936886     4500  < 2e-04 ***
## ExpDay             -0.0019533 -0.0038311 -0.0001559     4500 0.039556 *
## YearBatch          -0.7884719 -1.0530590 -0.5612309     4500  < 2e-04 ***
## Hour               -0.0347127 -0.0376664 -0.0318053     4315  < 2e-04 ***
## PredatorCue:ExpDay  0.0044636  0.0020524  0.0071656     4500 0.000444 ***
## ---
## Signif. codes:  0 '***' 0.001 '**' 0.01 '*' 0.05 '.' 0.1 ' ' 1
```

```r
# FishId, MomId
model_MedianVel_big_univariate_id_mom <- readRDS("model_MedianVel_big_univariate_id_mom_9_18_25.rds")
# model_MedianVel_big_univariate_id_mom <-
# MCMCglmm(MedianVel_hourly_scaled ~ PredatorCue*ExpDay +
# YearBatch + Hour, random = ~FishId + MomId, rcov =
# ~units, family = 'gaussian', data = hourly_data, prior =
# prior.univariate.FishId.MomId, nitt = 500000, burnin =
# 50000, thin = 100)
# saveRDS(model_MedianVel_big_univariate_id_mom, file =
# 'model_MedianVel_big_univariate_id_mom_9_18_25.rds')
summary(model_MedianVel_big_univariate_id_mom)
```

```
##
##  Iterations = 50001:499901
##  Thinning interval  = 100
##  Sample size  = 4500
##
##  DIC: 85486.33
##
##  G-structure:  ~FishId
##
##        post.mean l-95% CI u-95% CI eff.samp
## FishId    0.1107  0.07976   0.1457     4500
##
##               ~MomId
##
```

```
##       post.mean l-95% CI u-95% CI eff.samp
## MomId     1.399   0.3626    3.022     1648
##
##  R-structure:  ~units
##
##       post.mean l-95% CI u-95% CI eff.samp
## units    0.8033   0.7909   0.8159     4500
##
##  Location effects: MedianVel_hourly_scaled ~ PredatorCue * ExpDay + YearBatch + Hour
##
##                       post.mean   l-95% CI   u-95% CI eff.samp    pMCMC
## (Intercept)           2.1098608  1.3132440  2.9937013     4500  < 2e-04 ***
## PredatorCue          -0.2124243 -0.3387512 -0.0732052     4500 0.000889 ***
## ExpDay               -0.0010649 -0.0028167  0.0007262     4500 0.239556
## YearBatch            -0.8249251 -1.0531919 -0.5942496     4500  < 2e-04 ***
## Hour                 -0.0347244 -0.0376446 -0.0320467     4500  < 2e-04 ***
## PredatorCue:ExpDay    0.0044838  0.0020452  0.0068027     4500 0.000444 ***
## ---
## Signif. codes:  0 '***' 0.001 '**' 0.01 '*' 0.05 '.' 0.1 ' ' 1
```

```
# FishId with slopes, MomId
model_MedianVel_big_univariate_id_slopes_mom <- readRDS("model_MedianVel_big_univariate_id_slopes_mom_9_18_2
# model_MedianVel_big_univariate_id_slopes_mom <-
# MCMCglmm(MedianVel_hourly_scaled ~ PredatorCue*ExpDay +
# YearBatch + Hour, random = ~us(1 + ExpDay):FishId +
# MomId, rcov = ~units, family = 'gaussian', data =
# hourly_data, prior =
# prior.univariate.FishId_slopes.MomId, nitt = 500000,
# burnin = 50000, thin = 100)
# saveRDS(model_MedianVel_big_univariate_id_slopes_mom,
# file =
# 'model_MedianVel_big_univariate_id_slopes_mom_9_18_25.rds')
summary(model_MedianVel_big_univariate_id_slopes_mom)
```

```
##
##  Iterations = 50001:499901
##  Thinning interval  = 100
##  Sample size  = 4500
##
##  DIC: 82857.09
##
##  G-structure:  ~us(1 + ExpDay):FishId
##
##                                post.mean   l-95% CI  u-95% CI eff.samp
## (Intercept):(Intercept).FishId   0.20018  0.1389218  0.267627     4500
## ExpDay:(Intercept).FishId       -0.01124 -0.0156193 -0.007061     4750
## (Intercept):ExpDay.FishId       -0.01124 -0.0156193 -0.007061     4750
## ExpDay:ExpDay.FishId             0.00119  0.0008399  0.001582     5395
##
##                ~MomId
##
##       post.mean l-95% CI u-95% CI eff.samp
## MomId     2.907   0.8275    5.755     4500
##
##  R-structure:  ~units
##
##       post.mean l-95% CI u-95% CI eff.samp
## units     0.739   0.7277   0.7498     4500
##
##  Location effects: MedianVel_hourly_scaled ~ PredatorCue * ExpDay + YearBatch + Hour
##
##                     post.mean  l-95% CI  u-95% CI eff.samp  pMCMC
```

```
## (Intercept)         3.016915  1.928129  4.074508     4944 <2e-04 ***
## PredatorCue        -0.216171 -0.404992 -0.053634     4500 0.0138 *
## ExpDay             -0.001775 -0.011376  0.008061     4500 0.7244
## YearBatch          -1.232622 -1.452413 -1.017601     4786 <2e-04 ***
## Hour               -0.034751 -0.037389 -0.031959     4500 <2e-04 ***
## PredatorCue:ExpDay  0.001877 -0.010770  0.015945     4500 0.7862
## ---
## Signif. codes:  0 '***' 0.001 '**' 0.01 '*' 0.05 '.' 0.1 ' ' 1
```

```
# FishId with slopes, MomId with slopes
model_MedianVel_big_univariate_id_slopes_mom_slopes <- readRDS("model_MedianVel_big_univariate_id_slopes_mom
# model_MedianVel_big_univariate_id_slopes_mom_slopes <-
# MCMCglmm(MedianVel_hourly_scaled ~ PredatorCue*ExpDay +
# YearBatch + Hour, random = ~us(1 + ExpDay):FishId + us(1
# + ExpDay):MomId, rcov = ~units, family = 'gaussian', data
# = hourly_data, prior =
# prior.univariate.FishId_slopes.MomId_slopes, nitt =
# 500000, burnin = 50000, thin = 100)
# saveRDS(model_MedianVel_big_univariate_id_slopes_mom_slopes,
# file =
# 'model_MedianVel_big_univariate_id_slopes_mom_slopes_9_19_25.rds')
summary(model_MedianVel_big_univariate_id_slopes_mom_slopes)  # Best model
```

```
##
##  Iterations = 50001:499901
##  Thinning interval  = 100
##  Sample size  = 4500
##
##  DIC: 82853.05
##
##  G-structure:  ~us(1 + ExpDay):FishId
##
##                                    post.mean   l-95% CI  u-95% CI eff.samp
## (Intercept):(Intercept).FishId  0.1714056  0.1194732  0.223823     4500
## ExpDay:(Intercept).FishId      -0.0081161 -0.0115171 -0.005025     4500
## (Intercept):ExpDay.FishId      -0.0081161 -0.0115171 -0.005025     4500
## ExpDay:ExpDay.FishId            0.0008536  0.0005893  0.001153     4500
##
##                  ~us(1 + ExpDay):MomId
##
##                                post.mean   l-95% CI u-95% CI eff.samp
## (Intercept):(Intercept).MomId 2.7761889  7.905e-01 5.912436     4221
## ExpDay:(Intercept).MomId      0.0171187 -1.459e-02 0.054837     4415
## (Intercept):ExpDay.MomId      0.0171187 -1.459e-02 0.054837     4415
## ExpDay:ExpDay.MomId           0.0006946  5.665e-05 0.001609     2169
##
##  R-structure:  ~units
##
##       post.mean l-95% CI u-95% CI eff.samp
## units    0.7387   0.7271     0.75     5197
##
##  Location effects: MedianVel_hourly_scaled ~ PredatorCue * ExpDay + YearBatch + Hour
##
##                    post.mean  l-95% CI  u-95% CI eff.samp  pMCMC
## (Intercept)         3.032401  2.067419  4.155918     4500 <2e-04 ***
## PredatorCue        -0.209020 -0.367134 -0.041649     4500 0.0133 *
## ExpDay             -0.003388 -0.020083  0.013192     4243 0.6747
## YearBatch          -1.237547 -1.458156 -1.021622     4500 <2e-04 ***
## Hour               -0.034745 -0.037573 -0.032183     4263 <2e-04 ***
## PredatorCue:ExpDay  0.001723 -0.009661  0.013395     4500 0.7596
## ---
## Signif. codes:  0 '***' 0.001 '**' 0.01 '*' 0.05 '.' 0.1 ' ' 1
```

```r
# Now use best model (FishId with slopes, MomId with
# slopes) with heterogeneous residual variance
model_MedianVel_big_univariate_id_slopes_mom_slopes_hetero_residual <- readRDS("model_MedianVel_big_univaria
# model_MedianVel_big_univariate_id_slopes_mom_slopes_hetero_residual
# <- MCMCglmm(MedianVel_hourly_scaled ~ PredatorCue*ExpDay
# + YearBatch + Hour, random = ~us(1 + ExpDay):FishId +
# us(1 + ExpDay):MomId, rcov = ~idh(ExpDay_factor):units,
# family = 'gaussian', data = hourly_data, prior =
# prior.univariate.FishId_slopes.MomId_slopes.heteroResidual,
# nitt = 500000, burnin = 50000, thin = 100)
# saveRDS(model_MedianVel_big_univariate_id_slopes_mom_slopes_hetero_residual,
# file =
# 'model_MedianVel_big_univariate_id_slopes_mom_slopes_hetero_residual_9_22_25.rds')
summary(model_MedianVel_big_univariate_id_slopes_mom_slopes_hetero_residual)  # Better fit with heterogeneou
```

```
##
##  Iterations = 50001:499901
##  Thinning interval  = 100
##  Sample size  = 4500
##
##  DIC: 81650.91
##
##  G-structure:  ~us(1 + ExpDay):FishId
##
##                                  post.mean   l-95% CI  u-95% CI eff.samp
## (Intercept):(Intercept).FishId  0.1657317  0.1167728  0.220064     4500
## ExpDay:(Intercept).FishId      -0.0076094 -0.0106039 -0.004351     2444
## (Intercept):ExpDay.FishId      -0.0076094 -0.0106039 -0.004351     2444
## ExpDay:ExpDay.FishId            0.0007951  0.0005624  0.001076     1633
##
##                ~us(1 + ExpDay):MomId
##
##                                post.mean   l-95% CI u-95% CI eff.samp
## (Intercept):(Intercept).MomId 1.6979561  6.337e-02 4.040944     4500
## ExpDay:(Intercept).MomId      0.0095698 -1.520e-02 0.034627     4500
## (Intercept):ExpDay.MomId      0.0095698 -1.520e-02 0.034627     4500
## ExpDay:ExpDay.MomId           0.0005362  7.511e-06 0.001292     1035
##
##  R-structure:  ~idh(ExpDay_factor):units
##
##                        post.mean l-95% CI u-95% CI eff.samp
## ExpDay_factor0.units      2.1265   1.9544   2.3019     4500
## ExpDay_factor1.units      1.0691   0.9833   1.1576     4767
## ExpDay_factor2.units      0.6748   0.6205   0.7331     4500
## ExpDay_factor3.units      0.6755   0.6197   0.7340     5096
## ExpDay_factor4.units      0.6914   0.6364   0.7514     4500
## ExpDay_factor5.units      0.6220   0.5713   0.6736     4500
## ExpDay_factor6.units      0.6993   0.6419   0.7575     4500
## ExpDay_factor7.units      0.6281   0.5795   0.6813     4500
## ExpDay_factor8.units      0.7375   0.6797   0.7967     4700
## ExpDay_factor9.units      0.7284   0.6697   0.7871     4500
## ExpDay_factor10.units     0.6903   0.6320   0.7482     4809
## ExpDay_factor11.units     0.5168   0.4750   0.5602     4710
## ExpDay_factor12.units     0.6501   0.5999   0.7039     4500
## ExpDay_factor13.units     0.7330   0.6744   0.7898     4240
## ExpDay_factor14.units     0.7524   0.6913   0.8132     4500
## ExpDay_factor15.units     0.8796   0.8081   0.9470     4299
## ExpDay_factor16.units     0.5789   0.5308   0.6253     4500
## ExpDay_factor17.units     0.6403   0.5878   0.6924     4500
## ExpDay_factor18.units     0.5292   0.4862   0.5731     4439
## ExpDay_factor19.units     0.7942   0.7301   0.8572     4500
```

```
## ExpDay_factor20.units    0.6357   0.5847   0.6871     4973
## ExpDay_factor21.units    0.6273   0.5781   0.6793     4500
## ExpDay_factor22.units    0.6685   0.6152   0.7208     4711
## ExpDay_factor23.units    0.7035   0.6490   0.7611     4500
## ExpDay_factor24.units    0.5554   0.5125   0.6051     6016
## ExpDay_factor25.units    0.7375   0.6763   0.7976     4500
## ExpDay_factor26.units    0.6740   0.6176   0.7315     4500
## ExpDay_factor27.units    0.5991   0.5526   0.6553     4500
##
##  Location effects: MedianVel_hourly_scaled ~ PredatorCue * ExpDay + YearBatch + Hour
##
##                      post.mean   l-95% CI   u-95% CI eff.samp  pMCMC
## (Intercept)          2.2811848  1.0791251  3.5627985     4500 <2e-04 ***
## PredatorCue         -0.1848312 -0.3402910 -0.0202992     3990 0.0284 *
## ExpDay              -0.0005537 -0.0165530  0.0146559     4500 0.9307
## YearBatch           -0.9294984 -1.3893853 -0.4847935     3317 <2e-04 ***
## Hour                -0.0325741 -0.0351616 -0.0299407     4563 <2e-04 ***
## PredatorCue:ExpDay  0.0009519 -0.0104429  0.0122351     4560 0.8640
## ---
## Signif. codes:  0 '***' 0.001 '**' 0.01 '*' 0.05 '.' 0.1 ' ' 1
```

```
# Comparison of best fit model with one using more informed
# priors to see if that makes a difference
model_MedianVel_big_univariate_id_slopes_mom_slopes_hetero_residual_informed_priors <- readRDS("model_Median
# model_MedianVel_big_univariate_id_slopes_mom_slopes_hetero_residual_informed_priors
# <- MCMCglmm(MedianVel_hourly_scaled ~ PredatorCue*ExpDay
# + YearBatch + Hour, random = ~us(1 + ExpDay):FishId +
# us(1 + ExpDay):MomId, rcov = ~idh(ExpDay_factor):units,
# family = 'gaussian', data = hourly_data, prior =
# prior.univariate.FishId_slopes.MomId_slopes.heteroResidual_informed,
# nitt = 500000, burnin = 50000, thin = 100)
# saveRDS(model_MedianVel_big_univariate_id_slopes_mom_slopes_hetero_residual_informed_priors,
# file =
# 'model_MedianVel_big_univariate_id_slopes_mom_slopes_hetero_residual_informed_priors_9_22_25.rds')
summary(model_MedianVel_big_univariate_id_slopes_mom_slopes_hetero_residual_informed_priors)  # Worse fit (
```

```
##
##  Iterations = 50001:499901
##  Thinning interval  = 100
##  Sample size  = 4500
##
##  DIC: 81650.94
##
##  G-structure:  ~us(1 + ExpDay):FishId
##
##                                post.mean   l-95% CI  u-95% CI eff.samp
## (Intercept):(Intercept).FishId  0.1623415  0.1137840  0.215149     4450
## ExpDay:(Intercept).FishId      -0.0072466 -0.0103931 -0.004405     4510
## (Intercept):ExpDay.FishId      -0.0072466 -0.0103931 -0.004405     4510
## ExpDay:ExpDay.FishId            0.0007731  0.0005352  0.001023     3705
##
##                ~us(1 + ExpDay):MomId
##
##                               post.mean   l-95% CI u-95% CI eff.samp
## (Intercept):(Intercept).MomId 1.5477683  9.934e-02 3.529287    571.9
## ExpDay:(Intercept).MomId      0.0073029 -1.150e-02 0.030409   4500.0
## (Intercept):ExpDay.MomId      0.0073029 -1.150e-02 0.030409   4500.0
## ExpDay:ExpDay.MomId           0.0005346  4.476e-05 0.001235    862.9
##
##  R-structure:  ~idh(ExpDay_factor):units
##
##                        post.mean l-95% CI u-95% CI eff.samp
```

```
## ExpDay_factor0.units     2.1288   1.9626   2.3094     4038
## ExpDay_factor1.units     1.0684   0.9826   1.1507     4500
## ExpDay_factor2.units     0.6747   0.6204   0.7341     4500
## ExpDay_factor3.units     0.6754   0.6204   0.7326     4500
## ExpDay_factor4.units     0.6915   0.6360   0.7491     4500
## ExpDay_factor5.units     0.6220   0.5690   0.6719     4722
## ExpDay_factor6.units     0.6998   0.6441   0.7606     4500
## ExpDay_factor7.units     0.6282   0.5756   0.6795     3768
## ExpDay_factor8.units     0.7377   0.6796   0.7980     4500
## ExpDay_factor9.units     0.7288   0.6745   0.7890     4500
## ExpDay_factor10.units    0.6908   0.6321   0.7489     4500
## ExpDay_factor11.units    0.5164   0.4757   0.5632     4500
## ExpDay_factor12.units    0.6499   0.5992   0.7065     4500
## ExpDay_factor13.units    0.7340   0.6738   0.7929     4500
## ExpDay_factor14.units    0.7521   0.6916   0.8133     4500
## ExpDay_factor15.units    0.8790   0.8105   0.9471     4500
## ExpDay_factor16.units    0.5792   0.5339   0.6244     4500
## ExpDay_factor17.units    0.6415   0.5915   0.6954     4158
## ExpDay_factor18.units    0.5296   0.4875   0.5723     5451
## ExpDay_factor19.units    0.7940   0.7310   0.8595     4500
## ExpDay_factor20.units    0.6357   0.5866   0.6885     4206
## ExpDay_factor21.units    0.6278   0.5777   0.6797     4500
## ExpDay_factor22.units    0.6681   0.6146   0.7237     4500
## ExpDay_factor23.units    0.7018   0.6488   0.7602     4500
## ExpDay_factor24.units    0.5555   0.5106   0.6027     4500
## ExpDay_factor25.units    0.7384   0.6780   0.7998     3939
## ExpDay_factor26.units    0.6745   0.6167   0.7309     4735
## ExpDay_factor27.units    0.5989   0.5505   0.6527     4500
##
##  Location effects: MedianVel_hourly_scaled ~ PredatorCue * ExpDay + YearBatch + Hour
##
##                      post.mean   l-95% CI   u-95% CI eff.samp  pMCMC
## (Intercept)          2.2529653  1.1706398  3.5321828     1010 <2e-04 ***
## PredatorCue         -0.1863857 -0.3340494 -0.0279348     4500 0.0164 *
## ExpDay              -0.0005665 -0.0156434  0.0151012     4917 0.9311
## YearBatch           -0.9165757 -1.3503686 -0.4817851      492 <2e-04 ***
## Hour                -0.0325831 -0.0353016 -0.0300159     4500 <2e-04 ***
## PredatorCue:ExpDay   0.0010121 -0.0093283  0.0123812     4500 0.8396
## ---
## Signif. codes:  0 '***' 0.001 '**' 0.01 '*' 0.05 '.' 0.1 ' ' 1
```

Now we test for inclusion of additional fixed effects, YearBatch and Hour

```r
# Without YearBatch
model_MedianVel_big_univariate_id_slopes_mom_slopes_noYearBatch <- readRDS("model_MedianVel_big_univariate_i
# model_MedianVel_big_univariate_id_slopes_mom_slopes_noYearBatch
# <- MCMCglmm(MedianVel_hourly_scaled ~ PredatorCue*ExpDay
# + Hour, random = ~us(1 + ExpDay):FishId + us(1 +
# ExpDay):MomId, rcov = ~idh(ExpDay_factor):units, family =
# 'gaussian', data = hourly_data, prior =
# prior.univariate.FishId_slopes.MomId_slopes.heteroResidual,
# nitt = 500000, burnin = 50000, thin = 100)
# saveRDS(model_MedianVel_big_univariate_id_slopes_mom_slopes_noYearBatch,
# file =
# 'model_MedianVel_big_univariate_id_slopes_mom_slopes_noYearBatch_9_22_25.rds')
summary(model_MedianVel_big_univariate_id_slopes_mom_slopes_noYearBatch)
```

```
##
##  Iterations = 50001:499901
##  Thinning interval  = 100
##  Sample size  = 4500
##
##  DIC: 81697.54
```

```
##
##  G-structure:  ~us(1 + ExpDay):FishId
##
##                                  post.mean   l-95% CI  u-95% CI eff.samp
## (Intercept):(Intercept).FishId  0.1659925  0.1151098  0.217241     4500
## ExpDay:(Intercept).FishId      -0.0075101 -0.0110056 -0.004787     4500
## (Intercept):ExpDay.FishId      -0.0075101 -0.0110056 -0.004787     4500
## ExpDay:ExpDay.FishId            0.0007835  0.0005529  0.001053     3842
##
##                ~us(1 + ExpDay):MomId
##
##                                post.mean   l-95% CI u-95% CI eff.samp
## (Intercept):(Intercept).MomId  0.1287142  1.202e-02 0.284860     2995
## ExpDay:(Intercept).MomId      -0.0036520 -1.210e-02 0.003022     4500
## (Intercept):ExpDay.MomId      -0.0036520 -1.210e-02 0.003022     4500
## ExpDay:ExpDay.MomId            0.0005281  1.407e-05 0.001245     1873
##
##  R-structure:  ~idh(ExpDay_factor):units
##
##                          post.mean l-95% CI u-95% CI eff.samp
## ExpDay_factor0.units     2.2110   2.0308   2.3824     4669
## ExpDay_factor1.units     1.0740   0.9814   1.1574     4500
## ExpDay_factor2.units     0.6777   0.6213   0.7352     4725
## ExpDay_factor3.units     0.6778   0.6196   0.7356     4500
## ExpDay_factor4.units     0.6927   0.6358   0.7495     4500
## ExpDay_factor5.units     0.6230   0.5738   0.6751     4500
## ExpDay_factor6.units     0.6985   0.6407   0.7590     4500
## ExpDay_factor7.units     0.6283   0.5775   0.6811     4500
## ExpDay_factor8.units     0.7372   0.6803   0.8010     4500
## ExpDay_factor9.units     0.7272   0.6680   0.7877     4727
## ExpDay_factor10.units    0.6889   0.6290   0.7451     4500
## ExpDay_factor11.units    0.5146   0.4732   0.5571     4290
## ExpDay_factor12.units    0.6498   0.5946   0.7026     4500
## ExpDay_factor13.units    0.7334   0.6759   0.7942     4500
## ExpDay_factor14.units    0.7525   0.6940   0.8160     4500
## ExpDay_factor15.units    0.8793   0.8122   0.9528     4500
## ExpDay_factor16.units    0.5786   0.5317   0.6236     5153
## ExpDay_factor17.units    0.6415   0.5936   0.6969     4500
## ExpDay_factor18.units    0.5289   0.4840   0.5709     4306
## ExpDay_factor19.units    0.7948   0.7307   0.8582     4726
## ExpDay_factor20.units    0.6362   0.5858   0.6872     4500
## ExpDay_factor21.units    0.6272   0.5751   0.6768     4653
## ExpDay_factor22.units    0.6678   0.6135   0.7218     4500
## ExpDay_factor23.units    0.7015   0.6454   0.7597     4500
## ExpDay_factor24.units    0.5551   0.5088   0.6021     4269
## ExpDay_factor25.units    0.7379   0.6797   0.7983     4500
## ExpDay_factor26.units    0.6733   0.6155   0.7308     4500
## ExpDay_factor27.units    0.5963   0.5471   0.6525     4500
##
##  Location effects: MedianVel_hourly_scaled ~ PredatorCue * ExpDay + Hour
##
##                       post.mean   l-95% CI   u-95% CI eff.samp  pMCMC
## (Intercept)           0.2157678 -0.0237178  0.4395906     4500 0.0707 .
## PredatorCue          -0.1843683 -0.3405409 -0.0167541     4500 0.0249 *
## ExpDay               -0.0005483 -0.0158367  0.0142719     4787 0.9307
## Hour                 -0.0325464 -0.0350353 -0.0299626     4385 <2e-04 ***
## PredatorCue:ExpDay    0.0010073 -0.0095435  0.0128440     4500 0.8676
## ---
## Signif. codes:  0 '***' 0.001 '**' 0.01 '*' 0.05 '.' 0.1 ' ' 1
```

```
# Without Hour
model_MedianVel_big_univariate_id_slopes_mom_slopes_noHour <- readRDS("model_MedianVel_big_univariate_id_slo
```

```r
# model_MedianVel_big_univariate_id_slopes_mom_slopes_noHour
# <- MCMCglmm(MedianVel_hourly_scaled ~ PredatorCue*ExpDay
# + YearBatch, random = ~us(1 + ExpDay):FishId + us(1 +
# ExpDay):MomId, rcov = ~idh(ExpDay_factor):units, family =
# 'gaussian', data = hourly_data, prior =
# prior.univariate.FishId_slopes.MomId_slopes.heteroResidual,
# nitt = 500000, burnin = 50000, thin = 100)
# saveRDS(model_MedianVel_big_univariate_id_slopes_mom_slopes_noHour,
# file =
# 'model_MedianVel_big_univariate_id_slopes_mom_slopes_noHour_9_22_25.rds')
summary(model_MedianVel_big_univariate_id_slopes_mom_slopes_noHour)
```

```
##
##  Iterations = 50001:499901
##  Thinning interval  = 100
##  Sample size  = 4500
##
##  DIC: 82240.14
##
##  G-structure:  ~us(1 + ExpDay):FishId
##
##                                  post.mean   l-95% CI  u-95% CI eff.samp
## (Intercept):(Intercept).FishId  0.1658106  0.1163010  0.220710     4500
## ExpDay:(Intercept).FishId      -0.0075542 -0.0107889 -0.004660     4500
## (Intercept):ExpDay.FishId      -0.0075542 -0.0107889 -0.004660     4500
## ExpDay:ExpDay.FishId            0.0007863  0.0005542  0.001055     3278
##
##               ~us(1 + ExpDay):MomId
##
##                                post.mean   l-95% CI u-95% CI eff.samp
## (Intercept):(Intercept).MomId 1.5845927  0.0550192 3.822489     1102
## ExpDay:(Intercept).MomId      0.0088615 -0.0107357 0.037286     4500
## (Intercept):ExpDay.MomId      0.0088615 -0.0107357 0.037286     4500
## ExpDay:ExpDay.MomId           0.0005397  0.0000456 0.001278     1197
##
##  R-structure:  ~idh(ExpDay_factor):units
##
##                         post.mean l-95% CI u-95% CI eff.samp
## ExpDay_factor0.units     2.1966   2.0240   2.3846     4500
## ExpDay_factor1.units     1.1126   1.0222   1.2017     4729
## ExpDay_factor2.units     0.7127   0.6540   0.7747     4500
## ExpDay_factor3.units     0.6978   0.6389   0.7567     4433
## ExpDay_factor4.units     0.7220   0.6649   0.7843     4500
## ExpDay_factor5.units     0.6239   0.5738   0.6790     4500
## ExpDay_factor6.units     0.7226   0.6650   0.7817     4500
## ExpDay_factor7.units     0.6366   0.5856   0.6868     4500
## ExpDay_factor8.units     0.7501   0.6887   0.8119     4500
## ExpDay_factor9.units     0.7516   0.6945   0.8156     4500
## ExpDay_factor10.units    0.6964   0.6361   0.7557     4500
## ExpDay_factor11.units    0.5225   0.4785   0.5624     4150
## ExpDay_factor12.units    0.6510   0.5989   0.7068     4500
## ExpDay_factor13.units    0.7491   0.6876   0.8075     4500
## ExpDay_factor14.units    0.7476   0.6891   0.8093     4500
## ExpDay_factor15.units    0.8791   0.8096   0.9527     4500
## ExpDay_factor16.units    0.5882   0.5411   0.6336     4500
## ExpDay_factor17.units    0.6330   0.5843   0.6840     4500
## ExpDay_factor18.units    0.5375   0.4947   0.5822     4500
## ExpDay_factor19.units    0.7858   0.7245   0.8556     4500
## ExpDay_factor20.units    0.6452   0.5946   0.7002     4500
## ExpDay_factor21.units    0.6320   0.5805   0.6811     4500
## ExpDay_factor22.units    0.6735   0.6186   0.7259     5002
```

```
## ExpDay_factor23.units    0.7217   0.6655   0.7813     4178
## ExpDay_factor24.units    0.5747   0.5274   0.6189     4500
## ExpDay_factor25.units    0.7469   0.6847   0.8059     4708
## ExpDay_factor26.units    0.6990   0.6415   0.7566     4297
## ExpDay_factor27.units    0.6102   0.5587   0.6623     5139
##
##  Location effects: MedianVel_hourly_scaled ~ PredatorCue * ExpDay + YearBatch
##
##                     post.mean   l-95% CI   u-95% CI eff.samp  pMCMC
## (Intercept)         2.0247720  0.7528697  3.2875938     3225 <2e-04 ***
## PredatorCue        -0.1832828 -0.3336262 -0.0180726     4500 0.0258 *
## ExpDay             -0.0006334 -0.0157992  0.0148186     4500 0.9111
## YearBatch          -0.8902129 -1.3289187 -0.3822815     1757 <2e-04 ***
## PredatorCue:ExpDay  0.0008967 -0.0097537  0.0125925     4500 0.8649
## ---
## Signif. codes:  0 '***' 0.001 '**' 0.01 '*' 0.05 '.' 0.1 ' ' 1
```

Including YearBatch and Hour improves the fit of the model.

Testing for effect of body size

Next, we will look at the importance of body size. Not all individuals have body size data because not every individual has data through day 27. Here we will prepare model comparison only including individuals with body size data.

Body size model comparison

```r
# With body size
model_MedianVel_bodysize <- readRDS("model_MedianVel_bodysize_9_23_25.rds")
# model_MedianVel_bodysize <-
# MCMCglmm(MedianVel_hourly_scaled ~ PredatorCue*ExpDay +
# YearBatch + Hour + avg_length + avg_width, random = ~us(1
# + ExpDay):FishId + us(1 + ExpDay):MomId, rcov = ~units,
# family = 'gaussian', data = bodysize_only_data, prior =
# prior.univariate.FishId_slopes.MomId_slopes, nitt =
# 500000, burnin = 50000, thin = 100)
# saveRDS(model_MedianVel_bodysize, file =
# 'model_MedianVel_bodysize_9_23_25.rds')
summary(model_MedianVel_bodysize)
```

```
##
##  Iterations = 50001:499901
##  Thinning interval  = 100
##  Sample size  = 4500
##
##  DIC: 5019.762
##
##  G-structure:  ~us(1 + ExpDay):FishId
##
##                                post.mean   l-95% CI  u-95% CI eff.samp
## (Intercept):(Intercept).FishId  0.2490070  0.1511553  0.354485     4435
## ExpDay:(Intercept).FishId      -0.0101772 -0.0155733 -0.005432     4306
## (Intercept):ExpDay.FishId      -0.0101772 -0.0155733 -0.005432     4306
## ExpDay:ExpDay.FishId            0.0007993  0.0004901  0.001164     4604
##
##                ~us(1 + ExpDay):MomId
##
##                               post.mean   l-95% CI  u-95% CI eff.samp
## (Intercept):(Intercept).MomId  0.1209541  2.536e-10 0.3309233     3244
## ExpDay:(Intercept).MomId      -0.0004359 -7.401e-03 0.0047258     3816
## (Intercept):ExpDay.MomId      -0.0004359 -7.401e-03 0.0047258     3816
## ExpDay:ExpDay.MomId            0.0002619  7.856e-09 0.0007019     3568
##
```

```
##  R-structure:  ~units
##
##       post.mean l-95% CI u-95% CI eff.samp
## units    0.4221   0.3996   0.4486     4500
##
##  Location effects: MedianVel_hourly_scaled ~ PredatorCue * ExpDay + YearBatch + Hour + avg_length + avg_w
##
##                     post.mean  l-95% CI  u-95% CI eff.samp    pMCMC
## (Intercept)          0.813898 -0.460117  2.198946     4042 0.231111
## PredatorCue         -0.409120 -0.642183 -0.173971     4500 0.000889 ***
## ExpDay              -0.020131 -0.033898 -0.007568     4855 0.004889 **
## YearBatch           -0.083145 -0.279939  0.103497     3369 0.362222
## avg_length          -0.006283 -0.029338  0.016905     4500 0.588000
## avg_width            0.018693 -0.129626  0.153753     4570 0.783111
## PredatorCue:ExpDay   0.010933 -0.002358  0.024227     4500 0.114222
## ---
## Signif. codes:  0 '***' 0.001 '**' 0.01 '*' 0.05 '.' 0.1 ' ' 1
```

```r
# Without body size
model_MedianVel_no_bodysize <- readRDS("model_MedianVel_no_bodysize_9_23_25.rds")
# model_MedianVel_no_bodysize <-
# MCMCglmm(MedianVel_hourly_scaled ~ PredatorCue*ExpDay +
# YearBatch + Hour, random = ~us(1 + ExpDay):FishId + us(1
# + ExpDay):MomId, rcov = ~units, family = 'gaussian', data
# = bodysize_only_data, prior =
# prior.univariate.FishId_slopes.MomId_slopes, nitt =
# 500000, burnin = 50000, thin = 100)
# saveRDS(model_MedianVel_no_bodysize, file =
# 'model_MedianVel_no_bodysize_9_23_25.rds')
summary(model_MedianVel_no_bodysize)
```

```
##
##  Iterations = 50001:499901
##  Thinning interval  = 100
##  Sample size  = 4500
##
##  DIC: 5019.343
##
##  G-structure:  ~us(1 + ExpDay):FishId
##
##                                post.mean   l-95% CI  u-95% CI eff.samp
## (Intercept):(Intercept).FishId  0.2450431  0.1501006  0.345484     4064
## ExpDay:(Intercept).FishId      -0.0102233 -0.0154463 -0.005379     3902
## (Intercept):ExpDay.FishId      -0.0102233 -0.0154463 -0.005379     3902
## ExpDay:ExpDay.FishId            0.0008026  0.0004702  0.001146     3999
##
##                ~us(1 + ExpDay):MomId
##
##                               post.mean   l-95% CI  u-95% CI eff.samp
## (Intercept):(Intercept).MomId  0.1279167  3.839e-07 0.3508027     2621
## ExpDay:(Intercept).MomId      -0.0003157 -6.736e-03 0.0051295     3635
## (Intercept):ExpDay.MomId      -0.0003157 -6.736e-03 0.0051295     3635
## ExpDay:ExpDay.MomId            0.0002576  8.421e-11 0.0006746     3152
##
##  R-structure:  ~units
##
##       post.mean l-95% CI u-95% CI eff.samp
## units    0.4221   0.3973   0.4463     4500
##
##  Location effects: MedianVel_hourly_scaled ~ PredatorCue * ExpDay + YearBatch + Hour
##
##                     post.mean  l-95% CI  u-95% CI eff.samp    pMCMC
```

```
## (Intercept)          0.722838  0.271679  1.266186     4125 0.002667 **
## PredatorCue         -0.413477 -0.654683 -0.187404     4500 0.000889 ***
## ExpDay              -0.019995 -0.033156 -0.006814     4745 0.003556 **
## YearBatch           -0.080702 -0.284498  0.108037     4716 0.390222
## PredatorCue:ExpDay   0.010871 -0.003061  0.024301     4500 0.116889
## ---
## Signif. codes:  0 '***' 0.001 '**' 0.01 '*' 0.05 '.' 0.1 ' ' 1
```

Without body size is a better fit, so we exclude it from further model comparison and can use the full data set moving forward.

Our final model includes: Fixed effects: PredatorCue*ExpDay (day of life), YearBatch (time of year), Hour (time of day).
Random effects: FishId (individual ID + slopes), MomId (mother's ID + slopes)

Set model:

```
model <- model_MedianVel_big_univariate_id_slopes_mom_slopes_hetero_residual
```

Model diagnostics

```
plot(model$Sol)  # Plots look good
```

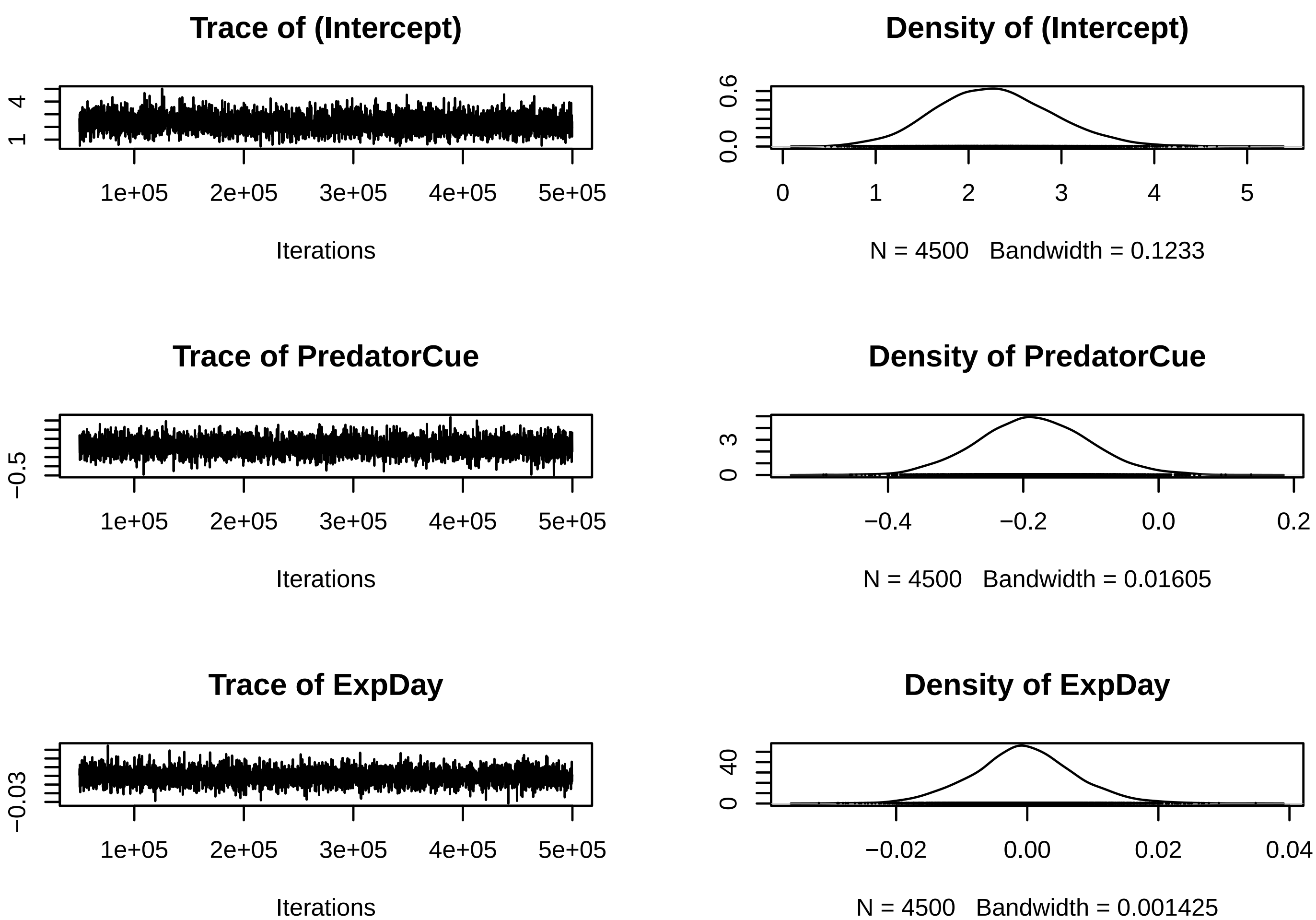

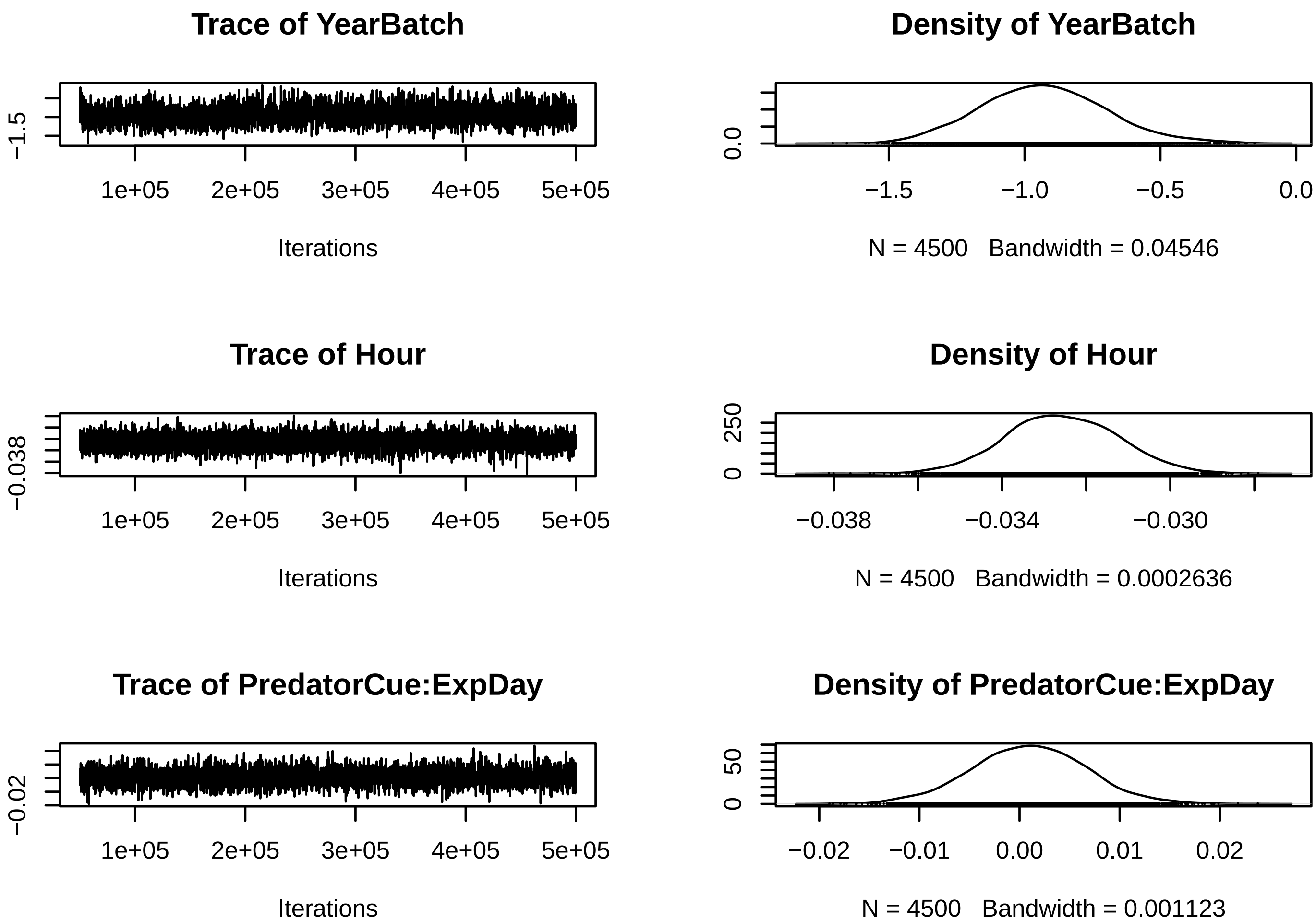


```
autocorr.diag(model$VCV)
```

```
##          (Intercept):(Intercept).FishId ExpDay:(Intercept).FishId
## Lag 0                       1.000000000               1.000000000
## Lag 100                    -0.008963315               0.006781369
## Lag 500                    -0.001119567              -0.005451599
## Lag 1000                   -0.002956833               0.010826376
## Lag 5000                    0.001198088               0.023982318
##          (Intercept):ExpDay.FishId ExpDay:ExpDay.FishId
## Lag 0                  1.000000000          1.000000000
## Lag 100                0.006781369          0.019250323
## Lag 500               -0.005451599         -0.007433310
## Lag 1000               0.010826376          0.008817522
## Lag 5000               0.023982318          0.053679868
##          (Intercept):(Intercept).MomId ExpDay:(Intercept).MomId
## Lag 0                      1.000000000             1.0000000000
## Lag 100                    0.012243927            -0.0049491777
## Lag 500                    0.007910695             0.0029552211
## Lag 1000                  -0.002388612            -0.0145048273
## Lag 5000                   0.015043453            -0.0005431909
##          (Intercept):ExpDay.MomId ExpDay:ExpDay.MomId ExpDay_factor0.units
## Lag 0                1.0000000000         1.000000000          1.000000000
## Lag 100             -0.0049491777         0.125078787          0.008045748
## Lag 500              0.0029552211         0.064990995         -0.029035029
## Lag 1000            -0.0145048273         0.064358019         -0.023144515
## Lag 5000            -0.0005431909         0.009083241          0.007082402
##          ExpDay_factor1.units ExpDay_factor2.units ExpDay_factor3.units
## Lag 0             1.000000000          1.000000000          1.000000000
## Lag 100          -0.006595881          0.004508651          0.009104159
```

```
## Lag 500           0.019019767          -0.006705305          -0.030534516
## Lag 1000         -0.009945495           0.005774515           0.008648662
## Lag 5000          0.006163187           0.003065201           0.002137660
##            ExpDay_factor4.units ExpDay_factor5.units ExpDay_factor6.units
## Lag 0             1.000000000           1.000000000           1.000000000
## Lag 100           0.006647675           0.006427960          -0.011975117
## Lag 500          -0.015281831           0.009090186          -0.009196761
## Lag 1000          0.003634143          -0.007319200           0.005198965
## Lag 5000          0.010286732          -0.002736530          -0.007595444
##            ExpDay_factor7.units ExpDay_factor8.units ExpDay_factor9.units
## Lag 0             1.000000000           1.000000000           1.000000000
## Lag 100          -0.009448315          -0.021897499           0.001990896
## Lag 500           0.026380403          -0.008329907           0.008589029
## Lag 1000         -0.007150801          -0.002211355          -0.009510209
## Lag 5000         -0.015499483           0.020304242          -0.038934066
##            ExpDay_factor10.units ExpDay_factor11.units ExpDay_factor12.units
## Lag 0              1.000000000            1.000000000            1.000000000
## Lag 100           -0.033278617           -0.022860072            0.001596950
## Lag 500            0.001269883            0.004742622            0.022931598
## Lag 1000          -0.007094619           -0.009838811            0.019218989
## Lag 5000          -0.012902415           -0.015085303            0.001615337
##            ExpDay_factor13.units ExpDay_factor14.units ExpDay_factor15.units
## Lag 0              1.000000000            1.000000000            1.000000000
## Lag 100            0.029603504            0.010200249            0.022747503
## Lag 500            0.006872711            0.010029403           -0.027156543
## Lag 1000           0.009632267            0.003935714            0.002949859
## Lag 5000          -0.013475886            0.018895430            0.029925607
##            ExpDay_factor16.units ExpDay_factor17.units ExpDay_factor18.units
## Lag 0             1.0000000000            1.000000000           1.000000e+00
## Lag 100           0.0209713768            0.003558411          -7.339539e-05
## Lag 500          -0.0005505733            0.004258212          -1.334249e-02
## Lag 1000         -0.0049197629            0.008554277           2.636815e-03
## Lag 5000          0.0137414572            0.005902721           7.492596e-03
##            ExpDay_factor19.units ExpDay_factor20.units ExpDay_factor21.units
## Lag 0               1.00000000             1.00000000             1.00000000
## Lag 100             0.01542926            -0.04999703            -0.01641987
## Lag 500             0.01880887            -0.01415466            -0.02676256
## Lag 1000            0.01089185             0.02425967            -0.02574246
## Lag 5000           -0.01488791             0.01289179            -0.01371410
##            ExpDay_factor22.units ExpDay_factor23.units ExpDay_factor24.units
## Lag 0              1.000000000            1.000000000            1.000000000
## Lag 100           -0.022987411            0.004110116           -0.034103343
## Lag 500            0.005588857           -0.005027424            0.016824393
## Lag 1000          -0.015252874           -0.031721351           -0.004451490
## Lag 5000          -0.007157472            0.011850736            0.001056068
##            ExpDay_factor25.units ExpDay_factor26.units ExpDay_factor27.units
## Lag 0              1.000000000            1.000000000             1.00000000
## Lag 100           -0.006581606            0.004150491            -0.01450604
## Lag 500           -0.005433707            0.017854309            -0.01806989
## Lag 1000          -0.031687733           -0.013741120             0.01630231
## Lag 5000          -0.001805603           -0.011973213            -0.01191141
```

```
autocorr.diag(model$Sol)  # Autocorr all well below 0.1
```

```
##             (Intercept)  PredatorCue       ExpDay   YearBatch        Hour
## Lag 0      1.0000000000  1.000000000  1.000000000 1.000000000  1.00000000
## Lag 100    0.0164101837 -0.005079525  0.014013306 0.030614110 -0.02773782
## Lag 500    0.0209408396  0.018966398 -0.007741822 0.025210027  0.01105990
## Lag 1000   0.0006462063 -0.014223930 -0.012908098 0.003953217  0.01010244
## Lag 5000   0.0004960909  0.001817479 -0.012794741 0.019892258  0.01436118
##            PredatorCue:ExpDay
## Lag 0              1.00000000
```

```
## Lag 100         -0.01690643
## Lag 500          0.01013667
## Lag 1000        -0.02445969
## Lag 5000        -0.02268434
```

```r
effectiveSize(model$VCV)
```

```
## (Intercept):(Intercept).FishId      ExpDay:(Intercept).FishId
##                        4500.000                       2443.702
##      (Intercept):ExpDay.FishId           ExpDay:ExpDay.FishId
##                        2443.702                       1632.531
##  (Intercept):(Intercept).MomId       ExpDay:(Intercept).MomId
##                        4500.000                       4500.000
##       (Intercept):ExpDay.MomId            ExpDay:ExpDay.MomId
##                        4500.000                       1035.408
##            ExpDay_factor0.units           ExpDay_factor1.units
##                        4500.000                       4766.705
##            ExpDay_factor2.units           ExpDay_factor3.units
##                        4500.000                       5095.696
##            ExpDay_factor4.units           ExpDay_factor5.units
##                        4500.000                       4500.000
##            ExpDay_factor6.units           ExpDay_factor7.units
##                        4500.000                       4500.000
##            ExpDay_factor8.units           ExpDay_factor9.units
##                        4700.445                       4500.000
##           ExpDay_factor10.units          ExpDay_factor11.units
##                        4808.749                       4709.507
##           ExpDay_factor12.units          ExpDay_factor13.units
##                        4500.000                       4240.286
##           ExpDay_factor14.units          ExpDay_factor15.units
##                        4500.000                       4298.870
##           ExpDay_factor16.units          ExpDay_factor17.units
##                        4500.000                       4500.000
##           ExpDay_factor18.units          ExpDay_factor19.units
##                        4439.051                       4500.000
##           ExpDay_factor20.units          ExpDay_factor21.units
##                        4972.549                       4500.000
##           ExpDay_factor22.units          ExpDay_factor23.units
##                        4710.707                       4500.000
##           ExpDay_factor24.units          ExpDay_factor25.units
##                        6015.589                       4500.000
##           ExpDay_factor26.units          ExpDay_factor27.units
##                        4500.000                       4500.000
```

```r
effectiveSize(model$Sol)  # Effective sample size all high (well above 1000)
```

```
##        (Intercept)        PredatorCue             ExpDay          YearBatch
##           4500.000           3989.616           4500.000           3317.311
##               Hour PredatorCue:ExpDay
##           4562.756           4560.487
```

Model outputs

```r
summary(model)
```

```
##
##  Iterations = 50001:499901
##  Thinning interval  = 100
##  Sample size  = 4500
##
##  DIC: 81650.91
##
##  G-structure:  ~us(1 + ExpDay):FishId
```

```
##
##                              post.mean   l-95% CI  u-95% CI eff.samp
## (Intercept):(Intercept).FishId  0.1657317  0.1167728  0.220064     4500
## ExpDay:(Intercept).FishId      -0.0076094 -0.0106039 -0.004351     2444
## (Intercept):ExpDay.FishId      -0.0076094 -0.0106039 -0.004351     2444
## ExpDay:ExpDay.FishId            0.0007951  0.0005624  0.001076     1633
##
##                ~us(1 + ExpDay):MomId
##
##                               post.mean   l-95% CI u-95% CI eff.samp
## (Intercept):(Intercept).MomId 1.6979561  6.337e-02 4.040944     4500
## ExpDay:(Intercept).MomId      0.0095698 -1.520e-02 0.034627     4500
## (Intercept):ExpDay.MomId      0.0095698 -1.520e-02 0.034627     4500
## ExpDay:ExpDay.MomId           0.0005362  7.511e-06 0.001292     1035
##
##  R-structure:  ~idh(ExpDay_factor):units
##
##                       post.mean l-95% CI u-95% CI eff.samp
## ExpDay_factor0.units     2.1265   1.9544   2.3019     4500
## ExpDay_factor1.units     1.0691   0.9833   1.1576     4767
## ExpDay_factor2.units     0.6748   0.6205   0.7331     4500
## ExpDay_factor3.units     0.6755   0.6197   0.7340     5096
## ExpDay_factor4.units     0.6914   0.6364   0.7514     4500
## ExpDay_factor5.units     0.6220   0.5713   0.6736     4500
## ExpDay_factor6.units     0.6993   0.6419   0.7575     4500
## ExpDay_factor7.units     0.6281   0.5795   0.6813     4500
## ExpDay_factor8.units     0.7375   0.6797   0.7967     4700
## ExpDay_factor9.units     0.7284   0.6697   0.7871     4500
## ExpDay_factor10.units    0.6903   0.6320   0.7482     4809
## ExpDay_factor11.units    0.5168   0.4750   0.5602     4710
## ExpDay_factor12.units    0.6501   0.5999   0.7039     4500
## ExpDay_factor13.units    0.7330   0.6744   0.7898     4240
## ExpDay_factor14.units    0.7524   0.6913   0.8132     4500
## ExpDay_factor15.units    0.8796   0.8081   0.9470     4299
## ExpDay_factor16.units    0.5789   0.5308   0.6253     4500
## ExpDay_factor17.units    0.6403   0.5878   0.6924     4500
## ExpDay_factor18.units    0.5292   0.4862   0.5731     4439
## ExpDay_factor19.units    0.7942   0.7301   0.8572     4500
## ExpDay_factor20.units    0.6357   0.5847   0.6871     4973
## ExpDay_factor21.units    0.6273   0.5781   0.6793     4500
## ExpDay_factor22.units    0.6685   0.6152   0.7208     4711
## ExpDay_factor23.units    0.7035   0.6490   0.7611     4500
## ExpDay_factor24.units    0.5554   0.5125   0.6051     6016
## ExpDay_factor25.units    0.7375   0.6763   0.7976     4500
## ExpDay_factor26.units    0.6740   0.6176   0.7315     4500
## ExpDay_factor27.units    0.5991   0.5526   0.6553     4500
##
##  Location effects: MedianVel_hourly_scaled ~ PredatorCue * ExpDay + YearBatch + Hour
##
##                    post.mean   l-95% CI   u-95% CI eff.samp  pMCMC
## (Intercept)         2.2811848  1.0791251  3.5627985     4500 <2e-04 ***
## PredatorCue        -0.1848312 -0.3402910 -0.0202992     3990 0.0284 *
## ExpDay             -0.0005537 -0.0165530  0.0146559     4500 0.9307
## YearBatch          -0.9294984 -1.3893853 -0.4847935     3317 <2e-04 ***
## Hour               -0.0325741 -0.0351616 -0.0299407     4563 <2e-04 ***
## PredatorCue:ExpDay  0.0009519 -0.0104429  0.0122351     4560 0.8640
## ---
## Signif. codes:  0 '***' 0.001 '**' 0.01 '*' 0.05 '.' 0.1 ' ' 1
```

```
summary(model$Sol)
```

```
##
```

```
## Iterations = 50001:499901
## Thinning interval = 100
## Number of chains = 1
## Sample size per chain = 4500
##
## 1. Empirical mean and standard deviation for each variable,
##    plus standard error of the mean:
##
##                          Mean       SD  Naive SE Time-series SE
## (Intercept)         2.2811848 0.633294 9.441e-03      9.441e-03
## PredatorCue        -0.1848312 0.081447 1.214e-03      1.289e-03
## ExpDay             -0.0005537 0.007840 1.169e-04      1.169e-04
## YearBatch          -0.9294984 0.230667 3.439e-03      4.005e-03
## Hour               -0.0325741 0.001338 1.994e-05      1.980e-05
## PredatorCue:ExpDay  0.0009519 0.005699 8.495e-05      8.439e-05
##
## 2. Quantiles for each variable:
##
##                        2.5%       25%        50%       75%    97.5%
## (Intercept)         1.09388  1.851341  2.2590637  2.689633  3.58494
## PredatorCue        -0.34369 -0.239215 -0.1856741 -0.129981 -0.02223
## ExpDay             -0.01614 -0.005397 -0.0006194  0.004290  0.01522
## YearBatch          -1.36013 -1.090418 -0.9339641 -0.779459 -0.44785
## Hour               -0.03520 -0.033486 -0.0325992 -0.031656 -0.02996
## PredatorCue:ExpDay -0.01047 -0.002847  0.0009960  0.004792  0.01221
```

```r
summary(model$VCV)
```

```
##
## Iterations = 50001:499901
## Thinning interval = 100
## Number of chains = 1
## Sample size per chain = 4500
##
## 1. Empirical mean and standard deviation for each variable,
##    plus standard error of the mean:
##
##                                     Mean        SD  Naive SE Time-series SE
## (Intercept):(Intercept).FishId  0.1657317 0.0274496 4.092e-04      4.092e-04
## ExpDay:(Intercept).FishId      -0.0076094 0.0016259 2.424e-05      3.289e-05
## (Intercept):ExpDay.FishId      -0.0076094 0.0016259 2.424e-05      3.289e-05
## ExpDay:ExpDay.FishId            0.0007951 0.0001348 2.009e-06      3.336e-06
## (Intercept):(Intercept).MomId   1.6979561 1.2538725 1.869e-02      1.869e-02
## ExpDay:(Intercept).MomId        0.0095698 0.0129306 1.928e-04      1.928e-04
## (Intercept):ExpDay.MomId        0.0095698 0.0129306 1.928e-04      1.928e-04
## ExpDay:ExpDay.MomId             0.0005362 0.0003910 5.829e-06      1.215e-05
## ExpDay_factor0.units            2.1265113 0.0900588 1.343e-03      1.343e-03
## ExpDay_factor1.units            1.0690692 0.0450644 6.718e-04      6.527e-04
## ExpDay_factor2.units            0.6748209 0.0291127 4.340e-04      4.340e-04
## ExpDay_factor3.units            0.6754834 0.0293542 4.376e-04      4.112e-04
## ExpDay_factor4.units            0.6914295 0.0294029 4.383e-04      4.383e-04
## ExpDay_factor5.units            0.6220387 0.0263975 3.935e-04      3.935e-04
## ExpDay_factor6.units            0.6992777 0.0297446 4.434e-04      4.434e-04
## ExpDay_factor7.units            0.6281381 0.0260490 3.883e-04      3.883e-04
## ExpDay_factor8.units            0.7375497 0.0298203 4.445e-04      4.350e-04
## ExpDay_factor9.units            0.7284394 0.0299447 4.464e-04      4.464e-04
## ExpDay_factor10.units           0.6902993 0.0299267 4.461e-04      4.316e-04
## ExpDay_factor11.units           0.5167801 0.0219027 3.265e-04      3.192e-04
## ExpDay_factor12.units           0.6501299 0.0271215 4.043e-04      4.043e-04
## ExpDay_factor13.units           0.7330471 0.0298978 4.457e-04      4.591e-04
## ExpDay_factor14.units           0.7523889 0.0312755 4.662e-04      4.662e-04
## ExpDay_factor15.units           0.8796353 0.0357412 5.328e-04      5.451e-04
```

```
## ExpDay_factor16.units           0.5789025 0.0239070 3.564e-04      3.564e-04
## ExpDay_factor17.units           0.6402633 0.0263492 3.928e-04      3.928e-04
## ExpDay_factor18.units           0.5291565 0.0224317 3.344e-04      3.367e-04
## ExpDay_factor19.units           0.7941789 0.0326395 4.866e-04      4.866e-04
## ExpDay_factor20.units           0.6357435 0.0260912 3.889e-04      3.700e-04
## ExpDay_factor21.units           0.6273130 0.0263003 3.921e-04      3.921e-04
## ExpDay_factor22.units           0.6685049 0.0273070 4.071e-04      3.979e-04
## ExpDay_factor23.units           0.7035062 0.0291539 4.346e-04      4.346e-04
## ExpDay_factor24.units           0.5554302 0.0239263 3.567e-04      3.085e-04
## ExpDay_factor25.units           0.7375412 0.0312942 4.665e-04      4.665e-04
## ExpDay_factor26.units           0.6740411 0.0290810 4.335e-04      4.335e-04
## ExpDay_factor27.units           0.5990611 0.0262032 3.906e-04      3.906e-04
##
## 2. Quantiles for each variable:
##
##                                      2.5%        25%        50%        75%
## (Intercept):(Intercept).FishId  1.202e-01  0.1463497  0.1628249  0.1823338
## ExpDay:(Intercept).FishId      -1.122e-02 -0.0085698 -0.0074552 -0.0064697
## (Intercept):ExpDay.FishId      -1.122e-02 -0.0085698 -0.0074552 -0.0064697
## ExpDay:ExpDay.FishId            5.755e-04  0.0007011  0.0007783  0.0008764
## (Intercept):(Intercept).MomId   2.946e-01  0.8830318  1.3829170  2.1345703
## ExpDay:(Intercept).MomId       -1.034e-02  0.0022408  0.0076982  0.0144558
## (Intercept):ExpDay.MomId       -1.034e-02  0.0022408  0.0076982  0.0144558
## ExpDay:ExpDay.MomId             8.923e-05  0.0002849  0.0004397  0.0006699
## ExpDay_factor0.units            1.959e+00  2.0644127  2.1249500  2.1848998
## ExpDay_factor1.units            9.864e-01  1.0376317  1.0672731  1.0982601
## ExpDay_factor2.units            6.212e-01  0.6546471  0.6741620  0.6936088
## ExpDay_factor3.units            6.205e-01  0.6556226  0.6746179  0.6945218
## ExpDay_factor4.units            6.352e-01  0.6715776  0.6904297  0.7110687
## ExpDay_factor5.units            5.723e-01  0.6041143  0.6212015  0.6401066
## ExpDay_factor6.units            6.435e-01  0.6789353  0.6983386  0.7187293
## ExpDay_factor7.units            5.785e-01  0.6105723  0.6271255  0.6449235
## ExpDay_factor8.units            6.806e-01  0.7173497  0.7365650  0.7572137
## ExpDay_factor9.units            6.709e-01  0.7073422  0.7277284  0.7487483
## ExpDay_factor10.units           6.347e-01  0.6697972  0.6895292  0.7095777
## ExpDay_factor11.units           4.760e-01  0.5017135  0.5162675  0.5312931
## ExpDay_factor12.units           6.003e-01  0.6316708  0.6494100  0.6682098
## ExpDay_factor13.units           6.777e-01  0.7119397  0.7315208  0.7531946
## ExpDay_factor14.units           6.943e-01  0.7306567  0.7515348  0.7728905
## ExpDay_factor15.units           8.120e-01  0.8552984  0.8790733  0.9030879
## ExpDay_factor16.units           5.338e-01  0.5627961  0.5783216  0.5949158
## ExpDay_factor17.units           5.882e-01  0.6228041  0.6396778  0.6577820
## ExpDay_factor18.units           4.876e-01  0.5137548  0.5284674  0.5436456
## ExpDay_factor19.units           7.319e-01  0.7723757  0.7933151  0.8160331
## ExpDay_factor20.units           5.858e-01  0.6177049  0.6354031  0.6529924
## ExpDay_factor21.units           5.785e-01  0.6091789  0.6268240  0.6446507
## ExpDay_factor22.units           6.168e-01  0.6502524  0.6678364  0.6858931
## ExpDay_factor23.units           6.494e-01  0.6832253  0.7027909  0.7226833
## ExpDay_factor24.units           5.116e-01  0.5383612  0.5547676  0.5717111
## ExpDay_factor25.units           6.790e-01  0.7163672  0.7373597  0.7577527
## ExpDay_factor26.units           6.190e-01  0.6542257  0.6731790  0.6934222
## ExpDay_factor27.units           5.488e-01  0.5806969  0.5980885  0.6164561
##                                     97.5%
## (Intercept):(Intercept).FishId  0.226582
## ExpDay:(Intercept).FishId      -0.004830
## (Intercept):ExpDay.FishId      -0.004830
## ExpDay:ExpDay.FishId            0.001099
## (Intercept):(Intercept).MomId   4.942295
## ExpDay:(Intercept).MomId        0.041979
## (Intercept):ExpDay.MomId        0.041979
## ExpDay:ExpDay.MomId             0.001564
```

```
## ExpDay_factor0.units            2.310702
## ExpDay_factor1.units            1.164634
## ExpDay_factor2.units            0.733882
## ExpDay_factor3.units            0.735248
## ExpDay_factor4.units            0.750709
## ExpDay_factor5.units            0.675057
## ExpDay_factor6.units            0.759582
## ExpDay_factor7.units            0.680948
## ExpDay_factor8.units            0.797631
## ExpDay_factor9.units            0.789850
## ExpDay_factor10.units           0.752995
## ExpDay_factor11.units           0.561619
## ExpDay_factor12.units           0.704578
## ExpDay_factor13.units           0.793823
## ExpDay_factor14.units           0.816842
## ExpDay_factor15.units           0.953296
## ExpDay_factor16.units           0.628884
## ExpDay_factor17.units           0.693281
## ExpDay_factor18.units           0.575156
## ExpDay_factor19.units           0.859586
## ExpDay_factor20.units           0.689159
## ExpDay_factor21.units           0.679778
## ExpDay_factor22.units           0.722768
## ExpDay_factor23.units           0.762139
## ExpDay_factor24.units           0.604464
## ExpDay_factor25.units           0.800657
## ExpDay_factor26.units           0.733281
## ExpDay_factor27.units           0.652665
```

Calculating probability of direction

To aid in interpreting treatment differences, in addition to confidence interval overlap, we can look at probability of direction (pd). Pd values can be analogous to p-values in frequentist statistics.

Caluclate probability of direction (pd) for treatment (PredatorCue):

```
# Treatment
post_diff <- model$Sol[, "PredatorCue"]
pd <- mean(post_diff < 0)
pd  # 0.986
```

```
## [1] 0.9857778
```

```
ppvalue_comparison <- pd_to_p(pd = pd)
ppvalue_comparison  # 0.028
```

```
## [1] 0.02844444
```

```
# Treatment x Time
post_diff <- model$Sol[, "PredatorCue:ExpDay"]
pd <- mean(post_diff > 0)
pd  # 0.568
```

```
## [1] 0.568
```

```
ppvalue_comparison <- pd_to_p(pd = pd)
ppvalue_comparison  # 0.864
```

```
## [1] 0.864
```

Now we can plot median velocity over time

```
## Plotting trait over time
colors <- c(`0` = "#1709b5", `1` = "#ed5715")
```

```r
# Convert pixels per second to cm per second for easier
# visual interpretation of behaviors
day_only_data$MedianVel_cm <- day_only_data$MedianVel * 0.0423

# Median velocity over time with expanded y axis
plot_name <- ggplot(day_only_data, aes(x = (ExpDay + 1), y = MedianVel_cm,
    color = factor(PredatorCue))) + geom_line(aes(group = FishId),
    linewidth = 0.8, alpha = 0.16) + stat_summary(fun = mean,
    geom = "line", aes(group = factor(PredatorCue)), linewidth = 1.5,
    linetype = "solid") + scale_color_manual(values = colors) +
    scale_x_continuous(limits = c(1, 28), breaks = seq(1, max(28,
        na.rm = TRUE), by = 3)) + scale_y_continuous(limits = c(0,
    1.6), breaks = seq(0, max(1.6, na.rm = TRUE), by = 0.4)) +
    theme_classic() + theme(legend.position = "none", axis.line = element_line(linewidth = 0.5,
    colour = "black"), axis.ticks = element_line(colour = "black"),
    axis.text = element_text(size = 14, colour = "black"), axis.title = element_text(size = 18,
        colour = "black"), plot.title = element_text(size = 0)) +
    labs(x = "Day", y = "Median velocity (cm per second)")
plot_name
```

```
## Warning: Removed 2 rows containing non-finite outside the scale range
## (`stat_summary()`).
```

```
## Warning: Removed 2 rows containing missing values or values outside the scale range
## (`geom_line()`).
```

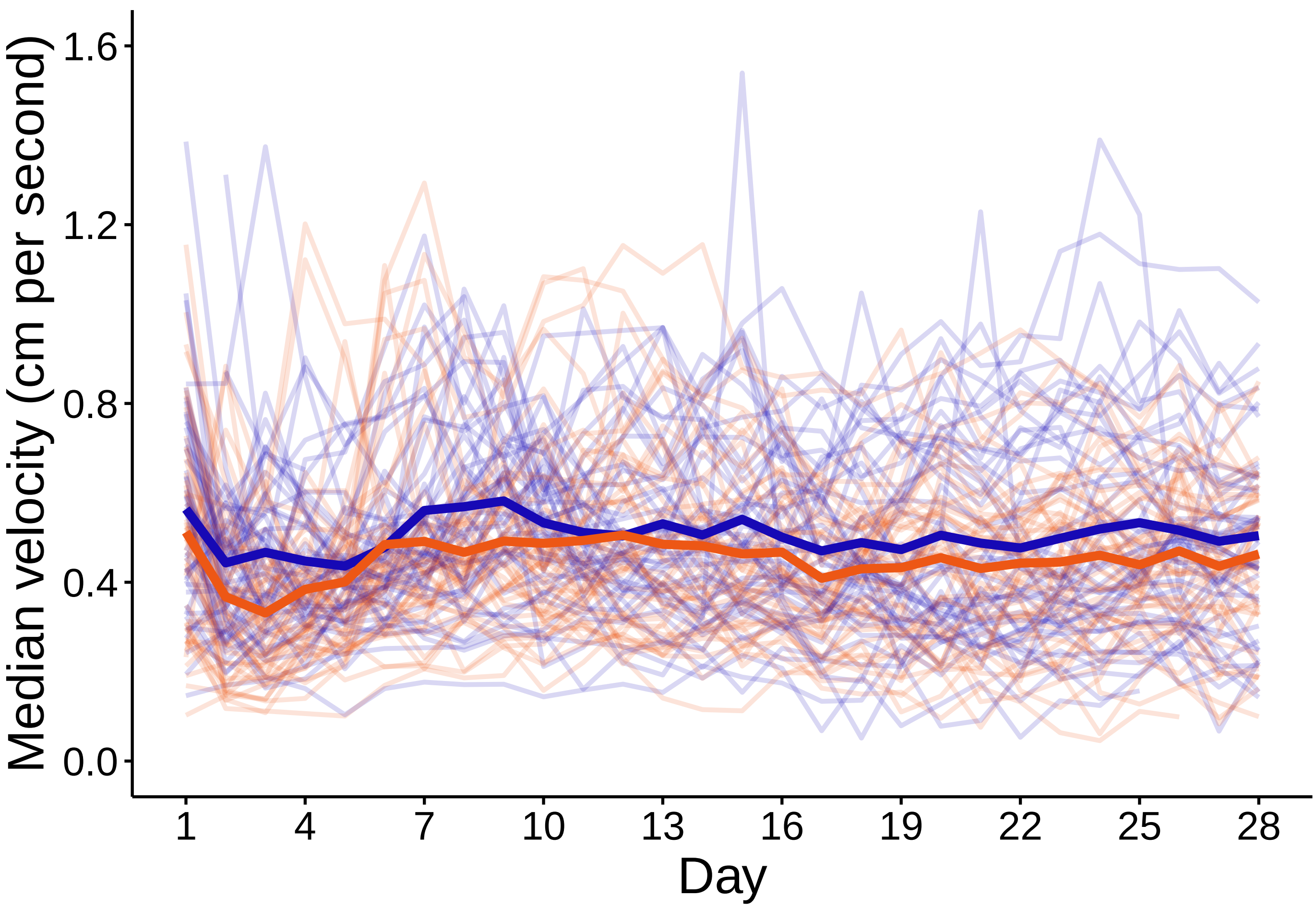


```r
ggsave("velocity_over_time_cm.pdf", plot = plot_name, width = 5,
    height = 5, dpi = 600)
```

```
## Warning: Removed 2 rows containing non-finite outside the scale range
## (`stat_summary()`).
## Removed 2 rows containing missing values or values outside the scale range
## (`geom_line()`).
```

Comparing behavior on day 1

Now we can run the model on a subset of the data to compare behavior on day 1 between treatments:

```
# Make day 1 subset
day1_hourly_data <- subset(hourly_data, ExpDay == 0)

### Model selection to see whether fixed effects are still
### relevant when only looking at day 1

# Including YearBatch and Hour
model_MedianVel_day1_YearBach_Hour <- readRDS("model_MedianVel_day1_YearBatch_Hour_10_14_25.rds")
# model_MedianVel_day1_YearBach_Hour <-
# MCMCglmm(MedianVel_hourly_scaled ~ PredatorCue +
# YearBatch + Hour, random = ~FishId + MomId, rcov =
# ~units, family = 'gaussian', data = day1_hourly_data,
# prior = prior.univariate.FishId.MomId, nitt = 500000,
# burnin = 50000, thin = 100)
# saveRDS(model_MedianVel_day1_YearBach_Hour, file =
# 'model_MedianVel_day1_YearBatch_Hour_10_14_25.rds')
summary(model_MedianVel_day1_YearBach_Hour)  #
```

```
##
##  Iterations = 50001:499901
##  Thinning interval  = 100
##  Sample size  = 4500
##
##  DIC: 3445.365
##
##  G-structure:  ~FishId
##
##        post.mean l-95% CI u-95% CI eff.samp
## FishId    0.9236   0.6279    1.232     4245
##
##                ~MomId
##
##       post.mean l-95% CI u-95% CI eff.samp
## MomId    0.5866  0.06523    1.352     4206
##
##  R-structure:  ~units
##
##       post.mean l-95% CI u-95% CI eff.samp
## units     0.965   0.8858    1.048     4500
##
##  Location effects: MedianVel_hourly_scaled ~ PredatorCue + YearBatch + Hour
##
##             post.mean l-95% CI u-95% CI eff.samp  pMCMC
## (Intercept)   1.40007  0.36826  2.51481     4500 0.0164 *
## PredatorCue  -0.43901 -0.83441 -0.04599     4773 0.0298 *
## YearBatch    -0.08613 -0.50088  0.33632     4500 0.6684
## Hour         -0.10081 -0.11724 -0.08452     4500 <2e-04 ***
## ---
## Signif. codes:  0 '***' 0.001 '**' 0.01 '*' 0.05 '.' 0.1 ' ' 1
```

```
# Including only YearBatch
model_MedianVel_day1_YearBach <- readRDS("model_MedianVel_day1_YearBatch_10_14_25.rds")
# model_MedianVel_day1_YearBach <-
# MCMCglmm(MedianVel_hourly_scaled ~ PredatorCue +
```

```r
# YearBatch, random = ~FishId + MomId, rcov = ~units,
# family = 'gaussian', data = day1_hourly_data, prior =
# prior.univariate.FishId.MomId, nitt = 500000, burnin =
# 50000, thin = 100) saveRDS(model_MedianVel_day1_YearBach,
# file = 'model_MedianVel_day1_YearBatch_10_14_25.rds')
summary(model_MedianVel_day1_YearBach)  #
```

```
##
##  Iterations = 50001:499901
##  Thinning interval  = 100
##  Sample size  = 4500
##
##  DIC: 3596.355
##
##  G-structure:  ~FishId
##
##        post.mean l-95% CI u-95% CI eff.samp
## FishId    0.9112   0.6338    1.234     4500
##
##                ~MomId
##
##       post.mean l-95% CI u-95% CI eff.samp
## MomId    0.5969  0.08157    1.409     4500
##
##  R-structure:  ~units
##
##       post.mean l-95% CI u-95% CI eff.samp
## units     1.095    1.007    1.192     4417
##
##  Location effects: MedianVel_hourly_scaled ~ PredatorCue + YearBatch
##
##             post.mean l-95% CI u-95% CI eff.samp  pMCMC
## (Intercept)   0.83649 -0.24668  1.92507     4500 0.1209
## PredatorCue  -0.43145 -0.84549 -0.05529     4500 0.0364 *
## YearBatch    -0.08006 -0.48173  0.39854     4500 0.6809
## ---
## Signif. codes:  0 '***' 0.001 '**' 0.01 '*' 0.05 '.' 0.1 ' ' 1
```

```r
# Including only Hour
model_MedianVel_day1_Hour <- readRDS("model_MedianVel_day1_Hour_10_14_25.rds")
# model_MedianVel_day1_Hour <-
# MCMCglmm(MedianVel_hourly_scaled ~ PredatorCue + Hour,
# random = ~FishId + MomId, rcov = ~units, family =
# 'gaussian', data = day1_hourly_data, prior =
# prior.univariate.FishId.MomId, nitt = 500000, burnin =
# 50000, thin = 100) saveRDS(model_MedianVel_day1_Hour,
# file = 'model_MedianVel_day1_Hour_10_14_25.rds')
summary(model_MedianVel_day1_Hour)  # Best fit
```

```
##
##  Iterations = 50001:499901
##  Thinning interval  = 100
##  Sample size  = 4500
##
##  DIC: 3445.385
##
##  G-structure:  ~FishId
##
##        post.mean l-95% CI u-95% CI eff.samp
## FishId    0.9267   0.6413    1.243     4500
##
##                ~MomId
```

```
##
##       post.mean l-95% CI u-95% CI eff.samp
## MomId    0.5319  0.06842    1.202     4277
##
##  R-structure:  ~units
##
##       post.mean l-95% CI u-95% CI eff.samp
## units    0.9655   0.8914    1.048     4109
##
##  Location effects: MedianVel_hourly_scaled ~ PredatorCue + Hour
##
##             post.mean l-95% CI u-95% CI eff.samp  pMCMC
## (Intercept)   1.21129  0.74127  1.74680     4500 <2e-04 ***
## PredatorCue  -0.43978 -0.84242 -0.04262     4500 0.0307 *
## Hour         -0.10078 -0.11665 -0.08530     4500 <2e-04 ***
## ---
## Signif. codes:  0 '***' 0.001 '**' 0.01 '*' 0.05 '.' 0.1 ' ' 1
```

```r
# Excluding YearBatch and Hour
model_MedianVel_day1 <- readRDS("model_MedianVel_day1_10_14_25.rds")
# model_MedianVel_day1 <- MCMCglmm(MedianVel_hourly_scaled
# ~ PredatorCue, random = ~FishId + MomId, rcov = ~units,
# family = 'gaussian', data = day1_hourly_data, prior =
# prior.univariate.FishId.MomId, nitt = 500000, burnin =
# 50000, thin = 100) saveRDS(model_MedianVel_day1, file =
# 'model_MedianVel_day1_10_14_25.rds')
summary(model_MedianVel_day1)  #
```

```
##
##  Iterations = 50001:499901
##  Thinning interval  = 100
##  Sample size  = 4500
##
##  DIC: 3596.228
##
##  G-structure:  ~FishId
##
##        post.mean l-95% CI u-95% CI eff.samp
## FishId    0.9105   0.6265    1.237     4500
##
##                ~MomId
##
##       post.mean l-95% CI u-95% CI eff.samp
## MomId    0.5137  0.05968    1.136     4266
##
##  R-structure:  ~units
##
##       post.mean l-95% CI u-95% CI eff.samp
## units     1.097    1.005     1.19     4221
##
##  Location effects: MedianVel_hourly_scaled ~ PredatorCue
##
##             post.mean l-95% CI u-95% CI eff.samp  pMCMC
## (Intercept)   0.65383  0.14615  1.12747     4500 0.0107 *
## PredatorCue  -0.43365 -0.82393 -0.03314     4500 0.0333 *
## ---
## Signif. codes:  0 '***' 0.001 '**' 0.01 '*' 0.05 '.' 0.1 ' ' 1
```

```r
day1_model <- model_MedianVel_day1_Hour
summary(day1_model)
```

```
##
##  Iterations = 50001:499901
```

```
##  Thinning interval  = 100
##  Sample size  = 4500
##
##  DIC: 3445.385
##
##  G-structure:  ~FishId
##
##        post.mean l-95% CI u-95% CI eff.samp
## FishId    0.9267   0.6413    1.243     4500
##
##                 ~MomId
##
##       post.mean l-95% CI u-95% CI eff.samp
## MomId    0.5319  0.06842    1.202     4277
##
##  R-structure:  ~units
##
##       post.mean l-95% CI u-95% CI eff.samp
## units    0.9655   0.8914    1.048     4109
##
##  Location effects: MedianVel_hourly_scaled ~ PredatorCue + Hour
##
##             post.mean l-95% CI u-95% CI eff.samp  pMCMC
## (Intercept)   1.21129  0.74127  1.74680     4500 <2e-04 ***
## PredatorCue  -0.43978 -0.84242 -0.04262     4500 0.0307 *
## Hour         -0.10078 -0.11665 -0.08530     4500 <2e-04 ***
## ---
## Signif. codes:  0 '***' 0.001 '**' 0.01 '*' 0.05 '.' 0.1 ' ' 1
```

Calculate probability of direction (pd) for treatment (PredatorCue):

```
post_samples <- as.data.frame(day1_model$Sol)
post_diff <- post_samples[, "PredatorCue"]
pd <- mean(post_diff < 0)
pd  # 0.985
```

```
## [1] 0.9846667
```

```
ppvalue_comparison <- pd_to_p(pd = pd)
ppvalue_comparison  # 0.031
```

```
## [1] 0.03066667
```

Calculate ICCs for variance components:

```
FishId_intercept <- model$VCV[, "(Intercept):(Intercept).FishId"]
FishId_slope <- model$VCV[, "ExpDay:ExpDay.FishId"]
FishId_cov <- model$VCV[, "(Intercept):ExpDay.FishId"]

MomId_intercept <- model$VCV[, "(Intercept):(Intercept).MomId"]
MomId_slope <- model$VCV[, "ExpDay:ExpDay.MomId"]
MomId_cov <- model$VCV[, "(Intercept):ExpDay.MomId"]

days <- 0:27
mean_days <- mean(days)
mean_days_sq <- mean(days^2)

res_columns <- paste0("ExpDay_factor", days, ".units")
residual_var <- rowMeans(model$VCV[, res_columns, drop = FALSE])

FishId_var <- FishId_intercept + 2 * FishId_cov * mean_days +
    FishId_slope * mean_days_sq
MomId_var <- MomId_intercept + 2 * MomId_cov * mean_days + MomId_slope *
```

```r
    mean_days_sq

# ICC calculations
ICC_FishId <- FishId_var/(FishId_var + residual_var)
ICC_FishId_output <- c(median = median(ICC_FishId), HPDinterval(as.mcmc(ICC_FishId))[1,
    ])
ICC_FishId_withmom <- FishId_var/(FishId_var + MomId_var + residual_var)
ICC_FishId_withmom_output <- c(median = median(ICC_FishId_withmom),
    HPDinterval(as.mcmc(ICC_FishId_withmom))[1, ])
ICC_MomId <- MomId_var/(FishId_var + MomId_var + residual_var)
ICC_MomId_output <- c(median = median(ICC_MomId), HPDinterval(as.mcmc(ICC_MomId))[1,
    ])
ICC_residual <- residual_var/(FishId_var + MomId_var + residual_var)
ICC_residual_output <- c(median = median(ICC_residual), HPDinterval(as.mcmc(ICC_residual))[1,
    ])

ICC_FishId_output  # 0.174 [0.140, 0.212]
```

```
##    median     lower     upper
## 0.1741097 0.1398740 0.2116399
```

```r
ICC_FishId_withmom_output  # 0.059 [0.020, 0.113]
```

```
##     median      lower      upper
## 0.05936489 0.01799897 0.11342588
```

```r
ICC_MomId_output  # 0.659 [0.368, 0.893]
```

```
##    median     lower     upper
## 0.6591544 0.3684064 0.8928523
```

```r
ICC_residual_output  # 0.281 [0.089, 0.520]
```

```
##    median     lower     upper
## 0.2811542 0.0887905 0.5204746
```

```r
ICC_data_for_plot <- data.frame(Component = c("Among individuals",
    "Maternal identity", "Within individuals"), ICCs = c(0.059,
    0.659, 0.281), Lower = c(0.02, 0.368, 0.089), Upper = c(0.113,
    0.893, 0.52))

# Plot
plot_name <- ggplot(ICC_data_for_plot, aes(x = Component, y = ICCs)) +
    geom_bar(stat = "identity", fill = "lightgrey", width = 0.6) +
    geom_errorbar(aes(ymin = Lower, ymax = Upper), width = 0.2,
        size = 0.7) + scale_y_continuous(limits = c(0, 0.9),
    breaks = seq(0, 0.9, by = 0.1), expand = c(0, 0)) + theme_classic()
```

```
## Warning: Using `size` aesthetic for lines was deprecated in ggplot2 3.4.0.
## i Please use `linewidth` instead.
## This warning is displayed once every 8 hours.
## Call `lifecycle::last_lifecycle_warnings()` to see where this warning was
## generated.
```

```r
plot_name
```

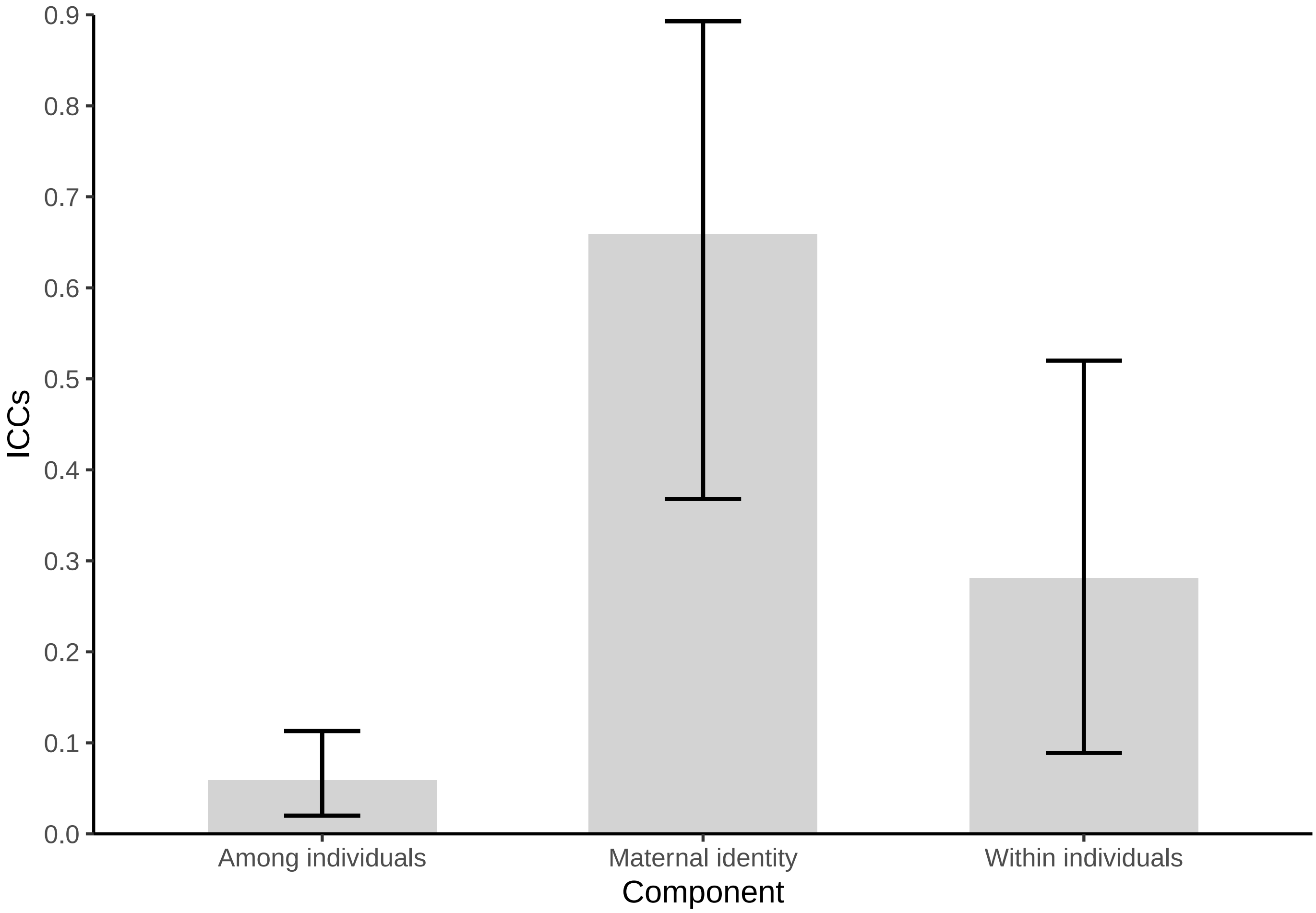


```
ggsave("velocity_ICCs_plot.pdf", plot = plot_name, dpi = 600,
    width = 6, height = 6)
```

Investigating maternal effects

We found that MomId contributes significantly to variance, providing evidence of maternal effects.

Linking mom and offspring behavior

We will first investigate whether mother behavior predicts offspring behavior. Does it matter what mom does, or just who she is? Like our investigation of body size earlier, we look at this using a subset of our data because not all babies have mom behavior data.
First, we will prepare model comparison only including individuals with mom behavior data.

Mom behavior model comparison

Model with mom behavior:

```
# Make day into factor for data subset
mombehavior_only_data_hourly$ExpDay_factor <- as.factor(mombehavior_only_data_hourly$ExpDay)

model_MedianVel_mom_behavior <- readRDS("model_MedianVel_mom_behavior_10_16_25.rds")
# model_MedianVel_mom_behavior <-
# MCMCglmm(MedianVel_hourly_scaled ~ PredatorCue*ExpDay +
# YearBatch + Hour + Avg_MedVel_mom, random = ~us(1 +
# ExpDay):FishId + us(1 + ExpDay):MomId, rcov =
# ~idh(ExpDay_factor):units, family = 'gaussian', data =
# mombehavior_only_data_hourly, prior =
```

```r
# prior.univariate.FishId_slopes.MomId_slopes.heteroResidual,
# nitt = 500000, burnin = 50000, thin = 100)
# saveRDS(model_MedianVel_mom_behavior, file =
# 'model_MedianVel_mom_behavior_10_16_25.rds')
summary(model_MedianVel_mom_behavior)
```

```
##
##  Iterations = 50001:499901
##  Thinning interval  = 100
##  Sample size  = 4500
##
##  DIC: 64518.99
##
##  G-structure:  ~us(1 + ExpDay):FishId
##
##                                post.mean   l-95% CI  u-95% CI eff.samp
## (Intercept):(Intercept).FishId  0.1507861  0.0994541  0.205644     4500
## ExpDay:(Intercept).FishId      -0.0070642 -0.0104983 -0.003908     4500
## (Intercept):ExpDay.FishId      -0.0070642 -0.0104983 -0.003908     4500
## ExpDay:ExpDay.FishId            0.0007205  0.0004857  0.001016     4500
##
##                ~us(1 + ExpDay):MomId
##
##                               post.mean   l-95% CI u-95% CI eff.samp
## (Intercept):(Intercept).MomId  0.2028070  1.561e-04 0.549826     2614
## ExpDay:(Intercept).MomId      -0.0056138 -2.546e-02 0.006967     4500
## (Intercept):ExpDay.MomId      -0.0056138 -2.546e-02 0.006967     4500
## ExpDay:ExpDay.MomId            0.0008145  2.398e-05 0.002020     2011
##
##  R-structure:  ~idh(ExpDay_factor):units
##
##                        post.mean l-95% CI u-95% CI eff.samp
## ExpDay_factor0.units     2.3131   2.1031   2.5145     4500
## ExpDay_factor1.units     1.2159   1.1086   1.3294     4500
## ExpDay_factor2.units     0.7398   0.6712   0.8065     4500
## ExpDay_factor3.units     0.7220   0.6569   0.7905     4500
## ExpDay_factor4.units     0.7864   0.7173   0.8599     4500
## ExpDay_factor5.units     0.7247   0.6607   0.7919     4500
## ExpDay_factor6.units     0.7853   0.7140   0.8583     4500
## ExpDay_factor7.units     0.6914   0.6270   0.7547     4250
## ExpDay_factor8.units     0.8218   0.7516   0.8998     4312
## ExpDay_factor9.units     0.8264   0.7512   0.9022     4500
## ExpDay_factor10.units    0.7102   0.6412   0.7775     5053
## ExpDay_factor11.units    0.4626   0.4205   0.5076     4500
## ExpDay_factor12.units    0.6397   0.5798   0.6981     4500
## ExpDay_factor13.units    0.7434   0.6805   0.8132     4500
## ExpDay_factor14.units    0.5498   0.5026   0.6037     4500
## ExpDay_factor15.units    0.6467   0.5874   0.7036     4500
## ExpDay_factor16.units    0.6072   0.5565   0.6650     4947
## ExpDay_factor17.units    0.5682   0.5192   0.6202     4500
## ExpDay_factor18.units    0.4531   0.4140   0.4965     4500
## ExpDay_factor19.units    0.6906   0.6260   0.7511     4791
## ExpDay_factor20.units    0.5159   0.4702   0.5640     4500
## ExpDay_factor21.units    0.5861   0.5333   0.6386     4500
## ExpDay_factor22.units    0.6402   0.5830   0.6994     4500
## ExpDay_factor23.units    0.6890   0.6281   0.7530     5895
## ExpDay_factor24.units    0.5274   0.4818   0.5754     4269
## ExpDay_factor25.units    0.7421   0.6770   0.8105     4500
## ExpDay_factor26.units    0.6731   0.6141   0.7377     4500
## ExpDay_factor27.units    0.5905   0.5351   0.6472     4730
##
```

```
##  Location effects: MedianVel_hourly_scaled ~ PredatorCue * ExpDay + YearBatch + Hour + Avg_MedVel_mom
##
##                     post.mean  l-95% CI  u-95% CI eff.samp  pMCMC
## (Intercept)         -0.229889 -1.111211  0.599304     4104  0.537
## PredatorCue         -0.181367 -0.348588 -0.001364     4500  0.040 *
## ExpDay              -0.001498 -0.020311  0.019918     4500  0.861
## YearBatch            0.204138 -0.090812  0.528925     4500  0.164
## Hour                -0.028556 -0.031590 -0.025888     4500 <2e-04 ***
## Avg_MedVel_mom       0.005048 -0.016137  0.025058     4324  0.576
## PredatorCue:ExpDay -0.001502 -0.013276  0.010629     4938  0.814
## ---
## Signif. codes:  0 '***' 0.001 '**' 0.01 '*' 0.05 '.' 0.1 ' ' 1
```

Mother behavior does not predict offspring behavior.

Plot mother by offspring behavior by treatment

```
# Create columns for control and predator treatments:
mombehavior_only_data_hourly$MedianVel_control_hourly <- ifelse(mombehavior_only_data_hourly$PredatorCue ==
    0, mombehavior_only_data_hourly$MedianVel_, NA)
mombehavior_only_data_hourly$MedianVel_predator_hourly <- ifelse(mombehavior_only_data_hourly$PredatorCue ==
    1, mombehavior_only_data_hourly$MedianVel_, NA)

# Sum per fish
mean_per_fish <- mombehavior_only_data_hourly %>%
    mutate(OffspringVelocity = ifelse(PredatorCue == 0, MedianVel_control_hourly,
        MedianVel_predator_hourly)) %>%
    group_by(FishId, MomId, Avg_MedVel_mom, PredatorCue) %>%
    summarize(OffspringMeanVel = mean(OffspringVelocity, na.rm = TRUE),
        .groups = "drop")
# Hourly data
raw_hourly <- mombehavior_only_data_hourly %>%
    mutate(OffspringVelocity = ifelse(PredatorCue == 0, MedianVel_control_hourly,
        MedianVel_predator_hourly))
# Set colors
colors <- c(`0` = "#1709b5", `1` = "#ed5715")
larger_fill_colors <- c(`0` = "#8e9ee9", `1` = "#f79a6f")

# Plot with only individual fish means
plot_name <- ggplot() + geom_smooth(data = mean_per_fish, aes(x = Avg_MedVel_mom,
    y = OffspringMeanVel), method = "lm", se = TRUE, color = "grey60",
    fill = "grey70", linetype = "solid", alpha = 0.3, linewidth = 0.5) +
    geom_boxplot(data = mean_per_fish, aes(x = Avg_MedVel_mom,
        y = OffspringMeanVel, group = Avg_MedVel_mom), fill = "white",
        color = "black", alpha = 1, width = 0.9, outlier.shape = NA) +
    geom_point(data = mean_per_fish, aes(x = Avg_MedVel_mom,
        y = OffspringMeanVel, color = factor(PredatorCue)), shape = 16,
        position = position_jitter(width = 0), size = 2, alpha = 1) +
    scale_color_manual(values = colors) + scale_fill_manual(values = colors) +
    scale_x_continuous(limits = c(10, 60), breaks = seq(10, max(60,
        na.rm = TRUE), by = 10)) + scale_y_continuous(limits = c(7,
    20.5), breaks = seq(8, max(21, na.rm = TRUE), by = 4)) +
    theme_classic() + theme(legend.position = "none", axis.line = element_line(linewidth = 0.5,
    colour = "black"), axis.ticks = element_line(colour = "black"),
    axis.text = element_text(size = 14, colour = "black"), axis.title = element_text(size = 18,
        colour = "black"), plot.title = element_text(size = 0)) +
    labs(x = "Maternal median velocity", y = "Mean hourly velocity (offspring)")
plot_name
```

```
## `geom_smooth()` using formula = 'y ~ x'
```

```
## Warning: No shared levels found between `names(values)` of the manual scale and the
```

```
## data's fill values.
```

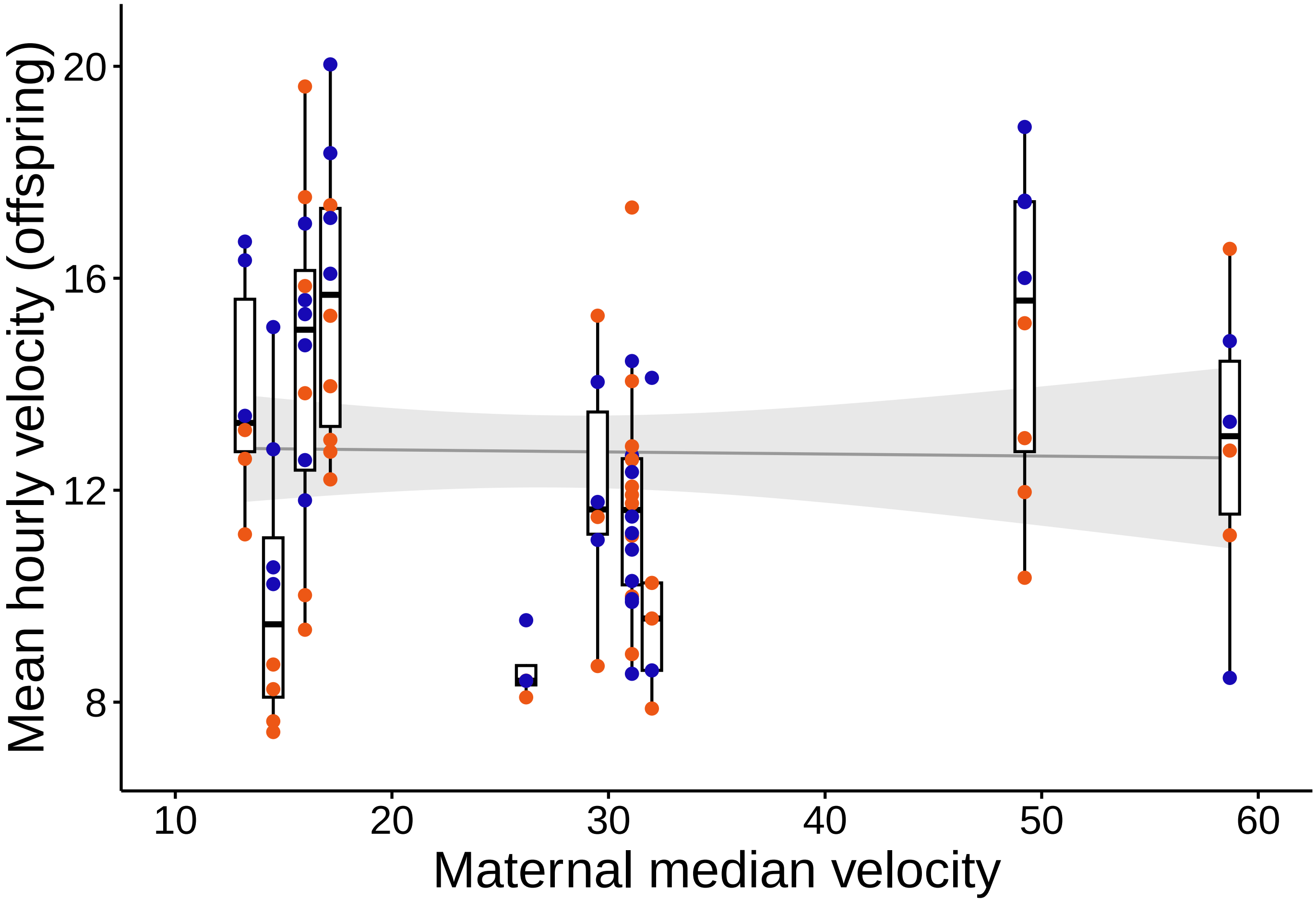


```r
ggsave("maternal_behavior_velocity.pdf", plot = plot_name, dpi = 600,
    width = 6, height = 6)
```

```
## `geom_smooth()` using formula = 'y ~ x'

## Warning: No shared levels found between `names(values)` of the manual scale and the
## data's fill values.
```

Plotting MomId by treatment

Now we will make a plot showing control by predator behavior, grouped by mother ID. This uses the full data set because we don't need mother behavior.
Each point on the plot will represent the mean values of all individuals who share a mother. Error bars will show the standard error of means for control (horizontal, blue) and predator (vertical, orange) offspring. The error bars are built from FishId means (not raw data) for each MomId.

```r
# Mean for each FishId
fish_means <- hourly_data %>%
    group_by(MomId, FishId, PredatorCue) %>%
    summarize(mean_MedianVel = mean(MedianVel_hourly_scaled,
        na.rm = TRUE), .groups = "drop")

# Mean and SE for MomId and treatment using FishId means
momid_by_treatment_data <- fish_means %>%
    group_by(MomId, PredatorCue) %>%
    summarize(mom_mean_MedianVel = mean(mean_MedianVel, na.rm = TRUE),
        se_MedianVel = sd(mean_MedianVel, na.rm = TRUE)/sqrt(n()),
        n_fish = n(), .groups = "drop")
```

```r
# Org into columns to plot
momid_by_treatment_data <- momid_by_treatment_data %>%
    pivot_wider(names_from = PredatorCue, values_from = c(mom_mean_MedianVel,
        se_MedianVel, n_fish), names_glue = "{.value}_{PredatorCue}") %>%
    rename(mean_control = mom_mean_MedianVel_0, se_control = se_MedianVel_0,
        n_fish_control = n_fish_0, mean_predator = mom_mean_MedianVel_1,
        se_predator = se_MedianVel_1, n_fish_predator = n_fish_1)

# Plot
colors <- c(`0` = "#1709b5", `1` = "#ed5715")
plot_name <- ggplot(momid_by_treatment_data, aes(x = mean_control,
    y = mean_predator)) + geom_errorbarh(aes(xmin = mean_control -
    se_control, xmax = mean_control + se_control), height = 0.05,
    size = 0.7, color = colors["0"], alpha = 1) + geom_errorbar(aes(ymin = mean_predator -
    se_predator, ymax = mean_predator + se_predator), width = 0.05,
    size = 0.7, color = colors["1"], alpha = 1) + geom_point(size = 2.5,
    shape = 21, color = "black", fill = "grey35", stroke = 1,
    alpha = 1) + xlim(-1, 1) + ylim(-1, 1) + coord_fixed(ratio = 1) +
    labs(x = "Control", y = "Predator") + theme_classic()
plot_name
```

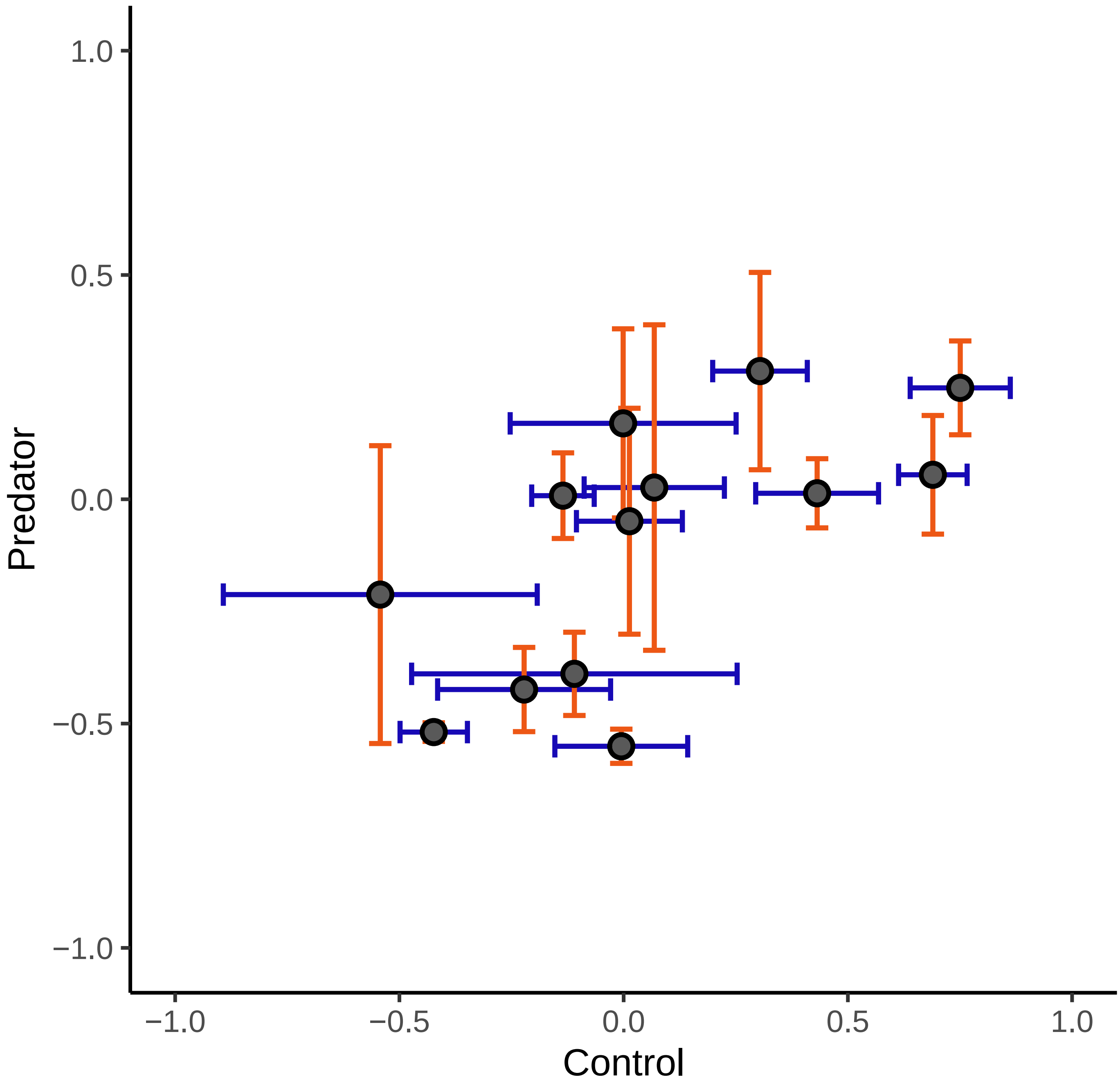


```
ggsave("maternal_effects_velocity.pdf", plot = plot_name, dpi = 600)
```

```
## Saving 6.5 x 4.5 in image
```

Testing for differences in individual variance between treatments over time

Now we will look at whether individual variance components differ by treatment using separate univariate models.

Prepare separate univariate control and predator models

Subset data by treatment for hourly:

```
control_data_hourly <- subset(hourly_data, PredatorCue == 0)
predator_data_hourly <- subset(hourly_data, PredatorCue == 1)
```

Set expanded priors for univariate models

```
# Prepare ExpDay to be a factor for heterogeneous residual
# variance
control_data_hourly$ExpDay_factor <- as.factor(control_data_hourly$ExpDay)
predator_data_hourly$ExpDay_factor <- as.factor(predator_data_hourly$ExpDay)

num_days <- nlevels(control_data_hourly$ExpDay_factor)
R_prior <- list(V = diag(num_days) * 0.5, nu = num_days + 0.002)

# Null
prior.null.univariate.idhExpDay <- list(R = R_prior)

# FishId
prior.fishId.univariate.idhExpDay <- list(R = R_prior, G = list(G1 = list(V = 1,
    nu = 1.002, alpha.mu = 0, alpha.V = 25^2)))

# MomId
prior.momId.univariate.idhExpDay <- list(R = R_prior, G = list(G1 = list(V = 1,
    nu = 1.002, alpha.mu = 0, alpha.V = 25^2)))

# FishId + MomId
prior.fishId.momId.univariate.idhExpDay <- list(R = R_prior,
    G = list(G1 = list(V = 1, nu = 1.002, alpha.mu = 0, alpha.V = 25^2),
        G2 = list(V = 1, nu = 1.002, alpha.mu = 0, alpha.V = 25^2)))

# FishId slopes
prior.fishId.slopes.univariate.idhExpDay <- list(R = R_prior,
    G = list(G1 = list(V = diag(2) * 0.5, nu = 2.002, alpha.mu = c(0,
        0), alpha.V = diag(2) * 25^2)))

# FishId slopes + MomId
prior.fishId.slopes.momId.univariate.idhExpDay <- list(R = R_prior,
    G = list(G1 = list(V = diag(2) * 0.5, nu = 2.002, alpha.mu = c(0,
        0), alpha.V = diag(2) * 25^2), G2 = list(V = 1, nu = 1.002,
        alpha.mu = 0, alpha.V = 25^2)))

# FishId slopes + MomId slopes
prior.fishId.slopes.momId.slopes.univariate.idhExpDay <- list(R = R_prior,
    G = list(G1 = list(V = diag(2) * 0.5, nu = 2.002, alpha.mu = c(0,
        0), alpha.V = diag(2) * 25^2), G2 = list(V = diag(2) *
        0.5, nu = 2.002, alpha.mu = c(0, 0), alpha.V = diag(2) *
        25^2)))

# FishId slopes + MomId - homogeneous residual variance
# (for confirmation)
prior.fishId.slopes.momId.univariate.homogemeous_residual <- list(R = list(V = 1,
    nu = 0.002), G = list(G1 = list(V = diag(2) * 0.5, nu = 2.002,
    alpha.mu = c(0, 0), alpha.V = diag(2) * 25^2), G2 = list(V = 1,
    nu = 1.002, alpha.mu = 0, alpha.V = 25^2)))
```

Model comparison to determine included random effects for CONTROL

```
# Null
model_control_hetero_residual_null <- readRDS("model_MedianVel_control_hetero_residual_null_8_20_25.rds")
# model_control_hetero_residual_null <-
# MCMCglmm(MedianVel_hourly_scaled ~ ExpDay + YearBatch +
# Hour, rcov = ~idh(ExpDay_factor):units, family =
# 'gaussian', data = control_data_hourly, prior =
# prior.null.univariate.idhExpDay, nitt = 200000, burnin =
```

```r
# 20000, thin = 100)
# saveRDS(model_control_hetero_residual_null, file =
# 'model_MedianVel_control_hetero_residual_null_8_20_25.rds')
summary(model_control_hetero_residual_null)
```

```
##
##  Iterations = 20001:199901
##  Thinning interval  = 100
##  Sample size  = 1800
##
##  DIC: 43446.35
##
##  R-structure:  ~idh(ExpDay_factor):units
##
##                       post.mean l-95% CI u-95% CI eff.samp
## ExpDay_factor0.units     3.2228   2.8876   3.6007     1545
## ExpDay_factor1.units     1.1199   0.9936   1.2475     1800
## ExpDay_factor2.units     0.7557   0.6736   0.8436     1800
## ExpDay_factor3.units     0.6212   0.5504   0.6974     1800
## ExpDay_factor4.units     0.5752   0.5129   0.6419     1800
## ExpDay_factor5.units     0.5737   0.5096   0.6431     1800
## ExpDay_factor6.units     0.9257   0.8180   1.0282     1800
## ExpDay_factor7.units     0.9774   0.8729   1.0906     1800
## ExpDay_factor8.units     0.9588   0.8508   1.0603     1800
## ExpDay_factor9.units     0.7562   0.6685   0.8403     2034
## ExpDay_factor10.units    0.7228   0.6372   0.8120     1800
## ExpDay_factor11.units    0.7070   0.6160   0.7849     1800
## ExpDay_factor12.units    0.8576   0.7610   0.9630     1947
## ExpDay_factor13.units    1.0353   0.9128   1.1480     1800
## ExpDay_factor14.units    1.1659   1.0422   1.3153     1800
## ExpDay_factor15.units    1.2435   1.1110   1.3772     1800
## ExpDay_factor16.units    0.8041   0.7119   0.8952     1800
## ExpDay_factor17.units    0.9354   0.8297   1.0429     2166
## ExpDay_factor18.units    0.8266   0.7398   0.9289     1660
## ExpDay_factor19.units    1.2264   1.0865   1.3668     1800
## ExpDay_factor20.units    0.9539   0.8356   1.0592     1800
## ExpDay_factor21.units    1.0024   0.8827   1.1141     1800
## ExpDay_factor22.units    1.0009   0.8882   1.1114     1800
## ExpDay_factor23.units    1.1806   1.0440   1.3141     1800
## ExpDay_factor24.units    0.8938   0.7968   0.9979     1800
## ExpDay_factor25.units    1.1472   1.0126   1.2754     1784
## ExpDay_factor26.units    0.9627   0.8535   1.0681     1800
## ExpDay_factor27.units    0.8647   0.7636   0.9650     1800
##
##  Location effects: MedianVel_hourly_scaled ~ ExpDay + YearBatch + Hour
##
##             post.mean  l-95% CI  u-95% CI eff.samp  pMCMC
## (Intercept)  0.424547  0.375925  0.475409     1800 <6e-04 ***
## ExpDay       0.003108  0.001148  0.004887     1953 <6e-04 ***
## YearBatch   -0.071294 -0.085974 -0.058889     1800 <6e-04 ***
## Hour        -0.042061 -0.046313 -0.037601     1800 <6e-04 ***
## ---
## Signif. codes:  0 '***' 0.001 '**' 0.01 '*' 0.05 '.' 0.1 ' ' 1
```

```r
# FishId
model_control_hetero_residual_id <- readRDS("model_MedianVel_control_hetero_residual_id_8_20_25.rds")
# model_control_hetero_residual_id <-
# MCMCglmm(MedianVel_hourly_scaled ~ ExpDay + YearBatch +
# Hour, random = ~FishId, rcov = ~idh(ExpDay_factor):units,
# family = 'gaussian', data = control_data_hourly, prior =
# prior.fishId.univariate.idhExpDay, nitt = 500000, burnin
```

```r
# = 50000, thin = 100)
# saveRDS(model_control_hetero_residual_id, file =
# 'model_MedianVel_control_hetero_residual_id_8_20_25.rds')
summary(model_control_hetero_residual_id)
```

```
##
##  Iterations = 50001:499901
##  Thinning interval  = 100
##  Sample size  = 4500
##
##  DIC: 40098.94
##
##  G-structure:  ~FishId
##
##        post.mean l-95% CI u-95% CI eff.samp
## FishId    0.1974   0.1228   0.2865     3287
##
##  R-structure:  ~idh(ExpDay_factor):units
##
##                        post.mean l-95% CI u-95% CI eff.samp
## ExpDay_factor0.units     2.9762   2.6457   3.3192     4500
## ExpDay_factor1.units     1.1199   0.9908   1.2415     4500
## ExpDay_factor2.units     0.7531   0.6729   0.8465     4500
## ExpDay_factor3.units     0.6232   0.5515   0.6954     4500
## ExpDay_factor4.units     0.5646   0.5027   0.6296     5715
## ExpDay_factor5.units     0.5404   0.4759   0.5983     4500
## ExpDay_factor6.units     0.9064   0.8051   1.0222     4500
## ExpDay_factor7.units     0.7730   0.6815   0.8601     4500
## ExpDay_factor8.units     0.8540   0.7559   0.9490     4201
## ExpDay_factor9.units     0.7351   0.6546   0.8234     4201
## ExpDay_factor10.units    0.6213   0.5441   0.6990     4091
## ExpDay_factor11.units    0.5275   0.4649   0.5903     4368
## ExpDay_factor12.units    0.6794   0.5949   0.7579     4500
## ExpDay_factor13.units    0.7922   0.7107   0.8875     4500
## ExpDay_factor14.units    0.8981   0.7970   1.0001     4500
## ExpDay_factor15.units    1.0724   0.9560   1.2037     4500
## ExpDay_factor16.units    0.5715   0.5067   0.6359     4500
## ExpDay_factor17.units    0.6866   0.6099   0.7675     4500
## ExpDay_factor18.units    0.5377   0.4720   0.5998     4500
## ExpDay_factor19.units    0.9034   0.7966   1.0077     4500
## ExpDay_factor20.units    0.6839   0.5997   0.7589     4500
## ExpDay_factor21.units    0.6958   0.6124   0.7750     4500
## ExpDay_factor22.units    0.6627   0.5866   0.7365     4500
## ExpDay_factor23.units    0.7768   0.6820   0.8640     4500
## ExpDay_factor24.units    0.6443   0.5713   0.7205     4500
## ExpDay_factor25.units    0.9104   0.8007   1.0160     4701
## ExpDay_factor26.units    0.7098   0.6283   0.7971     4500
## ExpDay_factor27.units    0.6266   0.5505   0.7005     4500
##
##  Location effects: MedianVel_hourly_scaled ~ ExpDay + YearBatch + Hour
##
##              post.mean   l-95% CI   u-95% CI eff.samp    pMCMC
## (Intercept)  0.5317214  0.2357674  0.8321399     4500 0.000444 ***
## ExpDay       0.0020744  0.0003034  0.0038891     4500 0.024889 *
## YearBatch   -0.1153168 -0.2290658 -0.0044104     4500 0.038667 *
## Hour        -0.0408700 -0.0448285 -0.0371669     4500  < 2e-04 ***
## ---
## Signif. codes:  0 '***' 0.001 '**' 0.01 '*' 0.05 '.' 0.1 ' ' 1
```

```r
# MomId
model_control_hetero_residual_mom <- readRDS("model_MedianVel_control_hetero_residual_mom_8_20_25.rds")
```

```r
# model_control_hetero_residual_mom <-
# MCMCglmm(MedianVel_hourly_scaled ~ ExpDay + YearBatch +
# Hour, random = ~MomId, rcov = ~idh(ExpDay_factor):units,
# family = 'gaussian', data = control_data_hourly, prior =
# prior.momId.univariate.idhExpDay, nitt = 300000, burnin =
# 30000, thin = 100)
# saveRDS(model_control_hetero_residual_mom, file =
# 'model_MedianVel_control_hetero_residual_mom_8_20_25.rds')
summary(model_control_hetero_residual_mom)
```

```
##
##  Iterations = 30001:299901
##  Thinning interval  = 100
##  Sample size  = 2700
##
##  DIC: 41129.64
##
##  G-structure:  ~MomId
##
##       post.mean l-95% CI u-95% CI eff.samp
## MomId    0.6091  0.06971    1.825      206
##
##  R-structure:  ~idh(ExpDay_factor):units
##
##                        post.mean l-95% CI u-95% CI eff.samp
## ExpDay_factor0.units     3.0195   2.6596   3.3631     2535
## ExpDay_factor1.units     1.1561   1.0336   1.2886     2700
## ExpDay_factor2.units     0.7862   0.7023   0.8837     2700
## ExpDay_factor3.units     0.6615   0.5895   0.7435     2700
## ExpDay_factor4.units     0.6082   0.5398   0.6790     2700
## ExpDay_factor5.units     0.5875   0.5238   0.6548     2700
## ExpDay_factor6.units     0.9847   0.8712   1.1019     2700
## ExpDay_factor7.units     0.8448   0.7498   0.9386     2535
## ExpDay_factor8.units     0.9070   0.8078   1.0169     2700
## ExpDay_factor9.units     0.7751   0.6918   0.8683     2897
## ExpDay_factor10.units    0.6374   0.5587   0.7189     2518
## ExpDay_factor11.units    0.5344   0.4736   0.5959     2700
## ExpDay_factor12.units    0.6692   0.5948   0.7496     2700
## ExpDay_factor13.units    0.7690   0.6883   0.8623     3069
## ExpDay_factor14.units    0.9441   0.8483   1.0606     2700
## ExpDay_factor15.units    1.1325   1.0134   1.2693     2700
## ExpDay_factor16.units    0.6293   0.5627   0.7045     2700
## ExpDay_factor17.units    0.7191   0.6392   0.8034     2700
## ExpDay_factor18.units    0.5951   0.5206   0.6635     2700
## ExpDay_factor19.units    0.9531   0.8461   1.0632     2700
## ExpDay_factor20.units    0.7233   0.6411   0.8062     2700
## ExpDay_factor21.units    0.7642   0.6780   0.8551     2700
## ExpDay_factor22.units    0.7571   0.6657   0.8396     2700
## ExpDay_factor23.units    0.8689   0.7664   0.9682     2700
## ExpDay_factor24.units    0.7385   0.6551   0.8224     2728
## ExpDay_factor25.units    1.0052   0.8847   1.1296     2411
## ExpDay_factor26.units    0.8126   0.7148   0.9076     2700
## ExpDay_factor27.units    0.7034   0.6211   0.7871     2700
##
##  Location effects: MedianVel_hourly_scaled ~ ExpDay + YearBatch + Hour
##
##             post.mean   l-95% CI   u-95% CI eff.samp   pMCMC
## (Intercept)  1.035e+00  1.052e-01  2.389e+00    205.0 0.00741 **
## ExpDay       1.715e-03 -4.276e-05  3.537e-03   2700.0 0.05926 .
## YearBatch   -3.642e-01 -9.290e-01  1.216e-02    215.2 0.02741 *
## Hour        -4.089e-02 -4.482e-02 -3.700e-02   2700.0 < 4e-04 ***
```

```
## ---
## Signif. codes:  0 '***' 0.001 '**' 0.01 '*' 0.05 '.' 0.1 ' ' 1
```

```r
# FishId + MomId
model_control_hetero_residual_id_mom <- readRDS("model_MedianVel_control_hetero_residual_id_mom_8_20_25.rds"
# model_control_hetero_residual_id_mom <-
# MCMCglmm(MedianVel_hourly_scaled ~ ExpDay + YearBatch +
# Hour, random = ~FishId + MomId, rcov =
# ~idh(ExpDay_factor):units, family = 'gaussian', data =
# control_data_hourly, prior =
# prior.fishId.momId.univariate.idhExpDay, nitt = 500000,
# burnin = 50000, thin = 100)
# saveRDS(model_control_hetero_residual_id_mom, file =
# 'model_MedianVel_control_hetero_residual_id_mom_8_20_25.rds')
summary(model_control_hetero_residual_id_mom)
```

```
##
##  Iterations = 50001:499901
##  Thinning interval  = 100
##  Sample size  = 4500
##
##  DIC: 40092.78
##
##  G-structure:  ~FishId
##
##        post.mean l-95% CI u-95% CI eff.samp
## FishId   0.07855  0.04458   0.1178     3770
##
##               ~MomId
##
##       post.mean l-95% CI u-95% CI eff.samp
## MomId    0.4312  0.03588     1.33    390.5
##
##  R-structure:  ~idh(ExpDay_factor):units
##
##                         post.mean l-95% CI u-95% CI eff.samp
## ExpDay_factor0.units       2.9463   2.6180   3.2885     4500
## ExpDay_factor1.units       1.1247   1.0033   1.2537     4500
## ExpDay_factor2.units       0.7522   0.6708   0.8455     4500
## ExpDay_factor3.units       0.6232   0.5468   0.6934     4500
## ExpDay_factor4.units       0.5663   0.5034   0.6329     5047
## ExpDay_factor5.units       0.5422   0.4805   0.6027     4500
## ExpDay_factor6.units       0.9088   0.8079   1.0224     5068
## ExpDay_factor7.units       0.7737   0.6870   0.8655     4500
## ExpDay_factor8.units       0.8546   0.7524   0.9515     4500
## ExpDay_factor9.units       0.7356   0.6497   0.8165     4500
## ExpDay_factor10.units      0.6205   0.5404   0.6951     4500
## ExpDay_factor11.units      0.5261   0.4630   0.5916     4500
## ExpDay_factor12.units      0.6756   0.5990   0.7623     4500
## ExpDay_factor13.units      0.7886   0.7044   0.8858     4450
## ExpDay_factor14.units      0.8983   0.7978   1.0047     4500
## ExpDay_factor15.units      1.0752   0.9589   1.2043     4803
## ExpDay_factor16.units      0.5707   0.5033   0.6391     4707
## ExpDay_factor17.units      0.6842   0.6087   0.7635     4500
## ExpDay_factor18.units      0.5355   0.4705   0.5977     4739
## ExpDay_factor19.units      0.9039   0.7989   1.0118     4500
## ExpDay_factor20.units      0.6827   0.6044   0.7626     4789
## ExpDay_factor21.units      0.6958   0.6134   0.7779     4500
## ExpDay_factor22.units      0.6636   0.5864   0.7382     4500
## ExpDay_factor23.units      0.7766   0.6849   0.8663     4500
## ExpDay_factor24.units      0.6462   0.5715   0.7214     4500
## ExpDay_factor25.units      0.9107   0.8043   1.0229     4444
```

```
## ExpDay_factor26.units    0.7110   0.6265   0.7942     4500
## ExpDay_factor27.units    0.6268   0.5548   0.7053     4500
##
##  Location effects: MedianVel_hourly_scaled ~ ExpDay + YearBatch + Hour
##
##              post.mean   l-95% CI   u-95% CI eff.samp   pMCMC
## (Intercept)  0.9743178  0.1635692  2.1047519    652.5 0.00356 **
## ExpDay       0.0020458  0.0002397  0.0037912   4500.0 0.02844 *
## YearBatch   -0.3317946 -0.7856415  0.0295036    529.4 0.02311 *
## Hour        -0.0408239 -0.0448704 -0.0370767   4500.0 < 2e-04 ***
## ---
## Signif. codes:  0 '***' 0.001 '**' 0.01 '*' 0.05 '.' 0.1 ' ' 1
```

```r
# FishId slopes
model_control_hetero_residual_id_slopes <- readRDS("model_MedianVel_control_hetero_residual_id_slopes_4_20_2
# model_control_hetero_residual_id_slopes <-
# MCMCglmm(MedianVel_hourly_scaled ~ ExpDay + YearBatch +
# Hour, random = ~us(1 + ExpDay):FishId, rcov =
# ~idh(ExpDay_factor):units, family = 'gaussian', data =
# control_data_hourly, prior =
# prior.fishId.slopes.univariate.idhExpDay, nitt = 500000,
# burnin = 50000, thin = 100)
# saveRDS(model_control_hetero_residual_id_slopes, file =
# 'model_MedianVel_control_hetero_residual_id_slopes_4_20_25.rds')
summary(model_control_hetero_residual_id_slopes)
```

```
##
##  Iterations = 50001:499901
##  Thinning interval  = 100
##  Sample size  = 4500
##
##  DIC: 38712.88
##
##  G-structure:  ~us(1 + ExpDay):FishId
##
##                               post.mean   l-95% CI  u-95% CI eff.samp
## (Intercept):(Intercept).FishId  0.218953  0.1301276  0.313759     3460
## ExpDay:(Intercept).FishId      -0.007809 -0.0129551 -0.002832     3819
## (Intercept):ExpDay.FishId      -0.007809 -0.0129551 -0.002832     3819
## ExpDay:ExpDay.FishId            0.001133  0.0006737  0.001603     2620
##
##  R-structure:  ~idh(ExpDay_factor):units
##
##                       post.mean l-95% CI u-95% CI eff.samp
## ExpDay_factor0.units     2.8090   2.4869   3.1483     4883
## ExpDay_factor1.units     1.0498   0.9364   1.1733     4956
## ExpDay_factor2.units     0.6939   0.6154   0.7758     4500
## ExpDay_factor3.units     0.5304   0.4693   0.5981     4500
## ExpDay_factor4.units     0.4675   0.4174   0.5250     4500
## ExpDay_factor5.units     0.4228   0.3743   0.4745     4500
## ExpDay_factor6.units     0.7543   0.6611   0.8433     4500
## ExpDay_factor7.units     0.7339   0.6570   0.8227     4500
## ExpDay_factor8.units     0.8211   0.7248   0.9125     4218
## ExpDay_factor9.units     0.6734   0.5995   0.7508     4500
## ExpDay_factor10.units    0.5968   0.5234   0.6739     5163
## ExpDay_factor11.units    0.5175   0.4585   0.5841     4500
## ExpDay_factor12.units    0.6901   0.6073   0.7726     4848
## ExpDay_factor13.units    0.8050   0.7145   0.8977     4500
## ExpDay_factor14.units    0.9165   0.8166   1.0299     4500
## ExpDay_factor15.units    1.0908   0.9703   1.2174     4500
## ExpDay_factor16.units    0.5816   0.5176   0.6480     4500
## ExpDay_factor17.units    0.6826   0.6018   0.7597     4154
```

```
## ExpDay_factor18.units    0.5084   0.4490   0.5674     4394
## ExpDay_factor19.units    0.8537   0.7625   0.9599     4500
## ExpDay_factor20.units    0.6413   0.5695   0.7188     4833
## ExpDay_factor21.units    0.6009   0.5302   0.6698     4500
## ExpDay_factor22.units    0.5324   0.4711   0.5930     4500
## ExpDay_factor23.units    0.6491   0.5758   0.7277     4500
## ExpDay_factor24.units    0.5167   0.4589   0.5794     4500
## ExpDay_factor25.units    0.8227   0.7275   0.9262     4490
## ExpDay_factor26.units    0.6321   0.5611   0.7049     4500
## ExpDay_factor27.units    0.5186   0.4586   0.5820     4287
##
##  Location effects: MedianVel_hourly_scaled ~ ExpDay + YearBatch + Hour
##
##             post.mean  l-95% CI  u-95% CI eff.samp  pMCMC
## (Intercept)  0.551461  0.285086  0.816814     4294 <2e-04 ***
## ExpDay       0.001827 -0.007568  0.011372     4500 0.6938
## YearBatch   -0.123742 -0.224135 -0.029343     4500 0.0156 *
## Hour        -0.040832 -0.044359 -0.037106     4500 <2e-04 ***
## ---
## Signif. codes:  0 '***' 0.001 '**' 0.01 '*' 0.05 '.' 0.1 ' ' 1
```

```r
# FishId slopes + MomId
model_control_hetero_residual_id_slopes_mom <- readRDS("model_MedianVel_control_hetero_residual_id_slopes_mo
# model_control_hetero_residual_id_slopes_mom <-
# MCMCglmm(MedianVel_hourly_scaled ~ ExpDay + YearBatch +
# Hour, random = ~us(1 + ExpDay):FishId + MomId, rcov =
# ~idh(ExpDay_factor):units, family = 'gaussian', data =
# control_data_hourly, prior =
# prior.fishId.slopes.momId.univariate.idhExpDay, nitt =
# 500000, burnin = 50000, thin = 100)
# saveRDS(model_control_hetero_residual_id_slopes_mom, file
# =
# 'model_MedianVel_control_hetero_residual_id_slopes_mom_include_hour_8_7_25.rds')
summary(model_control_hetero_residual_id_slopes_mom)  #
```

```
##
##  Iterations = 50001:499901
##  Thinning interval  = 100
##  Sample size  = 4500
##
##  DIC: 38709.59
##
##  G-structure:  ~us(1 + ExpDay):FishId
##
##                                post.mean   l-95% CI  u-95% CI eff.samp
## (Intercept):(Intercept).FishId  0.195115  0.1151073  0.287539     4557
## ExpDay:(Intercept).FishId      -0.010719 -0.0167673 -0.005360     4500
## (Intercept):ExpDay.FishId      -0.010719 -0.0167673 -0.005360     4500
## ExpDay:ExpDay.FishId            0.001096  0.0006716  0.001559     3557
##
##                ~MomId
##
##       post.mean l-95% CI u-95% CI eff.samp
## MomId    0.2703 0.004885   0.7757      246
##
##  R-structure:  ~idh(ExpDay_factor):units
##
##                         post.mean l-95% CI u-95% CI eff.samp
## ExpDay_factor0.units     2.7923   2.4465   3.0965     4500
## ExpDay_factor1.units     1.0504   0.9320   1.1696     4500
## ExpDay_factor2.units     0.6941   0.6158   0.7758     4500
## ExpDay_factor3.units     0.5324   0.4676   0.5995     4500
```

```
## ExpDay_factor4.units     0.4695   0.4143   0.5239     4500
## ExpDay_factor5.units     0.4233   0.3743   0.4725     5108
## ExpDay_factor6.units     0.7536   0.6651   0.8481     4500
## ExpDay_factor7.units     0.7337   0.6488   0.8141     4703
## ExpDay_factor8.units     0.8223   0.7281   0.9167     4500
## ExpDay_factor9.units     0.6725   0.5990   0.7515     4268
## ExpDay_factor10.units    0.5968   0.5222   0.6718     4500
## ExpDay_factor11.units    0.5155   0.4538   0.5746     4742
## ExpDay_factor12.units    0.6895   0.6081   0.7755     4997
## ExpDay_factor13.units    0.8024   0.7152   0.8942     4500
## ExpDay_factor14.units    0.9144   0.8145   1.0249     4500
## ExpDay_factor15.units    1.0924   0.9700   1.2151     4500
## ExpDay_factor16.units    0.5808   0.5140   0.6478     4500
## ExpDay_factor17.units    0.6822   0.6046   0.7617     4500
## ExpDay_factor18.units    0.5081   0.4516   0.5712     4500
## ExpDay_factor19.units    0.8542   0.7550   0.9526     4715
## ExpDay_factor20.units    0.6413   0.5650   0.7175     4809
## ExpDay_factor21.units    0.6008   0.5350   0.6722     4500
## ExpDay_factor22.units    0.5329   0.4764   0.5946     4500
## ExpDay_factor23.units    0.6501   0.5745   0.7256     4811
## ExpDay_factor24.units    0.5185   0.4587   0.5843     4786
## ExpDay_factor25.units    0.8227   0.7260   0.9247     4500
## ExpDay_factor26.units    0.6318   0.5595   0.7081     4500
## ExpDay_factor27.units    0.5177   0.4552   0.5831     4500
##
##  Location effects: MedianVel_hourly_scaled ~ ExpDay + YearBatch + Hour
##
##             post.mean  l-95% CI  u-95% CI eff.samp   pMCMC
## (Intercept)  0.833016  0.106937  1.705244    483.0 0.00489 **
## ExpDay       0.001534 -0.008077  0.010914   4500.0 0.75422
## YearBatch   -0.261408 -0.608734  0.031834    477.2 0.03022 *
## Hour        -0.040840 -0.044513 -0.037214   4500.0 < 2e-04 ***
## ---
## Signif. codes:  0 '***' 0.001 '**' 0.01 '*' 0.05 '.' 0.1 ' ' 1
```

```
# FishId slopes + MomId slopes
model_control_hetero_residual_id_slopes_mom_slopes <- readRDS("model_MedianVel_control_hetero_residual_id_sl
# model_control_hetero_residual_id_slopes_mom_slopes <-
# MCMCglmm(MedianVel_hourly_scaled ~ ExpDay + YearBatch +
# Hour, random = ~us(1 + ExpDay):FishId + us(1 +
# ExpDay):MomId, rcov = ~idh(ExpDay_factor):units, family =
# 'gaussian', data = control_data_hourly, prior =
# prior.fishId.slopes.momId.slopes.univariate.idhExpDay,
# nitt = 500000, burnin = 50000, thin = 100)
# saveRDS(model_control_hetero_residual_id_slopes_mom_slopes,
# file =
# 'model_MedianVel_control_hetero_residual_id_slopes_mom_slopes_8_20_25.rds')
summary(model_control_hetero_residual_id_slopes_mom_slopes)
```

```
##
##  Iterations = 50001:499901
##  Thinning interval  = 100
##  Sample size  = 4500
##
##  DIC: 38710.5
##
##  G-structure:  ~us(1 + ExpDay):FishId
##
##                                   post.mean   l-95% CI  u-95% CI eff.samp
## (Intercept):(Intercept).FishId  0.1854806  0.1066286  0.271636     4500
## ExpDay:(Intercept).FishId      -0.0087383 -0.0138691 -0.004346     4500
## (Intercept):ExpDay.FishId      -0.0087383 -0.0138691 -0.004346     4500
```

```
## ExpDay:ExpDay.FishId            0.0007836  0.0004404  0.001185     3734
##
##                ~us(1 + ExpDay):MomId
##
##                              post.mean   l-95% CI u-95% CI eff.samp
## (Intercept):(Intercept).MomId 0.1216257  1.230e-09 0.354947    499.4
## ExpDay:(Intercept).MomId      0.0009076 -6.146e-03 0.007055   3850.6
## (Intercept):ExpDay.MomId      0.0009076 -6.146e-03 0.007055   3850.6
## ExpDay:ExpDay.MomId           0.0006178  4.892e-08 0.001494   1319.7
##
##  R-structure:  ~idh(ExpDay_factor):units
##
##                       post.mean l-95% CI u-95% CI eff.samp
## ExpDay_factor0.units     2.7944   2.4766   3.1286     4500
## ExpDay_factor1.units     1.0496   0.9268   1.1669     4500
## ExpDay_factor2.units     0.6941   0.6151   0.7796     4500
## ExpDay_factor3.units     0.5314   0.4681   0.5955     4500
## ExpDay_factor4.units     0.4682   0.4158   0.5260     4500
## ExpDay_factor5.units     0.4228   0.3706   0.4724     4500
## ExpDay_factor6.units     0.7531   0.6661   0.8480     4500
## ExpDay_factor7.units     0.7333   0.6540   0.8239     4500
## ExpDay_factor8.units     0.8217   0.7284   0.9137     4500
## ExpDay_factor9.units     0.6728   0.5952   0.7478     4500
## ExpDay_factor10.units    0.5965   0.5248   0.6718     4500
## ExpDay_factor11.units    0.5165   0.4513   0.5767     4500
## ExpDay_factor12.units    0.6894   0.6114   0.7764     4500
## ExpDay_factor13.units    0.8020   0.7152   0.8976     4534
## ExpDay_factor14.units    0.9149   0.8135   1.0219     4500
## ExpDay_factor15.units    1.0905   0.9636   1.2146     4222
## ExpDay_factor16.units    0.5804   0.5166   0.6504     4812
## ExpDay_factor17.units    0.6813   0.6038   0.7602     4500
## ExpDay_factor18.units    0.5075   0.4531   0.5742     4867
## ExpDay_factor19.units    0.8543   0.7559   0.9519     4478
## ExpDay_factor20.units    0.6415   0.5681   0.7206     4500
## ExpDay_factor21.units    0.6007   0.5305   0.6686     4500
## ExpDay_factor22.units    0.5334   0.4727   0.5960     3760
## ExpDay_factor23.units    0.6496   0.5696   0.7228     4500
## ExpDay_factor24.units    0.5183   0.4608   0.5813     4500
## ExpDay_factor25.units    0.8264   0.7291   0.9269     4837
## ExpDay_factor26.units    0.6347   0.5560   0.7091     4500
## ExpDay_factor27.units    0.5183   0.4559   0.5812     4133
##
##  Location effects: MedianVel_hourly_scaled ~ ExpDay + YearBatch + Hour
##
##              post.mean   l-95% CI   u-95% CI eff.samp   pMCMC
## (Intercept)  0.7198891  0.2122607  1.3159729    862.3 0.00222 **
## ExpDay      -0.0003166 -0.0165406  0.0156189   4500.0 0.99111
## YearBatch   -0.2062084 -0.4431897 -0.0045960    646.4 0.01378 *
## Hour        -0.0408312 -0.0443411 -0.0371305   4500.0 < 2e-04 ***
## ---
## Signif. codes:  0 '***' 0.001 '**' 0.01 '*' 0.05 '.' 0.1 ' ' 1
```

```
# FishId slopes + MomId, but checking with homogeneous
# residual variance
model_control_homo_residual_id_slopes_mom <- readRDS("model_MedianVel_control_homo_residual_id_slopes_mom_8_
# model_control_homo_residual_id_slopes_mom <-
# MCMCglmm(MedianVel_hourly_scaled ~ ExpDay + YearBatch +
# Hour, random = ~us(1 + ExpDay):FishId + MomId, rcov =
# ~units, family = 'gaussian', data = control_data_hourly,
# prior =
# prior.fishId.slopes.momId.univariate.homogemeous_residual,
# nitt = 500000, burnin = 50000, thin = 100)
```

```r
# saveRDS(model_control_homo_residual_id_slopes_mom, file =
# 'model_MedianVel_control_homo_residual_id_slopes_mom_8_20_25.rds')
summary(model_control_homo_residual_id_slopes_mom)  # Worse fit than heterogeneous residual variance
```

```
##
##  Iterations = 50001:499901
##  Thinning interval  = 100
##  Sample size  = 4500
##
##  DIC: 39804.66
##
##  G-structure:  ~us(1 + ExpDay):FishId
##
##                                 post.mean   l-95% CI  u-95% CI eff.samp
## (Intercept):(Intercept).FishId  0.247162  0.1415812  0.368847     4500
## ExpDay:(Intercept).FishId      -0.013370 -0.0203330 -0.007221     4500
## (Intercept):ExpDay.FishId      -0.013370 -0.0203330 -0.007221     4500
## ExpDay:ExpDay.FishId            0.001172  0.0007118  0.001684     4201
##
##                ~MomId
##
##       post.mean l-95% CI u-95% CI eff.samp
## MomId     2.695   0.5789    5.756    342.2
##
##  R-structure:  ~units
##
##       post.mean l-95% CI u-95% CI eff.samp
## units    0.7576   0.7403   0.7746     4500
##
##  Location effects: MedianVel_hourly_scaled ~ ExpDay + YearBatch + Hour
##
##             post.mean  l-95% CI  u-95% CI eff.samp  pMCMC
## (Intercept)  2.871847  1.807219  4.098041   1793.5 <2e-04 ***
## ExpDay      -0.002204 -0.011557  0.007658   4174.9   0.62
## YearBatch   -1.138629 -1.475730 -0.810383    719.1 <2e-04 ***
## Hour        -0.044240 -0.048410 -0.040331   4500.0 <2e-04 ***
## ---
## Signif. codes:  0 '***' 0.001 '**' 0.01 '*' 0.05 '.' 0.1 ' ' 1
```

```r
# FishId + MomId + no YearBatch
model_control_hetero_residual_id_slopes_mom_noYearBatch <- readRDS("model_MedianVel_control_hetero_residual_
# model_control_hetero_residual_id_slopes_mom_noYearBatch
# <- MCMCglmm(MedianVel_hourly_scaled ~ ExpDay + Hour,
# random = ~us(1 + ExpDay):FishId + MomId, rcov =
# ~idh(ExpDay_factor):units, family = 'gaussian', data =
# control_data_hourly, prior =
# prior.fishId.slopes.momId.univariate.idhExpDay, nitt =
# 500000, burnin = 50000, thin = 100)
# saveRDS(model_control_hetero_residual_id_slopes_mom_noYearBatch,
# file =
# 'model_MedianVel_control_hetero_residual_id_slopes_mom_noYearBatch_8_20_25.rds')
summary(model_control_hetero_residual_id_slopes_mom_noYearBatch)  # Including YearBatch is better fit
```

```
##
##  Iterations = 50001:499901
##  Thinning interval  = 100
##  Sample size  = 4500
##
##  DIC: 38716.93
##
##  G-structure:  ~us(1 + ExpDay):FishId
##
```

```
##                                post.mean   l-95% CI  u-95% CI eff.samp
## (Intercept):(Intercept).FishId  0.195459  0.1151101  0.293839     4500
## ExpDay:(Intercept).FishId      -0.010691 -0.0166017 -0.005253     4500
## (Intercept):ExpDay.FishId      -0.010691 -0.0166017 -0.005253     4500
## ExpDay:ExpDay.FishId            0.001089  0.0006512  0.001538     4274
##
##                ~MomId
##
##       post.mean l-95% CI u-95% CI eff.samp
## MomId    0.1338 0.008126   0.3005     1165
##
##  R-structure:  ~idh(ExpDay_factor):units
##
##                         post.mean l-95% CI u-95% CI eff.samp
## ExpDay_factor0.units     2.8325   2.5245   3.1701     4199
## ExpDay_factor1.units     1.0513   0.9340   1.1742     4881
## ExpDay_factor2.units     0.6964   0.6154   0.7825     4500
## ExpDay_factor3.units     0.5338   0.4713   0.5973     4500
## ExpDay_factor4.units     0.4694   0.4162   0.5247     4500
## ExpDay_factor5.units     0.4244   0.3724   0.4725     4500
## ExpDay_factor6.units     0.7554   0.6650   0.8503     4500
## ExpDay_factor7.units     0.7352   0.6547   0.8225     4411
## ExpDay_factor8.units     0.8211   0.7277   0.9143     4500
## ExpDay_factor9.units     0.6714   0.5959   0.7475     4500
## ExpDay_factor10.units    0.5955   0.5221   0.6700     4868
## ExpDay_factor11.units    0.5148   0.4541   0.5781     4500
## ExpDay_factor12.units    0.6900   0.6113   0.7751     4500
## ExpDay_factor13.units    0.8015   0.7112   0.8899     3943
## ExpDay_factor14.units    0.9143   0.8148   1.0233     4500
## ExpDay_factor15.units    1.0898   0.9645   1.2099     4500
## ExpDay_factor16.units    0.5813   0.5182   0.6541     4500
## ExpDay_factor17.units    0.6816   0.6039   0.7613     4500
## ExpDay_factor18.units    0.5083   0.4481   0.5688     4391
## ExpDay_factor19.units    0.8543   0.7571   0.9538     4311
## ExpDay_factor20.units    0.6413   0.5685   0.7192     4500
## ExpDay_factor21.units    0.6012   0.5324   0.6734     4500
## ExpDay_factor22.units    0.5322   0.4716   0.5948     5011
## ExpDay_factor23.units    0.6498   0.5726   0.7267     5158
## ExpDay_factor24.units    0.5172   0.4577   0.5791     4500
## ExpDay_factor25.units    0.8237   0.7198   0.9260     5216
## ExpDay_factor26.units    0.6325   0.5624   0.7103     4785
## ExpDay_factor27.units    0.5177   0.4560   0.5849     5278
##
##  Location effects: MedianVel_hourly_scaled ~ ExpDay + Hour
##
##             post.mean  l-95% CI  u-95% CI eff.samp  pMCMC
## (Intercept)  0.242498  0.017968  0.490008     4226  0.048 *
## ExpDay       0.001460 -0.008471  0.010557     4500  0.744
## Hour        -0.040831 -0.044508 -0.037061     4500 <2e-04 ***
## ---
## Signif. codes:  0 '***' 0.001 '**' 0.01 '*' 0.05 '.' 0.1 ' ' 1
```

```
# FishId + MomId + no Hour
model_control_hetero_residual_id_slopes_mom_noHour <- readRDS("model_MedianVel_control_hetero_residual_id_sl
# model_control_hetero_residual_id_slopes_mom_noHour <-
# MCMCglmm(MedianVel_hourly_scaled ~ ExpDay + YearBatch,
# random = ~us(1 + ExpDay):FishId + MomId, rcov =
# ~idh(ExpDay_factor):units, family = 'gaussian', data =
# control_data_hourly, prior =
# prior.fishId.slopes.momId.univariate.idhExpDay, nitt =
# 500000, burnin = 50000, thin = 100)
# saveRDS(model_control_hetero_residual_id_slopes_mom_noHour,
```

```r
# file =
# 'model_MedianVel_control_hetero_residual_id_slopes_mom_noHour_8_20_25.rds')
summary(model_control_hetero_residual_id_slopes_mom_noHour)  # Including Hour is better fit
```

```
##
##  Iterations = 50001:499901
##  Thinning interval  = 100
##  Sample size  = 4500
##
##  DIC: 39162.96
##
##  G-structure:  ~us(1 + ExpDay):FishId
##
##                                post.mean   l-95% CI  u-95% CI eff.samp
## (Intercept):(Intercept).FishId  0.197377  0.1156082  0.294265     4273
## ExpDay:(Intercept).FishId      -0.010834 -0.0167238 -0.005435     4500
## (Intercept):ExpDay.FishId      -0.010834 -0.0167238 -0.005435     4500
## ExpDay:ExpDay.FishId            0.001094  0.0006943  0.001606     3595
##
##                ~MomId
##
##       post.mean  l-95% CI u-95% CI eff.samp
## MomId    0.2557 1.285e-06   0.7205    667.8
##
##  R-structure:  ~idh(ExpDay_factor):units
##
##                        post.mean l-95% CI u-95% CI eff.samp
## ExpDay_factor0.units     2.8971   2.5637   3.2380     4110
## ExpDay_factor1.units     1.1331   1.0020   1.2586     4500
## ExpDay_factor2.units     0.7408   0.6559   0.8249     4500
## ExpDay_factor3.units     0.5720   0.5029   0.6419     4500
## ExpDay_factor4.units     0.5055   0.4488   0.5660     4500
## ExpDay_factor5.units     0.4290   0.3788   0.4819     4500
## ExpDay_factor6.units     0.7716   0.6757   0.8617     4810
## ExpDay_factor7.units     0.7297   0.6452   0.8125     4500
## ExpDay_factor8.units     0.8440   0.7497   0.9422     4500
## ExpDay_factor9.units     0.7045   0.6234   0.7867     4241
## ExpDay_factor10.units    0.6107   0.5366   0.6895     4388
## ExpDay_factor11.units    0.5267   0.4618   0.5882     4500
## ExpDay_factor12.units    0.7001   0.6183   0.7823     4500
## ExpDay_factor13.units    0.8237   0.7253   0.9152     4303
## ExpDay_factor14.units    0.9216   0.8194   1.0349     4395
## ExpDay_factor15.units    1.1117   0.9931   1.2423     4500
## ExpDay_factor16.units    0.5965   0.5308   0.6636     4500
## ExpDay_factor17.units    0.6857   0.6108   0.7611     4500
## ExpDay_factor18.units    0.5200   0.4600   0.5834     4500
## ExpDay_factor19.units    0.8490   0.7560   0.9492     4500
## ExpDay_factor20.units    0.6572   0.5776   0.7331     4350
## ExpDay_factor21.units    0.6119   0.5386   0.6787     4272
## ExpDay_factor22.units    0.5440   0.4853   0.6098     4500
## ExpDay_factor23.units    0.6726   0.5929   0.7515     4877
## ExpDay_factor24.units    0.5389   0.4764   0.6050     4500
## ExpDay_factor25.units    0.8231   0.7268   0.9259     4500
## ExpDay_factor26.units    0.6558   0.5824   0.7381     4055
## ExpDay_factor27.units    0.5263   0.4638   0.5934     4941
##
##  Location effects: MedianVel_hourly_scaled ~ ExpDay + YearBatch
##
##             post.mean  l-95% CI  u-95% CI eff.samp  pMCMC
## (Intercept)  0.586336 -0.095881  1.444703     1294 0.0489 *
## ExpDay       0.001415 -0.007983  0.010685     4500 0.7649
```

```
## YearBatch   -0.250489 -0.589990  0.011017     1131 0.0284 *
## ---
## Signif. codes:  0 '***' 0.001 '**' 0.01 '*' 0.05 '.' 0.1 ' ' 1
```

```r
# Diagnostics on best fit model
autocorr.diag(model_control_hetero_residual_id_slopes_mom$Sol)
```

```
##           (Intercept)       ExpDay  YearBatch         Hour
## Lag 0      1.00000000  1.000000000 1.00000000  1.000000000
## Lag 100    0.23541757  0.003593538 0.29168977 -0.012621971
## Lag 500    0.22595256  0.007044282 0.26681988  0.007223131
## Lag 1000   0.15841771  0.001527958 0.17761030  0.012116271
## Lag 5000   0.01069454 -0.009907371 0.01228317 -0.001044382
```

```r
autocorr.diag(model_control_hetero_residual_id_slopes_mom$VCV)
```

```
##           (Intercept):(Intercept).FishId ExpDay:(Intercept).FishId
## Lag 0                         1.00000000              1.0000000000
## Lag 100                      -0.01586970              0.0007271021
## Lag 500                       0.01280544              0.0292582685
## Lag 1000                     -0.00172231              0.0065083926
## Lag 5000                      0.01198748              0.0096917009
##           (Intercept):ExpDay.FishId ExpDay:ExpDay.FishId        MomId
## Lag 0                  1.0000000000         1.0000000000 1.0000000000
## Lag 100                0.0007271021         0.0258623580 0.4622609276
## Lag 500                0.0292582685         0.0459919939 0.3708069173
## Lag 1000               0.0065083926        -0.0059889946 0.2915733330
## Lag 5000               0.0096917009        -0.0002376413 0.0002154738
##           ExpDay_factor0.units ExpDay_factor1.units ExpDay_factor2.units
## Lag 0              1.000000000          1.000000000          1.000000000
## Lag 100            0.001444368          0.015355693          0.013078661
## Lag 500            0.013198292         -0.003474485          0.007760885
## Lag 1000           0.005086230         -0.022727419          0.041374476
## Lag 5000           0.014696006          0.006892382         -0.016299105
##           ExpDay_factor3.units ExpDay_factor4.units ExpDay_factor5.units
## Lag 0              1.000000000          1.000000000          1.000000000
## Lag 100            0.004742796         -0.019600127         -0.025572777
## Lag 500           -0.001626216          0.006594685         -0.008075437
## Lag 1000           0.003515920          0.003179851          0.032410544
## Lag 5000          -0.009498159          0.004085722         -0.016695408
##           ExpDay_factor6.units ExpDay_factor7.units ExpDay_factor8.units
## Lag 0              1.000000000          1.000000000         1.0000000000
## Lag 100           -0.013981626         -0.022144110        -0.0137613409
## Lag 500           -0.006057313          0.021917737        -0.0034766142
## Lag 1000          -0.018606541          0.002186624         0.0008281552
## Lag 5000           0.013317659          0.011754862         0.0063448566
##           ExpDay_factor9.units ExpDay_factor10.units ExpDay_factor11.units
## Lag 0              1.000000000           1.000000000          1.0000000000
## Lag 100           -0.003384834           0.011174398         -0.0262873190
## Lag 500           -0.013771443           0.010974052         -0.0256736278
## Lag 1000           0.017850547           0.007699152          0.0006325215
## Lag 5000           0.026542964          -0.001704504          0.0357405909
##           ExpDay_factor12.units ExpDay_factor13.units ExpDay_factor14.units
## Lag 0               1.000000000            1.00000000           1.000000000
## Lag 100            -0.019064633           -0.01487488           0.011172481
## Lag 500             0.022832077            0.01544182           0.006711249
## Lag 1000           -0.002266196            0.02319849           0.022450223
## Lag 5000            0.007652511           -0.01388986           0.010639403
##           ExpDay_factor15.units ExpDay_factor16.units ExpDay_factor17.units
## Lag 0               1.000000000           1.000000000           1.000000000
## Lag 100             0.020071741           0.006138043           0.011563456
## Lag 500            -0.016277234          -0.031227950          -0.003264776
## Lag 1000            0.003258968           0.020766564          -0.005275780
```

```
## Lag 5000          -0.015555722          0.004145090          0.001493945
##          ExpDay_factor18.units ExpDay_factor19.units ExpDay_factor20.units
## Lag 0               1.000000000           1.000000000           1.000000000
## Lag 100             0.008083084          -0.023466340           0.004254723
## Lag 500             0.004873262           0.008452796          -0.011402065
## Lag 1000           -0.010844302           0.022997451          -0.013018523
## Lag 5000            0.011847928           0.006404397          -0.010202462
##          ExpDay_factor21.units ExpDay_factor22.units ExpDay_factor23.units
## Lag 0               1.000000000           1.000000000          1.0000000000
## Lag 100             0.002667499          -0.017118729          0.0004158886
## Lag 500             0.008124462           0.007573513         -0.0174372590
## Lag 1000           -0.025216456           0.007748959         -0.0234110128
## Lag 5000           -0.020420919          -0.015915456         -0.0042331163
##          ExpDay_factor24.units ExpDay_factor25.units ExpDay_factor26.units
## Lag 0               1.000000000          1.0000000000           1.000000000
## Lag 100            -0.030954594         -0.0106168640           0.016305448
## Lag 500             0.012765073         -0.0001157736           0.007422267
## Lag 1000           -0.022834308         -0.0008901515          -0.011394059
## Lag 5000           -0.002530424         -0.0001151175           0.006561504
##          ExpDay_factor27.units
## Lag 0               1.000000000
## Lag 100             0.002237151
## Lag 500            -0.027068843
## Lag 1000            0.010714622
## Lag 5000            0.004401828
```

```
effectiveSize(model_control_hetero_residual_id_slopes_mom$Sol)
```

```
## (Intercept)      ExpDay   YearBatch        Hour
##    483.0311   4500.0000    477.2259   4500.0000
```

```
effectiveSize(model_control_hetero_residual_id_slopes_mom$VCV)  # Numbers are a bit low, so will try the mo
```

```
## (Intercept):(Intercept).FishId      ExpDay:(Intercept).FishId
##                      4557.1371                      4500.0000
##      (Intercept):ExpDay.FishId           ExpDay:ExpDay.FishId
##                      4500.0000                      3557.1411
##                          MomId          ExpDay_factor0.units
##                       246.0282                      4500.0000
##           ExpDay_factor1.units           ExpDay_factor2.units
##                      4500.0000                      4500.0000
##           ExpDay_factor3.units           ExpDay_factor4.units
##                      4500.0000                      4500.0000
##           ExpDay_factor5.units           ExpDay_factor6.units
##                      5108.1387                      4500.0000
##           ExpDay_factor7.units           ExpDay_factor8.units
##                      4702.7647                      4500.0000
##           ExpDay_factor9.units          ExpDay_factor10.units
##                      4267.8170                      4500.0000
##          ExpDay_factor11.units          ExpDay_factor12.units
##                      4741.9188                      4996.9041
##          ExpDay_factor13.units          ExpDay_factor14.units
##                      4500.0000                      4500.0000
##          ExpDay_factor15.units          ExpDay_factor16.units
##                      4500.0000                      4500.0000
##          ExpDay_factor17.units          ExpDay_factor18.units
##                      4500.0000                      4500.0000
##          ExpDay_factor19.units          ExpDay_factor20.units
##                      4715.2239                      4808.6993
##          ExpDay_factor21.units          ExpDay_factor22.units
##                      4500.0000                      4500.0000
##          ExpDay_factor23.units          ExpDay_factor24.units
##                      4811.1932                      4786.4264
```

```
##          ExpDay_factor25.units          ExpDay_factor26.units
##                      4500.0000                      4500.0000
##          ExpDay_factor27.units
##                      4500.0000
```

```
# FishId slopes + MomId with more iterations
model_control_hetero_residual_id_slopes_mom_more_iterations <- readRDS("model_MedianVel_control_hetero_resic
# model_control_hetero_residual_id_slopes_mom_more_iterations
# <- MCMCglmm(MedianVel_hourly_scaled ~ ExpDay + YearBatch
# + Hour, random = ~us(1 + ExpDay):FishId + MomId, rcov =
# ~idh(ExpDay_factor):units, family = 'gaussian', data =
# control_data_hourly, prior =
# prior.fishId.slopes.momId.univariate.idhExpDay, nitt =
# 1500000, burnin = 150000, thin = 200)
# saveRDS(model_control_hetero_residual_id_slopes_mom_more_iterations,
# file =
# 'model_MedianVel_control_hetero_residual_id_slopes_mom_8_20_25_more_iterations.rds')
summary(model_control_hetero_residual_id_slopes_mom_more_iterations)  #
```

```
##
##  Iterations = 150001:1499801
##  Thinning interval  = 200
##  Sample size  = 6750
##
##  DIC: 38709.8
##
##  G-structure:  ~us(1 + ExpDay):FishId
##
##                                post.mean   l-95% CI  u-95% CI eff.samp
## (Intercept):(Intercept).FishId  0.194883  0.1124122  0.288182     6750
## ExpDay:(Intercept).FishId      -0.010668 -0.0166100 -0.005509     6279
## (Intercept):ExpDay.FishId      -0.010668 -0.0166100 -0.005509     6279
## ExpDay:ExpDay.FishId            0.001086  0.0006536  0.001536     5926
##
##                ~MomId
##
##       post.mean  l-95% CI u-95% CI eff.samp
## MomId    0.2611 4.738e-05   0.7585     1261
##
##  R-structure:  ~idh(ExpDay_factor):units
##
##                         post.mean l-95% CI u-95% CI eff.samp
## ExpDay_factor0.units       2.7933   2.4599   3.1142     6750
## ExpDay_factor1.units       1.0520   0.9395   1.1828     6750
## ExpDay_factor2.units       0.6947   0.6129   0.7744     6750
## ExpDay_factor3.units       0.5330   0.4679   0.5955     6750
## ExpDay_factor4.units       0.4683   0.4125   0.5226     6750
## ExpDay_factor5.units       0.4237   0.3771   0.4755     6750
## ExpDay_factor6.units       0.7550   0.6633   0.8466     6750
## ExpDay_factor7.units       0.7335   0.6527   0.8185     7061
## ExpDay_factor8.units       0.8208   0.7263   0.9115     6750
## ExpDay_factor9.units       0.6738   0.5997   0.7527     6750
## ExpDay_factor10.units      0.5963   0.5162   0.6671     6750
## ExpDay_factor11.units      0.5157   0.4572   0.5787     6750
## ExpDay_factor12.units      0.6891   0.6079   0.7694     7031
## ExpDay_factor13.units      0.8020   0.7113   0.8920     6750
## ExpDay_factor14.units      0.9154   0.8131   1.0213     6750
## ExpDay_factor15.units      1.0896   0.9676   1.2110     6750
## ExpDay_factor16.units      0.5814   0.5161   0.6510     6365
## ExpDay_factor17.units      0.6818   0.6040   0.7570     6750
## ExpDay_factor18.units      0.5088   0.4509   0.5701     7269
## ExpDay_factor19.units      0.8542   0.7614   0.9586     7062
```

```
## ExpDay_factor20.units    0.6409   0.5630   0.7112     6750
## ExpDay_factor21.units    0.6011   0.5343   0.6722     6750
## ExpDay_factor22.units    0.5324   0.4744   0.5968     7030
## ExpDay_factor23.units    0.6495   0.5735   0.7277     6750
## ExpDay_factor24.units    0.5167   0.4538   0.5770     7028
## ExpDay_factor25.units    0.8240   0.7258   0.9268     6750
## ExpDay_factor26.units    0.6324   0.5615   0.7105     7982
## ExpDay_factor27.units    0.5175   0.4573   0.5835     6750
##
##  Location effects: MedianVel_hourly_scaled ~ ExpDay + YearBatch + Hour
##
##              post.mean  l-95% CI  u-95% CI eff.samp   pMCMC
## (Intercept)  0.823241  0.191468  1.654387     1808 0.00267 **
## ExpDay       0.001726 -0.007231  0.011429     6750 0.73719
## YearBatch   -0.260724 -0.607365  0.006678     1467 0.02133 *
## Hour        -0.040851 -0.044525 -0.037158     6750 < 1e-04 ***
## ---
## Signif. codes:  0 '***' 0.001 '**' 0.01 '*' 0.05 '.' 0.1 ' ' 1
```

```r
# Diagnostics on best fit model
autocorr.diag(model_control_hetero_residual_id_slopes_mom_more_iterations$Sol)
```

```
##            (Intercept)       ExpDay  YearBatch         Hour
## Lag 0       1.00000000  1.000000000 1.00000000  1.000000000
## Lag 200     0.17111949 -0.005612968 0.21505421 -0.010928305
## Lag 1000    0.07524692  0.010497941 0.10004282 -0.015296638
## Lag 2000    0.04075424 -0.015343298 0.05099714  0.024192846
## Lag 10000   0.02446221 -0.007308261 0.01877807 -0.005356548
```

```r
autocorr.diag(model_control_hetero_residual_id_slopes_mom_more_iterations$VCV)
```

```
##            (Intercept):(Intercept).FishId ExpDay:(Intercept).FishId
## Lag 0                         1.000000000               1.000000000
## Lag 200                      -0.002032583              -0.006889463
## Lag 1000                      0.017474298               0.009204490
## Lag 2000                     -0.020867735              -0.021393998
## Lag 10000                    -0.002367352              -0.009572682
##            (Intercept):ExpDay.FishId ExpDay:ExpDay.FishId      MomId
## Lag 0                    1.000000000          1.000000000 1.00000000
## Lag 200                 -0.006889463          0.006781000 0.34956751
## Lag 1000                 0.009204490          0.014091669 0.17425189
## Lag 2000                -0.021393998         -0.020467144 0.09197195
## Lag 10000               -0.009572682         -0.002984191 0.01106186
##            ExpDay_factor0.units ExpDay_factor1.units ExpDay_factor2.units
## Lag 0               1.000000000         1.0000000000          1.000000000
## Lag 200             0.009061296         0.0031927090          0.016235279
## Lag 1000           -0.008355536        -0.0022312946         -0.003163191
## Lag 2000            0.026690122        -0.0004698195         -0.026735497
## Lag 10000          -0.001037203         0.0052307352          0.028157630
##            ExpDay_factor3.units ExpDay_factor4.units ExpDay_factor5.units
## Lag 0               1.000000000          1.000000000          1.000000000
## Lag 200             0.005666972         -0.002227192          0.004307085
## Lag 1000           -0.002202932         -0.008193844          0.006143137
## Lag 2000           -0.006485410         -0.023243045         -0.003075200
## Lag 10000           0.004996769         -0.014931200          0.001425085
##            ExpDay_factor6.units ExpDay_factor7.units ExpDay_factor8.units
## Lag 0               1.000000000          1.000000000          1.000000000
## Lag 200            -0.011283071         -0.022561346          0.014900143
## Lag 1000           -0.007663699         -0.003869795          0.014786516
## Lag 2000           -0.029708164          0.013118851          0.003915998
## Lag 10000          -0.028866242          0.007321321         -0.021308911
##            ExpDay_factor9.units ExpDay_factor10.units ExpDay_factor11.units
## Lag 0              1.0000000000           1.000000000           1.000000000
```

```
## Lag 200          -0.0024381771         -0.010058622          0.003054275
## Lag 1000          0.0045057849          0.003734751         -0.005518727
## Lag 2000         -0.0004265589          0.016479380         -0.005142135
## Lag 10000        -0.0066480862         -0.009276831          0.007967966
##             ExpDay_factor12.units ExpDay_factor13.units ExpDay_factor14.units
## Lag 0                 1.000000000           1.000000000           1.000000000
## Lag 200               0.004349099           0.011505625           0.003300545
## Lag 1000              0.012674563          -0.008497305          -0.002786231
## Lag 2000             -0.002329151           0.002570911          -0.004885427
## Lag 10000            -0.027599877          -0.002001050          -0.002237110
##             ExpDay_factor15.units ExpDay_factor16.units ExpDay_factor17.units
## Lag 0                1.0000000000           1.000000000           1.000000000
## Lag 200             -0.0038494187           0.003681212           0.002696878
## Lag 1000            -0.0088120234          -0.009583044          -0.002225762
## Lag 2000            -0.0108083343           0.004772028           0.001342437
## Lag 10000            0.0005071142           0.002473124          -0.004347789
##             ExpDay_factor18.units ExpDay_factor19.units ExpDay_factor20.units
## Lag 0                 1.000000000           1.000000000           1.000000000
## Lag 200              -0.014474181          -0.022638421          -0.002445865
## Lag 1000              0.004572459          -0.002440046          -0.006485586
## Lag 2000             -0.014267643          -0.001393329          -0.007217407
## Lag 10000            -0.004420129           0.003913137          -0.015703449
##             ExpDay_factor21.units ExpDay_factor22.units ExpDay_factor23.units
## Lag 0                1.0000000000           1.000000000          1.0000000000
## Lag 200             -0.0015784464           0.012296382          0.0098328352
## Lag 1000             0.0004168082          -0.002032284          0.0068702616
## Lag 2000            -0.0052590794          -0.022714629          0.0007375418
## Lag 10000            0.0071956377           0.012553545          0.0070762716
##             ExpDay_factor24.units ExpDay_factor25.units ExpDay_factor26.units
## Lag 0                 1.000000000           1.000000000          1.000000e+00
## Lag 200               0.005234604          -0.010147813         -1.994093e-02
## Lag 1000              0.016027687          -0.003061064         -9.911822e-05
## Lag 2000             -0.003447003           0.004008703          2.592222e-02
## Lag 10000             0.009299453          -0.006386869         -1.653290e-02
##             ExpDay_factor27.units
## Lag 0                 1.000000000
## Lag 200              -0.005611392
## Lag 1000              0.008375930
## Lag 2000             -0.008941265
## Lag 10000            -0.005353049
```

```
effectiveSize(model_control_hetero_residual_id_slopes_mom_more_iterations$Sol)
```

```
## (Intercept)      ExpDay   YearBatch        Hour
##    1808.115    6750.000    1466.680    6750.000
```

```
effectiveSize(model_control_hetero_residual_id_slopes_mom_more_iterations$VCV)
```

```
## (Intercept):(Intercept).FishId      ExpDay:(Intercept).FishId
##                       6750.000                       6278.727
##      (Intercept):ExpDay.FishId           ExpDay:ExpDay.FishId
##                       6278.727                       5925.560
##                          MomId          ExpDay_factor0.units
##                       1260.712                       6750.000
##           ExpDay_factor1.units           ExpDay_factor2.units
##                       6750.000                       6750.000
##           ExpDay_factor3.units           ExpDay_factor4.units
##                       6750.000                       6750.000
##           ExpDay_factor5.units           ExpDay_factor6.units
##                       6750.000                       6750.000
##           ExpDay_factor7.units           ExpDay_factor8.units
##                       7060.562                       6750.000
##           ExpDay_factor9.units          ExpDay_factor10.units
```

```
##                     6750.000                         6750.000
##          ExpDay_factor11.units         ExpDay_factor12.units
##                     6750.000                         7031.094
##          ExpDay_factor13.units         ExpDay_factor14.units
##                     6750.000                         6750.000
##          ExpDay_factor15.units         ExpDay_factor16.units
##                     6750.000                         6365.298
##          ExpDay_factor17.units         ExpDay_factor18.units
##                     6750.000                         7269.051
##          ExpDay_factor19.units         ExpDay_factor20.units
##                     7061.651                         6750.000
##          ExpDay_factor21.units         ExpDay_factor22.units
##                     6750.000                         7030.014
##          ExpDay_factor23.units         ExpDay_factor24.units
##                     6750.000                         7028.060
##          ExpDay_factor25.units         ExpDay_factor26.units
##                     6750.000                         7981.845
##          ExpDay_factor27.units
##                     6750.000
```

Model comparison to determine included random effects for PREDATOR

```
# Null
model_predator_hetero_residual_null <- readRDS("model_MedianVel_predator_hetero_residual_null_8_21_25.rds")
# model_predator_hetero_residual_null <-
# MCMCglmm(MedianVel_hourly_scaled ~ ExpDay + YearBatch +
# Hour, rcov = ~idh(ExpDay_factor):units, family =
# 'gaussian', data = predator_data_hourly, prior =
# prior.null.univariate.idhExpDay, nitt = 500000, burnin =
# 50000, thin = 100)
# saveRDS(model_predator_hetero_residual_null, file =
# 'model_MedianVel_predator_hetero_residual_null_8_21_25.rds')
summary(model_predator_hetero_residual_null)
```

```
##
##  Iterations = 50001:499901
##  Thinning interval  = 100
##  Sample size  = 4500
##
##  DIC: 47009.55
##
##  R-structure:  ~idh(ExpDay_factor):units
##
##                      post.mean l-95% CI u-95% CI eff.samp
## ExpDay_factor0.units     1.5874   1.4179   1.7616     4500
## ExpDay_factor1.units     1.1046   0.9833   1.2195     4500
## ExpDay_factor2.units     0.7126   0.6349   0.7934     4500
## ExpDay_factor3.units     0.8241   0.7307   0.9114     4500
## ExpDay_factor4.units     1.0247   0.9129   1.1376     4639
## ExpDay_factor5.units     1.0754   0.9585   1.1908     4269
## ExpDay_factor6.units     0.9326   0.8340   1.0429     4500
## ExpDay_factor7.units     0.6883   0.6159   0.7622     4188
## ExpDay_factor8.units     0.7886   0.7050   0.8749     4500
## ExpDay_factor9.units     0.9641   0.8583   1.0633     4254
## ExpDay_factor10.units    0.9965   0.8945   1.1094     4500
## ExpDay_factor11.units    0.7903   0.7075   0.8813     5863
## ExpDay_factor12.units    0.8988   0.8037   0.9952     4500
## ExpDay_factor13.units    0.8915   0.8021   0.9957     4500
## ExpDay_factor14.units    0.8359   0.7449   0.9206     4500
## ExpDay_factor15.units    0.8813   0.7869   0.9774     4500
## ExpDay_factor16.units    0.7946   0.7124   0.8883     4500
```

```
## ExpDay_factor17.units    0.7610   0.6830   0.8537     4500
## ExpDay_factor18.units    0.7928   0.7048   0.8819     4500
## ExpDay_factor19.units    0.9995   0.8939   1.1083     4500
## ExpDay_factor20.units    0.8528   0.7617   0.9502     4500
## ExpDay_factor21.units    0.9011   0.8098   1.0095     4500
## ExpDay_factor22.units    1.1127   0.9908   1.2321     4500
## ExpDay_factor23.units    1.0146   0.9111   1.1294     4500
## ExpDay_factor24.units    0.7849   0.7023   0.8750     4500
## ExpDay_factor25.units    0.8520   0.7594   0.9482     4500
## ExpDay_factor26.units    0.8752   0.7769   0.9708     4500
## ExpDay_factor27.units    0.7561   0.6753   0.8428     4500
##
##  Location effects: MedianVel_hourly_scaled ~ ExpDay + YearBatch + Hour
##
##             post.mean l-95% CI u-95% CI eff.samp  pMCMC
## (Intercept)  -0.11968 -0.16680 -0.07211     4500 <2e-04 ***
## ExpDay        0.00375  0.00180  0.00547     4821 <2e-04 ***
## YearBatch     0.04922  0.03656  0.06302     4221 <2e-04 ***
## Hour         -0.02556 -0.02956 -0.02136     4500 <2e-04 ***
## ---
## Signif. codes:  0 '***' 0.001 '**' 0.01 '*' 0.05 '.' 0.1 ' ' 1
```

```
# FishId
model_predator_hetero_residual_id <- readRDS("model_MedianVel_predator_hetero_residual_id_8_21_25.rds")
# model_predator_hetero_residual_id <-
# MCMCglmm(MedianVel_hourly_scaled ~ ExpDay + YearBatch +
# Hour, random = ~FishId, rcov = ~idh(ExpDay_factor):units,
# family = 'gaussian', data = predator_data_hourly, prior =
# prior.fishId.univariate.idhExpDay, nitt = 500000, burnin
# = 50000, thin = 100)
# saveRDS(model_predator_hetero_residual_id, file =
# 'model_MedianVel_predator_hetero_residual_id_8_21_25.rds')
summary(model_predator_hetero_residual_id)
```

```
##
##  Iterations = 50001:499901
##  Thinning interval  = 100
##  Sample size  = 4500
##
##  DIC: 43773.77
##
##  G-structure:  ~FishId
##
##        post.mean l-95% CI u-95% CI eff.samp
## FishId    0.1902   0.1228   0.2726     3924
##
##  R-structure:  ~idh(ExpDay_factor):units
##
##                       post.mean l-95% CI u-95% CI eff.samp
## ExpDay_factor0.units     1.6066   1.4362   1.7961     4500
## ExpDay_factor1.units     1.2288   1.1041   1.3687     4500
## ExpDay_factor2.units     0.8130   0.7238   0.9064     4500
## ExpDay_factor3.units     1.0130   0.9015   1.1253     4500
## ExpDay_factor4.units     1.1074   0.9863   1.2338     4500
## ExpDay_factor5.units     0.9265   0.8275   1.0346     4500
## ExpDay_factor6.units     0.7174   0.6329   0.7963     4500
## ExpDay_factor7.units     0.6269   0.5617   0.6989     4500
## ExpDay_factor8.units     0.7242   0.6465   0.8083     4500
## ExpDay_factor9.units     0.7731   0.6910   0.8607     4326
## ExpDay_factor10.units    0.7518   0.6689   0.8344     4500
## ExpDay_factor11.units    0.4865   0.4338   0.5422     4864
## ExpDay_factor12.units    0.5926   0.5284   0.6567     4500
```

```
## ExpDay_factor13.units    0.6578   0.5879   0.7325     4500
## ExpDay_factor14.units    0.5928   0.5255   0.6560     4500
## ExpDay_factor15.units    0.6680   0.5953   0.7395     4500
## ExpDay_factor16.units    0.5860   0.5227   0.6494     4500
## ExpDay_factor17.units    0.6000   0.5355   0.6672     4500
## ExpDay_factor18.units    0.5733   0.5125   0.6372     4500
## ExpDay_factor19.units    0.7465   0.6646   0.8309     4500
## ExpDay_factor20.units    0.6661   0.5949   0.7406     4500
## ExpDay_factor21.units    0.6835   0.6094   0.7620     4500
## ExpDay_factor22.units    0.8605   0.7718   0.9658     4500
## ExpDay_factor23.units    0.8029   0.7204   0.8933     4500
## ExpDay_factor24.units    0.6399   0.5692   0.7080     4500
## ExpDay_factor25.units    0.7168   0.6385   0.7976     4500
## ExpDay_factor26.units    0.7576   0.6766   0.8462     5237
## ExpDay_factor27.units    0.6476   0.5751   0.7197     4693
##
##  Location effects: MedianVel_hourly_scaled ~ ExpDay + YearBatch + Hour
##
##              post.mean   l-95% CI   u-95% CI eff.samp  pMCMC
## (Intercept)  0.0053669 -0.2862076  0.2801941     4500  0.985
## ExpDay       0.0019948  0.0002191  0.0037048     4730  0.028 *
## YearBatch    0.0163024 -0.0933694  0.1186114     4500  0.741
## Hour        -0.0239531 -0.0277254 -0.0203574     4500 <2e-04 ***
## ---
## Signif. codes:  0 '***' 0.001 '**' 0.01 '*' 0.05 '.' 0.1 ' ' 1
```

```
# MomId
model_predator_hetero_residual_mom <- readRDS("model_MedianVel_predator_hetero_residual_mom_8_21_25.rds")
# model_predator_hetero_residual_mom <-
# MCMCglmm(MedianVel_hourly_scaled ~ ExpDay + YearBatch +
# Hour, random = ~MomId, rcov = ~idh(ExpDay_factor):units,
# family = 'gaussian', data = predator_data_hourly, prior =
# prior.momId.univariate.idhExpDay, nitt = 500000, burnin =
# 50000, thin = 100)
# saveRDS(model_predator_hetero_residual_mom, file =
# 'model_MedianVel_predator_hetero_residual_mom_8_21_25.rds')
summary(model_predator_hetero_residual_mom)
```

```
##
##  Iterations = 50001:499901
##  Thinning interval  = 100
##  Sample size  = 4500
##
##  DIC: 45462.71
##
##  G-structure:  ~MomId
##
##       post.mean l-95% CI u-95% CI eff.samp
## MomId    0.1204  0.03315   0.2537     1576
##
##  R-structure:  ~idh(ExpDay_factor):units
##
##                       post.mean l-95% CI u-95% CI eff.samp
## ExpDay_factor0.units     1.6254   1.4570   1.8067     4500
## ExpDay_factor1.units     1.2102   1.0801   1.3435     4500
## ExpDay_factor2.units     0.7360   0.6588   0.8243     6262
## ExpDay_factor3.units     0.9382   0.8345   1.0448     4500
## ExpDay_factor4.units     1.0861   0.9712   1.2131     4500
## ExpDay_factor5.units     0.9778   0.8715   1.0870     4500
## ExpDay_factor6.units     0.7799   0.6945   0.8666     4500
## ExpDay_factor7.units     0.6922   0.6184   0.7728     4500
## ExpDay_factor8.units     0.7809   0.6966   0.8676     4500
```

```
## ExpDay_factor9.units     0.8452   0.7475   0.9345     4500
## ExpDay_factor10.units    0.8446   0.7532   0.9400     4500
## ExpDay_factor11.units    0.6220   0.5514   0.6881     4500
## ExpDay_factor12.units    0.7073   0.6333   0.7895     4500
## ExpDay_factor13.units    0.7331   0.6517   0.8083     4500
## ExpDay_factor14.units    0.7177   0.6421   0.7978     3745
## ExpDay_factor15.units    0.7773   0.6915   0.8621     4500
## ExpDay_factor16.units    0.6923   0.6234   0.7767     4500
## ExpDay_factor17.units    0.6930   0.6168   0.7701     4500
## ExpDay_factor18.units    0.6913   0.6183   0.7686     4500
## ExpDay_factor19.units    0.8394   0.7515   0.9399     4560
## ExpDay_factor20.units    0.7502   0.6656   0.8349     4500
## ExpDay_factor21.units    0.7862   0.7017   0.8699     4500
## ExpDay_factor22.units    0.9714   0.8586   1.0749     4500
## ExpDay_factor23.units    0.9157   0.8173   1.0166     4500
## ExpDay_factor24.units    0.7253   0.6495   0.8073     4500
## ExpDay_factor25.units    0.8317   0.7382   0.9183     4500
## ExpDay_factor26.units    0.8189   0.7289   0.9130     4500
## ExpDay_factor27.units    0.6978   0.6163   0.7764     4500
##
##  Location effects: MedianVel_hourly_scaled ~ ExpDay + YearBatch + Hour
##
##              post.mean   l-95% CI   u-95% CI eff.samp   pMCMC
## (Intercept) -0.0496095 -0.4598848  0.3398565     3939 0.74178
## ExpDay       0.0024761  0.0005919  0.0043334     4500 0.00578 **
## YearBatch    0.0093789 -0.1604295  0.1617825     3901 0.84044
## Hour        -0.0248343 -0.0286402 -0.0208455     4500 < 2e-04 ***
## ---
## Signif. codes:  0 '***' 0.001 '**' 0.01 '*' 0.05 '.' 0.1 ' ' 1
```

```r
# FishId + MomId
model_predator_hetero_residual_id_mom <- readRDS("model_MedianVel_predator_hetero_residual_id_mom_8_21_25.rd
# model_predator_hetero_residual_id_mom <-
# MCMCglmm(MedianVel_hourly_scaled ~ ExpDay + YearBatch +
# Hour, random = ~FishId + MomId, rcov =
# ~idh(ExpDay_factor):units, family = 'gaussian', data =
# predator_data_hourly, prior =
# prior.fishId.momId.univariate.idhExpDay, nitt = 500000,
# burnin = 50000, thin = 100)
# saveRDS(model_predator_hetero_residual_id_mom, file =
# 'model_MedianVel_predator_hetero_residual_id_mom_8_21_25.rds')
summary(model_predator_hetero_residual_id_mom)
```

```
##
##  Iterations = 50001:499901
##  Thinning interval  = 100
##  Sample size  = 4500
##
##  DIC: 43773.43
##
##  G-structure:  ~FishId
##
##        post.mean l-95% CI u-95% CI eff.samp
## FishId    0.1206   0.0717   0.1802     4185
##
##                ~MomId
##
##       post.mean  l-95% CI u-95% CI eff.samp
## MomId    0.1108 1.268e-05   0.2761     1075
##
##  R-structure:  ~idh(ExpDay_factor):units
##
```

```
##                        post.mean l-95% CI u-95% CI eff.samp
## ExpDay_factor0.units      1.6088   1.4356   1.7984     4500
## ExpDay_factor1.units      1.2307   1.0953   1.3672     5200
## ExpDay_factor2.units      0.8117   0.7258   0.9099     4500
## ExpDay_factor3.units      1.0134   0.9047   1.1318     5016
## ExpDay_factor4.units      1.1065   0.9821   1.2266     4500
## ExpDay_factor5.units      0.9251   0.8249   1.0276     4500
## ExpDay_factor6.units      0.7162   0.6331   0.7924     4500
## ExpDay_factor7.units      0.6289   0.5615   0.7012     4500
## ExpDay_factor8.units      0.7250   0.6443   0.8040     4500
## ExpDay_factor9.units      0.7731   0.6869   0.8551     4500
## ExpDay_factor10.units     0.7516   0.6695   0.8333     4500
## ExpDay_factor11.units     0.4867   0.4337   0.5429     4500
## ExpDay_factor12.units     0.5916   0.5301   0.6586     4500
## ExpDay_factor13.units     0.6563   0.5851   0.7296     4500
## ExpDay_factor14.units     0.5926   0.5272   0.6569     4500
## ExpDay_factor15.units     0.6674   0.5947   0.7428     4500
## ExpDay_factor16.units     0.5852   0.5199   0.6509     4726
## ExpDay_factor17.units     0.6008   0.5339   0.6660     4500
## ExpDay_factor18.units     0.5727   0.5118   0.6345     4500
## ExpDay_factor19.units     0.7461   0.6600   0.8214     4500
## ExpDay_factor20.units     0.6648   0.5935   0.7370     4500
## ExpDay_factor21.units     0.6837   0.6151   0.7599     4227
## ExpDay_factor22.units     0.8597   0.7620   0.9522     4500
## ExpDay_factor23.units     0.8029   0.7144   0.8965     4500
## ExpDay_factor24.units     0.6416   0.5665   0.7118     4303
## ExpDay_factor25.units     0.7178   0.6396   0.7978     4500
## ExpDay_factor26.units     0.7582   0.6734   0.8427     4500
## ExpDay_factor27.units     0.6464   0.5800   0.7279     4500
##
##  Location effects: MedianVel_hourly_scaled ~ ExpDay + YearBatch + Hour
##
##              post.mean   l-95% CI   u-95% CI eff.samp  pMCMC
## (Intercept)  0.0340900 -0.3937926  0.4918100     3567 0.9364
## ExpDay       0.0019750  0.0002748  0.0037917     4500 0.0262 *
## YearBatch   -0.0127937 -0.1976277  0.1546852     3217 0.9511
## Hour        -0.0239319 -0.0277645 -0.0203682     4500 <2e-04 ***
## ---
## Signif. codes:  0 '***' 0.001 '**' 0.01 '*' 0.05 '.' 0.1 ' ' 1
```

```
# FishId slopes
model_predator_hetero_residual_id_slopes <- readRDS("model_MedianVel_predator_hetero_residual_id_slopes_8_2
# model_predator_hetero_residual_id_slopes <-
# MCMCglmm(MedianVel_hourly_scaled ~ ExpDay + YearBatch +
# Hour, random = ~us(1 + ExpDay):FishId, rcov =
# ~idh(ExpDay_factor):units, family = 'gaussian', data =
# predator_data_hourly, prior =
# prior.fishId.slopes.univariate.idhExpDay, nitt = 500000,
# burnin = 50000, thin = 100)
# saveRDS(model_predator_hetero_residual_id_slopes, file =
# 'model_MedianVel_predator_hetero_residual_id_slopes_8_21_25.rds')
summary(model_predator_hetero_residual_id_slopes)
```

```
##
##  Iterations = 50001:499901
##  Thinning interval  = 100
##  Sample size  = 4500
##
##  DIC: 42662.91
##
##  G-structure:  ~us(1 + ExpDay):FishId
##
```

```
##                               post.mean   l-95% CI  u-95% CI eff.samp
## (Intercept):(Intercept).FishId  0.236899  0.1563221  0.344428     3550
## ExpDay:(Intercept).FishId      -0.008364 -0.0137773 -0.003656     4264
## (Intercept):ExpDay.FishId      -0.008364 -0.0137773 -0.003656     4264
## ExpDay:ExpDay.FishId            0.001015  0.0006122  0.001471     3674
##
##  R-structure:  ~idh(ExpDay_factor):units
##
##                         post.mean l-95% CI u-95% CI eff.samp
## ExpDay_factor0.units     1.6071   1.4196   1.7990     4500
## ExpDay_factor1.units     1.0735   0.9605   1.1992     4500
## ExpDay_factor2.units     0.6556   0.5833   0.7375     4500
## ExpDay_factor3.units     0.7924   0.7048   0.8871     4500
## ExpDay_factor4.units     0.8959   0.7945   1.0015     4190
## ExpDay_factor5.units     0.7960   0.7085   0.8835     4500
## ExpDay_factor6.units     0.6373   0.5662   0.7088     4500
## ExpDay_factor7.units     0.5350   0.4747   0.5965     5085
## ExpDay_factor8.units     0.6541   0.5836   0.7302     5242
## ExpDay_factor9.units     0.7716   0.6914   0.8607     4500
## ExpDay_factor10.units    0.7541   0.6752   0.8401     4500
## ExpDay_factor11.units    0.5156   0.4585   0.5764     4500
## ExpDay_factor12.units    0.6126   0.5500   0.6830     4313
## ExpDay_factor13.units    0.6657   0.5926   0.7396     4500
## ExpDay_factor14.units    0.6019   0.5378   0.6695     4500
## ExpDay_factor15.units    0.6709   0.5982   0.7423     4500
## ExpDay_factor16.units    0.5792   0.5175   0.6431     4500
## ExpDay_factor17.units    0.5934   0.5299   0.6611     4500
## ExpDay_factor18.units    0.5462   0.4897   0.6084     4891
## ExpDay_factor19.units    0.7293   0.6526   0.8111     4500
## ExpDay_factor20.units    0.6288   0.5591   0.6935     4500
## ExpDay_factor21.units    0.6389   0.5688   0.7092     4500
## ExpDay_factor22.units    0.7790   0.6958   0.8642     4500
## ExpDay_factor23.units    0.7377   0.6567   0.8203     4500
## ExpDay_factor24.units    0.5807   0.5163   0.6428     4500
## ExpDay_factor25.units    0.6605   0.5870   0.7345     4708
## ExpDay_factor26.units    0.7081   0.6331   0.7915     4500
## ExpDay_factor27.units    0.6696   0.5940   0.7547     3897
##
##  Location effects: MedianVel_hourly_scaled ~ ExpDay + YearBatch + Hour
##
##             post.mean  l-95% CI  u-95% CI eff.samp  pMCMC
## (Intercept)  0.152263 -0.138725  0.425369     4453  0.296
## ExpDay       0.001177 -0.007255  0.010182     4918  0.797
## YearBatch   -0.050946 -0.154703  0.051173     4462  0.348
## Hour        -0.024695 -0.028263 -0.021105     4500 <2e-04 ***
## ---
## Signif. codes:  0 '***' 0.001 '**' 0.01 '*' 0.05 '.' 0.1 ' ' 1
```

```r
# FishId slopes + MomId
model_predator_hetero_residual_id_slopes_mom <- readRDS("model_MedianVel_predator_hetero_residual_id_slopes_
# model_predator_hetero_residual_id_slopes_mom <-
# MCMCglmm(MedianVel_hourly_scaled ~ ExpDay + YearBatch +
# Hour, random = ~us(1 + ExpDay):FishId + MomId, rcov =
# ~idh(ExpDay_factor):units, family = 'gaussian', data =
# predator_data_hourly, prior =
# prior.fishId.slopes.momId.univariate.idhExpDay, nitt =
# 500000, burnin = 50000, thin = 100)
# saveRDS(model_predator_hetero_residual_id_slopes_mom,
# file =
# 'model_MedianVel_predator_hetero_residual_id_slopes_mom_8_21_25.rds')
summary(model_predator_hetero_residual_id_slopes_mom)
```

```
##
##  Iterations = 50001:499901
##  Thinning interval  = 100
##  Sample size  = 4500
##
##  DIC: 42647.42
##
##  G-structure:  ~us(1 + ExpDay):FishId
##
##                               post.mean   l-95% CI  u-95% CI eff.samp
## (Intercept):(Intercept).FishId  0.175179  0.0964578  0.266957     4185
## ExpDay:(Intercept).FishId      -0.008422 -0.0139001 -0.003039     4500
## (Intercept):ExpDay.FishId      -0.008422 -0.0139001 -0.003039     4500
## ExpDay:ExpDay.FishId            0.001020  0.0006245  0.001465     3385
##
##               ~MomId
##
##       post.mean l-95% CI u-95% CI eff.samp
## MomId    0.8465 0.009409    2.804    60.81
##
##  R-structure:  ~idh(ExpDay_factor):units
##
##                      post.mean l-95% CI u-95% CI eff.samp
## ExpDay_factor0.units     1.5690   1.3947   1.7584     2478
## ExpDay_factor1.units     1.0781   0.9625   1.2034     3170
## ExpDay_factor2.units     0.6552   0.5828   0.7307     4500
## ExpDay_factor3.units     0.7921   0.7088   0.8903     4500
## ExpDay_factor4.units     0.8954   0.7974   1.0011     4500
## ExpDay_factor5.units     0.7965   0.7054   0.8810     4500
## ExpDay_factor6.units     0.6349   0.5627   0.7082     4500
## ExpDay_factor7.units     0.5350   0.4752   0.5941     4058
## ExpDay_factor8.units     0.6537   0.5837   0.7278     4500
## ExpDay_factor9.units     0.7718   0.6857   0.8581     4500
## ExpDay_factor10.units    0.7535   0.6734   0.8375     4500
## ExpDay_factor11.units    0.5156   0.4590   0.5746     4175
## ExpDay_factor12.units    0.6118   0.5442   0.6788     5168
## ExpDay_factor13.units    0.6652   0.5931   0.7432     4500
## ExpDay_factor14.units    0.6017   0.5348   0.6663     4343
## ExpDay_factor15.units    0.6703   0.5949   0.7425     4500
## ExpDay_factor16.units    0.5784   0.5128   0.6408     4646
## ExpDay_factor17.units    0.5931   0.5312   0.6640     4500
## ExpDay_factor18.units    0.5462   0.4856   0.6050     4500
## ExpDay_factor19.units    0.7284   0.6523   0.8089     4638
## ExpDay_factor20.units    0.6283   0.5580   0.6975     4500
## ExpDay_factor21.units    0.6379   0.5699   0.7091     4282
## ExpDay_factor22.units    0.7803   0.6979   0.8696     4500
## ExpDay_factor23.units    0.7382   0.6645   0.8265     4500
## ExpDay_factor24.units    0.5813   0.5180   0.6480     4500
## ExpDay_factor25.units    0.6602   0.5838   0.7304     4500
## ExpDay_factor26.units    0.7094   0.6299   0.7885     4439
## ExpDay_factor27.units    0.6711   0.5902   0.7460     4500
##
##  Location effects: MedianVel_hourly_scaled ~ ExpDay + YearBatch + Hour
##
##             post.mean  l-95% CI  u-95% CI eff.samp  pMCMC
## (Intercept)  1.095474 -0.088326  2.557472    120.8 0.0431 *
## ExpDay       0.001077 -0.008301  0.009693   5308.2 0.7956
## YearBatch   -0.497704 -1.113841 -0.009248    125.6 0.0209 *
## Hour        -0.024712 -0.028395 -0.020990   4243.7 <2e-04 ***
## ---
## Signif. codes:  0 '***' 0.001 '**' 0.01 '*' 0.05 '.' 0.1 ' ' 1
```

```r
# FishId slopes + MomId slopes
model_predator_hetero_residual_id_slopes_mom_slopes <- readRDS("model_MedianVel_predator_hetero_residual_id_
# model_predator_hetero_residual_id_slopes_mom_slopes <-
# MCMCglmm(MedianVel_hourly_scaled ~ ExpDay + YearBatch +
# Hour, random = ~us(1 + ExpDay):FishId + us(1 +
# ExpDay):MomId, rcov = ~idh(ExpDay_factor):units, family =
# 'gaussian', data = predator_data_hourly, prior =
# prior.fishId.slopes.momId.slopes.univariate.idhExpDay,
# nitt = 500000, burnin = 50000, thin = 100)
# saveRDS(model_predator_hetero_residual_id_slopes_mom_slopes,
# file =
# 'model_MedianVel_predator_hetero_residual_id_slopes_mom_slopes_8_21_25.rds')
summary(model_predator_hetero_residual_id_slopes_mom_slopes)  # Best fit
```

```
##
##  Iterations = 50001:499901
##  Thinning interval  = 100
##  Sample size  = 4500
##
##  DIC: 42642.24
##
##  G-structure:  ~us(1 + ExpDay):FishId
##
##                                 post.mean   l-95% CI   u-95% CI eff.samp
## (Intercept):(Intercept).FishId  0.1500944  0.0814658  0.2246407     2488
## ExpDay:(Intercept).FishId      -0.0051115 -0.0090561 -0.0014691     4138
## (Intercept):ExpDay.FishId      -0.0051115 -0.0090561 -0.0014691     4138
## ExpDay:ExpDay.FishId            0.0005832  0.0003284  0.0009057     2385
##
##                ~us(1 + ExpDay):MomId
##
##                               post.mean   l-95% CI u-95% CI eff.samp
## (Intercept):(Intercept).MomId  1.086694  0.0088954 3.205246      139
## ExpDay:(Intercept).MomId       0.002938 -0.0196454 0.027276     1751
## (Intercept):ExpDay.MomId       0.002938 -0.0196454 0.027276     1751
## ExpDay:ExpDay.MomId            0.000882  0.0000495 0.002038     1043
##
##  R-structure:  ~idh(ExpDay_factor):units
##
##                      post.mean l-95% CI u-95% CI eff.samp
## ExpDay_factor0.units    1.5575   1.3749   1.7378     3451
## ExpDay_factor1.units    1.0772   0.9498   1.1970     4500
## ExpDay_factor2.units    0.6566   0.5801   0.7331     4867
## ExpDay_factor3.units    0.7915   0.6985   0.8804     4500
## ExpDay_factor4.units    0.8931   0.7969   0.9999     4500
## ExpDay_factor5.units    0.7929   0.7004   0.8794     4500
## ExpDay_factor6.units    0.6349   0.5659   0.7075     4979
## ExpDay_factor7.units    0.5347   0.4772   0.5982     4500
## ExpDay_factor8.units    0.6533   0.5830   0.7300     4500
## ExpDay_factor9.units    0.7722   0.6874   0.8602     5428
## ExpDay_factor10.units   0.7537   0.6719   0.8426     4500
## ExpDay_factor11.units   0.5158   0.4622   0.5755     4500
## ExpDay_factor12.units   0.6126   0.5471   0.6829     4500
## ExpDay_factor13.units   0.6650   0.5936   0.7385     4500
## ExpDay_factor14.units   0.6027   0.5376   0.6689     4505
## ExpDay_factor15.units   0.6708   0.6000   0.7440     4368
## ExpDay_factor16.units   0.5782   0.5165   0.6436     4500
## ExpDay_factor17.units   0.5923   0.5241   0.6567     4500
## ExpDay_factor18.units   0.5463   0.4867   0.6061     4500
## ExpDay_factor19.units   0.7293   0.6507   0.8087     4500
## ExpDay_factor20.units   0.6284   0.5614   0.6993     4500
```

```
## ExpDay_factor21.units    0.6385   0.5735   0.7125     4500
## ExpDay_factor22.units    0.7780   0.6982   0.8673     4702
## ExpDay_factor23.units    0.7380   0.6564   0.8172     4500
## ExpDay_factor24.units    0.5813   0.5177   0.6437     4500
## ExpDay_factor25.units    0.6610   0.5844   0.7302     4500
## ExpDay_factor26.units    0.7093   0.6308   0.7896     4830
## ExpDay_factor27.units    0.6734   0.5965   0.7528     4500
##
##  Location effects: MedianVel_hourly_scaled ~ ExpDay + YearBatch + Hour
##
##             post.mean   l-95% CI   u-95% CI eff.samp   pMCMC
## (Intercept)  1.3634795  0.0351500  2.7452899    172.0 0.01733 *
## ExpDay       0.0009609 -0.0181132  0.0185839   4500.0 0.90489
## YearBatch   -0.6188762 -1.1680861 -0.0393858    130.3 0.00711 **
## Hour        -0.0247592 -0.0283685 -0.0212023   4719.1 < 2e-04 ***
## ---
## Signif. codes:  0 '***' 0.001 '**' 0.01 '*' 0.05 '.' 0.1 ' ' 1
```

```r
# FishId slopes + MomId slopes no YearBatch
model_predator_hetero_residual_id_slopes_mom_slopes_noYearBatch <- readRDS("model_MedianVel_predator_hetero_
# model_predator_hetero_residual_id_slopes_mom_slopes_noYearBatch
# <- MCMCglmm(MedianVel_hourly_scaled ~ ExpDay + Hour,
# random = ~us(1 + ExpDay):FishId + us(1 + ExpDay):MomId,
# rcov = ~idh(ExpDay_factor):units, family = 'gaussian',
# data = predator_data_hourly, prior =
# prior.fishId.slopes.momId.slopes.univariate.idhExpDay,
# nitt = 500000, burnin = 50000, thin = 100)
# saveRDS(model_predator_hetero_residual_id_slopes_mom_slopes_noYearBatch,
# file =
# 'model_MedianVel_predator_hetero_residual_id_slopes_mom_slopes_noYearBatch_8_21_25.rds')
summary(model_predator_hetero_residual_id_slopes_mom_slopes_noYearBatch)
```

```
##
##  Iterations = 50001:499901
##  Thinning interval  = 100
##  Sample size  = 4500
##
##  DIC: 42663.73
##
##  G-structure:  ~us(1 + ExpDay):FishId
##
##                                post.mean   l-95% CI   u-95% CI eff.samp
## (Intercept):(Intercept).FishId  0.1581816  0.0870786  0.2440607     2998
## ExpDay:(Intercept).FishId      -0.0055797 -0.0098589 -0.0015654     3563
## (Intercept):ExpDay.FishId      -0.0055797 -0.0098589 -0.0015654     3563
## ExpDay:ExpDay.FishId            0.0006031  0.0003131  0.0009369     2684
##
##                ~us(1 + ExpDay):MomId
##
##                               post.mean   l-95% CI u-95% CI eff.samp
## (Intercept):(Intercept).MomId  0.1395106  1.176e-06 0.333223     1354
## ExpDay:(Intercept).MomId      -0.0050892 -1.554e-02 0.001679     3538
## (Intercept):ExpDay.MomId      -0.0050892 -1.554e-02 0.001679     3538
## ExpDay:ExpDay.MomId            0.0006789  5.678e-07 0.001529     1649
##
##  R-structure:  ~idh(ExpDay_factor):units
##
##                       post.mean l-95% CI u-95% CI eff.samp
## ExpDay_factor0.units     1.6171   1.4332   1.7979     4462
## ExpDay_factor1.units     1.0783   0.9547   1.1968     4500
## ExpDay_factor2.units     0.6589   0.5862   0.7386     4500
## ExpDay_factor3.units     0.7944   0.7023   0.8848     4500
```

```
## ExpDay_factor4.units     0.8972   0.7974   0.9992     4500
## ExpDay_factor5.units     0.7930   0.7040   0.8854     4500
## ExpDay_factor6.units     0.6361   0.5619   0.7080     4217
## ExpDay_factor7.units     0.5353   0.4788   0.5968     4191
## ExpDay_factor8.units     0.6536   0.5802   0.7236     4500
## ExpDay_factor9.units     0.7724   0.6889   0.8581     4500
## ExpDay_factor10.units    0.7526   0.6725   0.8368     4500
## ExpDay_factor11.units    0.5134   0.4547   0.5719     4840
## ExpDay_factor12.units    0.6123   0.5485   0.6813     4500
## ExpDay_factor13.units    0.6647   0.5897   0.7368     4500
## ExpDay_factor14.units    0.6011   0.5340   0.6695     4500
## ExpDay_factor15.units    0.6719   0.6019   0.7490     4500
## ExpDay_factor16.units    0.5788   0.5179   0.6442     4500
## ExpDay_factor17.units    0.5920   0.5317   0.6599     4500
## ExpDay_factor18.units    0.5474   0.4868   0.6059     4500
## ExpDay_factor19.units    0.7295   0.6498   0.8087     4500
## ExpDay_factor20.units    0.6296   0.5593   0.6984     4500
## ExpDay_factor21.units    0.6384   0.5664   0.7104     4500
## ExpDay_factor22.units    0.7785   0.6957   0.8651     4500
## ExpDay_factor23.units    0.7395   0.6596   0.8236     4581
## ExpDay_factor24.units    0.5807   0.5137   0.6419     4957
## ExpDay_factor25.units    0.6602   0.5900   0.7396     4500
## ExpDay_factor26.units    0.7078   0.6284   0.7897     4500
## ExpDay_factor27.units    0.6698   0.5898   0.7486     4500
##
##  Location effects: MedianVel_hourly_scaled ~ ExpDay + Hour
##
##             post.mean  l-95% CI  u-95% CI eff.samp  pMCMC
## (Intercept) -0.008698 -0.238230  0.237174     4500  0.977
## ExpDay       0.001166 -0.014343  0.017383     4214  0.872
## Hour        -0.024697 -0.028278 -0.021105     4500 <2e-04 ***
## ---
## Signif. codes:  0 '***' 0.001 '**' 0.01 '*' 0.05 '.' 0.1 ' ' 1
```

```r
# FishId slopes + MomId slopes no Hour
model_predator_hetero_residual_id_slopes_mom_slopes_noHour <- readRDS("model_MedianVel_predator_hetero_resid
# model_predator_hetero_residual_id_slopes_mom_slopes_noHour
# <- MCMCglmm(MedianVel_hourly_scaled ~ ExpDay + YearBatch,
# random = ~us(1 + ExpDay):FishId + us(1 + ExpDay):MomId,
# rcov = ~idh(ExpDay_factor):units, family = 'gaussian',
# data = predator_data_hourly, prior =
# prior.fishId.slopes.momId.slopes.univariate.idhExpDay,
# nitt = 500000, burnin = 50000, thin = 100)
# saveRDS(model_predator_hetero_residual_id_slopes_mom_slopes_noHour,
# file =
# 'model_MedianVel_predator_hetero_residual_id_slopes_mom_slopes_noHour_8_22_25.rds')
summary(model_predator_hetero_residual_id_slopes_mom_slopes_noHour)
```

```
##
##  Iterations = 50001:499901
##  Thinning interval  = 100
##  Sample size  = 4500
##
##  DIC: 42820.42
##
##  G-structure:  ~us(1 + ExpDay):FishId
##
##                                  post.mean   l-95% CI   u-95% CI eff.samp
## (Intercept):(Intercept).FishId  0.1513092  0.0865553  0.2322088     3596
## ExpDay:(Intercept).FishId      -0.0051607 -0.0091971 -0.0016489     4158
## (Intercept):ExpDay.FishId      -0.0051607 -0.0091971 -0.0016489     4158
## ExpDay:ExpDay.FishId            0.0005879  0.0003096  0.0009069     2629
```

```
##
##                ~us(1 + ExpDay):MomId
##
##                               post.mean   l-95% CI u-95% CI eff.samp
## (Intercept):(Intercept).MomId 0.8867325  0.0027147 2.579885    158.4
## ExpDay:(Intercept).MomId      0.0021250 -0.0160166 0.026734   1128.9
## (Intercept):ExpDay.MomId      0.0021250 -0.0160166 0.026734   1128.9
## ExpDay:ExpDay.MomId           0.0008234  0.0000617 0.001916    914.5
##
##  R-structure:  ~idh(ExpDay_factor):units
##
##                         post.mean l-95% CI u-95% CI eff.samp
## ExpDay_factor0.units      1.5960   1.4152   1.7893     3381
## ExpDay_factor1.units      1.0903   0.9736   1.2155     4162
## ExpDay_factor2.units      0.6848   0.6049   0.7654     4699
## ExpDay_factor3.units      0.8009   0.7123   0.9003     4500
## ExpDay_factor4.units      0.9165   0.8104   1.0149     4500
## ExpDay_factor5.units      0.7928   0.6996   0.8795     4500
## ExpDay_factor6.units      0.6591   0.5857   0.7346     4500
## ExpDay_factor7.units      0.5486   0.4857   0.6086     4250
## ExpDay_factor8.units      0.6585   0.5881   0.7323     4111
## ExpDay_factor9.units      0.7872   0.7020   0.8727     4500
## ExpDay_factor10.units     0.7570   0.6751   0.8446     4500
## ExpDay_factor11.units     0.5193   0.4641   0.5816     4123
## ExpDay_factor12.units     0.6070   0.5430   0.6766     4500
## ExpDay_factor13.units     0.6734   0.6015   0.7472     4500
## ExpDay_factor14.units     0.5905   0.5280   0.6552     4720
## ExpDay_factor15.units     0.6588   0.5901   0.7327     4374
## ExpDay_factor16.units     0.5833   0.5205   0.6488     4500
## ExpDay_factor17.units     0.5796   0.5150   0.6433     5136
## ExpDay_factor18.units     0.5499   0.4911   0.6106     4500
## ExpDay_factor19.units     0.7195   0.6435   0.8004     4500
## ExpDay_factor20.units     0.6328   0.5608   0.7011     4500
## ExpDay_factor21.units     0.6386   0.5698   0.7085     4575
## ExpDay_factor22.units     0.7796   0.6971   0.8662     4500
## ExpDay_factor23.units     0.7529   0.6712   0.8390     4500
## ExpDay_factor24.units     0.5959   0.5312   0.6637     4500
## ExpDay_factor25.units     0.6726   0.5976   0.7464     4500
## ExpDay_factor26.units     0.7308   0.6503   0.8138     4500
## ExpDay_factor27.units     0.6846   0.6062   0.7668     4955
##
##  Location effects: MedianVel_hourly_scaled ~ ExpDay + YearBatch
##
##             post.mean  l-95% CI  u-95% CI eff.samp  pMCMC
## (Intercept)  1.104651 -0.149779  2.446235    271.4 0.0676 .
## ExpDay       0.001224 -0.016568  0.018635   4500.0 0.8933
## YearBatch   -0.564723 -1.109364 -0.039902    247.1 0.0107 *
## ---
## Signif. codes:  0 '***' 0.001 '**' 0.01 '*' 0.05 '.' 0.1 ' ' 1
```

```
# Diagnostics on best fit model
autocorr.diag(model_predator_hetero_residual_id_slopes_mom_slopes$Sol)
```

```
##          (Intercept)       ExpDay YearBatch         Hour
## Lag 0      1.0000000  1.000000000 1.0000000  1.000000000
## Lag 100    0.3586021 -0.003134509 0.4205346 -0.023875226
## Lag 500    0.3299357 -0.006300463 0.3938237  0.007028493
## Lag 1000   0.2966511 -0.011196483 0.3508337  0.001122910
## Lag 5000   0.1062991  0.009890224 0.1279627 -0.005873505
```

```
autocorr.diag(model_predator_hetero_residual_id_slopes_mom_slopes$VCV)
```

```
##          (Intercept):(Intercept).FishId ExpDay:(Intercept).FishId
```

```
## Lag 0                    1.00000000            1.0000000000
## Lag 100                  0.06192093            0.0118310859
## Lag 500                  0.05248937           -0.0007576894
## Lag 1000                -0.00172497            0.0081664672
## Lag 5000                 0.01743569            0.0235812356
##          (Intercept):ExpDay.FishId ExpDay:ExpDay.FishId
## Lag 0                 1.0000000000          1.000000000
## Lag 100               0.0118310859          0.043808024
## Lag 500              -0.0007576894          0.027489336
## Lag 1000              0.0081664672          0.044191000
## Lag 5000              0.0235812356          0.005063253
##          (Intercept):(Intercept).MomId ExpDay:(Intercept).MomId
## Lag 0                        1.0000000              1.000000000
## Lag 100                      0.4663518             -0.003791428
## Lag 500                      0.4469540              0.053813587
## Lag 1000                     0.3785173              0.048565811
## Lag 5000                     0.1302939              0.001934455
##          (Intercept):ExpDay.MomId ExpDay:ExpDay.MomId ExpDay_factor0.units
## Lag 0                 1.000000000         1.000000000          1.000000000
## Lag 100              -0.003791428         0.243711332          0.043187621
## Lag 500               0.053813587         0.130772121          0.011665167
## Lag 1000              0.048565811         0.072250935          0.002920261
## Lag 5000              0.001934455        -0.001068706          0.008170953
##          ExpDay_factor1.units ExpDay_factor2.units ExpDay_factor3.units
## Lag 0             1.000000000         1.0000000000         1.0000000000
## Lag 100           0.007337872        -0.0085422915         0.0009117931
## Lag 500           0.003938657        -0.0040940392         0.0257474175
## Lag 1000          0.000585999        -0.0008375986        -0.0132677368
## Lag 5000         -0.001997916        -0.0239021183        -0.0099703512
##          ExpDay_factor4.units ExpDay_factor5.units ExpDay_factor6.units
## Lag 0             1.000000000          1.000000000          1.000000000
## Lag 100           0.001293854         -0.007787218         -0.008098026
## Lag 500          -0.005895711          0.008769768         -0.002238995
## Lag 1000         -0.017584106          0.012892081         -0.003261660
## Lag 5000          0.033151592         -0.006916220         -0.001690949
##          ExpDay_factor7.units ExpDay_factor8.units ExpDay_factor9.units
## Lag 0             1.000000000         1.0000000000          1.000000000
## Lag 100          -0.013350460         0.0001382045         -0.012461484
## Lag 500           0.001500281        -0.0055888710         -0.001879118
## Lag 1000         -0.017013842        -0.0025544799          0.018823311
## Lag 5000          0.003365660        -0.0047322794          0.016972985
##          ExpDay_factor10.units ExpDay_factor11.units ExpDay_factor12.units
## Lag 0              1.000000000           1.000000000           1.000000000
## Lag 100            0.016622667           0.014622004           0.015931870
## Lag 500            0.017347308          -0.003408192           0.003169807
## Lag 1000          -0.008196685          -0.029395687           0.010275473
## Lag 5000           0.027449653           0.011927099           0.018828689
##          ExpDay_factor13.units ExpDay_factor14.units ExpDay_factor15.units
## Lag 0               1.00000000           1.000000000           1.000000000
## Lag 100            -0.01593083          -0.022339917          -0.025060967
## Lag 500             0.01632848          -0.010953392          -0.017567452
## Lag 1000            0.01140910          -0.006843639           0.005612354
## Lag 5000            0.00595563          -0.015662209          -0.005578517
##          ExpDay_factor16.units ExpDay_factor17.units ExpDay_factor18.units
## Lag 0              1.000000000           1.000000000           1.000000000
## Lag 100            0.005713736          -0.008887462          -0.010906152
## Lag 500            0.012206840          -0.008479065          -0.014047846
## Lag 1000           0.001494379          -0.043592870          -0.019805825
## Lag 5000           0.020716404           0.003453136          -0.002635434
##          ExpDay_factor19.units ExpDay_factor20.units ExpDay_factor21.units
## Lag 0              1.000000000          1.0000000000           1.000000000
```

```
## Lag 100           -0.000388072         0.0028157383          0.015124119
## Lag 500           -0.019158950        -0.0002460054          0.008665955
## Lag 1000           0.008370777        -0.0218722411          0.020936472
## Lag 5000          -0.021193900         0.0070494926         -0.006041921
##          ExpDay_factor22.units ExpDay_factor23.units ExpDay_factor24.units
## Lag 0              1.0000000000          1.000000000           1.000000000
## Lag 100           -0.0221003696         -0.011872835          -0.005682646
## Lag 500            0.0004653851         -0.005794613           0.012475794
## Lag 1000          -0.0179204609          0.028171719          -0.021894949
## Lag 5000           0.0129699251         -0.029297708           0.020894077
##          ExpDay_factor25.units ExpDay_factor26.units ExpDay_factor27.units
## Lag 0                1.00000000          1.000000000           1.000000000
## Lag 100             -0.01553474         -0.015048616           0.003646467
## Lag 500              0.01029625          0.001202006           0.004823606
## Lag 1000            -0.02505136         -0.006916028           0.002279011
## Lag 5000            -0.01261801         -0.019275290           0.025877846
```

```r
effectiveSize(model_predator_hetero_residual_id_slopes_mom_slopes$Sol)
```

```
## (Intercept)      ExpDay   YearBatch        Hour
##    171.9789   4500.0000    130.2630   4719.0836
```

```r
effectiveSize(model_predator_hetero_residual_id_slopes_mom_slopes$VCV)
```

```
## (Intercept):(Intercept).FishId      ExpDay:(Intercept).FishId
##                      2487.5384                      4138.1295
##      (Intercept):ExpDay.FishId           ExpDay:ExpDay.FishId
##                      4138.1295                      2385.4031
##  (Intercept):(Intercept).MomId       ExpDay:(Intercept).MomId
##                       139.0271                      1751.0523
##       (Intercept):ExpDay.MomId            ExpDay:ExpDay.MomId
##                      1751.0523                      1042.9065
##           ExpDay_factor0.units           ExpDay_factor1.units
##                      3450.5175                      4500.0000
##           ExpDay_factor2.units           ExpDay_factor3.units
##                      4867.1575                      4500.0000
##           ExpDay_factor4.units           ExpDay_factor5.units
##                      4500.0000                      4500.0000
##           ExpDay_factor6.units           ExpDay_factor7.units
##                      4978.8871                      4500.0000
##           ExpDay_factor8.units           ExpDay_factor9.units
##                      4500.0000                      5428.3260
##          ExpDay_factor10.units          ExpDay_factor11.units
##                      4500.0000                      4500.0000
##          ExpDay_factor12.units          ExpDay_factor13.units
##                      4500.0000                      4500.0000
##          ExpDay_factor14.units          ExpDay_factor15.units
##                      4504.5448                      4367.5583
##          ExpDay_factor16.units          ExpDay_factor17.units
##                      4500.0000                      4500.0000
##          ExpDay_factor18.units          ExpDay_factor19.units
##                      4500.0000                      4500.0000
##          ExpDay_factor20.units          ExpDay_factor21.units
##                      4500.0000                      4500.0000
##          ExpDay_factor22.units          ExpDay_factor23.units
##                      4702.3531                      4500.0000
##          ExpDay_factor24.units          ExpDay_factor25.units
##                      4500.0000                      4500.0000
##          ExpDay_factor26.units          ExpDay_factor27.units
##                      4830.2116                      4500.0000
```

```r
# FishId slopes + MomId slopes with more iterations
model_predator_hetero_residual_id_slopes_mom_slopes_more_iterations <- readRDS("model_MedianVel_predator_het
```

```r
# model_predator_hetero_residual_id_slopes_mom_slopes_more_iterations
# <- MCMCglmm(MedianVel_hourly_scaled ~ ExpDay + YearBatch
# + Hour, random = ~us(1 + ExpDay):FishId + us(1 +
# ExpDay):MomId, rcov = ~idh(ExpDay_factor):units, family =
# 'gaussian', data = predator_data_hourly, prior =
# prior.fishId.slopes.momId.slopes.univariate.idhExpDay,
# nitt = 1500000, burnin = 150000, thin = 200)
# saveRDS(model_predator_hetero_residual_id_slopes_mom_slopes_more_iterations,
# file =
# 'model_MedianVel_predator_hetero_residual_id_slopes_mom_slopes_include_hour_8_8_25_more_iterations.rds')
summary(model_predator_hetero_residual_id_slopes_mom_slopes_more_iterations)
```

```
##
##  Iterations = 150001:1499801
##  Thinning interval  = 200
##  Sample size  = 6750
##
##  DIC: 42642.99
##
##  G-structure:  ~us(1 + ExpDay):FishId
##
##                                  post.mean  l-95% CI  u-95% CI eff.samp
## (Intercept):(Intercept).FishId  0.1509539  0.087670  0.230361     6043
## ExpDay:(Intercept).FishId      -0.0051597 -0.009158 -0.001730     4977
## (Intercept):ExpDay.FishId      -0.0051597 -0.009158 -0.001730     4977
## ExpDay:ExpDay.FishId            0.0005888  0.000315  0.000906     4493
##
##               ~us(1 + ExpDay):MomId
##
##                                post.mean   l-95% CI u-95% CI eff.samp
## (Intercept):(Intercept).MomId 1.0617849  1.541e-05 3.273291    225.2
## ExpDay:(Intercept).MomId      0.0025468 -1.912e-02 0.026330   3022.4
## (Intercept):ExpDay.MomId      0.0025468 -1.912e-02 0.026330   3022.4
## ExpDay:ExpDay.MomId           0.0008198  6.385e-05 0.001925   2496.8
##
##  R-structure:  ~idh(ExpDay_factor):units
##
##                         post.mean l-95% CI u-95% CI eff.samp
## ExpDay_factor0.units     1.5627   1.3951   1.7540     6054
## ExpDay_factor1.units     1.0772   0.9632   1.2053     6750
## ExpDay_factor2.units     0.6558   0.5809   0.7325     6750
## ExpDay_factor3.units     0.7923   0.6992   0.8812     6750
## ExpDay_factor4.units     0.8929   0.7914   0.9928     6750
## ExpDay_factor5.units     0.7938   0.7053   0.8811     6750
## ExpDay_factor6.units     0.6342   0.5635   0.7072     7238
## ExpDay_factor7.units     0.5351   0.4760   0.5958     7188
## ExpDay_factor8.units     0.6529   0.5809   0.7272     6750
## ExpDay_factor9.units     0.7720   0.6905   0.8596     6750
## ExpDay_factor10.units    0.7535   0.6710   0.8351     6750
## ExpDay_factor11.units    0.5157   0.4562   0.5731     6750
## ExpDay_factor12.units    0.6117   0.5481   0.6831     6750
## ExpDay_factor13.units    0.6641   0.5932   0.7382     6750
## ExpDay_factor14.units    0.6015   0.5366   0.6676     6750
## ExpDay_factor15.units    0.6708   0.5987   0.7427     6750
## ExpDay_factor16.units    0.5790   0.5149   0.6419     6750
## ExpDay_factor17.units    0.5923   0.5267   0.6558     6750
## ExpDay_factor18.units    0.5467   0.4875   0.6084     6750
## ExpDay_factor19.units    0.7295   0.6503   0.8118     6750
## ExpDay_factor20.units    0.6285   0.5628   0.6995     6750
## ExpDay_factor21.units    0.6386   0.5748   0.7175     6750
## ExpDay_factor22.units    0.7780   0.6907   0.8603     6750
```

```
## ExpDay_factor23.units    0.7384   0.6590   0.8224     6545
## ExpDay_factor24.units    0.5807   0.5195   0.6491     6750
## ExpDay_factor25.units    0.6612   0.5855   0.7328     6750
## ExpDay_factor26.units    0.7100   0.6267   0.7892     6750
## ExpDay_factor27.units    0.6724   0.5935   0.7500     6511
##
##  Location effects: MedianVel_hourly_scaled ~ ExpDay + YearBatch + Hour
##
##             post.mean   l-95% CI   u-95% CI eff.samp   pMCMC
## (Intercept)  1.3151809  0.0206929  2.7996497    324.1 0.02104 *
## ExpDay       0.0009867 -0.0167550  0.0185309   6750.0 0.89600
## YearBatch   -0.5975054 -1.1783167 -0.0452359    341.3 0.00919 **
## Hour        -0.0246992 -0.0283107 -0.0211033   6750.0 < 1e-04 ***
## ---
## Signif. codes:  0 '***' 0.001 '**' 0.01 '*' 0.05 '.' 0.1 ' ' 1
```

```
# Diagnostics on best fit model
autocorr.diag(model_predator_hetero_residual_id_slopes_mom_slopes_more_iterations$Sol)
```

```
##           (Intercept)       ExpDay  YearBatch          Hour
## Lag 0      1.00000000  1.000000000 1.00000000  1.0000000000
## Lag 200    0.35746968 -0.001880405 0.43719299  0.0006594019
## Lag 1000   0.30310967 -0.010729339 0.36722644 -0.0002676221
## Lag 2000   0.25008660 -0.002333558 0.30118938 -0.0028352184
## Lag 10000  0.05776781  0.012705649 0.07873048  0.0058845572
```

```
autocorr.diag(model_predator_hetero_residual_id_slopes_mom_slopes_more_iterations$VCV)
```

```
##           (Intercept):(Intercept).FishId ExpDay:(Intercept).FishId
## Lag 0                        1.000000000               1.000000000
## Lag 200                      0.018455695               0.002640315
## Lag 1000                    -0.004279370               0.015290845
## Lag 2000                    -0.009929144               0.015786417
## Lag 10000                    0.015774707               0.003694850
##           (Intercept):ExpDay.FishId ExpDay:ExpDay.FishId
## Lag 0                   1.000000000         1.0000000000
## Lag 200                 0.002640315         0.0439102427
## Lag 1000                0.015290845         0.0302405943
## Lag 2000                0.015786417         0.0315101994
## Lag 10000               0.003694850        -0.0003680252
##           (Intercept):(Intercept).MomId ExpDay:(Intercept).MomId
## Lag 0                        1.00000000             1.0000000000
## Lag 200                      0.49637284             0.0180040371
## Lag 1000                     0.44220149             0.0353013697
## Lag 2000                     0.37915005             0.0271670436
## Lag 10000                    0.09055574             0.0005645877
##           (Intercept):ExpDay.MomId ExpDay:ExpDay.MomId ExpDay_factor0.units
## Lag 0                 1.0000000000         1.000000000          1.000000000
## Lag 200               0.0180040371         0.233860627          0.027516199
## Lag 1000              0.0353013697         0.069791096          0.002223025
## Lag 2000              0.0271670436         0.042948980          0.021373131
## Lag 10000             0.0005645877        -0.002717564         -0.022496256
##           ExpDay_factor1.units ExpDay_factor2.units ExpDay_factor3.units
## Lag 0              1.000000000          1.000000000          1.000000000
## Lag 200            0.014421636          0.012118056         -0.007122055
## Lag 1000           0.016012077         -0.006203974         -0.016174891
## Lag 2000          -0.001328135          0.004967735          0.000762180
## Lag 10000          0.003988320          0.015141496         -0.010241882
##           ExpDay_factor4.units ExpDay_factor5.units ExpDay_factor6.units
## Lag 0              1.000000000          1.000000000          1.000000000
## Lag 200            0.003611221          0.007999522         -0.015080992
## Lag 1000           0.007543115         -0.013125338         -0.011534519
## Lag 2000          -0.030278342         -0.004548056         -0.004833665
```

```
## Lag 10000          -0.009132857         -0.003055805         -0.005188788
##             ExpDay_factor7.units ExpDay_factor8.units ExpDay_factor9.units
## Lag 0                1.000000000          1.000000000          1.000000000
## Lag 200              0.011993645         -0.007595558         -0.003352143
## Lag 1000            -0.007034605         -0.013044224         -0.018244804
## Lag 2000             0.001076382         -0.023646749         -0.019329570
## Lag 10000           -0.012756260         -0.003034045         -0.007082684
##             ExpDay_factor10.units ExpDay_factor11.units ExpDay_factor12.units
## Lag 0                 1.000000000           1.000000000          1.0000000000
## Lag 200              -0.002686498           0.001989374         -0.0012468902
## Lag 1000              0.021525437          -0.020288854         -0.0077862957
## Lag 2000              0.002070348           0.020527575         -0.0122579289
## Lag 10000            -0.025725900           0.006509091         -0.0008082614
##             ExpDay_factor13.units ExpDay_factor14.units ExpDay_factor15.units
## Lag 0                 1.000000000           1.000000000           1.000000000
## Lag 200               0.002693367           0.001829585          -0.005837657
## Lag 1000             -0.012949904           0.004422017          -0.001002733
## Lag 2000              0.014530353           0.019926315          -0.013421014
## Lag 10000             0.004385509          -0.018736762           0.003370189
##             ExpDay_factor16.units ExpDay_factor17.units ExpDay_factor18.units
## Lag 0                1.0000000000          1.0000000000          1.0000000000
## Lag 200             -0.0039252313         -0.0022973742         -0.0005266821
## Lag 1000            -0.0007697409          0.0009504182         -0.0140839786
## Lag 2000            -0.0038799692         -0.0137481759          0.0083153676
## Lag 10000           -0.0047081189          0.0191308150         -0.0176969060
##             ExpDay_factor19.units ExpDay_factor20.units ExpDay_factor21.units
## Lag 0                 1.000000000           1.000000000          1.0000000000
## Lag 200              -0.014347146          -0.012320728         -0.0096632698
## Lag 1000              0.022100511          -0.004358758         -0.0068066896
## Lag 2000             -0.016700697          -0.010435901         -0.0150886309
## Lag 10000            -0.002474098          -0.002946727         -0.0008291861
##             ExpDay_factor22.units ExpDay_factor23.units ExpDay_factor24.units
## Lag 0                 1.000000000           1.000000000           1.000000000
## Lag 200              -0.009225553          -0.008491650          -0.001995358
## Lag 1000             -0.020067253          -0.006389135          -0.006314713
## Lag 2000              0.011681693          -0.005935288           0.002418632
## Lag 10000             0.002515978           0.012947148           0.003789772
##             ExpDay_factor25.units ExpDay_factor26.units ExpDay_factor27.units
## Lag 0                1.0000000000           1.000000000           1.000000000
## Lag 200             -0.0007629081          -0.002784659          -0.008110794
## Lag 1000            -0.0048143646           0.005349859           0.001853622
## Lag 2000            -0.0217307896           0.011040528          -0.013627449
## Lag 10000            0.0016214667           0.023246791           0.018310204
```

```r
effectiveSize(model_predator_hetero_residual_id_slopes_mom_slopes_more_iterations$Sol)
```

```
## (Intercept)      ExpDay   YearBatch        Hour
##    324.1302   6750.0000    341.2673   6750.0000
```

```r
effectiveSize(model_predator_hetero_residual_id_slopes_mom_slopes_more_iterations$VCV)
```

```
## (Intercept):(Intercept).FishId      ExpDay:(Intercept).FishId
##                      6043.0256                      4977.3280
##      (Intercept):ExpDay.FishId           ExpDay:ExpDay.FishId
##                      4977.3280                      4492.5516
##  (Intercept):(Intercept).MomId       ExpDay:(Intercept).MomId
##                       225.1718                      3022.3906
##       (Intercept):ExpDay.MomId            ExpDay:ExpDay.MomId
##                      3022.3906                      2496.8386
##           ExpDay_factor0.units           ExpDay_factor1.units
##                      6053.6369                      6750.0000
##           ExpDay_factor2.units           ExpDay_factor3.units
##                      6750.0000                      6750.0000
```

```
##           ExpDay_factor4.units           ExpDay_factor5.units
##                      6750.0000                      6750.0000
##           ExpDay_factor6.units           ExpDay_factor7.units
##                      7237.7472                      7187.6600
##           ExpDay_factor8.units           ExpDay_factor9.units
##                      6750.0000                      6750.0000
##          ExpDay_factor10.units          ExpDay_factor11.units
##                      6750.0000                      6750.0000
##          ExpDay_factor12.units          ExpDay_factor13.units
##                      6750.0000                      6750.0000
##          ExpDay_factor14.units          ExpDay_factor15.units
##                      6750.0000                      6750.0000
##          ExpDay_factor16.units          ExpDay_factor17.units
##                      6750.0000                      6750.0000
##          ExpDay_factor18.units          ExpDay_factor19.units
##                      6750.0000                      6750.0000
##          ExpDay_factor20.units          ExpDay_factor21.units
##                      6750.0000                      6750.0000
##          ExpDay_factor22.units          ExpDay_factor23.units
##                      6750.0000                      6544.5937
##          ExpDay_factor24.units          ExpDay_factor25.units
##                      6750.0000                      6750.0000
##          ExpDay_factor26.units          ExpDay_factor27.units
##                      6750.0000                      6510.7093
```

```
# Since the best-fit predator model includes mom slopes, we
# must also include it in the control model so that the
# models have the same structure and we aren't missing
# important complexity in either model

# Run control model with FishId slopes + MomId slopes and
# more iterations
model_control_hetero_residual_id_slopes_mom_slopes_more_iterations <- readRDS("model_MedianVel_control_heter
# model_control_hetero_residual_id_slopes_mom_slopes_include_hour
# <- MCMCglmm(MedianVel_hourly_scaled ~ ExpDay + YearBatch
# + Hour, random = ~us(1 + ExpDay):FishId + us(1 +
# ExpDay):MomId, rcov = ~idh(ExpDay_factor):units, family =
# 'gaussian', data = control_data_hourly, prior =
# prior.fishId.slopes.momId.slopes.univariate.idhExpDay,
# nitt = 1500000, burnin = 150000, thin = 200)
# saveRDS(model_control_hetero_residual_id_slopes_mom_slopes_more_iterations,
# file =
# 'model_MedianVel_control_hetero_residual_id_slopes_mom_slopes_include_hour_8_8_25_more_iterations.rds')
summary(model_control_hetero_residual_id_slopes_mom_slopes_more_iterations)
```

```
##
##  Iterations = 150001:1499801
##  Thinning interval  = 200
##  Sample size  = 6750
##
##  DIC: 38710.61
##
##  G-structure:  ~us(1 + ExpDay):FishId
##
##                                post.mean   l-95% CI  u-95% CI eff.samp
## (Intercept):(Intercept).FishId  0.1857270  0.1110835  0.278085     6750
## ExpDay:(Intercept).FishId      -0.0087176 -0.0138194 -0.004394     6750
## (Intercept):ExpDay.FishId      -0.0087176 -0.0138194 -0.004394     6750
## ExpDay:ExpDay.FishId            0.0007819  0.0004278  0.001158     5900
##
##                ~us(1 + ExpDay):MomId
##
```

```
##                                 post.mean   l-95% CI  u-95% CI eff.samp
## (Intercept):(Intercept).MomId 0.1101785  5.326e-08 0.347021     2042
## ExpDay:(Intercept).MomId      0.0007759 -6.136e-03 0.007017     5585
## (Intercept):ExpDay.MomId      0.0007759 -6.136e-03 0.007017     5585
## ExpDay:ExpDay.MomId           0.0006128  5.127e-07 0.001496     2625
##
##  R-structure:  ~idh(ExpDay_factor):units
##
##                         post.mean l-95% CI u-95% CI eff.samp
## ExpDay_factor0.units      2.8003   2.4864   3.1228     6750
## ExpDay_factor1.units      1.0492   0.9286   1.1655     6750
## ExpDay_factor2.units      0.6935   0.6094   0.7725     6747
## ExpDay_factor3.units      0.5314   0.4692   0.5972     7191
## ExpDay_factor4.units      0.4685   0.4142   0.5216     7406
## ExpDay_factor5.units      0.4234   0.3782   0.4780     6750
## ExpDay_factor6.units      0.7538   0.6687   0.8489     6750
## ExpDay_factor7.units      0.7336   0.6452   0.8142     6997
## ExpDay_factor8.units      0.8205   0.7261   0.9136     6750
## ExpDay_factor9.units      0.6730   0.5969   0.7512     6750
## ExpDay_factor10.units     0.5977   0.5255   0.6777     6404
## ExpDay_factor11.units     0.5164   0.4571   0.5798     6750
## ExpDay_factor12.units     0.6899   0.6118   0.7758     6750
## ExpDay_factor13.units     0.8013   0.7093   0.8897     6415
## ExpDay_factor14.units     0.9151   0.8173   1.0282     6750
## ExpDay_factor15.units     1.0917   0.9701   1.2181     6750
## ExpDay_factor16.units     0.5811   0.5177   0.6518     6750
## ExpDay_factor17.units     0.6816   0.6062   0.7626     6750
## ExpDay_factor18.units     0.5079   0.4472   0.5682     6750
## ExpDay_factor19.units     0.8532   0.7512   0.9482     6750
## ExpDay_factor20.units     0.6408   0.5654   0.7168     6750
## ExpDay_factor21.units     0.6016   0.5369   0.6746     6750
## ExpDay_factor22.units     0.5331   0.4762   0.5996     6750
## ExpDay_factor23.units     0.6492   0.5758   0.7251     6750
## ExpDay_factor24.units     0.5187   0.4565   0.5796     6421
## ExpDay_factor25.units     0.8239   0.7300   0.9248     6750
## ExpDay_factor26.units     0.6332   0.5604   0.7119     6750
## ExpDay_factor27.units     0.5193   0.4587   0.5830     7234
##
##  Location effects: MedianVel_hourly_scaled ~ ExpDay + YearBatch + Hour
##
##              post.mean   l-95% CI   u-95% CI eff.samp   pMCMC
## (Intercept)  0.7060153  0.2289546  1.2696147     3849 0.00119 **
## ExpDay      -0.0001976 -0.0169033  0.0145965     7172 0.98104
## YearBatch   -0.2008097 -0.4375424 -0.0109358     3139 0.01807 *
## Hour        -0.0408431 -0.0445996 -0.0372435     7030 < 1e-04 ***
## ---
## Signif. codes:  0 '***' 0.001 '**' 0.01 '*' 0.05 '.' 0.1 ' ' 1
```

```
autocorr.diag(model_control_hetero_residual_id_slopes_mom_slopes_more_iterations$Sol)
```

```
##           (Intercept)        ExpDay    YearBatch         Hour
## Lag 0     1.000000000  1.0000000000 1.0000000000  1.000000000
## Lag 200   0.122060181  0.0003555803 0.1770380793 -0.020388989
## Lag 1000  0.021425689  0.0247564327 0.0416419647 -0.001910463
## Lag 2000  0.012407227  0.0008363254 0.0166717141 -0.022227593
## Lag 10000 0.006123091 -0.0116205752 0.0001465031  0.019686230
```

```
autocorr.diag(model_control_hetero_residual_id_slopes_mom_slopes_more_iterations$VCV)
```

```
##           (Intercept):(Intercept).FishId ExpDay:(Intercept).FishId
## Lag 0                        1.000000000              1.0000000000
## Lag 200                      0.010131938              0.0112118178
## Lag 1000                     0.003811202              0.0009134105
```

```
## Lag 2000                  -0.004344605             0.0007077612
## Lag 10000                 -0.001329431             0.0098602554
##          (Intercept):ExpDay.FishId ExpDay:ExpDay.FishId
## Lag 0                 1.0000000000         1.0000000000
## Lag 200               0.0112118178         0.0293809415
## Lag 1000              0.0009134105        -0.0003666887
## Lag 2000              0.0007077612         0.0244144337
## Lag 10000             0.0098602554         0.0037469874
##          (Intercept):(Intercept).MomId ExpDay:(Intercept).MomId
## Lag 0                       1.00000000              1.000000000
## Lag 200                     0.28796324              0.023444602
## Lag 1000                    0.08381948              0.015257066
## Lag 2000                    0.03086512             -0.003439155
## Lag 10000                   0.02127154             -0.012303019
##          (Intercept):ExpDay.MomId ExpDay:ExpDay.MomId ExpDay_factor0.units
## Lag 0                 1.000000000        1.0000000000          1.000000000
## Lag 200               0.023444602        0.2493665380          0.001160399
## Lag 1000              0.015257066        0.0742761508          0.016250910
## Lag 2000             -0.003439155        0.0133833151          0.020540028
## Lag 10000            -0.012303019       -0.0005655953          0.003410897
##          ExpDay_factor1.units ExpDay_factor2.units ExpDay_factor3.units
## Lag 0            1.0000000000           1.00000000          1.000000000
## Lag 200          0.0035159058          -0.02212563         -0.005408636
## Lag 1000        -0.0009514278          -0.01176573         -0.002065320
## Lag 2000         0.0006761063           0.01325676          0.005939690
## Lag 10000       -0.0106827296           0.02470136          0.013465920
##          ExpDay_factor4.units ExpDay_factor5.units ExpDay_factor6.units
## Lag 0            1.0000000000          1.000000000          1.000000000
## Lag 200          0.0009644676          0.008422648         -0.011396906
## Lag 1000        -0.0063218556          0.011604772         -0.001519396
## Lag 2000        -0.0019240309          0.018056013         -0.019636149
## Lag 10000       -0.0006232354          0.001132607          0.011971395
##          ExpDay_factor7.units ExpDay_factor8.units ExpDay_factor9.units
## Lag 0             1.000000000          1.000000000         1.0000000000
## Lag 200          -0.018044020         -0.011396666        -0.0045633810
## Lag 1000         -0.019381958          0.005004942        -0.0183149297
## Lag 2000          0.014697262          0.021196556        -0.0082585429
## Lag 10000        -0.005170962          0.011413507        -0.0009042604
##          ExpDay_factor10.units ExpDay_factor11.units ExpDay_factor12.units
## Lag 0             1.0000000000           1.000000000          1.0000000000
## Lag 200           0.0262129102           0.001388662         -0.0027916241
## Lag 1000         -0.0127083117           0.020450149         -0.0226180469
## Lag 2000          0.0005627785          -0.013538455          0.0298886605
## Lag 10000         0.0027843056           0.008578164          0.0005327153
##          ExpDay_factor13.units ExpDay_factor14.units ExpDay_factor15.units
## Lag 0              1.000000000           1.000000000           1.000000000
## Lag 200           -0.006563080          -0.013596741          -0.005545772
## Lag 1000          -0.009926919           0.008665156           0.001144655
## Lag 2000           0.013686179           0.008054665          -0.002107333
## Lag 10000          0.005318553          -0.009025423          -0.004186921
##          ExpDay_factor16.units ExpDay_factor17.units ExpDay_factor18.units
## Lag 0              1.000000000           1.000000000          1.000000e+00
## Lag 200           -0.005543052           0.001642521          6.770781e-03
## Lag 1000          -0.016952101           0.027209414          1.942068e-02
## Lag 2000           0.010522762           0.004652163         -8.979307e-05
## Lag 10000          0.006358250           0.007654437          1.223064e-02
##          ExpDay_factor19.units ExpDay_factor20.units ExpDay_factor21.units
## Lag 0             1.0000000000          1.0000000000          1.0000000000
## Lag 200          -0.0107602804          0.0144965231          0.0007232486
## Lag 1000          0.0152130941          0.0151069817          0.0035400080
## Lag 2000          0.0008116957         -0.0017599224          0.0027329912
```

```
## Lag 10000         -0.0077013094         -0.0001487064         -0.0235489503
##           ExpDay_factor22.units ExpDay_factor23.units ExpDay_factor24.units
## Lag 0              1.0000000000          1.0000000000           1.000000000
## Lag 200            0.0051790909         -0.0002596546           0.024907555
## Lag 1000           0.0134351629         -0.0142640103           0.006221701
## Lag 2000           0.0016752266         -0.0168853781          -0.032780680
## Lag 10000          0.0007543504         -0.0101007165           0.001933766
##           ExpDay_factor25.units ExpDay_factor26.units ExpDay_factor27.units
## Lag 0               1.000000000           1.000000000           1.000000000
## Lag 200             0.007986477           0.001443670          -0.018879210
## Lag 1000           -0.005772868           0.013836495           0.009043208
## Lag 2000            0.013868862           0.006679637          -0.011318139
## Lag 10000          -0.018634873           0.003908766          -0.008797643
```

```r
effectiveSize(model_control_hetero_residual_id_slopes_mom_slopes_more_iterations$Sol)
```

```
## (Intercept)      ExpDay   YearBatch        Hour
##    3849.030    7172.256    3139.307    7029.938
```

```r
effectiveSize(model_control_hetero_residual_id_slopes_mom_slopes_more_iterations$VCV)
```

```
## (Intercept):(Intercept).FishId      ExpDay:(Intercept).FishId
##                       6750.000                       6750.000
##      (Intercept):ExpDay.FishId           ExpDay:ExpDay.FishId
##                       6750.000                       5900.190
##  (Intercept):(Intercept).MomId       ExpDay:(Intercept).MomId
##                       2042.352                       5584.868
##       (Intercept):ExpDay.MomId            ExpDay:ExpDay.MomId
##                       5584.868                       2625.091
##           ExpDay_factor0.units           ExpDay_factor1.units
##                       6750.000                       6750.000
##           ExpDay_factor2.units           ExpDay_factor3.units
##                       6747.406                       7190.636
##           ExpDay_factor4.units           ExpDay_factor5.units
##                       7405.564                       6750.000
##           ExpDay_factor6.units           ExpDay_factor7.units
##                       6750.000                       6997.034
##           ExpDay_factor8.units           ExpDay_factor9.units
##                       6750.000                       6750.000
##          ExpDay_factor10.units          ExpDay_factor11.units
##                       6404.216                       6750.000
##          ExpDay_factor12.units          ExpDay_factor13.units
##                       6750.000                       6415.173
##          ExpDay_factor14.units          ExpDay_factor15.units
##                       6750.000                       6750.000
##          ExpDay_factor16.units          ExpDay_factor17.units
##                       6750.000                       6750.000
##          ExpDay_factor18.units          ExpDay_factor19.units
##                       6750.000                       6750.000
##          ExpDay_factor20.units          ExpDay_factor21.units
##                       6750.000                       6750.000
##          ExpDay_factor22.units          ExpDay_factor23.units
##                       6750.000                       6750.000
##          ExpDay_factor24.units          ExpDay_factor25.units
##                       6420.968                       6750.000
##          ExpDay_factor26.units          ExpDay_factor27.units
##                       6750.000                       7234.079
```

Our final univariate models include: Fixed effects: ExpDay, YearBatch, Hour.
Random effects: FishId with slopes, MomId with slopes

```r
model_control <- model_control_hetero_residual_id_slopes_mom_slopes_more_iterations
model_predator <- model_predator_hetero_residual_id_slopes_mom_slopes_more_iterations
summary(model_control)
```

```
##
##  Iterations = 150001:1499801
##  Thinning interval  = 200
##  Sample size  = 6750
##
##  DIC: 38710.61
##
##  G-structure:  ~us(1 + ExpDay):FishId
##
##                                 post.mean   l-95% CI  u-95% CI eff.samp
## (Intercept):(Intercept).FishId  0.1857270  0.1110835  0.278085     6750
## ExpDay:(Intercept).FishId      -0.0087176 -0.0138194 -0.004394     6750
## (Intercept):ExpDay.FishId      -0.0087176 -0.0138194 -0.004394     6750
## ExpDay:ExpDay.FishId            0.0007819  0.0004278  0.001158     5900
##
##                ~us(1 + ExpDay):MomId
##
##                               post.mean   l-95% CI u-95% CI eff.samp
## (Intercept):(Intercept).MomId 0.1101785  5.326e-08 0.347021     2042
## ExpDay:(Intercept).MomId      0.0007759 -6.136e-03 0.007017     5585
## (Intercept):ExpDay.MomId      0.0007759 -6.136e-03 0.007017     5585
## ExpDay:ExpDay.MomId           0.0006128  5.127e-07 0.001496     2625
##
##  R-structure:  ~idh(ExpDay_factor):units
##
##                        post.mean l-95% CI u-95% CI eff.samp
## ExpDay_factor0.units     2.8003   2.4864   3.1228     6750
## ExpDay_factor1.units     1.0492   0.9286   1.1655     6750
## ExpDay_factor2.units     0.6935   0.6094   0.7725     6747
## ExpDay_factor3.units     0.5314   0.4692   0.5972     7191
## ExpDay_factor4.units     0.4685   0.4142   0.5216     7406
## ExpDay_factor5.units     0.4234   0.3782   0.4780     6750
## ExpDay_factor6.units     0.7538   0.6687   0.8489     6750
## ExpDay_factor7.units     0.7336   0.6452   0.8142     6997
## ExpDay_factor8.units     0.8205   0.7261   0.9136     6750
## ExpDay_factor9.units     0.6730   0.5969   0.7512     6750
## ExpDay_factor10.units    0.5977   0.5255   0.6777     6404
## ExpDay_factor11.units    0.5164   0.4571   0.5798     6750
## ExpDay_factor12.units    0.6899   0.6118   0.7758     6750
## ExpDay_factor13.units    0.8013   0.7093   0.8897     6415
## ExpDay_factor14.units    0.9151   0.8173   1.0282     6750
## ExpDay_factor15.units    1.0917   0.9701   1.2181     6750
## ExpDay_factor16.units    0.5811   0.5177   0.6518     6750
## ExpDay_factor17.units    0.6816   0.6062   0.7626     6750
## ExpDay_factor18.units    0.5079   0.4472   0.5682     6750
## ExpDay_factor19.units    0.8532   0.7512   0.9482     6750
## ExpDay_factor20.units    0.6408   0.5654   0.7168     6750
## ExpDay_factor21.units    0.6016   0.5369   0.6746     6750
## ExpDay_factor22.units    0.5331   0.4762   0.5996     6750
## ExpDay_factor23.units    0.6492   0.5758   0.7251     6750
## ExpDay_factor24.units    0.5187   0.4565   0.5796     6421
## ExpDay_factor25.units    0.8239   0.7300   0.9248     6750
## ExpDay_factor26.units    0.6332   0.5604   0.7119     6750
## ExpDay_factor27.units    0.5193   0.4587   0.5830     7234
##
##  Location effects: MedianVel_hourly_scaled ~ ExpDay + YearBatch + Hour
##
##               post.mean   l-95% CI   u-95% CI eff.samp   pMCMC
## (Intercept)  0.7060153  0.2289546  1.2696147     3849 0.00119 **
## ExpDay      -0.0001976 -0.0169033  0.0145965     7172 0.98104
```

```
## YearBatch   -0.2008097 -0.4375424 -0.0109358     3139 0.01807 *
## Hour        -0.0408431 -0.0445996 -0.0372435     7030 < 1e-04 ***
## ---
## Signif. codes:  0 '***' 0.001 '**' 0.01 '*' 0.05 '.' 0.1 ' ' 1
```

```r
summary(model_predator)
```

```
##
##  Iterations = 150001:1499801
##  Thinning interval  = 200
##  Sample size  = 6750
##
##  DIC: 42642.99
##
##  G-structure:  ~us(1 + ExpDay):FishId
##
##                                post.mean  l-95% CI  u-95% CI eff.samp
## (Intercept):(Intercept).FishId  0.1509539  0.087670  0.230361     6043
## ExpDay:(Intercept).FishId      -0.0051597 -0.009158 -0.001730     4977
## (Intercept):ExpDay.FishId      -0.0051597 -0.009158 -0.001730     4977
## ExpDay:ExpDay.FishId            0.0005888  0.000315  0.000906     4493
##
##                ~us(1 + ExpDay):MomId
##
##                               post.mean   l-95% CI u-95% CI eff.samp
## (Intercept):(Intercept).MomId 1.0617849  1.541e-05 3.273291    225.2
## ExpDay:(Intercept).MomId      0.0025468 -1.912e-02 0.026330   3022.4
## (Intercept):ExpDay.MomId      0.0025468 -1.912e-02 0.026330   3022.4
## ExpDay:ExpDay.MomId           0.0008198  6.385e-05 0.001925   2496.8
##
##  R-structure:  ~idh(ExpDay_factor):units
##
##                         post.mean l-95% CI u-95% CI eff.samp
## ExpDay_factor0.units       1.5627   1.3951   1.7540     6054
## ExpDay_factor1.units       1.0772   0.9632   1.2053     6750
## ExpDay_factor2.units       0.6558   0.5809   0.7325     6750
## ExpDay_factor3.units       0.7923   0.6992   0.8812     6750
## ExpDay_factor4.units       0.8929   0.7914   0.9928     6750
## ExpDay_factor5.units       0.7938   0.7053   0.8811     6750
## ExpDay_factor6.units       0.6342   0.5635   0.7072     7238
## ExpDay_factor7.units       0.5351   0.4760   0.5958     7188
## ExpDay_factor8.units       0.6529   0.5809   0.7272     6750
## ExpDay_factor9.units       0.7720   0.6905   0.8596     6750
## ExpDay_factor10.units      0.7535   0.6710   0.8351     6750
## ExpDay_factor11.units      0.5157   0.4562   0.5731     6750
## ExpDay_factor12.units      0.6117   0.5481   0.6831     6750
## ExpDay_factor13.units      0.6641   0.5932   0.7382     6750
## ExpDay_factor14.units      0.6015   0.5366   0.6676     6750
## ExpDay_factor15.units      0.6708   0.5987   0.7427     6750
## ExpDay_factor16.units      0.5790   0.5149   0.6419     6750
## ExpDay_factor17.units      0.5923   0.5267   0.6558     6750
## ExpDay_factor18.units      0.5467   0.4875   0.6084     6750
## ExpDay_factor19.units      0.7295   0.6503   0.8118     6750
## ExpDay_factor20.units      0.6285   0.5628   0.6995     6750
## ExpDay_factor21.units      0.6386   0.5748   0.7175     6750
## ExpDay_factor22.units      0.7780   0.6907   0.8603     6750
## ExpDay_factor23.units      0.7384   0.6590   0.8224     6545
## ExpDay_factor24.units      0.5807   0.5195   0.6491     6750
## ExpDay_factor25.units      0.6612   0.5855   0.7328     6750
## ExpDay_factor26.units      0.7100   0.6267   0.7892     6750
## ExpDay_factor27.units      0.6724   0.5935   0.7500     6511
##
```

```
##  Location effects: MedianVel_hourly_scaled ~ ExpDay + YearBatch + Hour
##
##               post.mean   l-95% CI   u-95% CI eff.samp   pMCMC
## (Intercept)  1.3151809  0.0206929  2.7996497    324.1 0.02104 *
## ExpDay       0.0009867 -0.0167550  0.0185309   6750.0 0.89600
## YearBatch   -0.5975054 -1.1783167 -0.0452359    341.3 0.00919 **
## Hour        -0.0246992 -0.0283107 -0.0211033   6750.0 < 1e-04 ***
## ---
## Signif. codes:  0 '***' 0.001 '**' 0.01 '*' 0.05 '.' 0.1 ' ' 1
```

```r
summary(model_control$Sol)
```

```
##
## Iterations = 150001:1499801
## Thinning interval = 200
## Number of chains = 1
## Sample size per chain = 6750
##
## 1. Empirical mean and standard deviation for each variable,
##    plus standard error of the mean:
##
##                   Mean       SD  Naive SE Time-series SE
## (Intercept)  0.7060153 0.279030 3.396e-03      4.498e-03
## ExpDay      -0.0001976 0.007986 9.721e-05      9.430e-05
## YearBatch   -0.2008097 0.115755 1.409e-03      2.066e-03
## Hour        -0.0408431 0.001894 2.306e-05      2.259e-05
##
## 2. Quantiles for each variable:
##
##                 2.5%       25%        50%       75%    97.5%
## (Intercept)  0.27650  0.530262  0.6709938  0.838509  1.35010
## ExpDay      -0.01614 -0.005208 -0.0001481  0.004834  0.01561
## YearBatch   -0.47647 -0.251012 -0.1833754 -0.127560 -0.03159
## Hour        -0.04456 -0.042156 -0.0408135 -0.039544 -0.03718
```

```r
summary(model_predator$Sol)
```

```
##
## Iterations = 150001:1499801
## Thinning interval = 200
## Number of chains = 1
## Sample size per chain = 6750
##
## 1. Empirical mean and standard deviation for each variable,
##    plus standard error of the mean:
##
##                   Mean       SD  Naive SE Time-series SE
## (Intercept)  1.3151809 0.772136 9.398e-03      4.289e-02
## ExpDay       0.0009867 0.008871 1.080e-04      1.080e-04
## YearBatch   -0.5975054 0.318763 3.880e-03      1.726e-02
## Hour        -0.0246992 0.001838 2.237e-05      2.237e-05
##
## 2. Quantiles for each variable:
##
##                 2.5%       25%       50%       75%    97.5%
## (Intercept)  0.11055  0.705765  1.232219  1.836822  2.99345
## ExpDay      -0.01667 -0.004675  0.001094  0.006609  0.01867
## YearBatch   -1.22656 -0.832215 -0.588279 -0.339426 -0.07105
## Hour        -0.02831 -0.025928 -0.024706 -0.023464 -0.02109
```

```r
summary(model_control$VCV)
```

```
##
```

```
## Iterations = 150001:1499801
## Thinning interval = 200
## Number of chains = 1
## Sample size per chain = 6750
##
## 1. Empirical mean and standard deviation for each variable,
##    plus standard error of the mean:
##
##                                       Mean        SD  Naive SE Time-series SE
## (Intercept):(Intercept).FishId  0.1857270 0.0446929 5.440e-04      5.440e-04
## ExpDay:(Intercept).FishId      -0.0087176 0.0025081 3.053e-05      3.053e-05
## (Intercept):ExpDay.FishId      -0.0087176 0.0025081 3.053e-05      3.053e-05
## ExpDay:ExpDay.FishId            0.0007819 0.0001989 2.421e-06      2.589e-06
## (Intercept):(Intercept).MomId   0.1101785 0.1658529 2.019e-03      3.670e-03
## ExpDay:(Intercept).MomId        0.0007759 0.0033379 4.063e-05      4.466e-05
## (Intercept):ExpDay.MomId        0.0007759 0.0033379 4.063e-05      4.466e-05
## ExpDay:ExpDay.MomId             0.0006128 0.0004530 5.514e-06      8.842e-06
## ExpDay_factor0.units            2.8003390 0.1659888 2.020e-03      2.020e-03
## ExpDay_factor1.units            1.0491745 0.0610942 7.436e-04      7.436e-04
## ExpDay_factor2.units            0.6934877 0.0420393 5.117e-04      5.118e-04
## ExpDay_factor3.units            0.5314417 0.0328501 3.998e-04      3.874e-04
## ExpDay_factor4.units            0.4684885 0.0277901 3.382e-04      3.229e-04
## ExpDay_factor5.units            0.4233573 0.0255514 3.110e-04      3.110e-04
## ExpDay_factor6.units            0.7537673 0.0465648 5.668e-04      5.668e-04
## ExpDay_factor7.units            0.7335834 0.0429076 5.223e-04      5.130e-04
## ExpDay_factor8.units            0.8204592 0.0480706 5.851e-04      5.851e-04
## ExpDay_factor9.units            0.6730415 0.0398843 4.855e-04      4.855e-04
## ExpDay_factor10.units           0.5976523 0.0385044 4.687e-04      4.811e-04
## ExpDay_factor11.units           0.5164218 0.0317403 3.863e-04      3.863e-04
## ExpDay_factor12.units           0.6898596 0.0420683 5.120e-04      5.120e-04
## ExpDay_factor13.units           0.8013372 0.0468903 5.707e-04      5.854e-04
## ExpDay_factor14.units           0.9150710 0.0537226 6.539e-04      6.539e-04
## ExpDay_factor15.units           1.0916964 0.0638862 7.776e-04      7.776e-04
## ExpDay_factor16.units           0.5811224 0.0347738 4.233e-04      4.233e-04
## ExpDay_factor17.units           0.6815942 0.0404589 4.924e-04      4.924e-04
## ExpDay_factor18.units           0.5078669 0.0311195 3.788e-04      3.788e-04
## ExpDay_factor19.units           0.8531521 0.0508495 6.189e-04      6.189e-04
## ExpDay_factor20.units           0.6408210 0.0389262 4.738e-04      4.738e-04
## ExpDay_factor21.units           0.6016139 0.0354133 4.310e-04      4.310e-04
## ExpDay_factor22.units           0.5330595 0.0317808 3.868e-04      3.868e-04
## ExpDay_factor23.units           0.6491661 0.0390390 4.752e-04      4.752e-04
## ExpDay_factor24.units           0.5187411 0.0314661 3.830e-04      3.927e-04
## ExpDay_factor25.units           0.8238656 0.0505819 6.157e-04      6.157e-04
## ExpDay_factor26.units           0.6331609 0.0386228 4.701e-04      4.701e-04
## ExpDay_factor27.units           0.5192809 0.0320061 3.896e-04      3.763e-04
##
## 2. Quantiles for each variable:
##
##                                       2.5%        25%        50%        75%
## (Intercept):(Intercept).FishId  1.160e-01  0.1541569  0.1798968  0.2104158
## ExpDay:(Intercept).FishId      -1.454e-02 -0.0101358 -0.0083870 -0.0069771
## (Intercept):ExpDay.FishId      -1.454e-02 -0.0101358 -0.0083870 -0.0069771
## ExpDay:ExpDay.FishId            4.788e-04  0.0006405  0.0007532  0.0008884
## (Intercept):(Intercept).MomId   8.258e-04  0.0296912  0.0673256  0.1294560
## ExpDay:(Intercept).MomId       -5.966e-03 -0.0004939  0.0007325  0.0021436
## (Intercept):ExpDay.MomId       -5.966e-03 -0.0004939  0.0007325  0.0021436
## ExpDay:ExpDay.MomId             8.438e-05  0.0003087  0.0005011  0.0007858
## ExpDay_factor0.units            2.493e+00  2.6856568  2.7945100  2.9103888
## ExpDay_factor1.units            9.341e-01  1.0075378  1.0477768  1.0881786
## ExpDay_factor2.units            6.165e-01  0.6645585  0.6923202  0.7203171
## ExpDay_factor3.units            4.706e-01  0.5084653  0.5305442  0.5527158
```

```
## ExpDay_factor4.units            4.167e-01  0.4494588  0.4669611  0.4869331
## ExpDay_factor5.units            3.764e-01  0.4058333  0.4221957  0.4399561
## ExpDay_factor6.units            6.699e-01  0.7211174  0.7517444  0.7847106
## ExpDay_factor7.units            6.538e-01  0.7037564  0.7325825  0.7611576
## ExpDay_factor8.units            7.308e-01  0.7868612  0.8185631  0.8519071
## ExpDay_factor9.units            5.988e-01  0.6452962  0.6714527  0.6988001
## ExpDay_factor10.units           5.267e-01  0.5713691  0.5961745  0.6221345
## ExpDay_factor11.units           4.580e-01  0.4942635  0.5150114  0.5369869
## ExpDay_factor12.units           6.126e-01  0.6604820  0.6882152  0.7172735
## ExpDay_factor13.units           7.161e-01  0.7685731  0.7997194  0.8322116
## ExpDay_factor14.units           8.159e-01  0.8776098  0.9122408  0.9495131
## ExpDay_factor15.units           9.740e-01  1.0486071  1.0888570  1.1321374
## ExpDay_factor16.units           5.181e-01  0.5569912  0.5795853  0.6036167
## ExpDay_factor17.units           6.073e-01  0.6535719  0.6799190  0.7075974
## ExpDay_factor18.units           4.492e-01  0.4863984  0.5065076  0.5274605
## ExpDay_factor19.units           7.596e-01  0.8178864  0.8519795  0.8865220
## ExpDay_factor20.units           5.690e-01  0.6141031  0.6391124  0.6664041
## ExpDay_factor21.units           5.371e-01  0.5773726  0.6002878  0.6242788
## ExpDay_factor22.units           4.753e-01  0.5107611  0.5319705  0.5534177
## ExpDay_factor23.units           5.789e-01  0.6213207  0.6471136  0.6750586
## ExpDay_factor24.units           4.606e-01  0.4971609  0.5175809  0.5388128
## ExpDay_factor25.units           7.314e-01  0.7881525  0.8218896  0.8562532
## ExpDay_factor26.units           5.609e-01  0.6066296  0.6316176  0.6576286
## ExpDay_factor27.units           4.594e-01  0.4971665  0.5177626  0.5403942
##                                     97.5%
## (Intercept):(Intercept).FishId  0.288100
## ExpDay:(Intercept).FishId      -0.004675
## (Intercept):ExpDay.FishId      -0.004675
## ExpDay:ExpDay.FishId            0.001243
## (Intercept):(Intercept).MomId   0.471902
## ExpDay:(Intercept).MomId        0.007372
## (Intercept):ExpDay.MomId        0.007372
## ExpDay:ExpDay.MomId             0.001783
## ExpDay_factor0.units            3.138880
## ExpDay_factor1.units            1.172611
## ExpDay_factor2.units            0.781122
## ExpDay_factor3.units            0.599643
## ExpDay_factor4.units            0.525962
## ExpDay_factor5.units            0.476712
## ExpDay_factor6.units            0.851156
## ExpDay_factor7.units            0.823465
## ExpDay_factor8.units            0.919230
## ExpDay_factor9.units            0.754042
## ExpDay_factor10.units           0.679417
## ExpDay_factor11.units           0.581520
## ExpDay_factor12.units           0.776764
## ExpDay_factor13.units           0.896656
## ExpDay_factor14.units           1.027253
## ExpDay_factor15.units           1.222904
## ExpDay_factor16.units           0.652287
## ExpDay_factor17.units           0.763959
## ExpDay_factor18.units           0.572273
## ExpDay_factor19.units           0.959137
## ExpDay_factor20.units           0.721132
## ExpDay_factor21.units           0.674952
## ExpDay_factor22.units           0.599126
## ExpDay_factor23.units           0.728555
## ExpDay_factor24.units           0.584518
## ExpDay_factor25.units           0.927377
## ExpDay_factor26.units           0.713142
## ExpDay_factor27.units           0.583951
```

```r
summary(model_predator$VCV)
```

```
##
## Iterations = 150001:1499801
## Thinning interval = 200
## Number of chains = 1
## Sample size per chain = 6750
##
## 1. Empirical mean and standard deviation for each variable,
##    plus standard error of the mean:
##
##                                    Mean        SD  Naive SE Time-series SE
## (Intercept):(Intercept).FishId  0.1509539 0.0386575 4.705e-04      4.973e-04
## ExpDay:(Intercept).FishId      -0.0051597 0.0019543 2.379e-05      2.770e-05
## (Intercept):ExpDay.FishId      -0.0051597 0.0019543 2.379e-05      2.770e-05
## ExpDay:ExpDay.FishId            0.0005888 0.0001626 1.979e-06      2.426e-06
## (Intercept):(Intercept).MomId   1.0617849 1.2393166 1.508e-02      8.259e-02
## ExpDay:(Intercept).MomId        0.0025468 0.0117280 1.427e-04      2.133e-04
## (Intercept):ExpDay.MomId        0.0025468 0.0117280 1.427e-04      2.133e-04
## ExpDay:ExpDay.MomId             0.0008198 0.0005643 6.868e-06      1.129e-05
## ExpDay_factor0.units            1.5627197 0.0921165 1.121e-03      1.184e-03
## ExpDay_factor1.units            1.0771794 0.0620518 7.553e-04      7.553e-04
## ExpDay_factor2.units            0.6557823 0.0390238 4.750e-04      4.750e-04
## ExpDay_factor3.units            0.7923262 0.0471303 5.737e-04      5.737e-04
## ExpDay_factor4.units            0.8929119 0.0518234 6.308e-04      6.308e-04
## ExpDay_factor5.units            0.7937552 0.0459311 5.591e-04      5.591e-04
## ExpDay_factor6.units            0.6342413 0.0371552 4.522e-04      4.367e-04
## ExpDay_factor7.units            0.5351418 0.0306660 3.733e-04      3.617e-04
## ExpDay_factor8.units            0.6528699 0.0373106 4.541e-04      4.541e-04
## ExpDay_factor9.units            0.7719612 0.0434044 5.283e-04      5.283e-04
## ExpDay_factor10.units           0.7534904 0.0422854 5.147e-04      5.147e-04
## ExpDay_factor11.units           0.5156865 0.0302278 3.679e-04      3.679e-04
## ExpDay_factor12.units           0.6117107 0.0345892 4.210e-04      4.210e-04
## ExpDay_factor13.units           0.6640788 0.0376642 4.584e-04      4.584e-04
## ExpDay_factor14.units           0.6014592 0.0341271 4.154e-04      4.154e-04
## ExpDay_factor15.units           0.6707512 0.0370733 4.512e-04      4.512e-04
## ExpDay_factor16.units           0.5789562 0.0328195 3.995e-04      3.995e-04
## ExpDay_factor17.units           0.5923169 0.0334612 4.073e-04      4.073e-04
## ExpDay_factor18.units           0.5466603 0.0309391 3.766e-04      3.766e-04
## ExpDay_factor19.units           0.7294691 0.0412413 5.020e-04      5.020e-04
## ExpDay_factor20.units           0.6284914 0.0350661 4.268e-04      4.268e-04
## ExpDay_factor21.units           0.6386008 0.0363660 4.426e-04      4.426e-04
## ExpDay_factor22.units           0.7780352 0.0436787 5.316e-04      5.316e-04
## ExpDay_factor23.units           0.7383611 0.0419802 5.110e-04      5.189e-04
## ExpDay_factor24.units           0.5806981 0.0335899 4.088e-04      4.088e-04
## ExpDay_factor25.units           0.6611801 0.0376962 4.588e-04      4.588e-04
## ExpDay_factor26.units           0.7100369 0.0415899 5.062e-04      5.062e-04
## ExpDay_factor27.units           0.6724205 0.0404059 4.918e-04      5.008e-04
##
## 2. Quantiles for each variable:
##
##                                    2.5%        25%        50%        75%
## (Intercept):(Intercept).FishId  0.0922258  0.1235712  0.1452868  0.1714387
## ExpDay:(Intercept).FishId      -0.0098106 -0.0062260 -0.0049186 -0.0037812
## (Intercept):ExpDay.FishId      -0.0098106 -0.0062260 -0.0049186 -0.0037812
## ExpDay:ExpDay.FishId            0.0003459  0.0004726  0.0005626  0.0006748
## (Intercept):(Intercept).MomId   0.0659647  0.2829445  0.6698726  1.3919765
## ExpDay:(Intercept).MomId       -0.0177203 -0.0031912  0.0010908  0.0070539
## (Intercept):ExpDay.MomId       -0.0177203 -0.0031912  0.0010908  0.0070539
## ExpDay:ExpDay.MomId             0.0001673  0.0004520  0.0006864  0.0010242
## ExpDay_factor0.units            1.3923907  1.4995961  1.5587421  1.6223241
```

```
## ExpDay_factor1.units            0.9618821  1.0338639  1.0744152  1.1182856
## ExpDay_factor2.units            0.5846484  0.6282044  0.6538371  0.6807903
## ExpDay_factor3.units            0.7067365  0.7594636  0.7902995  0.8232356
## ExpDay_factor4.units            0.7976578  0.8572529  0.8912767  0.9271112
## ExpDay_factor5.units            0.7090936  0.7613331  0.7917383  0.8243914
## ExpDay_factor6.units            0.5656154  0.6082378  0.6330040  0.6585517
## ExpDay_factor7.units            0.4780884  0.5142108  0.5339606  0.5551471
## ExpDay_factor8.units            0.5817075  0.6270669  0.6519843  0.6773921
## ExpDay_factor9.units            0.6913059  0.7418430  0.7706117  0.8009199
## ExpDay_factor10.units           0.6736299  0.7239606  0.7514428  0.7811788
## ExpDay_factor11.units           0.4596592  0.4945962  0.5144451  0.5355270
## ExpDay_factor12.units           0.5465349  0.5876268  0.6102273  0.6341904
## ExpDay_factor13.units           0.5946190  0.6378783  0.6627648  0.6888743
## ExpDay_factor14.units           0.5384701  0.5774513  0.6005077  0.6237178
## ExpDay_factor15.units           0.6016380  0.6451618  0.6695445  0.6946307
## ExpDay_factor16.units           0.5175041  0.5561674  0.5776377  0.6010365
## ExpDay_factor17.units           0.5308156  0.5685554  0.5916164  0.6146074
## ExpDay_factor18.units           0.4893113  0.5258125  0.5456009  0.5663266
## ExpDay_factor19.units           0.6525914  0.7007565  0.7277670  0.7561749
## ExpDay_factor20.units           0.5638559  0.6042950  0.6272386  0.6514195
## ExpDay_factor21.units           0.5714064  0.6135865  0.6375456  0.6613685
## ExpDay_factor22.units           0.6971800  0.7477247  0.7765390  0.8068435
## ExpDay_factor23.units           0.6619520  0.7095662  0.7366528  0.7653909
## ExpDay_factor24.units           0.5192318  0.5575794  0.5795609  0.6028088
## ExpDay_factor25.units           0.5902256  0.6355391  0.6604417  0.6853082
## ExpDay_factor26.units           0.6330332  0.6808110  0.7084453  0.7372786
## ExpDay_factor27.units           0.5969898  0.6444614  0.6710758  0.6986433
##                                     97.5%
## (Intercept):(Intercept).FishId  0.2444755
## ExpDay:(Intercept).FishId      -0.0020884
## (Intercept):ExpDay.FishId      -0.0020884
## ExpDay:ExpDay.FishId            0.0009745
## (Intercept):(Intercept).MomId   4.2884341
## ExpDay:(Intercept).MomId        0.0288529
## (Intercept):ExpDay.MomId        0.0288529
## ExpDay:ExpDay.MomId             0.0022763
## ExpDay_factor0.units            1.7523760
## ExpDay_factor1.units            1.2045318
## ExpDay_factor2.units            0.7384017
## ExpDay_factor3.units            0.8901282
## ExpDay_factor4.units            1.0004309
## ExpDay_factor5.units            0.8858085
## ExpDay_factor6.units            0.7100866
## ExpDay_factor7.units            0.5992123
## ExpDay_factor8.units            0.7283590
## ExpDay_factor9.units            0.8605915
## ExpDay_factor10.units           0.8394987
## ExpDay_factor11.units           0.5781649
## ExpDay_factor12.units           0.6818081
## ExpDay_factor13.units           0.7406077
## ExpDay_factor14.units           0.6700350
## ExpDay_factor15.units           0.7474062
## ExpDay_factor16.units           0.6452807
## ExpDay_factor17.units           0.6609495
## ExpDay_factor18.units           0.6110510
## ExpDay_factor19.units           0.8152558
## ExpDay_factor20.units           0.7012408
## ExpDay_factor21.units           0.7147946
## ExpDay_factor22.units           0.8687329
## ExpDay_factor23.units           0.8257061
## ExpDay_factor24.units           0.6489190
```

```
## ExpDay_factor25.units           0.7382107
## ExpDay_factor26.units           0.7962511
## ExpDay_factor27.units           0.7559560
```

> Investigating variance components between treatments

```
# Control

sigma.a0 <- model_control$VCV[, "(Intercept):(Intercept).FishId"]
sigma.a1 <- model_control$VCV[, "ExpDay:ExpDay.FishId"]
cov <- model_control$VCV[, "(Intercept):ExpDay.FishId"]

mom_intercept <- model_control$VCV[, "(Intercept):(Intercept).MomId"]
mom_slope <- model_control$VCV[, "ExpDay:ExpDay.MomId"]
mom_cov <- model_control$VCV[, "(Intercept):ExpDay.MomId"]

# Loop to extract variance components per day
for (day in 0:27) {
    x_i <- day
    sigma.e <- model_control$VCV[, paste0("ExpDay_factor", day,
        ".units")]
    rep <- (sigma.a0 + sigma.a1 * x_i^2 + 2 * cov * x_i)/(sigma.a0 +
        sigma.a1 * x_i^2 + 2 * cov * x_i + sigma.e)
    rep_control <- median(rep)
    rep_control_CI <- HPDinterval(as.mcmc(rep))
    rep_with_mom <- (sigma.a0 + sigma.a1 * x_i^2 + 2 * cov *
        x_i + mom_intercept + mom_slope * x_i^2 + 2 * mom_cov *
        x_i)/(sigma.a0 + sigma.a1 * x_i^2 + 2 * cov * x_i + mom_intercept +
        mom_slope * x_i^2 + 2 * mom_cov * x_i + sigma.e)
    rep_with_mom_control <- median(rep_with_mom)
    rep_with_mom_control_CI <- HPDinterval(as.mcmc(rep_with_mom))
    among_val <- sigma.a0 + (sigma.a1 * x_i^2 + 2 * cov * x_i)
    among_control <- median(among_val)
    among_control_CI <- HPDinterval(as.mcmc(among_val))
    within_control <- median(sigma.e)
    within_control_CI <- HPDinterval(as.mcmc(sigma.e))
    mom_val <- mom_intercept + (mom_slope * x_i^2 + 2 * mom_cov *
        x_i)
    mom_control <- median(mom_val)
    mom_control_CI <- HPDinterval(as.mcmc(mom_val))
    mom_rep <- (mom_intercept + (mom_slope * x_i^2 + 2 * mom_cov *
        x_i))/((mom_intercept + (mom_slope * x_i^2 + 2 * mom_cov *
        x_i)) + (sigma.a0 + sigma.a1 * x_i^2 + 2 * cov * x_i +
        mom_intercept + mom_slope * x_i^2 + 2 * mom_cov * x_i +
        sigma.e))
    mom_rep_control <- median(mom_rep)
    mom_rep_control_CI <- HPDinterval(as.mcmc(mom_rep))
    assign(paste0("rep_control_day", day), rep_control)
    assign(paste0("rep_control_CI_day", day), rep_control_CI)
    assign(paste0("rep_with_mom_control_day", day), rep_with_mom_control)
    assign(paste0("rep_with_mom_control_CI_day", day), rep_with_mom_control_CI)
    assign(paste0("among_control_day", day), among_control)
    assign(paste0("among_control_CI_day", day), among_control_CI)
    assign(paste0("within_control_day", day), within_control)
    assign(paste0("within_control_CI_day", day), within_control_CI)
    assign(paste0("mom_control_day", day), mom_control)
    assign(paste0("mom_control_CI_day", day), mom_control_CI)
    assign(paste0("mom_rep_control_day", day), mom_rep_control)
    assign(paste0("mom_rep_control_CI_day", day), mom_rep_control_CI)
}
```

```r
# Predator

sigma.a0 <- model_predator$VCV[, "(Intercept):(Intercept).FishId"]
sigma.a1 <- model_predator$VCV[, "ExpDay:ExpDay.FishId"]
cov <- model_predator$VCV[, "(Intercept):ExpDay.FishId"]

mom_intercept <- model_predator$VCV[, "(Intercept):(Intercept).MomId"]
mom_slope <- model_predator$VCV[, "ExpDay:ExpDay.MomId"]
mom_cov <- model_predator$VCV[, "(Intercept):ExpDay.MomId"]

# Loop to extract variance components per day
for (day in 0:27) {
    x_i <- day
    sigma.e <- model_predator$VCV[, paste0("ExpDay_factor", day,
        ".units")]
    rep <- (sigma.a0 + sigma.a1 * x_i^2 + 2 * cov * x_i)/(sigma.a0 +
        sigma.a1 * x_i^2 + 2 * cov * x_i + sigma.e)
    rep_predator <- median(rep)
    rep_predator_CI <- HPDinterval(as.mcmc(rep))
    rep_with_mom <- (sigma.a0 + sigma.a1 * x_i^2 + 2 * cov *
        x_i + mom_intercept + mom_slope * x_i^2 + 2 * mom_cov *
        x_i)/(sigma.a0 + sigma.a1 * x_i^2 + 2 * cov * x_i + mom_intercept +
        mom_slope * x_i^2 + 2 * mom_cov * x_i + sigma.e)
    rep_with_mom_predator <- median(rep_with_mom)
    rep_with_mom_predator_CI <- HPDinterval(as.mcmc(rep_with_mom))
    among_val <- sigma.a0 + (sigma.a1 * x_i^2 + 2 * cov * x_i)
    among_predator <- median(among_val)
    among_predator_CI <- HPDinterval(as.mcmc(among_val))
    within_predator <- median(sigma.e)
    within_predator_CI <- HPDinterval(as.mcmc(sigma.e))
    mom_val <- mom_intercept + (mom_slope * x_i^2 + 2 * mom_cov *
        x_i)
    mom_predator <- median(mom_val)
    mom_predator_CI <- HPDinterval(as.mcmc(mom_val))
    mom_rep <- (mom_intercept + (mom_slope * x_i^2 + 2 * mom_cov *
        x_i))/((mom_intercept + (mom_slope * x_i^2 + 2 * mom_cov *
        x_i)) + (sigma.a0 + sigma.a1 * x_i^2 + 2 * cov * x_i +
        mom_intercept + mom_slope * x_i^2 + 2 * mom_cov * x_i +
        sigma.e))
    mom_rep_predator <- median(mom_rep)
    mom_rep_predator_CI <- HPDinterval(as.mcmc(mom_rep))
    assign(paste0("rep_predator_day", day), rep_predator)
    assign(paste0("rep_predator_CI_day", day), rep_predator_CI)
    assign(paste0("rep_with_mom_predator_day", day), rep_with_mom_predator)
    assign(paste0("rep_with_mom_predator_CI_day", day), rep_with_mom_predator_CI)
    assign(paste0("among_predator_day", day), among_predator)
    assign(paste0("among_predator_CI_day", day), among_predator_CI)
    assign(paste0("within_predator_day", day), within_predator)
    assign(paste0("within_predator_CI_day", day), within_predator_CI)
    assign(paste0("mom_predator_day", day), mom_predator)
    assign(paste0("mom_predator_CI_day", day), mom_predator_CI)
    assign(paste0("mom_rep_predator_day", day), mom_rep_predator)
    assign(paste0("mom_rep_predator_CI_day", day), mom_rep_predator_CI)
}

### Plots

## Repeatability - no mom

# Define colors for plotting:
colors <- c(Control = "#1709b5", Predator = "#ed5715")
```

```r
variance_components_table <- data.frame(Day = integer(), Repeatability = numeric(),
    RepeatabilityLowerCI = numeric(), RepeatabilityUpperCI = numeric(),
    Treatment = character(), stringsAsFactors = FALSE)

# Loop from day 0 through 27
for (day in 0:27) {
    # Control
    rep_ctrl_name <- paste0("rep_control_day", day)
    rep_ctrl_ci_name <- paste0("rep_control_CI_day", day)
    rep_ctrl_val <- get(rep_ctrl_name)
    rep_ctrl_CI <- get(rep_ctrl_ci_name)
    row_ctrl <- data.frame(Day = day, Repeatability = rep_ctrl_val,
        RepeatabilityLowerCI = rep_ctrl_CI[1], RepeatabilityUpperCI = rep_ctrl_CI[2],
        Treatment = "Control")
    variance_components_table <- rbind(variance_components_table,
        row_ctrl)

    # Pred
    rep_pred_name <- paste0("rep_predator_day", day)
    rep_pred_ci_name <- paste0("rep_predator_CI_day", day)
    rep_pred_val <- get(rep_pred_name)
    rep_pred_CI <- get(rep_pred_ci_name)
    row_pred <- data.frame(Day = day, Repeatability = rep_pred_val,
        RepeatabilityLowerCI = rep_pred_CI[1], RepeatabilityUpperCI = rep_pred_CI[2],
        Treatment = "Predator")
    variance_components_table <- rbind(variance_components_table,
        row_pred)
}

plot_name <- ggplot(variance_components_table, aes(x = Day +
    1, y = Repeatability, color = Treatment)) + geom_line(show.legend = FALSE) +
    geom_point(size = 2, show.legend = FALSE) + geom_errorbar(aes(ymin = RepeatabilityLowerCI,
    ymax = RepeatabilityUpperCI), width = 0.5, show.legend = FALSE) +
    scale_x_continuous(limits = c(0.5, 28.5), breaks = seq(1,
        29, by = 3)) + scale_y_continuous(limits = c(0, 0.7),
    breaks = seq(0, 0.7, by = 0.1)) + scale_color_manual(values = colors) +
    labs(x = "Day", y = "Repeatability") + theme_classic()
plot_name
```

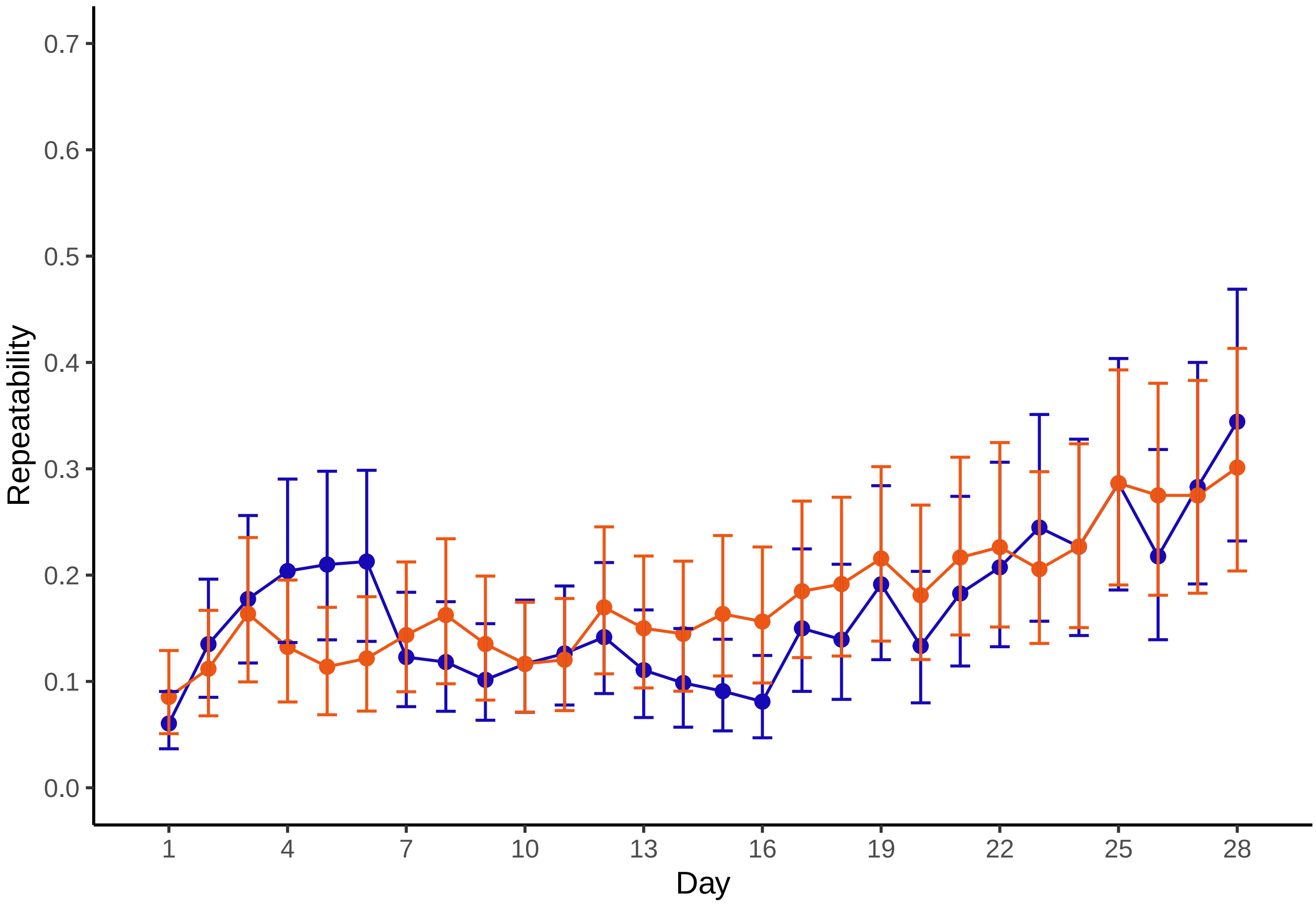


```
ggsave("repeatability_velocity_hour.pdf", plot = plot_name, dpi = 600)
```

```
## Saving 6.5 x 4.5 in image
```

```
## Repeatability - with mom

# Define colors for plotting:
colors <- c(Control = "#1709b5", Predator = "#ed5715")

variance_components_table <- data.frame(Day = integer(), Repeatability = numeric(),
    RepeatabilityLowerCI = numeric(), RepeatabilityUpperCI = numeric(),
    Treatment = character(), stringsAsFactors = FALSE)

# Loop from day 0 through 27
for (day in 0:27) {
    # Control
    rep_ctrl_name <- paste0("rep_with_mom_control_day", day)
    rep_ctrl_ci_name <- paste0("rep_with_mom_control_CI_day",
        day)
    rep_ctrl_val <- get(rep_ctrl_name)
    rep_ctrl_CI <- get(rep_ctrl_ci_name)
    row_ctrl <- data.frame(Day = day, Repeatability = rep_ctrl_val,
        RepeatabilityLowerCI = rep_ctrl_CI[1], RepeatabilityUpperCI = rep_ctrl_CI[2],
        Treatment = "Control")
    variance_components_table <- rbind(variance_components_table,
        row_ctrl)

    # Pred
    rep_pred_name <- paste0("rep_with_mom_predator_day", day)
```

```
    rep_pred_ci_name <- paste0("rep_with_mom_predator_CI_day",
        day)
    rep_pred_val <- get(rep_pred_name)
    rep_pred_CI <- get(rep_pred_ci_name)
    row_pred <- data.frame(Day = day, Repeatability = rep_pred_val,
        RepeatabilityLowerCI = rep_pred_CI[1], RepeatabilityUpperCI = rep_pred_CI[2],
        Treatment = "Predator")
    variance_components_table <- rbind(variance_components_table,
        row_pred)
}

plot_name <- ggplot(variance_components_table, aes(x = Day +
    1, y = Repeatability, color = Treatment)) + geom_line(show.legend = FALSE) +
    geom_point(size = 2, show.legend = FALSE) + geom_errorbar(aes(ymin = RepeatabilityLowerCI,
    ymax = RepeatabilityUpperCI), width = 0.5, show.legend = FALSE) +
    scale_x_continuous(limits = c(0.5, 28.5), breaks = seq(1,
        29, by = 3)) + scale_y_continuous(limits = c(0, 1), breaks = seq(0,
    1, by = 0.1)) + scale_color_manual(values = colors) + labs(x = "Day",
    y = "Repeatability") + theme_classic()
plot_name
```

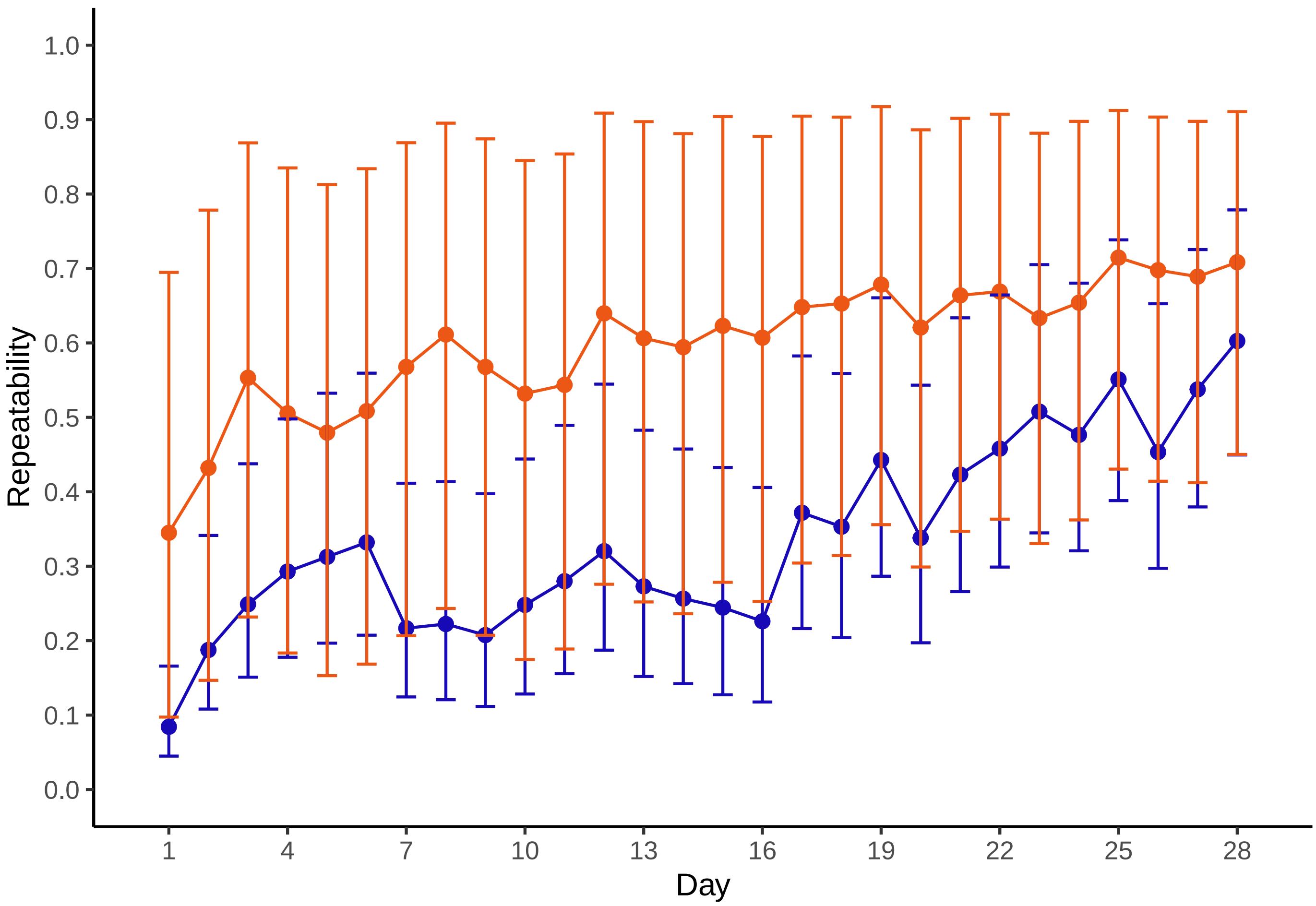


```
ggsave("repeatability_with_mom_velocity_hour.pdf", plot = plot_name,
    dpi = 600)
```

```
## Saving 6.5 x 4.5 in image
```

```
## Among-individual variance

# Define colors for plotting:
```

```r
colors <- c(Control = "#1709b5", Predator = "#ed5715")

variance_components_table <- data.frame(Day = integer(), Among = numeric(),
    AmongLowerCI = numeric(), AmongUpperCI = numeric(), Treatment = character(),
    stringsAsFactors = FALSE)

# Loop from day 0 through 27
for (day in 0:27) {
    # Control
    among_ctrl_name <- paste0("among_control_day", day)
    among_ctrl_ci_name <- paste0("among_control_CI_day", day)
    among_ctrl_val <- get(among_ctrl_name)
    among_ctrl_CI <- get(among_ctrl_ci_name)
    row_ctrl <- data.frame(Day = day, Among = among_ctrl_val,
        AmongLowerCI = among_ctrl_CI[1], AmongUpperCI = among_ctrl_CI[2],
        Treatment = "Control")
    variance_components_table <- rbind(variance_components_table,
        row_ctrl)

    # Pred
    among_pred_name <- paste0("among_predator_day", day)
    among_pred_ci_name <- paste0("among_predator_CI_day", day)
    among_pred_val <- get(among_pred_name)
    among_pred_CI <- get(among_pred_ci_name)
    row_pred <- data.frame(Day = day, Among = among_pred_val,
        AmongLowerCI = among_pred_CI[1], AmongUpperCI = among_pred_CI[2],
        Treatment = "Predator")
    variance_components_table <- rbind(variance_components_table,
        row_pred)
}

plot_name <- ggplot(variance_components_table, aes(x = Day +
    1, y = Among, color = Treatment)) + geom_line(show.legend = FALSE) +
    geom_errorbar(aes(ymin = AmongLowerCI, ymax = AmongUpperCI),
        width = 0.5, show.legend = FALSE) + geom_point(size = 2.5,
    shape = 24, fill = "white", show.legend = FALSE) + scale_x_continuous(limits = c(0.5,
    28.5), breaks = seq(1, 29, by = 3)) + scale_y_continuous(limits = c(0,
    3.5), breaks = seq(0, 3.5, by = 0.5)) + scale_color_manual(values = colors) +
    labs(x = "Day", y = "Among-individual variance") + theme_classic()
plot_name
```

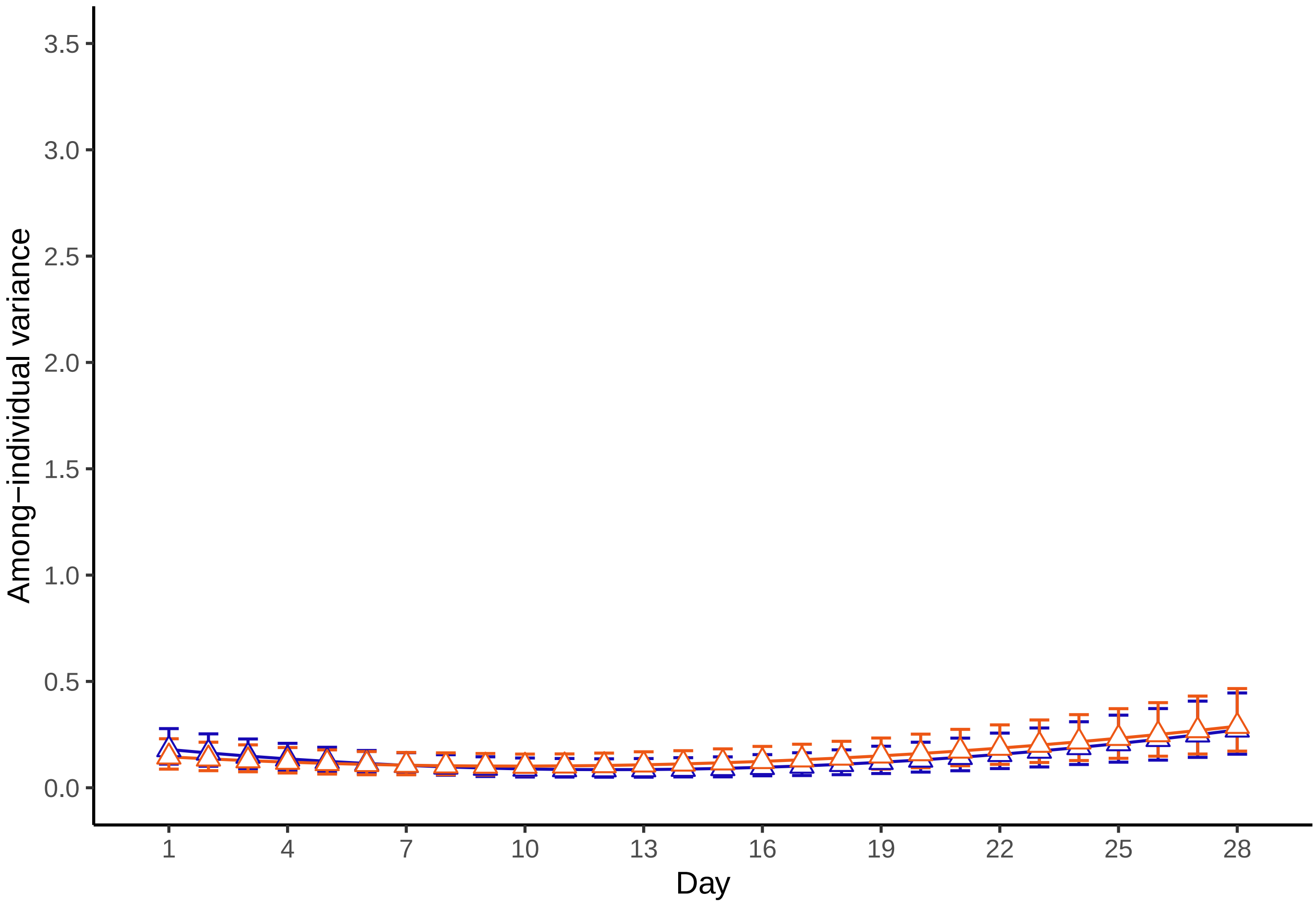


```
ggsave("among_velocity_hour.pdf", plot = plot_name, dpi = 600)
```

```
## Saving 6.5 x 4.5 in image
```

```
## Within-individual variance

# Define colors for plotting:
colors <- c(Control = "#1709b5", Predator = "#ed5715")

variance_components_table <- data.frame(Day = integer(), Within = numeric(),
    WithinLowerCI = numeric(), WithinUpperCI = numeric(), Treatment = character(),
    stringsAsFactors = FALSE)

# Loop from day 0 through 27
for (day in 0:27) {
    # Control
    within_ctrl_name <- paste0("within_control_day", day)
    within_ctrl_ci_name <- paste0("within_control_CI_day", day)
    within_ctrl_val <- get(within_ctrl_name)
    within_ctrl_CI <- get(within_ctrl_ci_name)
    row_ctrl <- data.frame(Day = day, Within = within_ctrl_val,
        WithinLowerCI = within_ctrl_CI[1], WithinUpperCI = within_ctrl_CI[2],
        Treatment = "Control")
    variance_components_table <- rbind(variance_components_table,
        row_ctrl)

    # Pred
    within_ctrl_name <- paste0("within_predator_day", day)
    within_ctrl_ci_name <- paste0("within_predator_CI_day", day)
```

```
    within_ctrl_val <- get(within_ctrl_name)
    within_ctrl_CI <- get(within_ctrl_ci_name)
    row_ctrl <- data.frame(Day = day, Within = within_ctrl_val,
        WithinLowerCI = within_ctrl_CI[1], WithinUpperCI = within_ctrl_CI[2],
        Treatment = "Predator")
    variance_components_table <- rbind(variance_components_table,
        row_ctrl)
}

plot_name <- ggplot(variance_components_table, aes(x = Day +
    1, y = Within, color = Treatment)) + geom_line(show.legend = FALSE) +
    geom_point(size = 2, show.legend = FALSE) + geom_errorbar(aes(ymin = WithinLowerCI,
    ymax = WithinUpperCI), width = 0.5, show.legend = FALSE) +
    scale_x_continuous(limits = c(0.5, 28.5), breaks = seq(1,
        29, by = 3)) + scale_y_continuous(limits = c(0, 3.5),
    breaks = seq(0, 3.5, by = 0.5)) + scale_color_manual(values = colors) +
    labs(x = "Day", y = "Within-individual variance") + theme_classic()
plot_name
```

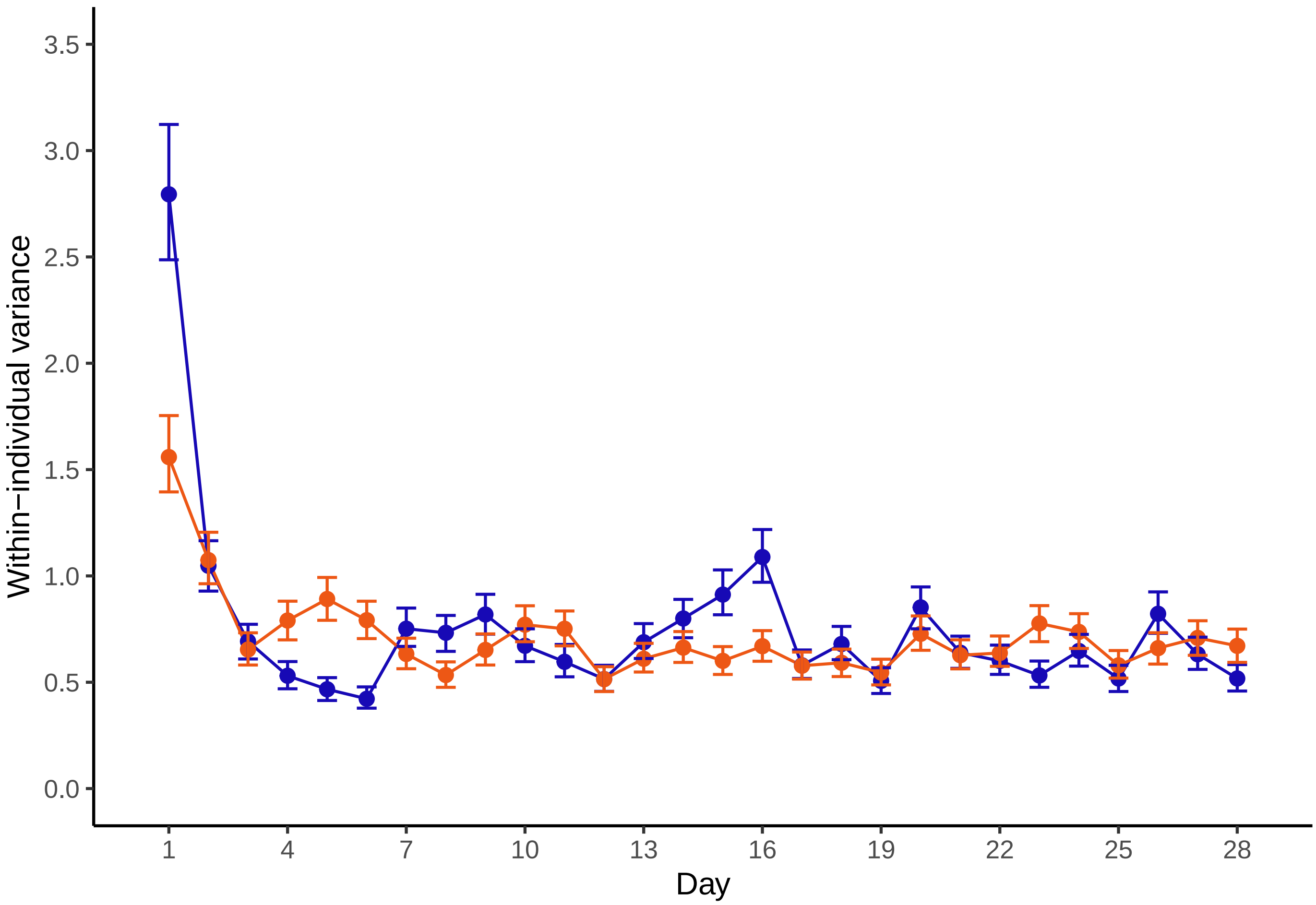


```
ggsave("within_velocity_hour.pdf", plot = plot_name, dpi = 600)
```

```
## Saving 6.5 x 4.5 in image
```

Refuge use (MeanCorner)

Mean effect of treatment

Model comparison to determine model structure

We will conduct model comparison to determine the best fit random effect structure. First we will set priors.

Prepare data for bivariate model

We need to create columns for control and predator treatments:

```
hourly_data$MeanCorner_control <- ifelse(hourly_data$PredatorCue ==
    0, hourly_data$MeanCorner_hourly_scaled, NA)
hourly_data$MeanCorner_predator <- ifelse(hourly_data$PredatorCue ==
    1, hourly_data$MeanCorner_hourly_scaled, NA)
```

Set priors

Now we need to set expanded priors for all of our models:

```
# Null
prior.null <- list(R = list(V = diag(2), nu = 0.002))

# FishId
prior.FishId <- list(R = list(V = diag(2), nu = 0.002), G = list(G1 = list(V = diag(2),
    nu = 0.002, alpha.mu = rep(0, 2), alpha.V = diag(25^2, 2))))

# MomId
prior.MomId <- list(R = list(V = diag(2), nu = 0.002), G = list(G1 = list(V = 1,
    nu = 0.002, alpha.mu = 0, alpha.V = 25^2)))

# FishId + MomId
prior.FishId_MomId <- list(R = list(V = diag(2), nu = 0.002),
    G = list(G1 = list(V = diag(2), nu = 0.002), G2 = list(V = 1,
        nu = 0.002)))

# FishId + MomId independent variance across treatments (no
# covariance)
prior.FishId_MomId_idh <- list(R = list(V = diag(2), nu = 0.002),
    G = list(G1 = list(V = diag(2), nu = 0.002), G2 = list(V = diag(2),
        nu = 0.002)))

# FishId + MomId unstructured (covariance)
prior.FishId_MomId_unstructured_mom <- list(R = list(V = diag(2),
    nu = 0.002), G = list(G1 = list(V = diag(2), nu = 0.002),
    G2 = list(V = diag(2), nu = 2)))

# FishId shared variance across treatments + MomId
prior.FishId_MomId_NOidh <- list(R = list(V = diag(2), nu = 0.002),
    G = list(G1 = list(V = 1, nu = 0.002), G2 = list(V = 1, nu = 0.002)))
```

Model comparison to determine included random effects

```
# Null
model_null <- readRDS("model_MeanCorner_null_8_14_25.rds")
# model_null <- MCMCglmm(cbind(MeanCorner_control,
# MeanCorner_predator) ~ trait*ExpDay - 1 + YearBatch +
# Hour, rcov = ~idh(trait):units, family = c('gaussian',
# 'gaussian'), data = hourly_data, prior = prior.null, nitt
# = 200000, burnin = 20000, thin = 100, verbose = TRUE)
```

```r
# saveRDS(model_null, file =
# 'model_MeanCorner_null_8_14_25.rds')
summary(model_null)
```

```
##
##  Iterations = 20001:199901
##  Thinning interval  = 100
##  Sample size  = 1800
##
##  DIC: 91143.25
##
##  R-structure:  ~idh(trait):units
##
##                                  post.mean l-95% CI u-95% CI eff.samp
## traitMeanCorner_control.units       0.834   0.8157   0.8524     1800
## traitMeanCorner_predator.units      1.088   1.0651   1.1126     1800
##
##  Location effects: cbind(MeanCorner_control, MeanCorner_predator) ~ trait * ExpDay - 1 + YearBatch + Hour
##
##                                   post.mean  l-95% CI  u-95% CI eff.samp  pMCMC
## traitMeanCorner_control            0.129468  0.091144  0.166944     1800 <6e-04
## traitMeanCorner_predator           0.349567  0.306946  0.388706     1956 <6e-04
## ExpDay                            -0.008982 -0.010874 -0.007322     1800 <6e-04
## YearBatch                         -0.043561 -0.053072 -0.033874     1800 <6e-04
## Hour                              -0.013599 -0.016557 -0.010423     1729 <6e-04
## traitMeanCorner_predator:ExpDay    0.007982  0.005478  0.010626     1800 <6e-04
##
## traitMeanCorner_control          ***
## traitMeanCorner_predator         ***
## ExpDay                           ***
## YearBatch                        ***
## Hour                             ***
## traitMeanCorner_predator:ExpDay ***
## ---
## Signif. codes:  0 '***' 0.001 '**' 0.01 '*' 0.05 '.' 0.1 ' ' 1
```

```r
# FishId
model_id <- readRDS("model_MeanCorner_id_8_14_25.rds")
# model_id <- MCMCglmm(cbind(MeanCorner_control,
# MeanCorner_predator) ~ trait*ExpDay - 1 + YearBatch +
# Hour, random = ~idh(trait):FishId, rcov =
# ~idh(trait):units, family = c('gaussian', 'gaussian'),
# data = hourly_data, prior = prior.FishId, nitt = 500000,
# burnin = 50000, thin = 100, verbose = TRUE)
# saveRDS(model_id, file =
# 'model_MeanCorner_id_8_14_25.rds')
summary(model_id)  # Best fit model, but we want to include MomId for our research question
```

```
##
##  Iterations = 50001:499901
##  Thinning interval  = 100
##  Sample size  = 4500
##
##  DIC: 82403.15
##
##  G-structure:  ~idh(trait):FishId
##
##                                   post.mean l-95% CI u-95% CI eff.samp
## traitMeanCorner_control.FishId      0.2232   0.1423   0.3154     2495
## traitMeanCorner_predator.FishId     0.2523   0.1634   0.3557     2710
##
##  R-structure:  ~idh(trait):units
```

```
##
##                                post.mean l-95% CI u-95% CI eff.samp
## traitMeanCorner_control.units     0.6103   0.5971   0.6241     4581
## traitMeanCorner_predator.units    0.8606   0.8427   0.8797     4715
##
##  Location effects: cbind(MeanCorner_control, MeanCorner_predator) ~ trait * ExpDay - 1 + YearBatch + Hour
##
##                                 post.mean  l-95% CI  u-95% CI eff.samp  pMCMC
## traitMeanCorner_control          0.135624 -0.072169  0.382874     1983  0.236
## traitMeanCorner_predator         0.353741  0.128790  0.602028     1998  0.004
## ExpDay                          -0.008176 -0.009739 -0.006722     5028 <2e-04
## YearBatch                       -0.046087 -0.124021  0.030934     2140  0.242
## Hour                            -0.013592 -0.016184 -0.010890     4500 <2e-04
## traitMeanCorner_predator:ExpDay  0.006510  0.004236  0.008832     4500 <2e-04
##
## traitMeanCorner_control
## traitMeanCorner_predator        **
## ExpDay                          ***
## YearBatch
## Hour                            ***
## traitMeanCorner_predator:ExpDay ***
## ---
## Signif. codes:  0 '***' 0.001 '**' 0.01 '*' 0.05 '.' 0.1 ' ' 1
```

```r
# MomId
model_mom <- readRDS("model_MeanCorner_mom_8_14_25.rds")
# model_mom <- MCMCglmm(cbind(MeanCorner_control,
# MeanCorner_predator) ~ trait*ExpDay - 1 + YearBatch +
# Hour, random = ~MomId, rcov = ~idh(trait):units, family =
# c('gaussian', 'gaussian'), data = hourly_data, prior =
# prior.MomId, nitt = 300000, burnin = 30000, thin = 100,
# verbose = TRUE) saveRDS(model_id, file =
# 'model_MeanCorner_mom_8_14_25.rds')
summary(model_mom)
```

```
##
##  Iterations = 50001:499901
##  Thinning interval  = 100
##  Sample size  = 4500
##
##  DIC: 82403.15
##
##  G-structure:  ~idh(trait):FishId
##
##                                 post.mean l-95% CI u-95% CI eff.samp
## traitMeanCorner_control.FishId     0.2232   0.1423   0.3154     2495
## traitMeanCorner_predator.FishId    0.2523   0.1634   0.3557     2710
##
##  R-structure:  ~idh(trait):units
##
##                                post.mean l-95% CI u-95% CI eff.samp
## traitMeanCorner_control.units     0.6103   0.5971   0.6241     4581
## traitMeanCorner_predator.units    0.8606   0.8427   0.8797     4715
##
##  Location effects: cbind(MeanCorner_control, MeanCorner_predator) ~ trait * ExpDay - 1 + YearBatch + Hour
##
##                                 post.mean  l-95% CI  u-95% CI eff.samp  pMCMC
## traitMeanCorner_control          0.135624 -0.072169  0.382874     1983  0.236
## traitMeanCorner_predator         0.353741  0.128790  0.602028     1998  0.004
## ExpDay                          -0.008176 -0.009739 -0.006722     5028 <2e-04
## YearBatch                       -0.046087 -0.124021  0.030934     2140  0.242
## Hour                            -0.013592 -0.016184 -0.010890     4500 <2e-04
```

```
## traitMeanCorner_predator:ExpDay  0.006510  0.004236  0.008832     4500 <2e-04
##
## traitMeanCorner_control
## traitMeanCorner_predator        **
## ExpDay                          ***
## YearBatch
## Hour                            ***
## traitMeanCorner_predator:ExpDay ***
## ---
## Signif. codes:  0 '***' 0.001 '**' 0.01 '*' 0.05 '.' 0.1 ' ' 1
```

```
# FishId + MomId
model_id_mom <- readRDS("model_MeanCorner_id_mom_8_14_25.rds")
# model_id_mom <- MCMCglmm(cbind(MeanCorner_control,
# MeanCorner_predator) ~ trait*ExpDay - 1 + YearBatch +
# Hour, random = ~idh(trait):FishId + MomId, rcov =
# ~idh(trait):units, family = c('gaussian', 'gaussian'),
# data = hourly_data, prior = prior.FishId_MomId, nitt =
# 1000000, burnin = 100000, thin = 100, verbose = TRUE)
# saveRDS(model_id_mom, file =
# 'model_MeanCorner_id_mom_8_14_25.rds')
summary(model_id_mom)  # Best fit model that includes MomId
```

```
##
##  Iterations = 100001:999901
##  Thinning interval  = 100
##  Sample size  = 9000
##
##  DIC: 82403.07
##
##  G-structure:  ~idh(trait):FishId
##
##                                 post.mean l-95% CI u-95% CI eff.samp
## traitMeanCorner_control.FishId     0.2134   0.1345   0.3059     5132
## traitMeanCorner_predator.FishId    0.2297   0.1414   0.3250     5504
##
##                ~MomId
##
##       post.mean  l-95% CI u-95% CI eff.samp
## MomId    0.0223 0.0002022  0.07108     4662
##
##  R-structure:  ~idh(trait):units
##
##                                post.mean l-95% CI u-95% CI eff.samp
## traitMeanCorner_control.units     0.6101   0.5969   0.6239     9000
## traitMeanCorner_predator.units    0.8607   0.8425   0.8791     9000
##
##  Location effects: cbind(MeanCorner_control, MeanCorner_predator) ~ trait * ExpDay - 1 + YearBatch + Hour
##
##                                 post.mean  l-95% CI  u-95% CI eff.samp  pMCMC
## traitMeanCorner_control          0.179065 -0.096973  0.448510     4997  0.180
## traitMeanCorner_predator         0.394583  0.124455  0.669724     5203  0.004
## ExpDay                          -0.008172 -0.009657 -0.006574     9000 <1e-04
## YearBatch                       -0.061354 -0.158718  0.032608     5328  0.192
## Hour                            -0.013597 -0.016260 -0.010885     9389 <1e-04
## traitMeanCorner_predator:ExpDay  0.006513  0.004284  0.008957     9000 <1e-04
##
## traitMeanCorner_control
## traitMeanCorner_predator        **
## ExpDay                          ***
## YearBatch
## Hour                            ***
```

```
## traitMeanCorner_predator:ExpDay ***
## ---
## Signif. codes:  0 '***' 0.001 '**' 0.01 '*' 0.05 '.' 0.1 ' ' 1
```

```
# FishId + MomId independent variance across treatments (no
# covariance)
model_id_mom_idh <- readRDS("model_MeanCorner_id_mom_slopes_8_14_25.rds")
# model_id_mom_idh <- MCMCglmm(cbind(MeanCorner_control,
# MeanCorner_predator) ~ trait*ExpDay - 1 + YearBatch +
# Hour, random = ~idh(trait):FishId + idh(trait):MomId,
# rcov = ~idh(trait):units, family = c('gaussian',
# 'gaussian'), data = hourly_data, prior =
# prior.FishId_MomId_idh, nitt = 1000000, burnin = 100000,
# thin = 100, verbose = TRUE) saveRDS(model_id_mom_idh,
# file = 'model_MeanCorner_id_mom_slopes_8_14_25.rds')
summary(model_id_mom_idh)  # Best fit that includes MomId
```

```
##
##  Iterations = 100001:999901
##  Thinning interval  = 100
##  Sample size  = 9000
##
##  DIC: 82403.16
##
##  G-structure:  ~idh(trait):FishId
##
##                                  post.mean l-95% CI u-95% CI eff.samp
## traitMeanCorner_control.FishId     0.2194   0.1386   0.3126     4614
## traitMeanCorner_predator.FishId    0.2348   0.1448   0.3331     5160
##
##                ~idh(trait):MomId
##
##                                 post.mean  l-95% CI u-95% CI eff.samp
## traitMeanCorner_control.MomId    0.01183 0.0001635  0.04431     6999
## traitMeanCorner_predator.MomId   0.02297 0.0001775  0.08198     6215
##
##  R-structure:  ~idh(trait):units
##
##                                 post.mean l-95% CI u-95% CI eff.samp
## traitMeanCorner_control.units     0.6102   0.5968   0.6236     8703
## traitMeanCorner_predator.units    0.8609   0.8431   0.8794     9000
##
##  Location effects: cbind(MeanCorner_control, MeanCorner_predator) ~ trait * ExpDay - 1 + YearBatch + Hour
##
##                                  post.mean  l-95% CI  u-95% CI eff.samp   pMCMC
## traitMeanCorner_control           0.157569 -0.097579  0.404046     4092 0.21133
## traitMeanCorner_predator          0.381471  0.122217  0.631619     4744 0.00267
## ExpDay                           -0.008176 -0.009747 -0.006675     9000 < 1e-04
## YearBatch                        -0.055346 -0.139178  0.032384     4490 0.20400
## Hour                             -0.013606 -0.016239 -0.010979     9957 < 1e-04
## traitMeanCorner_predator:ExpDay  0.006512  0.004169  0.008802     9000 < 1e-04
##
## traitMeanCorner_control
## traitMeanCorner_predator        **
## ExpDay                          ***
## YearBatch
## Hour                            ***
## traitMeanCorner_predator:ExpDay ***
## ---
## Signif. codes:  0 '***' 0.001 '**' 0.01 '*' 0.05 '.' 0.1 ' ' 1
```

```r
# # FishId + MomId unstructured (covariance)
model_id_mom_us <- readRDS("model_MeanCorner_id_mom_slopes_9_11_25.rds")
# model_id_mom_us <- MCMCglmm(cbind(MeanCorner_control,
# MeanCorner_predator) ~ trait*ExpDay - 1 + YearBatch +
# Hour, random = ~idh(trait):FishId + us(trait):MomId, rcov
# = ~idh(trait):units, family = c('gaussian', 'gaussian'),
# data = hourly_data, prior =
# prior.FishId_MomId_unstructured_mom, nitt = 1500000,
# burnin = 150000, thin = 100, verbose = TRUE)
# saveRDS(model_id_mom_us, file =
# 'model_MeanCorner_id_mom_slopes_9_11_25.rds')
summary(model_id_mom_us)
```

```
##
##  Iterations = 150001:1499901
##  Thinning interval  = 100
##  Sample size  = 13500
##
##  DIC: 82403.49
##
##  G-structure:  ~idh(trait):FishId
##
##                                   post.mean l-95% CI u-95% CI eff.samp
## traitMeanCorner_control.FishId      0.2282   0.1398   0.3339     5372
## traitMeanCorner_predator.FishId     0.2233   0.1353   0.3232     8196
##
##                ~us(trait):MomId
##
##                                                         post.mean l-95% CI
## traitMeanCorner_control:traitMeanCorner_control.MomId     0.27560  0.08946
## traitMeanCorner_predator:traitMeanCorner_control.MomId    0.03372 -0.15613
## traitMeanCorner_control:traitMeanCorner_predator.MomId    0.03372 -0.15613
## traitMeanCorner_predator:traitMeanCorner_predator.MomId   0.29982  0.09795
##                                                         u-95% CI eff.samp
## traitMeanCorner_control:traitMeanCorner_control.MomId     0.5320    13008
## traitMeanCorner_predator:traitMeanCorner_control.MomId    0.2306    13500
## traitMeanCorner_control:traitMeanCorner_predator.MomId    0.2306    13500
## traitMeanCorner_predator:traitMeanCorner_predator.MomId   0.5924    13390
##
##  R-structure:  ~idh(trait):units
##
##                                  post.mean l-95% CI u-95% CI eff.samp
## traitMeanCorner_control.units      0.6101    0.597   0.6243    13500
## traitMeanCorner_predator.units     0.8608    0.843   0.8796    12664
##
##  Location effects: cbind(MeanCorner_control, MeanCorner_predator) ~ trait * ExpDay - 1 + YearBatch + Hour
##
##                                   post.mean  l-95% CI  u-95% CI eff.samp  pMCMC
## traitMeanCorner_control           0.265596 -0.195500  0.705287     9816 0.2410
## traitMeanCorner_predator          0.486364  0.024908  0.940188    10578 0.0341
## ExpDay                           -0.008156 -0.009705 -0.006613    12947 <7e-05
## YearBatch                        -0.097091 -0.239529  0.034898    11658 0.1687
## Hour                             -0.013602 -0.016250 -0.010943    13500 <7e-05
## traitMeanCorner_predator:ExpDay   0.006502  0.004117  0.008786    12735 <7e-05
##
## traitMeanCorner_control
## traitMeanCorner_predator         *
## ExpDay                           ***
## YearBatch
## Hour                             ***
## traitMeanCorner_predator:ExpDay ***
```

```
## ---
## Signif. codes:  0 '***' 0.001 '**' 0.01 '*' 0.05 '.' 0.1 ' ' 1
```

```r
# FishId shared variance across treatments + MomId
model_id_mom_noidh <- readRDS("model_MeanCorner_id_mom_noidh_10_30_25.rds")
# model_id_mom_noidh <- MCMCglmm(cbind(MeanCorner_control,
# MeanCorner_predator) ~ trait*ExpDay - 1 + YearBatch +
# Hour, random = ~FishId + MomId, rcov = ~idh(trait):units,
# family = c('gaussian', 'gaussian'), data = hourly_data,
# prior = prior.FishId_MomId_NOidh, nitt = 1500000, burnin
# = 150000, thin = 100, verbose = TRUE)
# saveRDS(model_id_mom_noidh, file =
# 'model_MeanCorner_id_mom_noidh_10_30_25.rds')
summary(model_id_mom_noidh)  # Best fit that includes MomId
```

```
##
##  Iterations = 150001:1499901
##  Thinning interval  = 100
##  Sample size  = 13500
##
##  DIC: 82403.09
##
##  G-structure:  ~FishId
##
##        post.mean l-95% CI u-95% CI eff.samp
## FishId    0.2178   0.1585   0.2836    13500
##
##               ~MomId
##
##       post.mean  l-95% CI u-95% CI eff.samp
## MomId   0.02165 0.0002388  0.06891    13500
##
##  R-structure:  ~idh(trait):units
##
##                                post.mean l-95% CI u-95% CI eff.samp
## traitMeanCorner_control.units     0.6102   0.5968   0.6238    13500
## traitMeanCorner_predator.units    0.8607   0.8427   0.8792    13500
##
##  Location effects: cbind(MeanCorner_control, MeanCorner_predator) ~ trait * ExpDay - 1 + YearBatch + Hour
##
##                                 post.mean  l-95% CI  u-95% CI eff.samp  pMCMC
## traitMeanCorner_control          0.175617 -0.104803  0.447216     8121  0.194
## traitMeanCorner_predator         0.391729  0.127396  0.674936     8910  0.004
## ExpDay                          -0.008180 -0.009751 -0.006646    13500 <7e-05
## YearBatch                       -0.060242 -0.154201  0.038514    13500  0.207
## Hour                            -0.013621 -0.016264 -0.010990    13500 <7e-05
## traitMeanCorner_predator:ExpDay  0.006530  0.004190  0.008868    13500 <7e-05
##
## traitMeanCorner_control
## traitMeanCorner_predator        **
## ExpDay                          ***
## YearBatch
## Hour                            ***
## traitMeanCorner_predator:ExpDay ***
## ---
## Signif. codes:  0 '***' 0.001 '**' 0.01 '*' 0.05 '.' 0.1 ' ' 1
```

Now that we have determined the random effects structure (FishId + MomId), we will test the contribution of the fixed effects of Hour and YearBatch.

```r
# Without YearBatch
model_id_mom_hour_noYearBatch <- readRDS("model_MeanCorner_bivariate_id_mom_hour_noYearBatch_8_12_25.rds")
# model_id_mom_noYearBatch <-
```

```r
# MCMCglmm(cbind(MeanCorner_control, MeanCorner_predator) ~
# trait*ExpDay - 1 + Hour, random = ~idh(trait):FishId +
# MomId, rcov = ~idh(trait):units, family = c('gaussian',
# 'gaussian'), data = hourly_data, prior =
# prior.FishId_MomId, nitt = 1500000, burnin = 150000, thin
# = 400, verbose = TRUE)
# saveRDS(model_id_mom_hour_noYearBatch, file =
# 'model_MeanCorner_bivariate_id_mom_hour_noYearBatch_8_12_25.rds')
summary(model_id_mom_hour_noYearBatch)  # Final model selected
```

```
##
##  Iterations = 150001:1499601
##  Thinning interval  = 400
##  Sample size  = 3375
##
##  DIC: 82403.91
##
##  G-structure:  ~idh(trait):FishId
##
##                                  post.mean l-95% CI u-95% CI eff.samp
## traitMeanCorner_control.FishId     0.2161   0.1394   0.3099     3119
## traitMeanCorner_predator.FishId    0.2279   0.1407   0.3224     3375
##
##                ~MomId
##
##       post.mean  l-95% CI u-95% CI eff.samp
## MomId    0.0175 0.0001847  0.05676     2920
##
##  R-structure:  ~idh(trait):units
##
##                                 post.mean l-95% CI u-95% CI eff.samp
## traitMeanCorner_control.units     0.6101   0.5962   0.6227     3375
## traitMeanCorner_predator.units    0.8607   0.8419   0.8779     3643
##
##  Location effects: cbind(MeanCorner_control, MeanCorner_predator) ~ trait * ExpDay - 1 + Hour
##
##                                  post.mean  l-95% CI  u-95% CI eff.samp   pMCMC
## traitMeanCorner_control           0.035342 -0.109786  0.196813     2962 0.66074
## traitMeanCorner_predator          0.249255  0.092598  0.400452     3033 0.00296
## ExpDay                           -0.008198 -0.009704 -0.006705     3375 < 3e-04
## Hour                             -0.013585 -0.016458 -0.011052     3777 < 3e-04
## traitMeanCorner_predator:ExpDay  0.006498  0.004167  0.008835     3375 < 3e-04
##
## traitMeanCorner_control
## traitMeanCorner_predator        **
## ExpDay                          ***
## Hour                            ***
## traitMeanCorner_predator:ExpDay ***
## ---
## Signif. codes:  0 '***' 0.001 '**' 0.01 '*' 0.05 '.' 0.1 ' ' 1
```

```r
# Without Hour or YearBatch
model_id_mom_noYearBatch_noHour <- readRDS("model_MeanCorner_bivariate_id_mom_noYearBatch_noHour_8_14_25.rds
# model_id_mom_noYearBatch_noHour <-
# MCMCglmm(cbind(MeanCorner_control, MeanCorner_predator) ~
# trait*ExpDay - 1, random = ~idh(trait):FishId + MomId,
# rcov = ~idh(trait):units, family = c('gaussian',
# 'gaussian'), data = hourly_data, prior =
# prior.FishId_MomId, nitt = 1500000, burnin = 150000, thin
# = 400, verbose = TRUE)
# saveRDS(model_id_mom_noYearBatch_noHour, file =
```

```r
# 'model_MeanCorner_bivariate_id_mom_noYearBatch_noHour_8_14_25.rds')
summary(model_id_mom_noYearBatch_noHour)
```

```
##
##  Iterations = 150001:1499601
##  Thinning interval  = 400
##  Sample size  = 3375
##
##  DIC: 82501.91
##
##  G-structure:  ~idh(trait):FishId
##
##                                   post.mean l-95% CI u-95% CI eff.samp
## traitMeanCorner_control.FishId     0.2172   0.1379   0.3105     3003
## traitMeanCorner_predator.FishId    0.2268   0.1452   0.3238     3065
##
##                ~MomId
##
##       post.mean l-95% CI u-95% CI eff.samp
## MomId   0.01769 0.000142  0.05631     3082
##
##  R-structure:  ~idh(trait):units
##
##                                  post.mean l-95% CI u-95% CI eff.samp
## traitMeanCorner_control.units     0.6127   0.5998    0.627     3375
## traitMeanCorner_predator.units    0.8629   0.8446    0.881     3375
##
##  Location effects: cbind(MeanCorner_control, MeanCorner_predator) ~ trait * ExpDay - 1
##
##                                   post.mean  l-95% CI  u-95% CI eff.samp  pMCMC
## traitMeanCorner_control         -0.039598 -0.176288  0.127227     2782 0.5807
## traitMeanCorner_predator         0.172815  0.023855  0.328915     3109 0.0279
## ExpDay                          -0.008215 -0.009813 -0.006730     3813 <3e-04
## traitMeanCorner_predator:ExpDay  0.006530  0.004249  0.008781     4074 <3e-04
##
## traitMeanCorner_control
## traitMeanCorner_predator        *
## ExpDay                          ***
## traitMeanCorner_predator:ExpDay ***
## ---
## Signif. codes:  0 '***' 0.001 '**' 0.01 '*' 0.05 '.' 0.1 ' ' 1
```

Including YearBatch does not significantly improve the fit of the model, so we will not include it in our final model. Including Hour does significantly improve the fit of the model, so we will include it.

Variance does not differ by treatment, so we should use a univariate model to partition variance (as with velocity, above).

Univariate model for estimating overall variance components

```r
# Set priors:

# Null
prior.univariate.null <- list(R = list(V = 1, nu = 0.002))

# FishId
prior.univariate.FishId <- list(R = list(V = 1, nu = 0.002),
    G = list(G1 = list(V = 1, nu = 0.002, alpha.mu = 0, alpha.V = 25^2)))

# MomId
```

```r
prior.univariate.MomId <- list(R = list(V = 1, nu = 0.002), G = list(G1 = list(V = 1,
    nu = 0.002, alpha.mu = 0, alpha.V = 25^2)))

# FishId + MomId
prior.univariate.FishId.MomId <- list(R = list(V = 1, nu = 0.002),
    G = list(G1 = list(V = 1, nu = 0.002, alpha.mu = 0, alpha.V = 25^2),
        G2 = list(V = 1, nu = 0.002, alpha.mu = 0, alpha.V = 25^2)))

# FishId slopes + MomId
prior.univariate.FishId_slopes.MomId <- list(R = list(V = 1,
    nu = 0.002), G = list(G1 = list(V = diag(2), nu = 0.002,
    alpha.mu = rep(0, 2), alpha.V = diag(25^2, 2)), G2 = list(V = 1,
    nu = 0.002, alpha.mu = 0, alpha.V = 25^2)))

# FishId slopes + MomId slopes
prior.univariate.FishId_slopes.MomId_slopes <- list(R = list(V = 1,
    nu = 0.002), G = list(G1 = list(V = diag(2), nu = 0.002,
    alpha.mu = rep(0, 2), alpha.V = diag(25^2, 2)), G2 = list(V = diag(2),
    nu = 0.002, alpha.mu = rep(0, 2), alpha.V = diag(25^2, 2))))

# FishId slopes + MomId with heterogeneous residual
# variance
hourly_data$ExpDay_factor <- as.factor(hourly_data$ExpDay)
num_days <- nlevels(hourly_data$ExpDay_factor)
R_prior <- list(V = diag(num_days) * 0.5, nu = num_days + 0.002)
prior.univariate.FishId_slopes.MomId.heteroResidual <- list(R = R_prior,
    G = list(G1 = list(V = diag(2), nu = 0.002, alpha.mu = rep(0,
        2), alpha.V = diag(25^2, 2)), G2 = list(V = 1, nu = 0.002,
        alpha.mu = 0, alpha.V = 25^2)))

# FishId slopes + MomId with heterogeneous residual
# variance MORE INFORMED PRIORS
prior.univariate.FishId_slopes.MomId.heteroResidual_informed <- list(R = R_prior,
    G = list(G1 = list(V = diag(2) * 0.5, nu = 2.002, alpha.mu = c(0,
        0), alpha.V = diag(2) * 25^2), G2 = list(V = 1, nu = 1.002,
        alpha.mu = 0, alpha.V = 25^2)))

### Model comparison

# Null
model_MeanCorner_big_univariate_null <- readRDS("model_MeanCorner_big_univariate_null_9_19_25.rds")
# model_MeanCorner_big_univariate_null <-
# MCMCglmm(MeanCorner_hourly_scaled ~ PredatorCue*ExpDay +
# YearBatch + Hour, rcov = ~units, family = 'gaussian',
# data = hourly_data, prior = prior.univariate.null, nitt =
# 500000, burnin = 50000, thin = 100)
# saveRDS(model_MeanCorner_big_univariate_null, file =
# 'model_MeanCorner_big_univariate_null_9_19_25.rds')
summary(model_MeanCorner_big_univariate_null)
```

```
##
##  Iterations = 50001:499901
##  Thinning interval  = 100
##  Sample size  = 4500
##
##  DIC: 91426.75
##
##  R-structure:  ~units
##
##       post.mean l-95% CI u-95% CI eff.samp
## units    0.9667   0.9519    0.982     4500
```

```
##
##  Location effects: MeanCorner_hourly_scaled ~ PredatorCue * ExpDay + YearBatch + Hour
##
##                    post.mean  l-95% CI  u-95% CI eff.samp  pMCMC
## (Intercept)         0.124160  0.081915  0.165362     4079 <2e-04 ***
## PredatorCue         0.220093  0.179271  0.261262     4921 <2e-04 ***
## ExpDay             -0.009008 -0.010959 -0.007153     4500 <2e-04 ***
## YearBatch          -0.041283 -0.051407 -0.031813     4807 <2e-04 ***
## Hour               -0.013636 -0.016827 -0.010583     4500 <2e-04 ***
## PredatorCue:ExpDay  0.008004  0.005389  0.010677     5092 <2e-04 ***
## ---
## Signif. codes:  0 '***' 0.001 '**' 0.01 '*' 0.05 '.' 0.1 ' ' 1
```

```
# FishId
model_MeanCorner_big_univariate_id <- readRDS("model_MeanCorner_big_univariate_id_9_20_25.rds")
# model_MeanCorner_big_univariate_id <-
# MCMCglmm(MeanCorner_hourly_scaled ~ PredatorCue*ExpDay +
# YearBatch + Hour, random = ~FishId, rcov = ~units, family
# = 'gaussian', data = hourly_data, prior =
# prior.univariate.FishId, nitt = 500000, burnin = 50000,
# thin = 100) saveRDS(model_MeanCorner_big_univariate_id,
# file = 'model_MeanCorner_big_univariate_id_9_20_25.rds')
summary(model_MeanCorner_big_univariate_id)
```

```
##
##  Iterations = 50001:499901
##  Thinning interval  = 100
##  Sample size  = 4500
##
##  DIC: 82877.47
##
##  G-structure:  ~FishId
##
##        post.mean l-95% CI u-95% CI eff.samp
## FishId    0.2322   0.1735   0.3025     4500
##
##  R-structure:  ~units
##
##       post.mean l-95% CI u-95% CI eff.samp
## units    0.7415   0.7309   0.7535     4500
##
##  Location effects: MeanCorner_hourly_scaled ~ PredatorCue * ExpDay + YearBatch + Hour
##
##                    post.mean  l-95% CI  u-95% CI eff.samp  pMCMC
## (Intercept)         0.114050 -0.123235  0.336680     4212  0.338
## PredatorCue         0.219557  0.034783  0.406936     4500  0.024 *
## ExpDay             -0.008183 -0.009769 -0.006435     4500 <2e-04 ***
## YearBatch          -0.037859 -0.116490  0.039068     4500  0.341
## Hour               -0.013618 -0.016264 -0.010790     4500 <2e-04 ***
## PredatorCue:ExpDay  0.006494  0.004367  0.008964     3949 <2e-04 ***
## ---
## Signif. codes:  0 '***' 0.001 '**' 0.01 '*' 0.05 '.' 0.1 ' ' 1
```

```
# MomId
model_MeanCorner_big_univariate_mom <- readRDS("model_MeanCorner_big_univariate_mom_9_20_25.rds")
# model_MeanCorner_big_univariate_mom <-
# MCMCglmm(MeanCorner_hourly_scaled ~ PredatorCue*ExpDay +
# YearBatch + Hour, random = ~MomId, rcov = ~units, family
# = 'gaussian', data = hourly_data, prior =
# prior.univariate.MomId, nitt = 500000, burnin = 50000,
# thin = 100) saveRDS(model_MeanCorner_big_univariate_mom,
# file = 'model_MeanCorner_big_univariate_mom_9_20_25.rds')
summary(model_MeanCorner_big_univariate_mom)
```

```
##
##  Iterations = 50001:499901
##  Thinning interval  = 100
##  Sample size  = 4500
##
##  DIC: 89989.95
##
##  G-structure:  ~MomId
##
##       post.mean l-95% CI u-95% CI eff.samp
## MomId   0.07732  0.02289   0.1572     3638
##
##  R-structure:  ~units
##
##       post.mean l-95% CI u-95% CI eff.samp
## units    0.9248   0.9112   0.9394     4500
##
##  Location effects: MeanCorner_hourly_scaled ~ PredatorCue * ExpDay + YearBatch + Hour
##
##                    post.mean  l-95% CI  u-95% CI eff.samp  pMCMC
## (Intercept)         0.237300 -0.070981  0.522760     4395  0.111
## PredatorCue         0.206303  0.168079  0.247651     4500 <2e-04 ***
## ExpDay             -0.008923 -0.010937 -0.007097     3982 <2e-04 ***
## YearBatch          -0.078606 -0.184419  0.042297     4525  0.166
## Hour               -0.013580 -0.016563 -0.010618     4942 <2e-04 ***
## PredatorCue:ExpDay  0.007870  0.005356  0.010534     4500 <2e-04 ***
## ---
## Signif. codes:  0 '***' 0.001 '**' 0.01 '*' 0.05 '.' 0.1 ' ' 1
```

```r
# FishId, MomId
model_MeanCorner_big_univariate_id_mom <- readRDS("model_MeanCorner_big_univariate_id_mom_9_19_25.rds")
# model_MeanCorner_big_univariate_id_mom <-
# MCMCglmm(MeanCorner_hourly_scaled ~ PredatorCue*ExpDay +
# YearBatch + Hour, random = ~FishId + MomId, rcov =
# ~units, family = 'gaussian', data = hourly_data, prior =
# prior.univariate.FishId.MomId, nitt = 500000, burnin =
# 50000, thin = 100)
# saveRDS(model_MeanCorner_big_univariate_id_mom, file =
# 'model_MeanCorner_big_univariate_id_mom_9_19_25.rds')
summary(model_MeanCorner_big_univariate_id_mom)
```

```
##
##  Iterations = 50001:499901
##  Thinning interval  = 100
##  Sample size  = 4500
##
##  DIC: 82877.65
##
##  G-structure:  ~FishId
##
##        post.mean l-95% CI u-95% CI eff.samp
## FishId    0.2193    0.158   0.2856     3574
##
##                ~MomId
##
##       post.mean  l-95% CI u-95% CI eff.samp
## MomId   0.02958 5.709e-09  0.09272     1204
##
##  R-structure:  ~units
##
##       post.mean l-95% CI u-95% CI eff.samp
```

```
## units    0.7415   0.7303   0.7521     4500
##
##  Location effects: MeanCorner_hourly_scaled ~ PredatorCue * ExpDay + YearBatch + Hour
##
##                    post.mean  l-95% CI  u-95% CI eff.samp  pMCMC
## (Intercept)         0.161584 -0.125944  0.450267     4500 0.2587
## PredatorCue         0.213905  0.028429  0.388913     4500 0.0196 *
## ExpDay             -0.008184 -0.009877 -0.006405     4500 <2e-04 ***
## YearBatch          -0.053379 -0.158025  0.050765     4242 0.2831
## Hour               -0.013603 -0.016353 -0.011154     4270 <2e-04 ***
## PredatorCue:ExpDay  0.006494  0.004095  0.008805     4500 <2e-04 ***
## ---
## Signif. codes:  0 '***' 0.001 '**' 0.01 '*' 0.05 '.' 0.1 ' ' 1
```

```
# FishId with slopes, MomId
model_MeanCorner_big_univariate_id_slopes_mom <- readRDS("model_MeanCorner_big_univariate_id_slopes_mom_9_19
# model_MeanCorner_big_univariate_id_slopes_mom <-
# MCMCglmm(MeanCorner_hourly_scaled ~ PredatorCue*ExpDay +
# YearBatch + Hour, random = ~us(1 + ExpDay):FishId +
# MomId, rcov = ~units, family = 'gaussian', data =
# hourly_data, prior =
# prior.univariate.FishId_slopes.MomId, nitt = 500000,
# burnin = 50000, thin = 100)
# saveRDS(model_MeanCorner_big_univariate_id_slopes_mom,
# file =
# 'model_MeanCorner_big_univariate_id_slopes_mom_9_19_25.rds')
summary(model_MeanCorner_big_univariate_id_slopes_mom)  # Best model!
```

```
##
##  Iterations = 50001:499901
##  Thinning interval  = 100
##  Sample size  = 4500
##
##  DIC: 81218.55
##
##  G-structure:  ~us(1 + ExpDay):FishId
##
##                                post.mean  l-95% CI   u-95% CI eff.samp
## (Intercept):(Intercept).FishId  0.2621474  0.189838  0.3458344     4201
## ExpDay:(Intercept).FishId      -0.0056987 -0.008782 -0.0026389     4500
## (Intercept):ExpDay.FishId      -0.0056987 -0.008782 -0.0026389     4500
## ExpDay:ExpDay.FishId            0.0006384  0.000452  0.0008329     4500
##
##                ~MomId
##
##       post.mean  l-95% CI u-95% CI eff.samp
## MomId   0.03196 8.458e-08   0.0932     1784
##
##  R-structure:  ~units
##
##       post.mean l-95% CI u-95% CI eff.samp
## units    0.7026   0.6919   0.7133     4790
##
##  Location effects: MeanCorner_hourly_scaled ~ PredatorCue * ExpDay + YearBatch + Hour
##
##                    post.mean  l-95% CI  u-95% CI eff.samp  pMCMC
## (Intercept)         0.062644 -0.243252  0.342106     4569 0.6787
## PredatorCue         0.218416  0.013300  0.419834     4500 0.0391 *
## ExpDay             -0.008658 -0.016079 -0.001178     4500 0.0249 *
## YearBatch          -0.010257 -0.114257  0.089214     4890 0.8360
## Hour               -0.013564 -0.016216 -0.010934     4500 <2e-04 ***
## PredatorCue:ExpDay  0.006528 -0.003686  0.016646     4500 0.2187
```

```
## ---
## Signif. codes:  0 '***' 0.001 '**' 0.01 '*' 0.05 '.' 0.1 ' ' 1
```

```r
# FishId with slopes, MomId with slopes
model_MeanCorner_big_univariate_id_slopes_mom_slopes <- readRDS("model_MeanCorner_big_univariate_id_slopes_m
# model_MeanCorner_big_univariate_id_slopes_mom_slopes <-
# MCMCglmm(MeanCorner_hourly_scaled ~ PredatorCue*ExpDay +
# YearBatch + Hour, random = ~us(1 + ExpDay):FishId + us(1
# + ExpDay):MomId, rcov = ~units, family = 'gaussian', data
# = hourly_data, prior =
# prior.univariate.FishId_slopes.MomId_slopes, nitt =
# 500000, burnin = 50000, thin = 100)
# saveRDS(model_MeanCorner_big_univariate_id_slopes_mom_slopes,
# file =
# 'model_MeanCorner_big_univariate_id_slopes_mom_slopes_9_19_25.rds')
summary(model_MeanCorner_big_univariate_id_slopes_mom_slopes)
```

```
##
##  Iterations = 50001:499901
##  Thinning interval  = 100
##  Sample size  = 4500
##
##  DIC: 81218.43
##
##  G-structure:  ~us(1 + ExpDay):FishId
##
##                                   post.mean   l-95% CI   u-95% CI eff.samp
## (Intercept):(Intercept).FishId  0.2606031  0.1853611  0.3391936     4500
## ExpDay:(Intercept).FishId      -0.0054806 -0.0086711 -0.0026429     4281
## (Intercept):ExpDay.FishId      -0.0054806 -0.0086711 -0.0026429     4281
## ExpDay:ExpDay.FishId            0.0006203  0.0004408  0.0008213     4146
##
##                ~us(1 + ExpDay):MomId
##
##                                  post.mean   l-95% CI  u-95% CI eff.samp
## (Intercept):(Intercept).MomId  3.859e-02  1.753e-08 0.1122935     2412
## ExpDay:(Intercept).MomId      -5.070e-04 -2.583e-03 0.0012097     4500
## (Intercept):ExpDay.MomId      -5.070e-04 -2.583e-03 0.0012097     4500
## ExpDay:ExpDay.MomId            4.926e-05  9.689e-12 0.0001717     3060
##
##  R-structure:  ~units
##
##       post.mean l-95% CI u-95% CI eff.samp
## units    0.7027   0.6922   0.7134     4500
##
##  Location effects: MeanCorner_hourly_scaled ~ PredatorCue * ExpDay + YearBatch + Hour
##
##                     post.mean   l-95% CI   u-95% CI eff.samp  pMCMC
## (Intercept)         0.0576502 -0.2145507  0.3672419     4500 0.6902
## PredatorCue         0.2233035  0.0278220  0.4241607     4028 0.0284 *
## ExpDay             -0.0085357 -0.0168780 -0.0004456     4500 0.0489 *
## YearBatch          -0.0094305 -0.1049848  0.0898379     4500 0.8244
## Hour               -0.0136048 -0.0161958 -0.0110199     4500 <2e-04 ***
## PredatorCue:ExpDay  0.0062569 -0.0040191  0.0155894     4500 0.2018
## ---
## Signif. codes:  0 '***' 0.001 '**' 0.01 '*' 0.05 '.' 0.1 ' ' 1
```

```r
# Now use best model (FishId with slopes, MomId with
# intercepts) with heterogeneous residual variance
model_MeanCorner_big_univariate_id_slopes_mom_hetero_residual <- readRDS("model_MeanCorner_big_univariate_id
# model_MeanCorner_big_univariate_id_slopes_mom_hetero_residual
# <- MCMCglmm(MeanCorner_hourly_scaled ~ PredatorCue*ExpDay
```

```r
# + YearBatch + Hour, random = ~us(1 + ExpDay):FishId +
# MomId, rcov = ~idh(ExpDay_factor):units, family =
# 'gaussian', data = hourly_data, prior =
# prior.univariate.FishId_slopes.MomId.heteroResidual, nitt
# = 500000, burnin = 50000, thin = 100)
# saveRDS(model_MeanCorner_big_univariate_id_slopes_mom_hetero_residual,
# file =
# 'model_MeanCorner_big_univariate_id_slopes_mom_hetero_residual_9_23_25.rds')
summary(model_MeanCorner_big_univariate_id_slopes_mom_hetero_residual)  # Better fit with heterogeneous res
```

```
##
##  Iterations = 50001:499901
##  Thinning interval  = 100
##  Sample size  = 4500
##
##  DIC: 80051.6
##
##  G-structure:  ~us(1 + ExpDay):FishId
##
##                                  post.mean   l-95% CI   u-95% CI eff.samp
## (Intercept):(Intercept).FishId  0.2629970  0.1880861  0.3446324     3939
## ExpDay:(Intercept).FishId      -0.0056168 -0.0086424 -0.0028413     4110
## (Intercept):ExpDay.FishId      -0.0056168 -0.0086424 -0.0028413     4110
## ExpDay:ExpDay.FishId            0.0006257  0.0004454  0.0008142     4016
##
##                ~MomId
##
##       post.mean  l-95% CI u-95% CI eff.samp
## MomId   0.03148 2.215e-10  0.08761     1512
##
##  R-structure:  ~idh(ExpDay_factor):units
##
##                        post.mean l-95% CI u-95% CI eff.samp
## ExpDay_factor0.units      1.3443   1.2417   1.4578     4500
## ExpDay_factor1.units      1.0416   0.9557   1.1219     4500
## ExpDay_factor2.units      1.1581   1.0699   1.2529     4235
## ExpDay_factor3.units      1.0657   0.9840   1.1519     4500
## ExpDay_factor4.units      1.0137   0.9309   1.0934     4814
## ExpDay_factor5.units      0.6316   0.5810   0.6833     4500
## ExpDay_factor6.units      0.6715   0.6174   0.7275     4500
## ExpDay_factor7.units      0.6894   0.6320   0.7427     4500
## ExpDay_factor8.units      0.5180   0.4789   0.5624     4500
## ExpDay_factor9.units      0.7035   0.6497   0.7620     4500
## ExpDay_factor10.units     0.6665   0.6114   0.7226     4500
## ExpDay_factor11.units     0.5802   0.5339   0.6290     4500
## ExpDay_factor12.units     0.6787   0.6275   0.7364     4500
## ExpDay_factor13.units     0.5942   0.5497   0.6440     4500
## ExpDay_factor14.units     0.5329   0.4919   0.5773     4500
## ExpDay_factor15.units     0.6528   0.6034   0.7065     5041
## ExpDay_factor16.units     0.7324   0.6745   0.7906     4500
## ExpDay_factor17.units     0.6240   0.5762   0.6766     4500
## ExpDay_factor18.units     0.5969   0.5453   0.6454     4500
## ExpDay_factor19.units     0.5565   0.5128   0.6007     4694
## ExpDay_factor20.units     0.6224   0.5717   0.6722     4500
## ExpDay_factor21.units     0.6368   0.5855   0.6880     4500
## ExpDay_factor22.units     0.5674   0.5209   0.6133     4500
## ExpDay_factor23.units     0.4739   0.4333   0.5116     4500
## ExpDay_factor24.units     0.5397   0.4993   0.5856     4500
## ExpDay_factor25.units     0.4821   0.4447   0.5247     4500
## ExpDay_factor26.units     0.6141   0.5629   0.6656     4500
## ExpDay_factor27.units     0.5632   0.5139   0.6096     4907
```

```
##
##  Location effects: MeanCorner_hourly_scaled ~ PredatorCue * ExpDay + YearBatch + Hour
##
##                    post.mean  l-95% CI  u-95% CI eff.samp  pMCMC
## (Intercept)         0.075095 -0.234620  0.369503     4296 0.6196
## PredatorCue         0.241752  0.047817  0.443691     4500 0.0182 *
## ExpDay             -0.006392 -0.013203  0.001073     4500 0.0782 .
## YearBatch          -0.026225 -0.133901  0.084818     4500 0.6018
## Hour               -0.015722 -0.018252 -0.013104     4500 <2e-04 ***
## PredatorCue:ExpDay  0.005246 -0.004967  0.015073     4500 0.2991
## ---
## Signif. codes:  0 '***' 0.001 '**' 0.01 '*' 0.05 '.' 0.1 ' ' 1
```

```
# Comparison of best fit model with one using more informed
# priors to see if that makes a difference
model_MeanCorner_big_univariate_id_slopes_mom_slopes_hetero_residual_informed_priors <- readRDS("model_Mean(
# model_MeanCorner_big_univariate_id_slopes_mom_slopes_hetero_residual_informed_priors
# <- MCMCglmm(MeanCorner_hourly_scaled ~ PredatorCue*ExpDay
# + YearBatch + Hour, random = ~us(1 + ExpDay):FishId +
# MomId, rcov = ~idh(ExpDay_factor):units, family =
# 'gaussian', data = hourly_data, prior =
# prior.univariate.FishId_slopes.MomId.heteroResidual_informed,
# nitt = 500000, burnin = 50000, thin = 100)
# saveRDS(model_MeanCorner_big_univariate_id_slopes_mom_slopes_hetero_residual_informed_priors,
# file =
# 'model_MeanCorner_big_univariate_id_slopes_mom_hetero_residual_informed_priors_9_23_25.rds')
summary(model_MeanCorner_big_univariate_id_slopes_mom_slopes_hetero_residual_informed_priors)  # Negligible
```

```
##
##  Iterations = 50001:499901
##  Thinning interval  = 100
##  Sample size  = 4500
##
##  DIC: 80051.63
##
##  G-structure:  ~us(1 + ExpDay):FishId
##
##                                post.mean   l-95% CI  u-95% CI eff.samp
## (Intercept):(Intercept).FishId  0.2635933  0.1890068  0.349861     3131
## ExpDay:(Intercept).FishId      -0.0054962 -0.0085557 -0.002662     5022
## (Intercept):ExpDay.FishId      -0.0054962 -0.0085557 -0.002662     5022
## ExpDay:ExpDay.FishId            0.0006185  0.0004458  0.000804     3824
##
##                ~MomId
##
##       post.mean l-95% CI u-95% CI eff.samp
## MomId   0.03204 1.05e-07  0.09725     1091
##
##  R-structure:  ~idh(ExpDay_factor):units
##
##                       post.mean l-95% CI u-95% CI eff.samp
## ExpDay_factor0.units     1.3442   1.2389   1.4591     4500
## ExpDay_factor1.units     1.0403   0.9585   1.1271     4500
## ExpDay_factor2.units     1.1584   1.0671   1.2532     4500
## ExpDay_factor3.units     1.0663   0.9858   1.1574     4500
## ExpDay_factor4.units     1.0130   0.9277   1.0909     4500
## ExpDay_factor5.units     0.6321   0.5796   0.6819     4500
## ExpDay_factor6.units     0.6715   0.6149   0.7273     4500
## ExpDay_factor7.units     0.6890   0.6351   0.7458     4500
## ExpDay_factor8.units     0.5181   0.4763   0.5599     4500
## ExpDay_factor9.units     0.7030   0.6481   0.7577     4737
## ExpDay_factor10.units    0.6665   0.6160   0.7267     4500
```

```
## ExpDay_factor11.units    0.5800   0.5295   0.6270     4500
## ExpDay_factor12.units    0.6788   0.6246   0.7348     4500
## ExpDay_factor13.units    0.5939   0.5454   0.6414     4500
## ExpDay_factor14.units    0.5336   0.4939   0.5787     4500
## ExpDay_factor15.units    0.6530   0.6021   0.7086     4500
## ExpDay_factor16.units    0.7333   0.6772   0.7936     4500
## ExpDay_factor17.units    0.6239   0.5771   0.6769     4500
## ExpDay_factor18.units    0.5967   0.5488   0.6468     4500
## ExpDay_factor19.units    0.5574   0.5143   0.6047     4500
## ExpDay_factor20.units    0.6224   0.5711   0.6728     4500
## ExpDay_factor21.units    0.6366   0.5862   0.6868     4500
## ExpDay_factor22.units    0.5670   0.5253   0.6137     4500
## ExpDay_factor23.units    0.4742   0.4383   0.5132     4500
## ExpDay_factor24.units    0.5395   0.4962   0.5832     4443
## ExpDay_factor25.units    0.4816   0.4463   0.5227     3999
## ExpDay_factor26.units    0.6148   0.5664   0.6661     4500
## ExpDay_factor27.units    0.5640   0.5180   0.6155     4500
##
##  Location effects: MeanCorner_hourly_scaled ~ PredatorCue * ExpDay + YearBatch + Hour
##
##                      post.mean  l-95% CI  u-95% CI eff.samp  pMCMC
## (Intercept)           0.078997 -0.224987  0.389145     4500 0.5956
## PredatorCue           0.237253  0.044372  0.443719     4500 0.0227 *
## ExpDay               -0.006352 -0.013288  0.001247     4500 0.0840 .
## YearBatch            -0.027673 -0.135696  0.078968     4500 0.6093
## Hour                 -0.015707 -0.018353 -0.013184     4636 <2e-04 ***
## PredatorCue:ExpDay    0.005265 -0.005059  0.015180     4718 0.3000
## ---
## Signif. codes:  0 '***' 0.001 '**' 0.01 '*' 0.05 '.' 0.1 ' ' 1
```

Now we test for inclusion of additional fixed effects, YearBatch and Hour

```
# Without YearBatch
model_MeanCorner_big_univariate_id_slopes_mom_noYearBatch <- readRDS("model_MeanCorner_big_univariate_id_slo
# model_MeanCorner_big_univariate_id_slopes_mom_noYearBatch
# <- MCMCglmm(MeanCorner_hourly_scaled ~ PredatorCue*ExpDay
# + Hour, random = ~us(1 + ExpDay):FishId + MomId, rcov =
# ~idh(ExpDay_factor):units, family = 'gaussian', data =
# hourly_data, prior =
# prior.univariate.FishId_slopes.MomId.heteroResidual, nitt
# = 500000, burnin = 50000, thin = 100)
# saveRDS(model_MeanCorner_big_univariate_id_slopes_mom_noYearBatch,
# file =
# 'model_MeanCorner_big_univariate_id_slopes_mom_noYearBatch_9_23_25.rds')
summary(model_MeanCorner_big_univariate_id_slopes_mom_noYearBatch)  # Including YearBatch does not improve
```

```
##
##  Iterations = 50001:499901
##  Thinning interval  = 100
##  Sample size  = 4500
##
##  DIC: 80051.12
##
##  G-structure:  ~us(1 + ExpDay):FishId
##
##                                 post.mean   l-95% CI   u-95% CI eff.samp
## (Intercept):(Intercept).FishId  0.2639355  0.1898373  0.3467658     3673
## ExpDay:(Intercept).FishId      -0.0056266 -0.0085289 -0.0025519     4500
## (Intercept):ExpDay.FishId      -0.0056266 -0.0085289 -0.0025519     4500
## ExpDay:ExpDay.FishId            0.0006234  0.0004467  0.0008165     3877
##
##                ~MomId
##
```

```
##       post.mean l-95% CI u-95% CI eff.samp
## MomId   0.02932 6.28e-08  0.08464     1704
##
##  R-structure:  ~idh(ExpDay_factor):units
##
##                         post.mean l-95% CI u-95% CI eff.samp
## ExpDay_factor0.units       1.3443   1.2415   1.4570     4500
## ExpDay_factor1.units       1.0393   0.9550   1.1202     4500
## ExpDay_factor2.units       1.1570   1.0631   1.2497     4500
## ExpDay_factor3.units       1.0648   0.9795   1.1466     4500
## ExpDay_factor4.units       1.0139   0.9307   1.0942     4500
## ExpDay_factor5.units       0.6313   0.5801   0.6818     4500
## ExpDay_factor6.units       0.6714   0.6188   0.7294     4500
## ExpDay_factor7.units       0.6896   0.6330   0.7423     4500
## ExpDay_factor8.units       0.5176   0.4777   0.5607     4500
## ExpDay_factor9.units       0.7022   0.6482   0.7566     5123
## ExpDay_factor10.units      0.6663   0.6099   0.7206     4500
## ExpDay_factor11.units      0.5806   0.5314   0.6275     4500
## ExpDay_factor12.units      0.6787   0.6254   0.7339     4788
## ExpDay_factor13.units      0.5944   0.5464   0.6398     4500
## ExpDay_factor14.units      0.5336   0.4915   0.5756     4500
## ExpDay_factor15.units      0.6538   0.6033   0.7090     4267
## ExpDay_factor16.units      0.7333   0.6762   0.7949     4500
## ExpDay_factor17.units      0.6249   0.5728   0.6734     4500
## ExpDay_factor18.units      0.5972   0.5492   0.6470     4304
## ExpDay_factor19.units      0.5576   0.5155   0.6038     4500
## ExpDay_factor20.units      0.6229   0.5737   0.6743     4500
## ExpDay_factor21.units      0.6369   0.5868   0.6897     4500
## ExpDay_factor22.units      0.5677   0.5230   0.6149     4500
## ExpDay_factor23.units      0.4739   0.4379   0.5158     4793
## ExpDay_factor24.units      0.5390   0.4954   0.5835     4500
## ExpDay_factor25.units      0.4824   0.4429   0.5224     4500
## ExpDay_factor26.units      0.6147   0.5648   0.6652     4500
## ExpDay_factor27.units      0.5639   0.5177   0.6097     4500
##
##  Location effects: MeanCorner_hourly_scaled ~ PredatorCue * ExpDay + Hour
##
##                     post.mean   l-95% CI   u-95% CI eff.samp  pMCMC
## (Intercept)         0.0137760 -0.1501135  0.1996049     4500 0.8796
## PredatorCue         0.2407956  0.0383440  0.4348958     4500 0.0169 *
## ExpDay             -0.0064578 -0.0134922  0.0009482     4500 0.0836 .
## Hour               -0.0156988 -0.0182102 -0.0131676     4500 <2e-04 ***
## PredatorCue:ExpDay  0.0053428 -0.0046435  0.0155781     4500 0.2902
## ---
## Signif. codes:  0 '***' 0.001 '**' 0.01 '*' 0.05 '.' 0.1 ' ' 1
```

```r
# Without Hour
model_MeanCorner_big_univariate_id_slopes_mom_noHour <- readRDS("model_MeanCorner_big_univariate_id_slopes_m
# model_MeanCorner_big_univariate_id_slopes_mom_noHour <-
# MCMCglmm(MeanCorner_hourly_scaled ~ PredatorCue*ExpDay,
# random = ~us(1 + ExpDay):FishId + MomId, rcov =
# ~idh(ExpDay_factor):units, family = 'gaussian', data =
# hourly_data, prior =
# prior.univariate.FishId_slopes.MomId.heteroResidual, nitt
# = 500000, burnin = 50000, thin = 100)
# saveRDS(model_MeanCorner_big_univariate_id_slopes_mom_noHour,
# file =
# 'model_MeanCorner_big_univariate_id_slopes_mom_noHour_9_23_25.rds')
summary(model_MeanCorner_big_univariate_id_slopes_mom_noHour)
```

```
##
##  Iterations = 50001:499901
```

```
##  Thinning interval  = 100
##  Sample size  = 4500
##
##  DIC: 80195.45
##
##  G-structure:  ~us(1 + ExpDay):FishId
##
##                                 post.mean   l-95% CI   u-95% CI eff.samp
## (Intercept):(Intercept).FishId  0.2641139  0.1919855  0.3493616     4500
## ExpDay:(Intercept).FishId      -0.0056323 -0.0084436 -0.0024908     4738
## (Intercept):ExpDay.FishId      -0.0056323 -0.0084436 -0.0024908     4738
## ExpDay:ExpDay.FishId            0.0006248  0.0004511  0.0008137     4500
##
##                ~MomId
##
##       post.mean l-95% CI u-95% CI eff.samp
## MomId   0.02939 1.88e-08  0.08786     1552
##
##  R-structure:  ~idh(ExpDay_factor):units
##
##                       post.mean l-95% CI u-95% CI eff.samp
## ExpDay_factor0.units     1.3382   1.2253   1.4495     4799
## ExpDay_factor1.units     1.0330   0.9535   1.1159     4500
## ExpDay_factor2.units     1.1506   1.0616   1.2460     4500
## ExpDay_factor3.units     1.0600   0.9770   1.1467     4500
## ExpDay_factor4.units     1.0098   0.9296   1.0916     4500
## ExpDay_factor5.units     0.6328   0.5796   0.6817     4500
## ExpDay_factor6.units     0.6673   0.6161   0.7222     4500
## ExpDay_factor7.units     0.6816   0.6267   0.7356     4500
## ExpDay_factor8.units     0.5175   0.4762   0.5588     4500
## ExpDay_factor9.units     0.7079   0.6530   0.7658     4500
## ExpDay_factor10.units    0.6611   0.6066   0.7147     4296
## ExpDay_factor11.units    0.5849   0.5354   0.6326     4212
## ExpDay_factor12.units    0.6776   0.6223   0.7310     4500
## ExpDay_factor13.units    0.6025   0.5551   0.6525     4288
## ExpDay_factor14.units    0.5414   0.4996   0.5857     4500
## ExpDay_factor15.units    0.6644   0.6098   0.7161     4500
## ExpDay_factor16.units    0.7424   0.6828   0.8008     4500
## ExpDay_factor17.units    0.6296   0.5806   0.6810     4500
## ExpDay_factor18.units    0.5979   0.5490   0.6461     4500
## ExpDay_factor19.units    0.5629   0.5176   0.6078     4500
## ExpDay_factor20.units    0.6304   0.5786   0.6817     4500
## ExpDay_factor21.units    0.6438   0.5949   0.6977     4500
## ExpDay_factor22.units    0.5697   0.5243   0.6158     4500
## ExpDay_factor23.units    0.4773   0.4391   0.5184     4500
## ExpDay_factor24.units    0.5399   0.4950   0.5827     4500
## ExpDay_factor25.units    0.4918   0.4492   0.5315     4500
## ExpDay_factor26.units    0.6214   0.5703   0.6717     4043
## ExpDay_factor27.units    0.5702   0.5209   0.6182     4500
##
##  Location effects: MeanCorner_hourly_scaled ~ PredatorCue * ExpDay
##
##                     post.mean   l-95% CI   u-95% CI eff.samp  pMCMC
## (Intercept)        -0.0700705 -0.2391709  0.1085908     4500 0.4138
## PredatorCue         0.2392679  0.0260443  0.4347305     4500 0.0178 *
## ExpDay             -0.0063352 -0.0132915  0.0007035     4500 0.0804 .
## PredatorCue:ExpDay  0.0051966 -0.0047134  0.0150727     4500 0.3093
## ---
## Signif. codes:  0 '***' 0.001 '**' 0.01 '*' 0.05 '.' 0.1 ' ' 1
```

Including Hour improves the fit of the model, but YearBatch doesn't.

Testing for effect of body size

Next, we will look at the importance of body size. Not all individuals have body size data because not every individual has data through day 27. Here we will prepare model comparison only including individuals with body size data.

Body size model comparison

```
# With body size
model_MeanCorner_bodysize <- readRDS("model_MeanCorner_bodysize_9_24_25.rds")
# model_MeanCorner_bodysize <-
# MCMCglmm(MeanCorner_hourly_scaled ~ PredatorCue*ExpDay +
# Hour + avg_length + avg_width, random = ~us(1 +
# ExpDay):FishId + MomId, rcov = ~units, family =
# 'gaussian', data = bodysize_only_data, prior =
# prior.univariate.FishId_slopes.MomId, nitt = 500000,
# burnin = 50000, thin = 100)
# saveRDS(model_MeanCorner_bodysize, file =
# 'model_MeanCorner_bodysize_9_24_25.rds')
summary(model_MeanCorner_bodysize)
```

```
##
##  Iterations = 50001:499901
##  Thinning interval  = 100
##  Sample size  = 4500
##
##  DIC: 4823.021
##
##  G-structure:  ~us(1 + ExpDay):FishId
##
##                                post.mean   l-95% CI u-95% CI eff.samp
## (Intercept):(Intercept).FishId   0.37794  0.2479624  0.51144     4500
## ExpDay:(Intercept).FishId       -0.01111 -0.0174340 -0.00619     4500
## (Intercept):ExpDay.FishId       -0.01111 -0.0174340 -0.00619     4500
## ExpDay:ExpDay.FishId             0.00093  0.0006032  0.00129     4500
##
##               ~MomId
##
##       post.mean  l-95% CI u-95% CI eff.samp
## MomId   0.01165 5.675e-09  0.04437     4124
##
##  R-structure:  ~units
##
##       post.mean l-95% CI u-95% CI eff.samp
## units    0.3888   0.3657   0.4102     4500
##
##  Location effects: MeanCorner_hourly_scaled ~ PredatorCue * ExpDay + Hour + avg_length + avg_width
##
##                     post.mean   l-95% CI   u-95% CI eff.samp pMCMC
## (Intercept)        -0.6897010 -2.2397137  0.8164845     4500 0.388
## PredatorCue         0.2603623 -0.0038177  0.5378759     4969 0.056 .
## ExpDay              0.0006649 -0.0094043  0.0106234     4840 0.902
## avg_length         -0.0091690 -0.0361793  0.0204464     4500 0.520
## avg_width           0.1158265 -0.0607203  0.3033696     4500 0.211
## PredatorCue:ExpDay  0.0034404 -0.0099253  0.0173972     4855 0.627
## ---
## Signif. codes:  0 '***' 0.001 '**' 0.01 '*' 0.05 '.' 0.1 ' ' 1
```

```
# Without body size
model_MeanCorner_no_bodysize <- readRDS("model_MeanCorner_no_bodysize_9_24_25.rds")
# model_MeanCorner_no_bodysize <-
# MCMCglmm(MeanCorner_hourly_scaled ~ PredatorCue*ExpDay +
# Hour, random = ~us(1 + ExpDay):FishId + MomId, rcov =
```

```r
# ~units, family = 'gaussian', data = bodysize_only_data,
# prior = prior.univariate.FishId_slopes.MomId, nitt =
# 500000, burnin = 50000, thin = 100)
# saveRDS(model_MeanCorner_no_bodysize, file =
# 'model_MeanCorner_no_bodysize_9_24_25.rds')
summary(model_MeanCorner_no_bodysize)
```

```
##
##  Iterations = 50001:499901
##  Thinning interval  = 100
##  Sample size  = 4500
##
##  DIC: 4822.659
##
##  G-structure:  ~us(1 + ExpDay):FishId
##
##                                post.mean   l-95% CI  u-95% CI eff.samp
## (Intercept):(Intercept).FishId  0.3751239  0.2464921  0.511483     4500
## ExpDay:(Intercept).FishId      -0.0109127 -0.0165672 -0.005329     4500
## (Intercept):ExpDay.FishId      -0.0109127 -0.0165672 -0.005329     4500
## ExpDay:ExpDay.FishId            0.0009254  0.0006167  0.001288     4817
##
##               ~MomId
##
##       post.mean l-95% CI u-95% CI eff.samp
## MomId   0.01178 2.28e-09  0.04466     4500
##
##  R-structure:  ~units
##
##       post.mean l-95% CI u-95% CI eff.samp
## units    0.3889   0.3665   0.4129     4500
##
##  Location effects: MeanCorner_hourly_scaled ~ PredatorCue * ExpDay + Hour
##
##                     post.mean   l-95% CI   u-95% CI eff.samp  pMCMC
## (Intercept)        -0.0230486 -0.2379028  0.1699044     4312 0.8324
## PredatorCue         0.2428894 -0.0245389  0.5131067     4246 0.0733 .
## ExpDay              0.0007585 -0.0089933  0.0107748     4274 0.8809
## PredatorCue:ExpDay  0.0033196 -0.0110607  0.0166747     4500 0.6262
## ---
## Signif. codes:  0 '***' 0.001 '**' 0.01 '*' 0.05 '.' 0.1 ' ' 1
```

Including body size does not improve the model.

Our final model includes:
Fixed effects: PredatorCue*ExpDay, Hour (time of day).
Random effects: FishId (individual ID + slopes), MomId (mother's ID)

Set model:

```r
model <- model_MeanCorner_big_univariate_id_slopes_mom_noYearBatch
```

Model diagnostics

```r
plot(model$Sol)  # Plots looks good
```

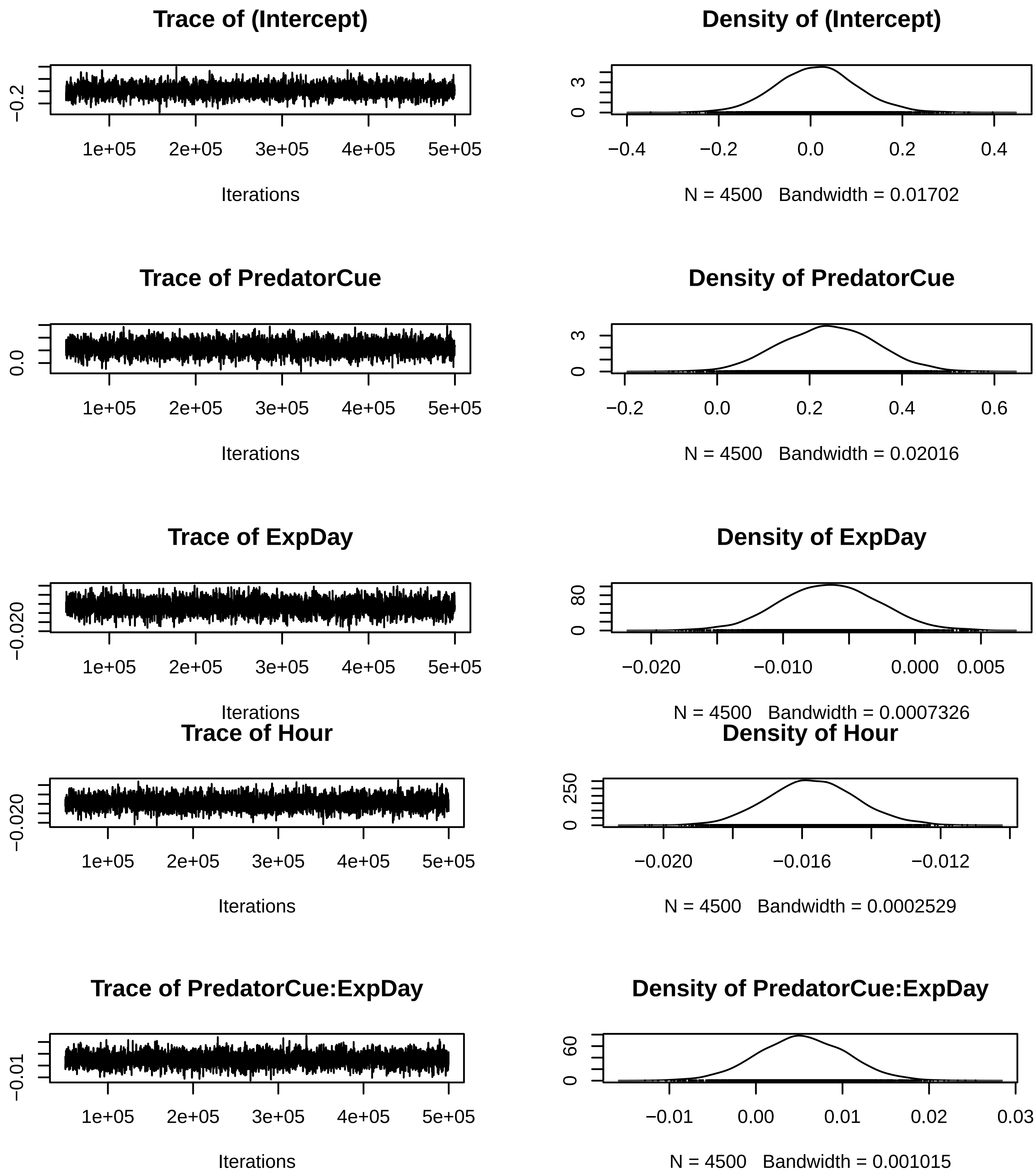


```
autocorr.diag(model$VCV)
```

```
##          (Intercept):(Intercept).FishId ExpDay:(Intercept).FishId
## Lag 0                       1.000000000               1.000000000
## Lag 100                     0.022088390              -0.015092834
## Lag 500                    -0.004066485              -0.003682675
## Lag 1000                    0.007510281              -0.019079537
## Lag 5000                    0.016343956               0.034028758
```

```
##          (Intercept):ExpDay.FishId ExpDay:ExpDay.FishId      MomId
## Lag 0                  1.000000000           1.00000000 1.00000000
## Lag 100               -0.015092834           0.01206269 0.13106582
## Lag 500               -0.003682675           0.03864782 0.08326263
## Lag 1000              -0.019079537           0.02432907 0.03137130
## Lag 5000               0.034028758           0.01126314 0.01119392
##          ExpDay_factor0.units ExpDay_factor1.units ExpDay_factor2.units
## Lag 0             1.000000000          1.000000000          1.000000000
## Lag 100          -0.007420011          0.001005291          0.003438985
## Lag 500           0.001049768         -0.004815219          0.002779077
## Lag 1000         -0.025180505          0.010745033         -0.007205698
## Lag 5000         -0.019159230          0.023284274          0.051484884
##          ExpDay_factor3.units ExpDay_factor4.units ExpDay_factor5.units
## Lag 0             1.000000000          1.000000000          1.000000000
## Lag 100           0.010133888          0.001673033         -0.001887230
## Lag 500          -0.007572546         -0.007959819          0.003738017
## Lag 1000         -0.001690078          0.026877359         -0.011805265
## Lag 5000          0.017908960          0.015900762         -0.014494133
##          ExpDay_factor6.units ExpDay_factor7.units ExpDay_factor8.units
## Lag 0             1.000000000           1.00000000          1.000000000
## Lag 100          -0.010791103          -0.01310018         -0.008865169
## Lag 500           0.002078225          -0.02211790         -0.003856896
## Lag 1000          0.018581564           0.02361305         -0.002209202
## Lag 5000          0.007856531           0.01795801          0.032771962
##          ExpDay_factor9.units ExpDay_factor10.units ExpDay_factor11.units
## Lag 0             1.000000000           1.000000000           1.000000000
## Lag 100          -0.007458760           0.009657144           0.018339040
## Lag 500          -0.011624790          -0.019448719          -0.002836280
## Lag 1000          0.015586695           0.024277235           0.029279437
## Lag 5000          0.006022957          -0.015750529          -0.009181678
##          ExpDay_factor12.units ExpDay_factor13.units ExpDay_factor14.units
## Lag 0             1.0000000000           1.000000000           1.000000000
## Lag 100          -0.0310675815           0.004340013           0.003481353
## Lag 500           0.0071400941           0.001318659          -0.010673792
## Lag 1000         -0.0172414030          -0.001160458          -0.010618491
## Lag 5000          0.0002461304          -0.013609702           0.009387256
##          ExpDay_factor15.units ExpDay_factor16.units ExpDay_factor17.units
## Lag 0               1.00000000           1.000000000           1.000000000
## Lag 100             0.02645843          -0.013148423           0.006513499
## Lag 500             0.02532477          -0.009534031          -0.026245141
## Lag 1000            0.01951649          -0.025349284          -0.009257071
## Lag 5000           -0.02329045           0.007506831           0.011853532
##          ExpDay_factor18.units ExpDay_factor19.units ExpDay_factor20.units
## Lag 0              1.000000000           1.000000000           1.000000000
## Lag 100            0.022180607           0.007826850           0.007211618
## Lag 500           -0.019759101           0.008021185           0.006202771
## Lag 1000          -0.005427162           0.014130424          -0.018648554
## Lag 5000          -0.002283387           0.021774012           0.016749972
##          ExpDay_factor21.units ExpDay_factor22.units ExpDay_factor23.units
## Lag 0              1.000000000           1.000000000           1.000000000
## Lag 100           -0.006025920          -0.003513910          -0.031589506
## Lag 500           -0.014853897           0.010211769           0.020199014
## Lag 1000           0.002176424          -0.004314593          -0.003656807
## Lag 5000           0.012878153          -0.026824730          -0.009117905
##          ExpDay_factor24.units ExpDay_factor25.units ExpDay_factor26.units
## Lag 0             1.0000000000           1.000000000           1.000000000
## Lag 100           0.0135438771          -0.008376101           0.003945133
## Lag 500           0.0205043719           0.019699209          -0.002501441
## Lag 1000          0.0008124355          -0.020289977           0.006425567
## Lag 5000         -0.0088504360          -0.006857573           0.034421984
##          ExpDay_factor27.units
```

```
## Lag 0             1.000000000
## Lag 100           0.008899206
## Lag 500           0.001308887
## Lag 1000          0.033263306
## Lag 5000          0.018786708
```

```r
autocorr.diag(model$Sol)  # MomId autocorrelation slightly high
```

```
##            (Intercept)  PredatorCue       ExpDay         Hour PredatorCue:ExpDay
## Lag 0     1.000000000  1.000000000  1.000000000  1.000000000       1.0000000000
## Lag 100   0.019343345  0.011595524  0.015524506 -0.005742188       0.0032269159
## Lag 500   0.006843563 -0.009395444 -0.008344377  0.019609225       0.0003849443
## Lag 1000 -0.025282622 -0.009366412 -0.010808342  0.007645317      -0.0096563795
## Lag 5000 -0.012543792 -0.018932050 -0.004616197  0.005460998       0.0050301151
```

```r
effectiveSize(model$VCV)
```

```
## (Intercept):(Intercept).FishId      ExpDay:(Intercept).FishId
##                       3672.995                       4500.000
##      (Intercept):ExpDay.FishId           ExpDay:ExpDay.FishId
##                       4500.000                       3876.996
##                          MomId           ExpDay_factor0.units
##                       1703.967                       4500.000
##           ExpDay_factor1.units           ExpDay_factor2.units
##                       4500.000                       4500.000
##           ExpDay_factor3.units           ExpDay_factor4.units
##                       4500.000                       4500.000
##           ExpDay_factor5.units           ExpDay_factor6.units
##                       4500.000                       4500.000
##           ExpDay_factor7.units           ExpDay_factor8.units
##                       4500.000                       4500.000
##           ExpDay_factor9.units          ExpDay_factor10.units
##                       5122.560                       4500.000
##          ExpDay_factor11.units          ExpDay_factor12.units
##                       4500.000                       4787.509
##          ExpDay_factor13.units          ExpDay_factor14.units
##                       4500.000                       4500.000
##          ExpDay_factor15.units          ExpDay_factor16.units
##                       4267.063                       4500.000
##          ExpDay_factor17.units          ExpDay_factor18.units
##                       4500.000                       4303.749
##          ExpDay_factor19.units          ExpDay_factor20.units
##                       4500.000                       4500.000
##          ExpDay_factor21.units          ExpDay_factor22.units
##                       4500.000                       4500.000
##          ExpDay_factor23.units          ExpDay_factor24.units
##                       4792.514                       4500.000
##          ExpDay_factor25.units          ExpDay_factor26.units
##                       4500.000                       4500.000
##          ExpDay_factor27.units
##                       4500.000
```

```r
effectiveSize(model$Sol)  # Effective sample size all high (>2000) -- looks good
```

```
##        (Intercept)        PredatorCue             ExpDay               Hour
##               4500               4500               4500               4500
## PredatorCue:ExpDay
##               4500
```

```r
# MomId autocorrelation is slightly high, so we'll rerun
# the model with more iterations and greater thinning

model_MeanCorner_big_univariate_id_slopes_mom_noYearBatch_more_iterations <- readRDS("model_MeanCorner_big_u
# model_MeanCorner_big_univariate_id_slopes_mom_noYearBatch_more_iterations
```

```r
# <- MCMCglmm(MeanCorner_hourly_scaled ~ PredatorCue*ExpDay
# + Hour, random = ~us(1 + ExpDay):FishId + MomId, rcov =
# ~idh(ExpDay_factor):units, family = 'gaussian', data =
# hourly_data, prior =
# prior.univariate.FishId_slopes.MomId.heteroResidual, nitt
# = 1200000, burnin = 120000, thin = 500)
# saveRDS(model_MeanCorner_big_univariate_id_slopes_mom_noYearBatch_more_iterations,
# file =
# 'model_MeanCorner_big_univariate_id_slopes_mom_noYearBatch_more_iterations_9_24_25.rds')
summary(model_MeanCorner_big_univariate_id_slopes_mom_noYearBatch_more_iterations)  #
```

```
##
##  Iterations = 150001:1499601
##  Thinning interval  = 400
##  Sample size  = 3375
##
##  DIC: 80051.1
##
##  G-structure:  ~us(1 + ExpDay):FishId
##
##                                 post.mean   l-95% CI   u-95% CI eff.samp
## (Intercept):(Intercept).FishId  0.2646286  0.1900334  0.3482902     3375
## ExpDay:(Intercept).FishId      -0.0056328 -0.0089058 -0.0030155     3375
## (Intercept):ExpDay.FishId      -0.0056328 -0.0089058 -0.0030155     3375
## ExpDay:ExpDay.FishId            0.0006214  0.0004352  0.0008062     3375
##
##               ~MomId
##
##       post.mean  l-95% CI u-95% CI eff.samp
## MomId   0.02885 8.255e-11  0.08457     1955
##
##  R-structure:  ~idh(ExpDay_factor):units
##
##                       post.mean l-95% CI u-95% CI eff.samp
## ExpDay_factor0.units     1.3419   1.2333   1.4543     3552
## ExpDay_factor1.units     1.0417   0.9657   1.1305     3375
## ExpDay_factor2.units     1.1596   1.0653   1.2563     3375
## ExpDay_factor3.units     1.0659   0.9844   1.1596     3566
## ExpDay_factor4.units     1.0128   0.9308   1.0964     3375
## ExpDay_factor5.units     0.6315   0.5802   0.6838     4101
## ExpDay_factor6.units     0.6710   0.6154   0.7223     3375
## ExpDay_factor7.units     0.6891   0.6352   0.7453     3375
## ExpDay_factor8.units     0.5174   0.4746   0.5561     3375
## ExpDay_factor9.units     0.7030   0.6491   0.7615     3375
## ExpDay_factor10.units    0.6665   0.6143   0.7257     3375
## ExpDay_factor11.units    0.5811   0.5374   0.6328     3375
## ExpDay_factor12.units    0.6782   0.6221   0.7350     3375
## ExpDay_factor13.units    0.5942   0.5475   0.6419     3375
## ExpDay_factor14.units    0.5331   0.4931   0.5787     3375
## ExpDay_factor15.units    0.6528   0.5978   0.7029     3375
## ExpDay_factor16.units    0.7332   0.6749   0.7913     3375
## ExpDay_factor17.units    0.6236   0.5758   0.6721     3323
## ExpDay_factor18.units    0.5963   0.5504   0.6464     3375
## ExpDay_factor19.units    0.5572   0.5120   0.6033     4312
## ExpDay_factor20.units    0.6228   0.5729   0.6730     3748
## ExpDay_factor21.units    0.6365   0.5878   0.6919     3614
## ExpDay_factor22.units    0.5676   0.5218   0.6147     3375
## ExpDay_factor23.units    0.4738   0.4351   0.5141     3375
## ExpDay_factor24.units    0.5385   0.4950   0.5822     3375
## ExpDay_factor25.units    0.4819   0.4456   0.5233     3375
## ExpDay_factor26.units    0.6145   0.5679   0.6674     3225
```

```
## ExpDay_factor27.units    0.5631   0.5156   0.6089     3375
##
##  Location effects: MeanCorner_hourly_scaled ~ PredatorCue * ExpDay + Hour
##
##                      post.mean   l-95% CI   u-95% CI eff.samp  pMCMC
## (Intercept)           0.0149942 -0.1590680  0.1837690     3375 0.8557
## PredatorCue           0.2413134  0.0262173  0.4341392     3375 0.0207 *
## ExpDay               -0.0065329 -0.0137255  0.0005822     3375 0.0764 .
## Hour                 -0.0156617 -0.0182390 -0.0130252     3028 <3e-04 ***
## PredatorCue:ExpDay    0.0054258 -0.0048108  0.0154604     3375 0.2839
## ---
## Signif. codes:  0 '***' 0.001 '**' 0.01 '*' 0.05 '.' 0.1 ' ' 1
```

```r
model <- model_MeanCorner_big_univariate_id_slopes_mom_noYearBatch_more_iterations

plot(model$Sol)  # Plots looks good
```

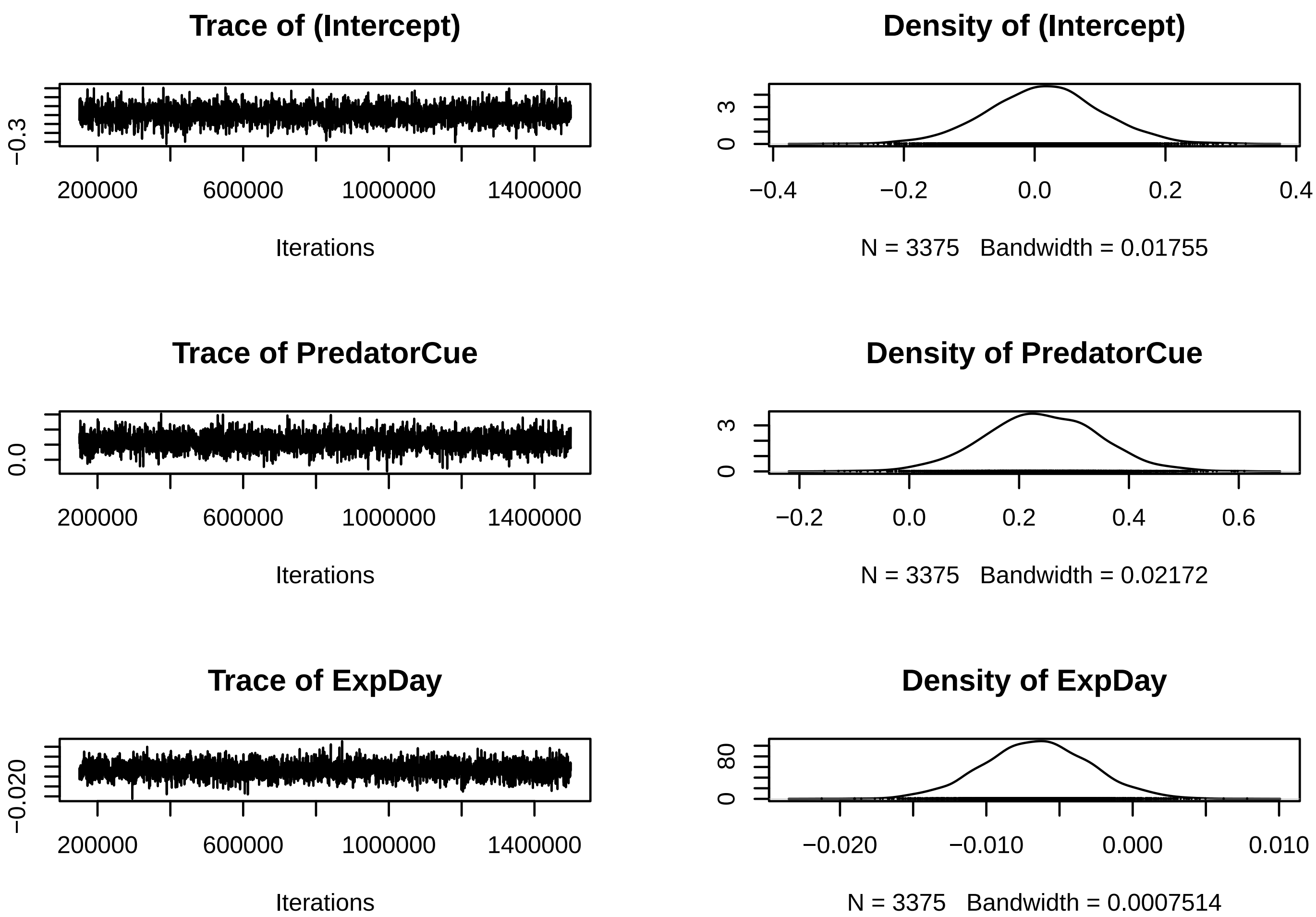

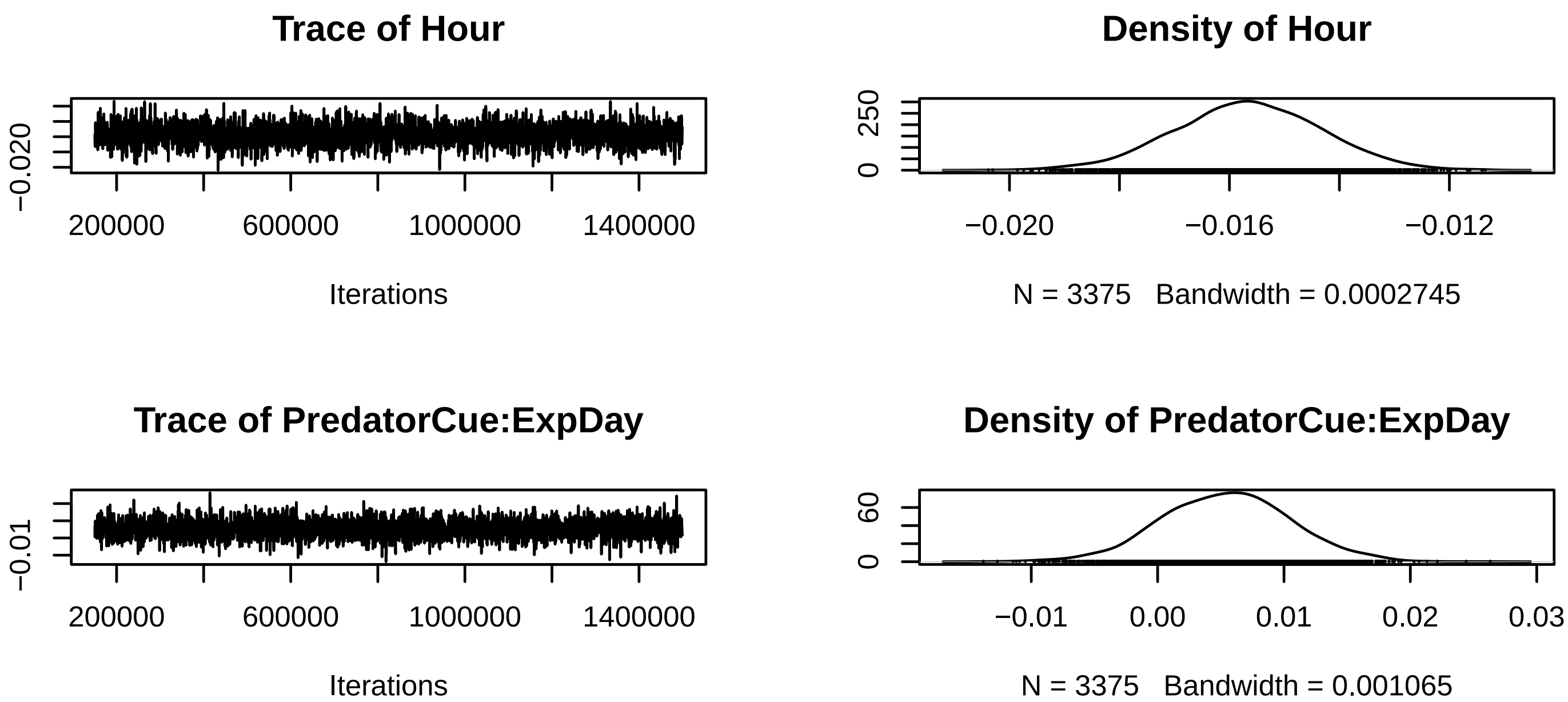


```r
autocorr.diag(model$VCV)
```

```
##           (Intercept):(Intercept).FishId ExpDay:(Intercept).FishId
## Lag 0                        1.000000000               1.000000000
## Lag 400                      0.009425310              -0.004918154
## Lag 2000                     0.025421063              -0.012534934
## Lag 4000                    -0.007754193              -0.010053529
## Lag 20000                   -0.009967184               0.006991053
##           (Intercept):ExpDay.FishId ExpDay:ExpDay.FishId       MomId
## Lag 0                   1.000000000          1.000000000  1.00000000
## Lag 400                -0.004918154         -0.014944977  0.17684600
## Lag 2000               -0.012534934         -0.029397488  0.01060671
## Lag 4000               -0.010053529         -0.005485285 -0.01251880
## Lag 20000               0.006991053          0.006387203 -0.01235316
##           ExpDay_factor0.units ExpDay_factor1.units ExpDay_factor2.units
## Lag 0               1.00000000          1.000000000          1.000000000
## Lag 400            -0.02575924          0.001817758         -0.004925793
## Lag 2000           -0.03002714          0.026132931          0.002053879
## Lag 4000            0.01893916         -0.014149888          0.005053968
## Lag 20000          -0.02244907          0.010829412          0.020243451
##           ExpDay_factor3.units ExpDay_factor4.units ExpDay_factor5.units
## Lag 0              1.000000000          1.000000000         1.0000000000
## Lag 400           -0.027655000         -0.023825807         0.0083888998
## Lag 2000           0.006785978         -0.006984138        -0.0008306623
## Lag 4000           0.018129652         -0.006874241        -0.0150799227
## Lag 20000         -0.044774218         -0.031174359         0.0033280834
##           ExpDay_factor6.units ExpDay_factor7.units ExpDay_factor8.units
## Lag 0              1.000000000          1.000000000         1.0000000000
## Lag 400           -0.006829586          0.003109347        -0.0003262755
## Lag 2000          -0.003097875          0.017763334         0.0095860184
## Lag 4000          -0.023382889         -0.016968631        -0.0341912639
## Lag 20000         -0.021266918          0.010549807        -0.0034434376
##           ExpDay_factor9.units ExpDay_factor10.units ExpDay_factor11.units
## Lag 0             1.0000000000           1.000000000          1.0000000000
## Lag 400          -0.0161746570           0.007543045         -0.0144338270
## Lag 2000          0.0050894194           0.009348624          0.0179697134
## Lag 4000          0.0004595208          -0.016124222         -0.0003091593
## Lag 20000         0.0004513960           0.022612628          0.0020844722
##           ExpDay_factor12.units ExpDay_factor13.units ExpDay_factor14.units
## Lag 0               1.000000000           1.000000000           1.000000000
```

```
## Lag 400            -0.020736460          -0.006304328          -0.002195128
## Lag 2000            0.006451553          -0.005202623           0.023667625
## Lag 4000            0.008541181          -0.002431438          -0.006415646
## Lag 20000           0.033805959           0.010514710           0.011356768
##           ExpDay_factor15.units ExpDay_factor16.units ExpDay_factor17.units
## Lag 0               1.000000000           1.000000000          1.000000e+00
## Lag 400            -0.004248845          -0.007448256          3.306016e-02
## Lag 2000            0.024716746          -0.016469469         -5.720849e-03
## Lag 4000            0.013252648          -0.028260008         -2.978081e-03
## Lag 20000          -0.021218638           0.039051031          3.867992e-05
##           ExpDay_factor18.units ExpDay_factor19.units ExpDay_factor20.units
## Lag 0               1.000000000           1.000000000           1.000000000
## Lag 400            -0.003791579          -0.024287240          -0.006008388
## Lag 2000           -0.018533773          -0.006000248          -0.016899171
## Lag 4000           -0.010940268          -0.042808233          -0.021539839
## Lag 20000           0.023061841           0.018158919          -0.021044136
##           ExpDay_factor21.units ExpDay_factor22.units ExpDay_factor23.units
## Lag 0              1.0000000000          1.0000000000           1.000000000
## Lag 400           -0.0343446816         -0.0194393867           0.008541365
## Lag 2000           0.0002861100          0.0159528304          -0.027345381
## Lag 4000           0.0250646087          0.0006469188          -0.022260575
## Lag 20000         -0.0005210326         -0.0020714484          -0.003859484
##           ExpDay_factor24.units ExpDay_factor25.units ExpDay_factor26.units
## Lag 0               1.000000000           1.000000000           1.000000000
## Lag 400            -0.008700727          -0.005432195          -0.011922135
## Lag 2000           -0.022392250           0.017126924          -0.014709261
## Lag 4000            0.010007746           0.009794360           0.009549765
## Lag 20000           0.007542628           0.002469855          -0.006685318
##           ExpDay_factor27.units
## Lag 0               1.000000000
## Lag 400            -0.015639720
## Lag 2000           -0.001869654
## Lag 4000           -0.005586041
## Lag 20000           0.052482457
```

```r
autocorr.diag(model$Sol)  # MomId autocorrelation all below <0.1
```

```
##           (Intercept)  PredatorCue       ExpDay         Hour PredatorCue:ExpDay
## Lag 0      1.00000000  1.000000000  1.000000000  1.000000000       1.0000000000
## Lag 400   -0.01621754  0.001162910  0.015532218  0.024600418       0.0009977582
## Lag 2000  -0.01801973 -0.021007256 -0.009101057  0.003256313       0.0102094931
## Lag 4000   0.01036589 -0.001076734 -0.009807980  0.012846079      -0.0010436874
## Lag 20000 -0.02309774  0.008304064  0.013341121 -0.009637404       0.0069480502
```

```r
effectiveSize(model$VCV)
```

```
## (Intercept):(Intercept).FishId      ExpDay:(Intercept).FishId
##                       3375.000                       3375.000
##      (Intercept):ExpDay.FishId           ExpDay:ExpDay.FishId
##                       3375.000                       3375.000
##                          MomId           ExpDay_factor0.units
##                       1955.365                       3552.419
##           ExpDay_factor1.units           ExpDay_factor2.units
##                       3375.000                       3375.000
##           ExpDay_factor3.units           ExpDay_factor4.units
##                       3565.923                       3375.000
##           ExpDay_factor5.units           ExpDay_factor6.units
##                       4101.062                       3375.000
##           ExpDay_factor7.units           ExpDay_factor8.units
##                       3375.000                       3375.000
##           ExpDay_factor9.units          ExpDay_factor10.units
##                       3375.000                       3375.000
##          ExpDay_factor11.units          ExpDay_factor12.units
```

```
##                      3375.000                       3375.000
##          ExpDay_factor13.units          ExpDay_factor14.units
##                      3375.000                       3375.000
##          ExpDay_factor15.units          ExpDay_factor16.units
##                      3375.000                       3375.000
##          ExpDay_factor17.units          ExpDay_factor18.units
##                      3323.066                       3375.000
##          ExpDay_factor19.units          ExpDay_factor20.units
##                      4312.187                       3748.096
##          ExpDay_factor21.units          ExpDay_factor22.units
##                      3614.000                       3375.000
##          ExpDay_factor23.units          ExpDay_factor24.units
##                      3375.000                       3375.000
##          ExpDay_factor25.units          ExpDay_factor26.units
##                      3375.000                       3224.938
##          ExpDay_factor27.units
##                      3375.000
```

```
effectiveSize(model$Sol)  # Effective sample sizes all high -- looks good
```

```
##        (Intercept)        PredatorCue             ExpDay               Hour
##           3375.000           3375.000           3375.000           3028.496
## PredatorCue:ExpDay
##           3375.000
```

Model outputs

```
summary(model)
```

```
##
##  Iterations = 150001:1499601
##  Thinning interval  = 400
##  Sample size  = 3375
##
##  DIC: 80051.1
##
##  G-structure:  ~us(1 + ExpDay):FishId
##
##                                  post.mean   l-95% CI   u-95% CI eff.samp
## (Intercept):(Intercept).FishId  0.2646286  0.1900334  0.3482902     3375
## ExpDay:(Intercept).FishId      -0.0056328 -0.0089058 -0.0030155     3375
## (Intercept):ExpDay.FishId      -0.0056328 -0.0089058 -0.0030155     3375
## ExpDay:ExpDay.FishId            0.0006214  0.0004352  0.0008062     3375
##
##               ~MomId
##
##       post.mean  l-95% CI u-95% CI eff.samp
## MomId   0.02885 8.255e-11  0.08457     1955
##
##  R-structure:  ~idh(ExpDay_factor):units
##
##                       post.mean l-95% CI u-95% CI eff.samp
## ExpDay_factor0.units     1.3419   1.2333   1.4543     3552
## ExpDay_factor1.units     1.0417   0.9657   1.1305     3375
## ExpDay_factor2.units     1.1596   1.0653   1.2563     3375
## ExpDay_factor3.units     1.0659   0.9844   1.1596     3566
## ExpDay_factor4.units     1.0128   0.9308   1.0964     3375
## ExpDay_factor5.units     0.6315   0.5802   0.6838     4101
## ExpDay_factor6.units     0.6710   0.6154   0.7223     3375
## ExpDay_factor7.units     0.6891   0.6352   0.7453     3375
## ExpDay_factor8.units     0.5174   0.4746   0.5561     3375
## ExpDay_factor9.units     0.7030   0.6491   0.7615     3375
```

```
## ExpDay_factor10.units    0.6665   0.6143   0.7257     3375
## ExpDay_factor11.units    0.5811   0.5374   0.6328     3375
## ExpDay_factor12.units    0.6782   0.6221   0.7350     3375
## ExpDay_factor13.units    0.5942   0.5475   0.6419     3375
## ExpDay_factor14.units    0.5331   0.4931   0.5787     3375
## ExpDay_factor15.units    0.6528   0.5978   0.7029     3375
## ExpDay_factor16.units    0.7332   0.6749   0.7913     3375
## ExpDay_factor17.units    0.6236   0.5758   0.6721     3323
## ExpDay_factor18.units    0.5963   0.5504   0.6464     3375
## ExpDay_factor19.units    0.5572   0.5120   0.6033     4312
## ExpDay_factor20.units    0.6228   0.5729   0.6730     3748
## ExpDay_factor21.units    0.6365   0.5878   0.6919     3614
## ExpDay_factor22.units    0.5676   0.5218   0.6147     3375
## ExpDay_factor23.units    0.4738   0.4351   0.5141     3375
## ExpDay_factor24.units    0.5385   0.4950   0.5822     3375
## ExpDay_factor25.units    0.4819   0.4456   0.5233     3375
## ExpDay_factor26.units    0.6145   0.5679   0.6674     3225
## ExpDay_factor27.units    0.5631   0.5156   0.6089     3375
##
##  Location effects: MeanCorner_hourly_scaled ~ PredatorCue * ExpDay + Hour
##
##                       post.mean   l-95% CI   u-95% CI eff.samp  pMCMC
## (Intercept)          0.0149942 -0.1590680  0.1837690     3375 0.8557
## PredatorCue          0.2413134  0.0262173  0.4341392     3375 0.0207 *
## ExpDay              -0.0065329 -0.0137255  0.0005822     3375 0.0764 .
## Hour                -0.0156617 -0.0182390 -0.0130252     3028 <3e-04 ***
## PredatorCue:ExpDay   0.0054258 -0.0048108  0.0154604     3375 0.2839
## ---
## Signif. codes:  0 '***' 0.001 '**' 0.01 '*' 0.05 '.' 0.1 ' ' 1
```

```r
summary(model$Sol)
```

```
##
## Iterations = 150001:1499601
## Thinning interval = 400
## Number of chains = 1
## Sample size per chain = 3375
##
## 1. Empirical mean and standard deviation for each variable,
##    plus standard error of the mean:
##
##                          Mean       SD  Naive SE Time-series SE
## (Intercept)          0.014994 0.087242 1.502e-03      1.502e-03
## PredatorCue          0.241313 0.104061 1.791e-03      1.791e-03
## ExpDay              -0.006533 0.003643 6.272e-05      6.272e-05
## Hour                -0.015662 0.001331 2.291e-05      2.418e-05
## PredatorCue:ExpDay   0.005426 0.005101 8.781e-05      8.781e-05
##
## 2. Quantiles for each variable:
##
##                          2.5%       25%       50%       75%      97.5%
## (Intercept)         -0.158300 -0.041926  0.015748  0.070700  0.1857004
## PredatorCue          0.034743  0.172134  0.241012  0.312312  0.4473251
## ExpDay              -0.013692 -0.008919 -0.006511 -0.004096  0.0006178
## Hour                -0.018340 -0.016532 -0.015671 -0.014769 -0.0130789
## PredatorCue:ExpDay -0.004745  0.001915  0.005490  0.008767  0.0156060
```

```r
summary(model$VCV)
```

```
##
## Iterations = 150001:1499601
## Thinning interval = 400
## Number of chains = 1
```

```
## Sample size per chain = 3375
##
## 1. Empirical mean and standard deviation for each variable,
##    plus standard error of the mean:
##
##                                    Mean        SD  Naive SE Time-series SE
## (Intercept):(Intercept).FishId  0.2646286 4.170e-02 7.177e-04      7.177e-04
## ExpDay:(Intercept).FishId      -0.0056328 1.528e-03 2.630e-05      2.630e-05
## (Intercept):ExpDay.FishId      -0.0056328 1.528e-03 2.630e-05      2.630e-05
## ExpDay:ExpDay.FishId            0.0006214 9.696e-05 1.669e-06      1.669e-06
## MomId                           0.0288493 3.159e-02 5.438e-04      7.145e-04
## ExpDay_factor0.units            1.3419112 5.731e-02 9.865e-04      9.615e-04
## ExpDay_factor1.units            1.0417041 4.233e-02 7.287e-04      7.287e-04
## ExpDay_factor2.units            1.1595883 4.806e-02 8.273e-04      8.273e-04
## ExpDay_factor3.units            1.0659067 4.435e-02 7.634e-04      7.427e-04
## ExpDay_factor4.units            1.0128149 4.193e-02 7.217e-04      7.217e-04
## ExpDay_factor5.units            0.6314990 2.624e-02 4.517e-04      4.098e-04
## ExpDay_factor6.units            0.6709926 2.779e-02 4.783e-04      4.783e-04
## ExpDay_factor7.units            0.6890677 2.831e-02 4.873e-04      4.873e-04
## ExpDay_factor8.units            0.5173525 2.111e-02 3.634e-04      3.634e-04
## ExpDay_factor9.units            0.7030071 2.913e-02 5.015e-04      5.015e-04
## ExpDay_factor10.units           0.6665300 2.840e-02 4.888e-04      4.888e-04
## ExpDay_factor11.units           0.5811189 2.447e-02 4.212e-04      4.212e-04
## ExpDay_factor12.units           0.6782184 2.870e-02 4.941e-04      4.941e-04
## ExpDay_factor13.units           0.5942127 2.437e-02 4.194e-04      4.194e-04
## ExpDay_factor14.units           0.5330750 2.194e-02 3.777e-04      3.777e-04
## ExpDay_factor15.units           0.6528383 2.740e-02 4.716e-04      4.716e-04
## ExpDay_factor16.units           0.7331792 3.032e-02 5.218e-04      5.218e-04
## ExpDay_factor17.units           0.6235731 2.487e-02 4.281e-04      4.314e-04
## ExpDay_factor18.units           0.5962639 2.469e-02 4.250e-04      4.250e-04
## ExpDay_factor19.units           0.5571966 2.344e-02 4.034e-04      3.569e-04
## ExpDay_factor20.units           0.6227997 2.620e-02 4.510e-04      4.279e-04
## ExpDay_factor21.units           0.6364936 2.672e-02 4.599e-04      4.444e-04
## ExpDay_factor22.units           0.5676012 2.364e-02 4.070e-04      4.070e-04
## ExpDay_factor23.units           0.4738055 2.029e-02 3.492e-04      3.492e-04
## ExpDay_factor24.units           0.5385390 2.275e-02 3.916e-04      3.916e-04
## ExpDay_factor25.units           0.4818843 1.996e-02 3.435e-04      3.435e-04
## ExpDay_factor26.units           0.6145322 2.561e-02 4.408e-04      4.509e-04
## ExpDay_factor27.units           0.5630648 2.427e-02 4.177e-04      4.177e-04
##
## 2. Quantiles for each variable:
##
##                                    2.5%        25%       50%        75%
## (Intercept):(Intercept).FishId  0.1932284  0.2353431  0.259970  0.2904330
## ExpDay:(Intercept).FishId      -0.0088767 -0.0065830 -0.005547 -0.0045691
## (Intercept):ExpDay.FishId      -0.0088767 -0.0065830 -0.005547 -0.0045691
## ExpDay:ExpDay.FishId            0.0004540  0.0005541  0.000611  0.0006789
## MomId                           0.0001386  0.0082386  0.020213  0.0393195
## ExpDay_factor0.units            1.2349912  1.3023581  1.339597  1.3782549
## ExpDay_factor1.units            0.9639391  1.0125785  1.040408  1.0693586
## ExpDay_factor2.units            1.0679039  1.1274751  1.158605  1.1907322
## ExpDay_factor3.units            0.9828481  1.0357115  1.064374  1.0943359
## ExpDay_factor4.units            0.9332154  0.9835354  1.011394  1.0398855
## ExpDay_factor5.units            0.5815868  0.6140620  0.630542  0.6484925
## ExpDay_factor6.units            0.6186205  0.6521302  0.670234  0.6888754
## ExpDay_factor7.units            0.6363912  0.6701398  0.687893  0.7068791
## ExpDay_factor8.units            0.4776402  0.5029254  0.516811  0.5310379
## ExpDay_factor9.units            0.6492873  0.6827433  0.701594  0.7220391
## ExpDay_factor10.units           0.6126841  0.6464093  0.665701  0.6852235
## ExpDay_factor11.units           0.5358247  0.5643947  0.580010  0.5968162
## ExpDay_factor12.units           0.6240668  0.6584629  0.677240  0.6971637
```

```
## ExpDay_factor13.units           0.5488627  0.5772611  0.593488  0.6104611
## ExpDay_factor14.units           0.4921414  0.5174123  0.532686  0.5473263
## ExpDay_factor15.units           0.6015899  0.6340599  0.652498  0.6708364
## ExpDay_factor16.units           0.6757814  0.7116701  0.732689  0.7530441
## ExpDay_factor17.units           0.5763910  0.6060414  0.623224  0.6407474
## ExpDay_factor18.units           0.5502662  0.5789555  0.595358  0.6120838
## ExpDay_factor19.units           0.5131939  0.5412946  0.556486  0.5728131
## ExpDay_factor20.units           0.5734135  0.6045466  0.622261  0.6404932
## ExpDay_factor21.units           0.5856691  0.6185192  0.635533  0.6538515
## ExpDay_factor22.units           0.5224516  0.5514162  0.566885  0.5831797
## ExpDay_factor23.units           0.4361234  0.4599927  0.472611  0.4869268
## ExpDay_factor24.units           0.4964042  0.5224675  0.537697  0.5541118
## ExpDay_factor25.units           0.4447254  0.4684279  0.481514  0.4945053
## ExpDay_factor26.units           0.5676378  0.5963836  0.613764  0.6315956
## ExpDay_factor27.units           0.5164652  0.5466869  0.563215  0.5788132
##                                     97.5%
## (Intercept):(Intercept).FishId  0.3543581
## ExpDay:(Intercept).FishId      -0.0029696
## (Intercept):ExpDay.FishId      -0.0029696
## ExpDay:ExpDay.FishId            0.0008295
## MomId                           0.1049323
## ExpDay_factor0.units            1.4581086
## ExpDay_factor1.units            1.1290984
## ExpDay_factor2.units            1.2597183
## ExpDay_factor3.units            1.1588646
## ExpDay_factor4.units            1.1004520
## ExpDay_factor5.units            0.6860185
## ExpDay_factor6.units            0.7267622
## ExpDay_factor7.units            0.7471746
## ExpDay_factor8.units            0.5609729
## ExpDay_factor9.units            0.7622906
## ExpDay_factor10.units           0.7243832
## ExpDay_factor11.units           0.6315771
## ExpDay_factor12.units           0.7382344
## ExpDay_factor13.units           0.6447405
## ExpDay_factor14.units           0.5780743
## ExpDay_factor15.units           0.7075598
## ExpDay_factor16.units           0.7932256
## ExpDay_factor17.units           0.6731329
## ExpDay_factor18.units           0.6462832
## ExpDay_factor19.units           0.6050510
## ExpDay_factor20.units           0.6742653
## ExpDay_factor21.units           0.6902429
## ExpDay_factor22.units           0.6170325
## ExpDay_factor23.units           0.5160210
## ExpDay_factor24.units           0.5840186
## ExpDay_factor25.units           0.5225144
## ExpDay_factor26.units           0.6672731
## ExpDay_factor27.units           0.6103958
```

Calculating probability of direction

To aid in interpreting treatment differences, in addition to confidence interval overlap, we can look at probability of direction (pd). Pd values can be analogous to p-values in frequentist statistics.

Calculate probability of direction (pd) for treatment (PredatorCue):

```
# Treatment
post_diff <- model$Sol[, "PredatorCue"]
pd <- mean(post_diff > 0)
pd  # 0.990
```

```
## [1] 0.9896296
```

```r
ppvalue_comparison <- pd_to_p(pd = pd)
ppvalue_comparison  # 0.021
```

```
## [1] 0.02074074
```

```r
# Treatment x Time
post_diff <- model$Sol[, "PredatorCue:ExpDay"]
pd <- mean(post_diff > 0)
pd  # 0.858
```

```
## [1] 0.8580741
```

```r
ppvalue_comparison <- pd_to_p(pd = pd)
ppvalue_comparison  # 0.284
```

```
## [1] 0.2838519
```

Now we can plot refuge use over time

```r
## Plotting trait over time
colors <- c(`0` = "#1709b5", `1` = "#ed5715")

# Mean Corner over time
plot_name <- ggplot(day_only_data, aes(x = (ExpDay + 1), y = MeanCorner,
    color = factor(PredatorCue))) + geom_line(aes(group = FishId),
    linewidth = 0.8, alpha = 0.16) + stat_summary(fun = mean,
    geom = "line", aes(group = factor(PredatorCue)), linewidth = 1.5,
    linetype = "solid") + scale_color_manual(values = colors) +
    scale_x_continuous(limits = c(1, 28), breaks = seq(1, max(28,
        na.rm = TRUE), by = 3)) + scale_y_continuous(limits = c(0.1,
    0.82), breaks = seq(0.1, max(0.82, na.rm = TRUE), by = 0.1)) +
    theme_classic() + theme(legend.position = "none", axis.line = element_line(linewidth = 0.5,
    colour = "black"), axis.ticks = element_line(colour = "black"),
    axis.text = element_text(size = 14, colour = "black"), axis.title = element_text(size = 18,
        colour = "black"), plot.title = element_text(size = 0)) +
    labs(x = "Day", y = "Refuge use (prop. time)")
plot_name
```

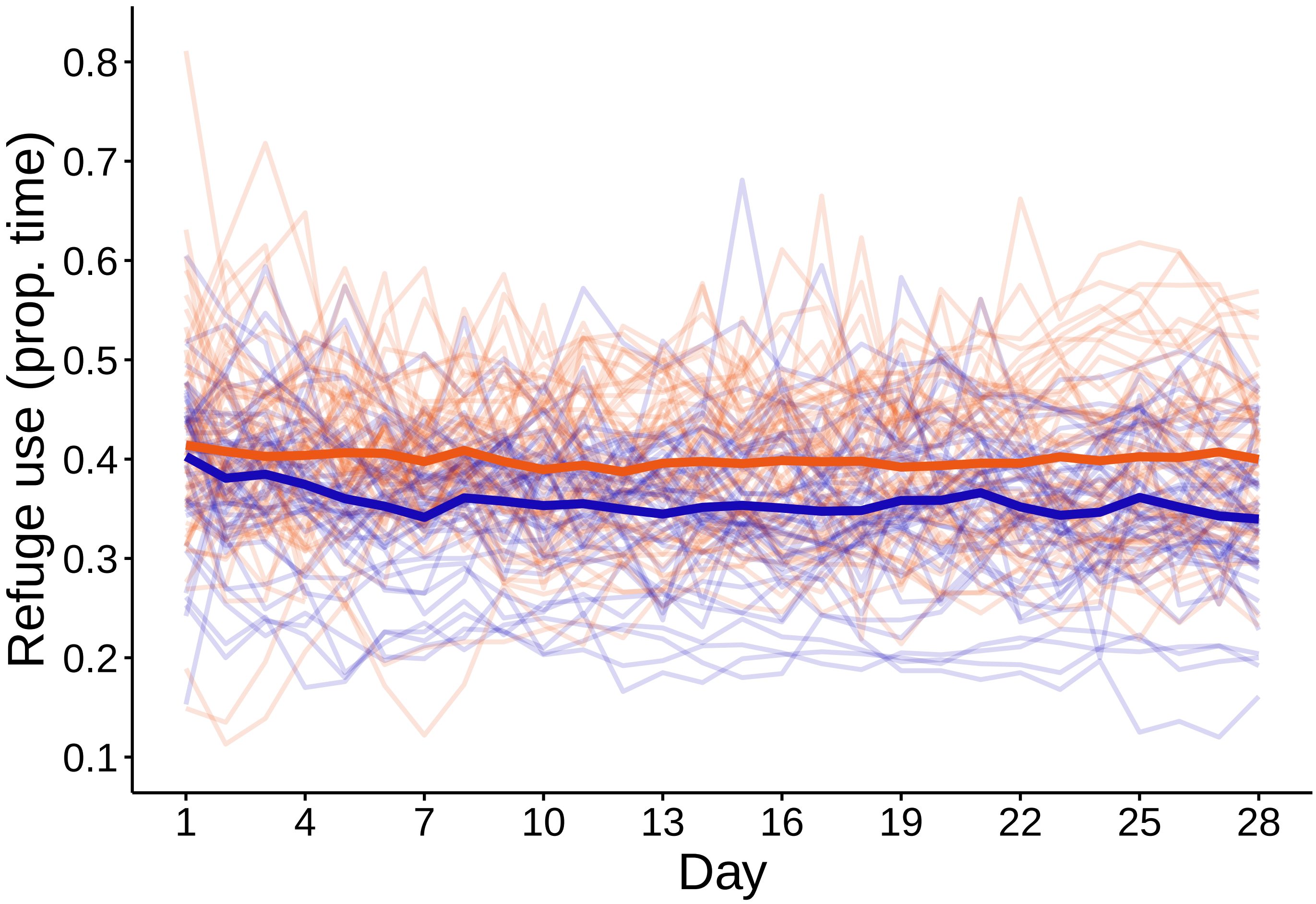


```r
ggsave("refuge_use_over_time_nonscaled.pdf", plot = plot_name,
    dpi = 600)
```

```
## Saving 6.5 x 4.5 in image
```

Comparing behavior on day 1

Now we can run the model on a subset of the data to compare behavior on day 1 between treatments:

```r
# Make day 1 subset
day1_hourly_data <- subset(hourly_data, ExpDay == 0)

### Model selection to see whether fixed effects are still
### relevant when only looking at day 1

# Including YearBatch and Hour
model_MeanCorner_day1_YearBach_Hour <- readRDS("model_MeanCorner_day1_YearBatch_Hour_10_14_25.rds")
# model_MeanCorner_day1_YearBach_Hour <-
# MCMCglmm(MeanCorner_hourly_scaled ~ PredatorCue +
# YearBatch + Hour, random = ~FishId + MomId, rcov =
# ~units, family = 'gaussian', data = day1_hourly_data,
# prior = prior.univariate.FishId.MomId, nitt = 500000,
# burnin = 50000, thin = 100)
# saveRDS(model_MeanCorner_day1_YearBach_Hour, file =
# 'model_MeanCorner_day1_YearBatch_Hour_10_14_25.rds')
summary(model_MeanCorner_day1_YearBach_Hour)
```

```
##
##  Iterations = 50001:499901
##  Thinning interval  = 100
```

```
##  Sample size  = 4500
##
##  DIC: 3444.567
##
##  G-structure:  ~FishId
##
##        post.mean l-95% CI u-95% CI eff.samp
## FishId    0.5175   0.3544   0.6996     4500
##
##               ~MomId
##
##       post.mean  l-95% CI u-95% CI eff.samp
## MomId   0.04414 1.518e-07   0.1557     4500
##
##  R-structure:  ~units
##
##       post.mean l-95% CI u-95% CI eff.samp
## units     0.969   0.8894    1.051     4500
##
##  Location effects: MeanCorner_hourly_scaled ~ PredatorCue + YearBatch + Hour
##
##             post.mean  l-95% CI  u-95% CI eff.samp pMCMC
## (Intercept)  0.327466 -0.148740  0.829071     4500 0.181
## PredatorCue  0.032422 -0.268790  0.342083     4500 0.823
## YearBatch   -0.064077 -0.254018  0.112710     4500 0.474
## Hour         0.007708 -0.008563  0.023425     4703 0.357
```

```
# Including only YearBatch
model_MeanCorner_day1_YearBach <- readRDS("model_MeanCorner_day1_YearBatch_10_14_25.rds")
# model_MeanCorner_day1_YearBach <-
# MCMCglmm(MeanCorner_hourly_scaled ~ PredatorCue +
# YearBatch, random = ~FishId + MomId, rcov = ~units,
# family = 'gaussian', data = day1_hourly_data, prior =
# prior.univariate.FishId.MomId, nitt = 500000, burnin =
# 50000, thin = 100)
# saveRDS(model_MeanCorner_day1_YearBach, file =
# 'model_MeanCorner_day1_YearBatch_10_14_25.rds')
summary(model_MeanCorner_day1_YearBach)
```

```
##
##  Iterations = 50001:499901
##  Thinning interval  = 100
##  Sample size  = 4500
##
##  DIC: 3443.48
##
##  G-structure:  ~FishId
##
##        post.mean l-95% CI u-95% CI eff.samp
## FishId    0.5198   0.3523   0.6963     4500
##
##               ~MomId
##
##       post.mean  l-95% CI u-95% CI eff.samp
## MomId   0.04323 6.158e-09   0.1455     4238
##
##  R-structure:  ~units
##
##       post.mean l-95% CI u-95% CI eff.samp
## units      0.97    0.884    1.047     4892
##
##  Location effects: MeanCorner_hourly_scaled ~ PredatorCue + YearBatch
```

```
##
##              post.mean l-95% CI u-95% CI eff.samp pMCMC
## (Intercept)   0.37117 -0.09987  0.85902     4500 0.128
## PredatorCue   0.03198 -0.27950  0.33184     4500 0.839
## YearBatch    -0.06462 -0.24720  0.12770     4500 0.465
```

```r
# Including only Hour
model_MeanCorner_day1_Hour <- readRDS("model_MeanCorner_day1_Hour_10_14_25.rds")
# model_MeanCorner_day1_Hour <-
# MCMCglmm(MeanCorner_hourly_scaled ~ PredatorCue + Hour,
# random = ~FishId + MomId, rcov = ~units, family =
# 'gaussian', data = day1_hourly_data, prior =
# prior.univariate.FishId.MomId, nitt = 500000, burnin =
# 50000, thin = 100) saveRDS(model_MeanCorner_day1_Hour,
# file = 'model_MeanCorner_day1_Hour_10_14_25.rds')
summary(model_MeanCorner_day1_Hour)
```

```
##
##  Iterations = 50001:499901
##  Thinning interval  = 100
##  Sample size  = 4500
##
##  DIC: 3444.454
##
##  G-structure:  ~FishId
##
##        post.mean l-95% CI u-95% CI eff.samp
## FishId    0.5163   0.3421   0.6885     4500
##
##                ~MomId
##
##       post.mean  l-95% CI u-95% CI eff.samp
## MomId   0.03964 3.065e-11   0.1364     4500
##
##  R-structure:  ~units
##
##       post.mean l-95% CI u-95% CI eff.samp
## units    0.9704    0.885    1.047     4500
##
##  Location effects: MeanCorner_hourly_scaled ~ PredatorCue + Hour
##
##              post.mean  l-95% CI  u-95% CI eff.samp pMCMC
## (Intercept)  0.182167 -0.064686  0.449691     4500 0.160
## PredatorCue  0.028104 -0.271168  0.343475     4500 0.865
## Hour         0.008060 -0.009399  0.023261     4832 0.356
```

```r
# Excluding YearBatch and Hour
model_MeanCorner_day1 <- readRDS("model_MeanCorner_day1_10_14_25.rds")
# model_MeanCorner_day1 <-
# MCMCglmm(MeanCorner_hourly_scaled ~ PredatorCue, random =
# ~FishId + MomId, rcov = ~units, family = 'gaussian', data
# = day1_hourly_data, prior =
# prior.univariate.FishId.MomId, nitt = 500000, burnin =
# 50000, thin = 100) saveRDS(model_MeanCorner_day1, file =
# 'model_MeanCorner_day1_10_14_25.rds')
summary(model_MeanCorner_day1)  # Best fit
```

```
##
##  Iterations = 50001:499901
##  Thinning interval  = 100
##  Sample size  = 4500
##
##  DIC: 3443.328
```

```
##
##  G-structure:  ~FishId
##
##        post.mean l-95% CI u-95% CI eff.samp
## FishId    0.5164   0.3576   0.6989     4500
##
##                ~MomId
##
##       post.mean  l-95% CI u-95% CI eff.samp
## MomId    0.0404 4.556e-10   0.1392     3862
##
##  R-structure:  ~units
##
##       post.mean l-95% CI u-95% CI eff.samp
## units    0.9693   0.8876    1.047     4500
##
##  Location effects: MeanCorner_hourly_scaled ~ PredatorCue
##
##             post.mean  l-95% CI  u-95% CI eff.samp  pMCMC
## (Intercept)  0.223495 -0.009375  0.484027     4500 0.0782 .
## PredatorCue  0.031077 -0.282889  0.329498     4077 0.8489
## ---
## Signif. codes:  0 '***' 0.001 '**' 0.01 '*' 0.05 '.' 0.1 ' ' 1
```

```r
day1_model <- model_MeanCorner_day1
summary(day1_model)
```

```
##
##  Iterations = 50001:499901
##  Thinning interval  = 100
##  Sample size  = 4500
##
##  DIC: 3443.328
##
##  G-structure:  ~FishId
##
##        post.mean l-95% CI u-95% CI eff.samp
## FishId    0.5164   0.3576   0.6989     4500
##
##                ~MomId
##
##       post.mean  l-95% CI u-95% CI eff.samp
## MomId    0.0404 4.556e-10   0.1392     3862
##
##  R-structure:  ~units
##
##       post.mean l-95% CI u-95% CI eff.samp
## units    0.9693   0.8876    1.047     4500
##
##  Location effects: MeanCorner_hourly_scaled ~ PredatorCue
##
##             post.mean  l-95% CI  u-95% CI eff.samp  pMCMC
## (Intercept)  0.223495 -0.009375  0.484027     4500 0.0782 .
## PredatorCue  0.031077 -0.282889  0.329498     4077 0.8489
## ---
## Signif. codes:  0 '***' 0.001 '**' 0.01 '*' 0.05 '.' 0.1 ' ' 1
```

Calculate probability of direction (pd) for treatment (PredatorCue):

```r
post_samples <- as.data.frame(day1_model$Sol)
post_diff <- post_samples[, "PredatorCue"]
pd <- mean(post_diff < 0)
pd  # 0.424 (very low chance of difference)
```

```
## [1] 0.4244444
```

```r
ppvalue_comparison <- pd_to_p(pd = pd)
```

```
## Warning: pd-values smaller than 0.5 detected, indicating inconsistent direction
##   of the probability mass. This usually happens when the parameters space
##   is not continuous. Affected values are set to 1. See help('p_direction')
##   for more info.
```

```r
ppvalue_comparison  # Too insignificant to register
```

```
## [1] 1
```

Calculate ICCs for variance components:

```r
FishId_intercept <- model$VCV[, "(Intercept):(Intercept).FishId"]
FishId_slope <- model$VCV[, "ExpDay:ExpDay.FishId"]
FishId_cov <- model$VCV[, "(Intercept):ExpDay.FishId"]

MomId_intercept <- model$VCV[, "MomId"]

days <- 0:27
mean_days <- mean(days)
mean_days_sq <- mean(days^2)

res_columns <- paste0("ExpDay_factor", days, ".units")
residual_var <- rowMeans(model$VCV[, res_columns, drop = FALSE])

FishId_var <- FishId_intercept + 2 * FishId_cov * mean_days +
    FishId_slope * mean_days_sq
MomId_var <- MomId_intercept

# ICC calculations
ICC_FishId <- FishId_var/(FishId_var + residual_var)
ICC_FishId_output <- c(median = median(ICC_FishId), HPDinterval(as.mcmc(ICC_FishId))[1,
    ])
ICC_FishId_withmom <- FishId_var/(FishId_var + MomId_var + residual_var)
ICC_FishId_withmom_output <- c(median = median(ICC_FishId_withmom),
    HPDinterval(as.mcmc(ICC_FishId_withmom))[1, ])
ICC_MomId <- MomId_var/(FishId_var + MomId_var + residual_var)
ICC_MomId_output <- c(median = median(ICC_MomId), HPDinterval(as.mcmc(ICC_MomId))[1,
    ])
ICC_residual <- residual_var/(FishId_var + MomId_var + residual_var)
ICC_residual_output <- c(median = median(ICC_residual), HPDinterval(as.mcmc(ICC_residual))[1,
    ])

ICC_FishId_output  # 0.274 [0.223, 0.326]
```

```
##    median     lower     upper
## 0.2739652 0.2228093 0.3255404
```

```r
ICC_FishId_withmom_output  # 0.267 [0.214, 0.323]
```

```
##    median     lower     upper
## 0.2665554 0.2143400 0.3231944
```

```r
ICC_MomId_output  # 0.021 [<0.0001, 0.081]
```

```
##       median        lower        upper
## 2.053222e-02 8.543751e-11 8.117267e-02
```

```r
ICC_residual_output  # 0.707 [0.644, 0.762]
```

```
##    median     lower     upper
```

```
## 0.7074183 0.6440542 0.7623383
```

```r
# Bar plot comparing ICCs

ICC_data_for_plot <- data.frame(Component = c("Among individuals",
    "Maternal identity", "Within individuals"), ICCs = c(0.267,
    0.021, 0.707), Lower = c(0.214, 1e-04, 0.644), Upper = c(0.323,
    0.081, 0.726))

# Plot
plot_name <- ggplot(ICC_data_for_plot, aes(x = Component, y = ICCs)) +
    geom_bar(stat = "identity", fill = "lightgrey", width = 0.6) +
    geom_errorbar(aes(ymin = Lower, ymax = Upper), width = 0.2,
        size = 0.7) + scale_y_continuous(limits = c(0, 0.9),
    breaks = seq(0, 0.9, by = 0.1), expand = c(0, 0)) + theme_classic()
plot_name
```

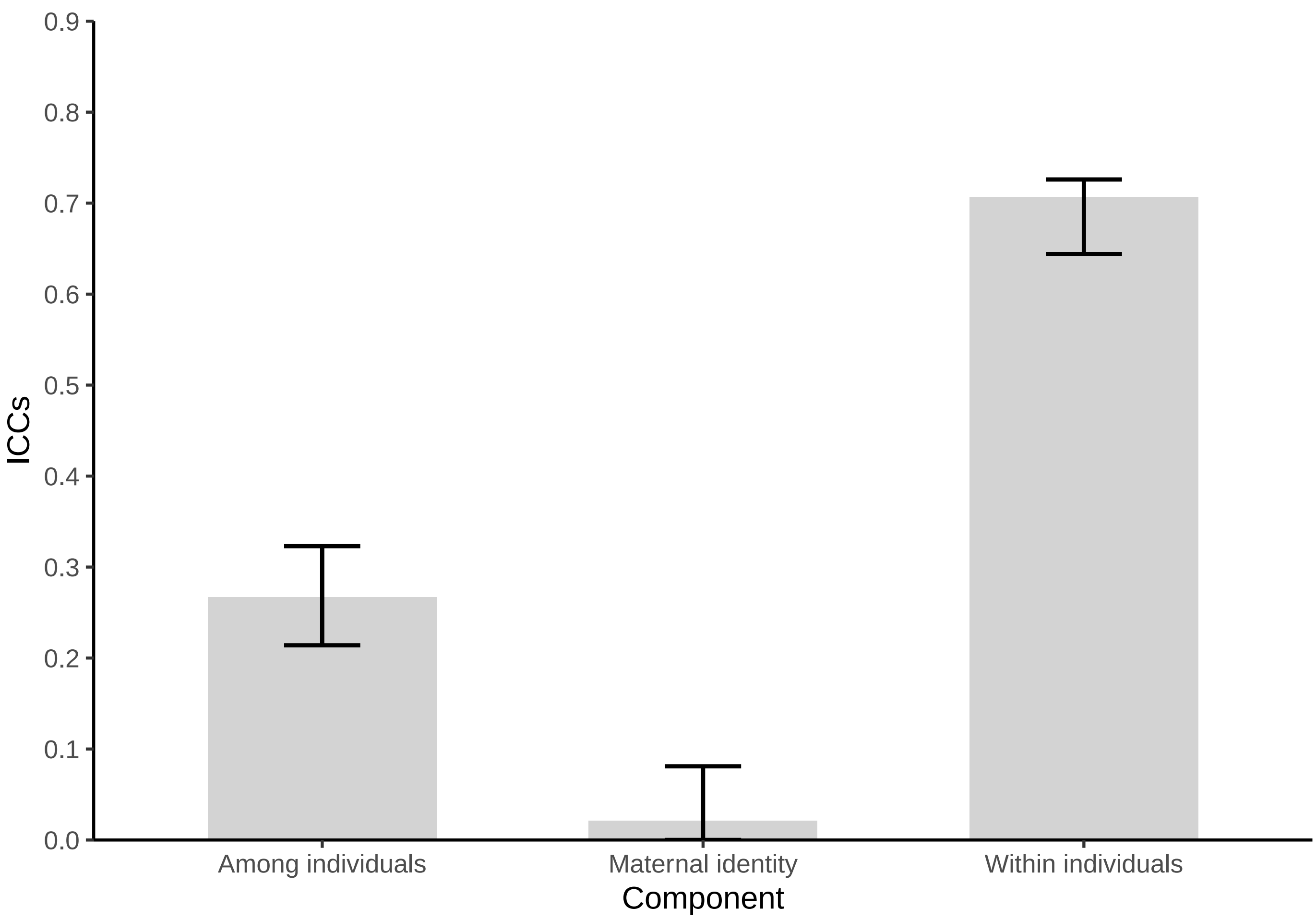


```r
ggsave("refuge_use_ICCs_plot.pdf", plot = plot_name, dpi = 600,
    width = 6, height = 6)
```

Investigating maternal effects

We found that MomId does not contribute significantly to variance.

Linking mom and offspring behavior

We will first investigate whether mother behavior predicts offspring behavior. Does it matter what mom does, or just who she is? Like our investigation of body size earlier, we look at this using a subset of our data because not all babies

have mom behavior data.
First, we will prepare model comparison only including individuals with mom behavior data.

Mom behavior model comparison

Model with mom behavior:

```
# Make day into factor for data subset
mombehavior_only_data_hourly$ExpDay_factor <- as.factor(mombehavior_only_data_hourly$ExpDay)

model_MeanCorner_mom_behavior <- readRDS("model_MeanCorner_mom_behavior_10_16_25.rds")
# model_MeanCorner_mom_behavior <-
# MCMCglmm(MeanCorner_hourly_scaled ~ PredatorCue*ExpDay +
# Hour + Avg_MeanCorner_mom, random = ~us(1 +
# ExpDay):FishId + MomId, rcov = ~idh(ExpDay_factor):units,
# family = 'gaussian', data = mombehavior_only_data_hourly,
# prior =
# prior.univariate.FishId_slopes.MomId.heteroResidual, nitt
# = 500000, burnin = 50000, thin = 100)
# saveRDS(model_MeanCorner_mom_behavior, file =
# 'model_MeanCorner_mom_behavior_10_16_25.rds')
summary(model_MeanCorner_mom_behavior)
```

```
##
##  Iterations = 50001:499901
##  Thinning interval  = 100
##  Sample size  = 4500
##
##  DIC: 61760.14
##
##  G-structure:  ~us(1 + ExpDay):FishId
##
##                                post.mean   l-95% CI   u-95% CI eff.samp
## (Intercept):(Intercept).FishId  0.296149  0.2001394  0.3959581     4500
## ExpDay:(Intercept).FishId      -0.005663 -0.0093846 -0.0022977     4500
## (Intercept):ExpDay.FishId      -0.005663 -0.0093846 -0.0022977     4500
## ExpDay:ExpDay.FishId            0.000617  0.0004284  0.0008365     4500
##
##                ~MomId
##
##       post.mean l-95% CI u-95% CI eff.samp
## MomId   0.03916 2.21e-09   0.1344     1389
##
##  R-structure:  ~idh(ExpDay_factor):units
##
##                       post.mean l-95% CI u-95% CI eff.samp
## ExpDay_factor0.units     1.4180   1.2893   1.5421     4500
## ExpDay_factor1.units     0.9982   0.9140   1.0926     4500
## ExpDay_factor2.units     1.0750   0.9816   1.1782     4500
## ExpDay_factor3.units     1.0467   0.9541   1.1419     4500
## ExpDay_factor4.units     0.9551   0.8719   1.0424     4500
## ExpDay_factor5.units     0.5581   0.5093   0.6103     4500
## ExpDay_factor6.units     0.5730   0.5236   0.6267     4500
## ExpDay_factor7.units     0.6815   0.6251   0.7479     4300
## ExpDay_factor8.units     0.4809   0.4373   0.5227     4500
## ExpDay_factor9.units     0.6578   0.5978   0.7170     4500
## ExpDay_factor10.units    0.5873   0.5363   0.6450     4500
## ExpDay_factor11.units    0.5193   0.4688   0.5644     4500
## ExpDay_factor12.units    0.6423   0.5885   0.7023     4500
## ExpDay_factor13.units    0.5762   0.5269   0.6306     4310
## ExpDay_factor14.units    0.4884   0.4428   0.5306     4266
## ExpDay_factor15.units    0.6310   0.5762   0.6877     4500
```

```
## ExpDay_factor16.units    0.7106   0.6449   0.7758     4500
## ExpDay_factor17.units    0.5227   0.4744   0.5700     4500
## ExpDay_factor18.units    0.5643   0.5142   0.6172     4500
## ExpDay_factor19.units    0.5248   0.4784   0.5708     5678
## ExpDay_factor20.units    0.5888   0.5385   0.6455     4500
## ExpDay_factor21.units    0.6340   0.5757   0.6901     4500
## ExpDay_factor22.units    0.4736   0.4336   0.5201     4790
## ExpDay_factor23.units    0.4224   0.3838   0.4597     4500
## ExpDay_factor24.units    0.4998   0.4551   0.5442     4268
## ExpDay_factor25.units    0.4855   0.4405   0.5297     4500
## ExpDay_factor26.units    0.5673   0.5159   0.6213     4926
## ExpDay_factor27.units    0.5101   0.4637   0.5604     4223
##
##  Location effects: MeanCorner_hourly_scaled ~ PredatorCue * ExpDay + Hour + Avg_MeanCorner_mom
##
##                    post.mean  l-95% CI  u-95% CI eff.samp  pMCMC
## (Intercept)        -0.011901 -0.754445  0.717128     4500  0.977
## PredatorCue         0.191260 -0.040546  0.421353     4500  0.117
## ExpDay             -0.004929 -0.013181  0.002658     4500  0.229
## Hour               -0.015097 -0.017840 -0.012190     4500 <2e-04 ***
## Avg_MeanCorner_mom  0.023477 -1.282170  1.405095     4500  0.989
## PredatorCue:ExpDay  0.006249 -0.005223  0.017274     4500  0.280
## ---
## Signif. codes:  0 '***' 0.001 '**' 0.01 '*' 0.05 '.' 0.1 ' ' 1
```

Mother behavior nor mother ID predicts offspring behavior.

Plot mother by offspring behavior by treatment

```r
# Create columns for control and predator treatments
mombehavior_only_data_hourly$MeanCorner_control <- ifelse(mombehavior_only_data_hourly$PredatorCue ==
    0, mombehavior_only_data_hourly$MeanCorner_, NA)
mombehavior_only_data_hourly$MeanCorner_predator <- ifelse(mombehavior_only_data_hourly$PredatorCue ==
    1, mombehavior_only_data_hourly$MeanCorner_, NA)

# Sum per fish
mean_per_fish <- mombehavior_only_data_hourly %>%
    mutate(OffspringCorner = ifelse(PredatorCue == 0, MeanCorner_control,
        MeanCorner_predator)) %>%
    group_by(FishId, MomId, Avg_MeanCorner_mom, PredatorCue) %>%
    summarize(OffspringCorner = mean(OffspringCorner, na.rm = TRUE),
        .groups = "drop")
# Hourly data
raw_hourly <- mombehavior_only_data_hourly %>%
    mutate(OffspringCorner = ifelse(PredatorCue == 0, MeanCorner_control,
        MeanCorner_predator))
# Set colors
colors <- c(`0` = "#1709b5", `1` = "#ed5715")
larger_fill_colors <- c(`0` = "#8e9ee9", `1` = "#f79a6f")

# Plot with only individual fish means
plot_name <- ggplot() + geom_smooth(data = mean_per_fish, aes(x = Avg_MeanCorner_mom,
    y = OffspringCorner), method = "lm", se = TRUE, color = "grey60",
    fill = "grey70", linetype = "solid", alpha = 0.3, linewidth = 0.5) +
    geom_boxplot(data = mean_per_fish, aes(x = Avg_MeanCorner_mom,
        y = OffspringCorner, group = Avg_MeanCorner_mom), fill = "white",
        color = "black", alpha = 1, width = 0.01, outlier.shape = NA) +
    geom_point(data = mean_per_fish, aes(x = Avg_MeanCorner_mom,
        y = OffspringCorner, color = factor(PredatorCue)), shape = 16,
        position = position_jitter(width = 0), size = 2, alpha = 1) +
    scale_color_manual(values = colors) + scale_fill_manual(values = colors) +
```

```
    scale_x_continuous(limits = c(0.29, 0.7), breaks = seq(0.3,
        max(0.7, na.rm = TRUE), by = 0.1)) + scale_y_continuous(limits = c(0.2,
    0.51), breaks = seq(0.2, max(0.5, na.rm = TRUE), by = 0.1)) +
    theme_classic() + theme(legend.position = "none", axis.line = element_line(linewidth = 0.5,
    colour = "black"), axis.ticks = element_line(colour = "black"),
    axis.text = element_text(size = 14, colour = "black"), axis.title = element_text(size = 18,
        colour = "black"), plot.title = element_text(size = 0)) +
    labs(x = "Maternal corner", y = "Offspring corner")
plot_name
```

```
## `geom_smooth()` using formula = 'y ~ x'

## Warning: Removed 1 row containing non-finite outside the scale range
## (`stat_smooth()`).

## Warning: Removed 1 row containing non-finite outside the scale range
## (`stat_boxplot()`).

## Warning: No shared levels found between `names(values)` of the manual scale and the
## data's fill values.

## Warning: Removed 1 row containing missing values or values outside the scale range
## (`geom_point()`).
```

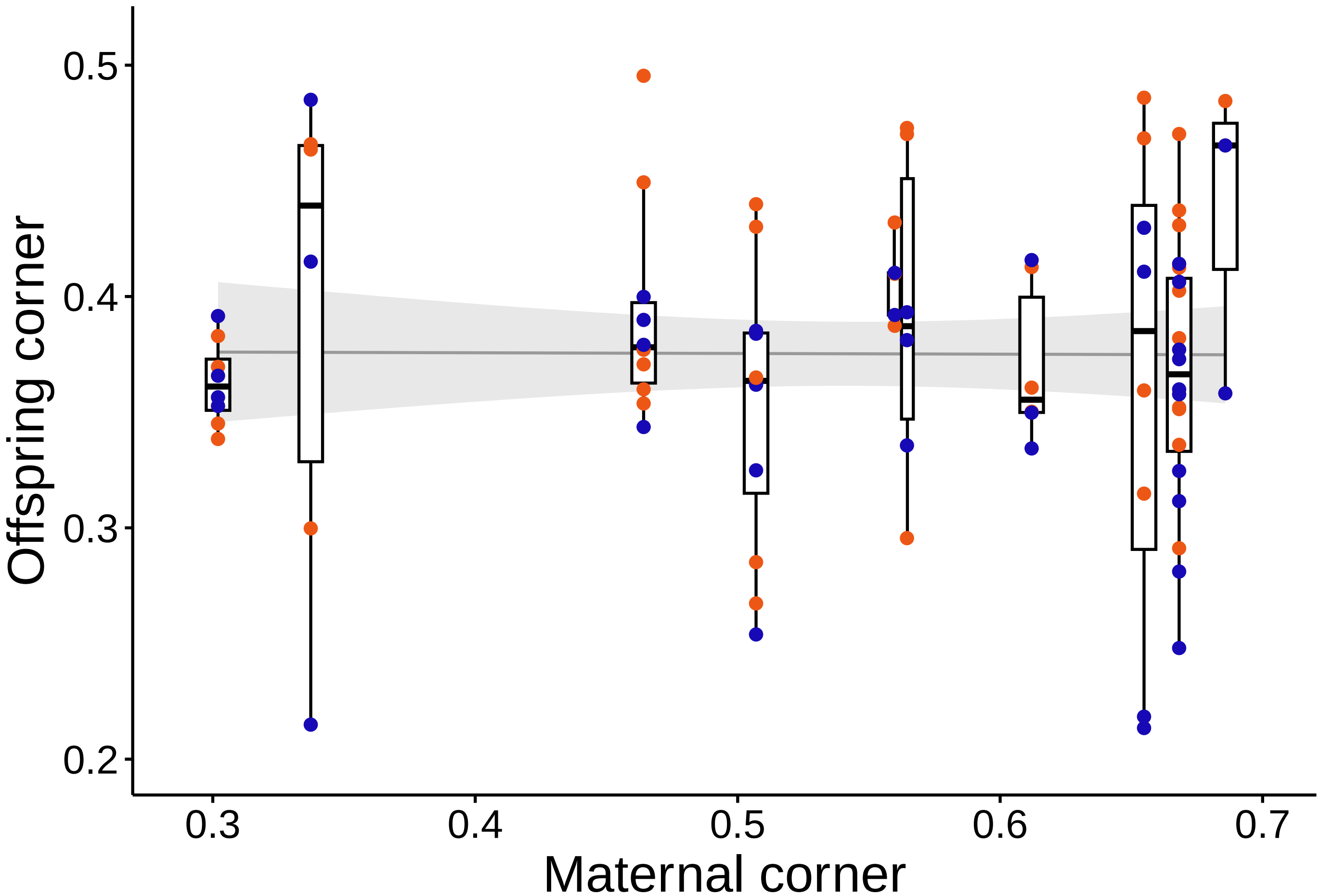


```
ggsave("maternal_behavior_refuge_use.pdf", plot = plot_name,
    dpi = 600, width = 6, height = 6)
```

```
## `geom_smooth()` using formula = 'y ~ x'

## Warning: Removed 1 row containing non-finite outside the scale range
## (`stat_smooth()`).
```

```
## Warning: Removed 1 row containing non-finite outside the scale range
## (`stat_boxplot()`).

## Warning: No shared levels found between `names(values)` of the manual scale and the
## data's fill values.

## Warning: Removed 1 row containing missing values or values outside the scale range
## (`geom_point()`).
```

Plotting MomId by treatment

Now we will make a plot showing control by predator behavior, grouped by mother ID. This uses the full data set because we don't need mother behavior.
Each point on the plot will represent the mean values of all individuals who share a mother. Error bars will show the standard error of means for control (horizontal, blue) and predator (vertical, orange) offspring. The error bars are built from FishId means (not raw data) for each MomId.

```
# Mean for each FishId
fish_means <- hourly_data %>%
    group_by(MomId, FishId, PredatorCue) %>%
    summarize(mean_MeanCorner = mean(MeanCorner_hourly_scaled,
        na.rm = TRUE), .groups = "drop")

# Mean and SE for MomId and treatment using FishId means
momid_by_treatment_data <- fish_means %>%
    group_by(MomId, PredatorCue) %>%
    summarize(mom_mean_MeanCorner = mean(mean_MeanCorner, na.rm = TRUE),
        se_MeanCorner = sd(mean_MeanCorner, na.rm = TRUE)/sqrt(n()),
        n_fish = n(), .groups = "drop")

# Organize into cols
momid_by_treatment_data <- momid_by_treatment_data %>%
    pivot_wider(names_from = PredatorCue, values_from = c(mom_mean_MeanCorner,
        se_MeanCorner, n_fish), names_glue = "{.value}_{PredatorCue}") %>%
    rename(mean_control = mom_mean_MeanCorner_0, se_control = se_MeanCorner_0,
        n_fish_control = n_fish_0, mean_predator = mom_mean_MeanCorner_1,
        se_predator = se_MeanCorner_1, n_fish_predator = n_fish_1)

# Plot
colors <- c(`0` = "#1709b5", `1` = "#ed5715")
plot_name <- ggplot(momid_by_treatment_data, aes(x = mean_control,
    y = mean_predator)) + geom_errorbarh(aes(xmin = mean_control -
    se_control, xmax = mean_control + se_control), height = 0.05,
    size = 0.7, color = colors["0"], alpha = 1) + geom_errorbar(aes(ymin = mean_predator -
    se_predator, ymax = mean_predator + se_predator), width = 0.05,
    size = 0.7, color = colors["1"], alpha = 1) + geom_point(size = 2.5,
    shape = 21, color = "black", fill = "grey35", stroke = 1,
    alpha = 1) + xlim(-1.1, 1.1) + ylim(-1.1, 1.1) + coord_fixed(ratio = 1) +
    labs(x = "Control", y = "Predator") + theme_classic()
plot_name
```

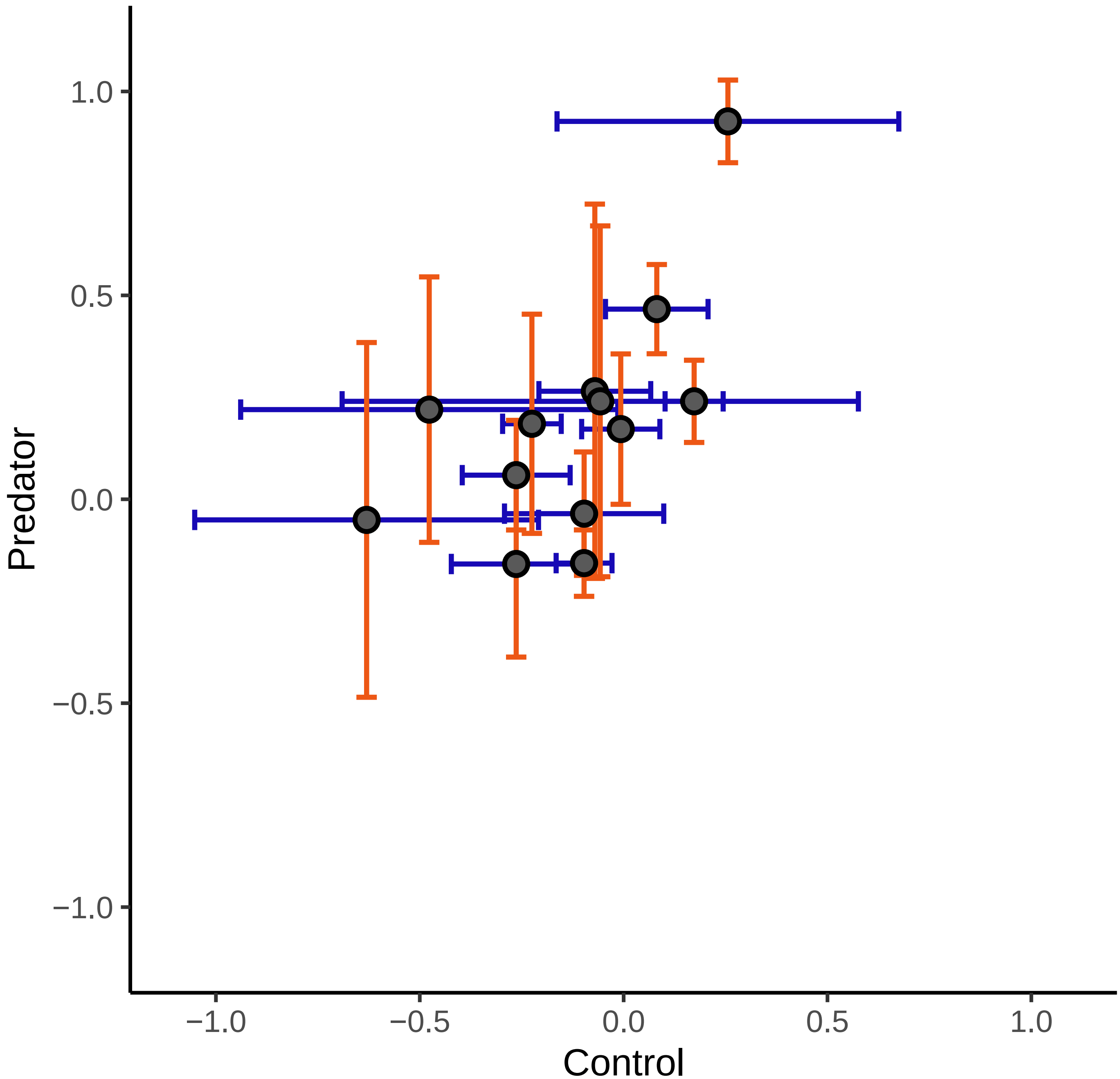


```
ggsave("maternal_effects_refuge_use.pdf", plot = plot_name, dpi = 600)
```

```
## Saving 6.5 x 4.5 in image
```

Testing for differences in individual variance between treatments

Now we will look at whether individual variance components differ by treatment using separate univariate models.

Prepare separate univariate control and predator models

Subset data by treatment for hourly:

```
control_data_hourly <- subset(hourly_data, PredatorCue == 0)
predator_data_hourly <- subset(hourly_data, PredatorCue == 1)
```

Set expanded priors for univariate models

```
# Prepare ExpDay to be a factor for heterogeneous residual
# variance
control_data_hourly$ExpDay_factor <- as.factor(control_data_hourly$ExpDay)
predator_data_hourly$ExpDay_factor <- as.factor(predator_data_hourly$ExpDay)

num_days <- nlevels(control_data_hourly$ExpDay_factor)
R_prior <- list(V = diag(num_days) * 0.5, nu = num_days + 0.002)

# Null
prior.null.univariate.idhExpDay <- list(R = R_prior)

# FishId
prior.fishId.univariate.idhExpDay <- list(R = R_prior, G = list(G1 = list(V = 1,
    nu = 1.002, alpha.mu = 0, alpha.V = 25^2)))

# MomId
prior.momId.univariate.idhExpDay <- list(R = R_prior, G = list(G1 = list(V = 1,
    nu = 1.002, alpha.mu = 0, alpha.V = 25^2)))

# FishId + MomId
prior.fishId.momId.univariate.idhExpDay <- list(R = R_prior,
    G = list(G1 = list(V = 1, nu = 1.002, alpha.mu = 0, alpha.V = 25^2),
        G2 = list(V = 1, nu = 1.002, alpha.mu = 0, alpha.V = 25^2)))

# FishId slopes
prior.fishId.slopes.univariate.idhExpDay <- list(R = R_prior,
    G = list(G1 = list(V = diag(2) * 0.5, nu = 2.002, alpha.mu = c(0,
        0), alpha.V = diag(2) * 25^2)))

# FishId slopes + MomId
prior.fishId.slopes.momId.univariate.idhExpDay <- list(R = R_prior,
    G = list(G1 = list(V = diag(2) * 0.5, nu = 2.002, alpha.mu = c(0,
        0), alpha.V = diag(2) * 25^2), G2 = list(V = 1, nu = 1.002,
        alpha.mu = 0, alpha.V = 25^2)))

# FishId slopes + MomId slopes
prior.fishId.slopes.momId.slopes.univariate.idhExpDay <- list(R = R_prior,
    G = list(G1 = list(V = diag(2) * 0.5, nu = 2.002, alpha.mu = c(0,
        0), alpha.V = diag(2) * 25^2), G2 = list(V = diag(2) *
        0.5, nu = 2.002, alpha.mu = c(0, 0), alpha.V = diag(2) *
        25^2)))

# FishId slopes + MomId - homogeneous residual variance
# (for confirmation)
prior.fishId.slopes.momId.univariate.homogemeous_residual <- list(R = list(V = 1,
    nu = 0.002), G = list(G1 = list(V = diag(2) * 0.5, nu = 2.002,
    alpha.mu = c(0, 0), alpha.V = diag(2) * 25^2), G2 = list(V = 1,
    nu = 1.002, alpha.mu = 0, alpha.V = 25^2)))
```

Model comparison to determine included random effects for CONTROL

```
# Null
model_control_hetero_residual_null <- readRDS("model_MeanCorner_control_hetero_residual_null_8_22_25.rds")
# model_control_hetero_residual_null <-
# MCMCglmm(MeanCorner_hourly_scaled ~ ExpDay + YearBatch +
# Hour, rcov = ~idh(ExpDay_factor):units, family =
# 'gaussian', data = control_data_hourly, prior =
# prior.null.univariate.idhExpDay, nitt = 500000, burnin =
# 50000, thin = 100)
```

```r
# saveRDS(model_control_hetero_residual_null, file =
# 'model_MeanCorner_control_hetero_residual_null_8_22_25.rds')
summary(model_control_hetero_residual_null)
```

```
##
##  Iterations = 50001:499901
##  Thinning interval  = 100
##  Sample size  = 4500
##
##  DIC: 40979.85
##
##  R-structure:  ~idh(ExpDay_factor):units
##
##                        post.mean l-95% CI u-95% CI eff.samp
## ExpDay_factor0.units      1.3070   1.1606   1.4597     4500
## ExpDay_factor1.units      0.9421   0.8412   1.0480     4500
## ExpDay_factor2.units      1.0992   0.9746   1.2231     4277
## ExpDay_factor3.units      1.0858   0.9532   1.2072     4743
## ExpDay_factor4.units      1.0129   0.9038   1.1329     3504
## ExpDay_factor5.units      0.6367   0.5626   0.7071     4500
## ExpDay_factor6.units      0.6094   0.5382   0.6800     4500
## ExpDay_factor7.units      0.7708   0.6870   0.8618     4819
## ExpDay_factor8.units      0.6033   0.5370   0.6715     4500
## ExpDay_factor9.units      0.7527   0.6673   0.8349     4728
## ExpDay_factor10.units     0.9132   0.7981   1.0228     4500
## ExpDay_factor11.units     0.7000   0.6221   0.7848     4500
## ExpDay_factor12.units     0.7628   0.6758   0.8555     3913
## ExpDay_factor13.units     0.7303   0.6469   0.8107     4500
## ExpDay_factor14.units     0.7567   0.6774   0.8425     5387
## ExpDay_factor15.units     0.8898   0.7879   0.9931     4500
## ExpDay_factor16.units     0.8041   0.7161   0.8987     4500
## ExpDay_factor17.units     0.7857   0.6967   0.8778     4500
## ExpDay_factor18.units     0.7992   0.7048   0.8925     4715
## ExpDay_factor19.units     0.8312   0.7407   0.9298     4741
## ExpDay_factor20.units     0.8983   0.7950   1.0024     4500
## ExpDay_factor21.units     0.8437   0.7460   0.9408     4296
## ExpDay_factor22.units     0.7621   0.6815   0.8547     4500
## ExpDay_factor23.units     0.6740   0.5996   0.7549     4500
## ExpDay_factor24.units     0.7726   0.6900   0.8657     4500
## ExpDay_factor25.units     0.6727   0.5926   0.7512     4567
## ExpDay_factor26.units     0.7629   0.6713   0.8527     4312
## ExpDay_factor27.units     0.7440   0.6560   0.8334     4500
##
##  Location effects: MeanCorner_hourly_scaled ~ ExpDay + YearBatch + Hour
##
##             post.mean  l-95% CI  u-95% CI eff.samp  pMCMC
## (Intercept)  0.154983  0.107350  0.203378     4735 <2e-04 ***
## ExpDay      -0.007126 -0.008920 -0.005258     4500 <2e-04 ***
## YearBatch   -0.065015 -0.078120 -0.052098     4941 <2e-04 ***
## Hour        -0.015285 -0.019339 -0.011182     4859 <2e-04 ***
## ---
## Signif. codes:  0 '***' 0.001 '**' 0.01 '*' 0.05 '.' 0.1 ' ' 1
```

```r
# FishId
model_control_hetero_residual_id <- readRDS("model_MeanCorner_control_hetero_residual_id_8_22_25.rds")
# model_control_hetero_residual_id <-
# MCMCglmm(MeanCorner_hourly_scaled ~ ExpDay + YearBatch +
# Hour, random = ~FishId, rcov = ~idh(ExpDay_factor):units,
# family = 'gaussian', data = control_data_hourly, prior =
# prior.fishId.univariate.idhExpDay, nitt = 500000, burnin
# = 50000, thin = 100)
```

```r
# saveRDS(model_control_hetero_residual_id, file =
# 'model_MeanCorner_control_hetero_residual_id_8_22_25.rds')
summary(model_control_hetero_residual_id)
```

```
##
##  Iterations = 50001:499901
##  Thinning interval  = 100
##  Sample size  = 4500
##
##  DIC: 35880.7
##
##  G-structure:  ~FishId
##
##        post.mean l-95% CI u-95% CI eff.samp
## FishId    0.2338   0.1492   0.3347     2713
##
##  R-structure:  ~idh(ExpDay_factor):units
##
##                       post.mean l-95% CI u-95% CI eff.samp
## ExpDay_factor0.units     1.2743   1.1320   1.4181     4500
## ExpDay_factor1.units     0.8007   0.7115   0.8945     4500
## ExpDay_factor2.units     0.9076   0.8064   1.0102     4500
## ExpDay_factor3.units     0.8825   0.7863   0.9884     4500
## ExpDay_factor4.units     0.8250   0.7270   0.9130     4500
## ExpDay_factor5.units     0.4882   0.4346   0.5479     4500
## ExpDay_factor6.units     0.4549   0.4000   0.5077     4372
## ExpDay_factor7.units     0.6323   0.5638   0.7055     4102
## ExpDay_factor8.units     0.4453   0.3974   0.4994     4073
## ExpDay_factor9.units     0.5887   0.5235   0.6563     4500
## ExpDay_factor10.units    0.6844   0.6001   0.7675     4500
## ExpDay_factor11.units    0.5039   0.4470   0.5650     4500
## ExpDay_factor12.units    0.4930   0.4372   0.5517     4192
## ExpDay_factor13.units    0.4882   0.4344   0.5431     4500
## ExpDay_factor14.units    0.4785   0.4261   0.5359     4500
## ExpDay_factor15.units    0.6259   0.5578   0.6959     4500
## ExpDay_factor16.units    0.5724   0.5092   0.6373     4500
## ExpDay_factor17.units    0.5315   0.4767   0.5946     4764
## ExpDay_factor18.units    0.5135   0.4538   0.5755     4500
## ExpDay_factor19.units    0.5514   0.4860   0.6109     4355
## ExpDay_factor20.units    0.6175   0.5469   0.6896     4500
## ExpDay_factor21.units    0.6165   0.5485   0.6880     4500
## ExpDay_factor22.units    0.5508   0.4875   0.6136     4500
## ExpDay_factor23.units    0.4525   0.4003   0.5068     4695
## ExpDay_factor24.units    0.5294   0.4681   0.5895     4500
## ExpDay_factor25.units    0.3931   0.3476   0.4386     4849
## ExpDay_factor26.units    0.5472   0.4861   0.6115     4500
## ExpDay_factor27.units    0.5081   0.4468   0.5694     4500
##
##  Location effects: MeanCorner_hourly_scaled ~ ExpDay + YearBatch + Hour
##
##             post.mean  l-95% CI  u-95% CI eff.samp  pMCMC
## (Intercept)  0.225311 -0.085228  0.532871     4500 0.1467
## ExpDay      -0.005803 -0.007218 -0.004084     4697 <2e-04 ***
## YearBatch   -0.095630 -0.213468  0.015609     4500 0.0956 .
## Hour        -0.016410 -0.019820 -0.013057     4500 <2e-04 ***
## ---
## Signif. codes:  0 '***' 0.001 '**' 0.01 '*' 0.05 '.' 0.1 ' ' 1
```

```r
# MomId
model_control_hetero_residual_mom <- readRDS("model_MeanCorner_control_hetero_residual_mom_8_22_25.rds")
# model_control_hetero_residual_mom <-
```

```r
# MCMCglmm(MeanCorner_hourly_scaled ~ ExpDay + YearBatch +
# Hour, random = ~MomId, rcov = ~idh(ExpDay_factor):units,
# family = 'gaussian', data = control_data_hourly, prior =
# prior.momId.univariate.idhExpDay, nitt = 500000, burnin =
# 50000, thin = 100)
# saveRDS(model_control_hetero_residual_mom, file =
# 'model_MeanCorner_control_hetero_residual_mom_8_22_25.rds')
summary(model_control_hetero_residual_mom)
```

```
##
##  Iterations = 50001:499901
##  Thinning interval  = 100
##  Sample size  = 4500
##
##  DIC: 40234.72
##
##  G-structure:  ~MomId
##
##       post.mean l-95% CI u-95% CI eff.samp
## MomId   0.07703  0.02199   0.1592     2374
##
##  R-structure:  ~idh(ExpDay_factor):units
##
##                        post.mean l-95% CI u-95% CI eff.samp
## ExpDay_factor0.units     1.3412   1.1907   1.4967     4500
## ExpDay_factor1.units     0.9328   0.8317   1.0336     4500
## ExpDay_factor2.units     1.0394   0.9236   1.1564     4500
## ExpDay_factor3.units     1.0063   0.8880   1.1199     4500
## ExpDay_factor4.units     0.9512   0.8396   1.0567     4500
## ExpDay_factor5.units     0.5666   0.5015   0.6314     4500
## ExpDay_factor6.units     0.5786   0.5130   0.6498     4904
## ExpDay_factor7.units     0.7428   0.6576   0.8256     4307
## ExpDay_factor8.units     0.5704   0.5052   0.6367     4500
## ExpDay_factor9.units     0.7282   0.6486   0.8128     4500
## ExpDay_factor10.units    0.8845   0.7792   0.9934     4500
## ExpDay_factor11.units    0.6704   0.5928   0.7481     4500
## ExpDay_factor12.units    0.7105   0.6254   0.7919     5008
## ExpDay_factor13.units    0.7045   0.6269   0.7841     4556
## ExpDay_factor14.units    0.7043   0.6292   0.7853     4500
## ExpDay_factor15.units    0.8181   0.7279   0.9112     4500
## ExpDay_factor16.units    0.7649   0.6792   0.8539     4500
## ExpDay_factor17.units    0.7357   0.6474   0.8176     4500
## ExpDay_factor18.units    0.7604   0.6733   0.8503     4500
## ExpDay_factor19.units    0.7915   0.7014   0.8863     4500
## ExpDay_factor20.units    0.8451   0.7465   0.9424     4554
## ExpDay_factor21.units    0.8175   0.7304   0.9150     4500
## ExpDay_factor22.units    0.7431   0.6624   0.8334     4500
## ExpDay_factor23.units    0.6676   0.5883   0.7448     4291
## ExpDay_factor24.units    0.7332   0.6538   0.8213     4819
## ExpDay_factor25.units    0.6306   0.5549   0.7052     3830
## ExpDay_factor26.units    0.7453   0.6595   0.8322     4266
## ExpDay_factor27.units    0.7271   0.6456   0.8146     4500
##
##  Location effects: MeanCorner_hourly_scaled ~ ExpDay + YearBatch + Hour
##
##             post.mean  l-95% CI  u-95% CI eff.samp  pMCMC
## (Intercept)  0.284089 -0.027873  0.606421     4032 0.0676 .
## ExpDay      -0.006776 -0.008541 -0.004971     4500 <2e-04 ***
## YearBatch   -0.120209 -0.245694 -0.001674     5110 0.0431 *
## Hour        -0.015300 -0.019320 -0.011396     4500 <2e-04 ***
## ---
```

```
## Signif. codes:  0 '***' 0.001 '**' 0.01 '*' 0.05 '.' 0.1 ' ' 1
```

```
# FishId + MomId
model_control_hetero_residual_id_mom <- readRDS("model_MeanCorner_control_hetero_residual_id_mom_8_22_25.rds
# model_control_hetero_residual_id_mom <-
# MCMCglmm(MeanCorner_hourly_scaled ~ ExpDay + YearBatch +
# Hour, random = ~FishId + MomId, rcov =
# ~idh(ExpDay_factor):units, family = 'gaussian', data =
# control_data_hourly, prior =
# prior.fishId.momId.univariate.idhExpDay, nitt = 500000,
# burnin = 50000, thin = 100)
# saveRDS(model_control_hetero_residual_id_mom, file =
# 'model_MeanCorner_control_hetero_residual_id_mom_8_22_25.rds')
summary(model_control_hetero_residual_id_mom)
```

```
##
##  Iterations = 50001:499901
##  Thinning interval  = 100
##  Sample size  = 4500
##
##  DIC: 35880.5
##
##  G-structure:  ~FishId
##
##        post.mean l-95% CI u-95% CI eff.samp
## FishId    0.2337   0.1419    0.333     3078
##
##               ~MomId
##
##       post.mean  l-95% CI u-95% CI eff.samp
## MomId   0.02163 2.516e-09  0.08309     1466
##
##  R-structure:  ~idh(ExpDay_factor):units
##
##                        post.mean l-95% CI u-95% CI eff.samp
## ExpDay_factor0.units     1.2715   1.1295   1.4183     4713
## ExpDay_factor1.units     0.7992   0.7116   0.8919     4500
## ExpDay_factor2.units     0.9075   0.8150   1.0190     4500
## ExpDay_factor3.units     0.8794   0.7749   0.9824     4500
## ExpDay_factor4.units     0.8251   0.7345   0.9189     4500
## ExpDay_factor5.units     0.4872   0.4300   0.5425     4500
## ExpDay_factor6.units     0.4544   0.4011   0.5081     4294
## ExpDay_factor7.units     0.6331   0.5632   0.7060     4734
## ExpDay_factor8.units     0.4460   0.3966   0.4967     4500
## ExpDay_factor9.units     0.5879   0.5224   0.6547     4500
## ExpDay_factor10.units    0.6841   0.5998   0.7697     4500
## ExpDay_factor11.units    0.5043   0.4455   0.5644     4500
## ExpDay_factor12.units    0.4930   0.4380   0.5496     4500
## ExpDay_factor13.units    0.4883   0.4345   0.5438     4500
## ExpDay_factor14.units    0.4788   0.4273   0.5340     4500
## ExpDay_factor15.units    0.6257   0.5538   0.6953     4500
## ExpDay_factor16.units    0.5718   0.5078   0.6361     4500
## ExpDay_factor17.units    0.5318   0.4751   0.5952     4228
## ExpDay_factor18.units    0.5135   0.4572   0.5748     4500
## ExpDay_factor19.units    0.5512   0.4909   0.6186     4500
## ExpDay_factor20.units    0.6177   0.5484   0.6925     4138
## ExpDay_factor21.units    0.6162   0.5492   0.6893     4500
## ExpDay_factor22.units    0.5512   0.4874   0.6135     4275
## ExpDay_factor23.units    0.4521   0.3985   0.5047     4176
## ExpDay_factor24.units    0.5294   0.4692   0.5899     4500
## ExpDay_factor25.units    0.3936   0.3457   0.4412     4500
## ExpDay_factor26.units    0.5488   0.4842   0.6131     4497
```

```
## ExpDay_factor27.units    0.5088   0.4503   0.5713     4500
##
##  Location effects: MeanCorner_hourly_scaled ~ ExpDay + YearBatch + Hour
##
##             post.mean  l-95% CI  u-95% CI eff.samp  pMCMC
## (Intercept)  0.258089 -0.092328  0.624014     3562 0.1364
## ExpDay      -0.005841 -0.007437 -0.004278     4304 <2e-04 ***
## YearBatch   -0.109473 -0.244743  0.031456     3568 0.0916 .
## Hour        -0.016431 -0.019860 -0.013085     4500 <2e-04 ***
## ---
## Signif. codes:  0 '***' 0.001 '**' 0.01 '*' 0.05 '.' 0.1 ' ' 1
```

```
# FishId slopes
model_control_hetero_residual_id_slopes <- readRDS("model_MeanCorner_control_hetero_residual_id_slopes_8_22_
# model_control_hetero_residual_id_slopes <-
# MCMCglmm(MeanCorner_hourly_scaled ~ ExpDay + YearBatch +
# Hour, random = ~us(1 + ExpDay):FishId, rcov =
# ~idh(ExpDay_factor):units, family = 'gaussian', data =
# control_data_hourly, prior =
# prior.fishId.slopes.univariate.idhExpDay, nitt = 500000,
# burnin = 50000, thin = 100)
# saveRDS(model_control_hetero_residual_id_slopes, file =
# 'model_MeanCorner_control_hetero_residual_id_slopes_8_22_25.rds')
summary(model_control_hetero_residual_id_slopes)
```

```
##
##  Iterations = 50001:499901
##  Thinning interval  = 100
##  Sample size  = 4500
##
##  DIC: 35107.35
##
##  G-structure:  ~us(1 + ExpDay):FishId
##
##                              post.mean   l-95% CI   u-95% CI eff.samp
## (Intercept):(Intercept).FishId  0.282558  0.1749122  0.3970844     2730
## ExpDay:(Intercept).FishId      -0.005013 -0.0089601 -0.0013130     4500
## (Intercept):ExpDay.FishId      -0.005013 -0.0089601 -0.0013130     4500
## ExpDay:ExpDay.FishId            0.000541  0.0003412  0.0008018     3817
##
##  R-structure:  ~idh(ExpDay_factor):units
##
##                        post.mean l-95% CI u-95% CI eff.samp
## ExpDay_factor0.units      1.2343   1.0854   1.3719     4500
## ExpDay_factor1.units      0.7800   0.6971   0.8701     4500
## ExpDay_factor2.units      0.8627   0.7636   0.9622     4610
## ExpDay_factor3.units      0.8145   0.7232   0.9092     4500
## ExpDay_factor4.units      0.7449   0.6620   0.8317     4500
## ExpDay_factor5.units      0.4155   0.3700   0.4649     4500
## ExpDay_factor6.units      0.4101   0.3609   0.4584     4500
## ExpDay_factor7.units      0.6155   0.5467   0.6874     4500
## ExpDay_factor8.units      0.4320   0.3852   0.4819     4500
## ExpDay_factor9.units      0.5709   0.5041   0.6364     4500
## ExpDay_factor10.units     0.6714   0.5868   0.7545     4309
## ExpDay_factor11.units     0.4978   0.4403   0.5573     4285
## ExpDay_factor12.units     0.4880   0.4334   0.5486     4405
## ExpDay_factor13.units     0.4867   0.4322   0.5422     5139
## ExpDay_factor14.units     0.4778   0.4254   0.5348     4500
## ExpDay_factor15.units     0.6254   0.5542   0.6934     4220
## ExpDay_factor16.units     0.5728   0.5054   0.6372     4500
## ExpDay_factor17.units     0.5397   0.4823   0.6054     4500
## ExpDay_factor18.units     0.5083   0.4478   0.5676     4459
```

```
## ExpDay_factor19.units    0.5271   0.4670   0.5889     4500
## ExpDay_factor20.units    0.5950   0.5302   0.6676     4500
## ExpDay_factor21.units    0.5823   0.5186   0.6475     4500
## ExpDay_factor22.units    0.5133   0.4542   0.5711     4500
## ExpDay_factor23.units    0.4318   0.3790   0.4818     4500
## ExpDay_factor24.units    0.4907   0.4343   0.5479     4500
## ExpDay_factor25.units    0.3331   0.2948   0.3754     4500
## ExpDay_factor26.units    0.4696   0.4146   0.5257     4500
## ExpDay_factor27.units    0.4663   0.4092   0.5216     4262
##
##  Location effects: MeanCorner_hourly_scaled ~ ExpDay + YearBatch + Hour
##
##             post.mean  l-95% CI  u-95% CI eff.samp  pMCMC
## (Intercept)  0.203735 -0.090693  0.540618     4500 0.1929
## ExpDay      -0.005931 -0.012364  0.001033     4597 0.0809 .
## YearBatch   -0.087557 -0.202754  0.031464     5017 0.1342
## Hour        -0.016386 -0.019908 -0.013138     4451 <2e-04 ***
## ---
## Signif. codes:  0 '***' 0.001 '**' 0.01 '*' 0.05 '.' 0.1 ' ' 1
```

```r
# FishId slopes + MomId
model_control_hetero_residual_id_slopes_mom <- readRDS("model_MeanCorner_control_hetero_residual_id_slopes_m
# model_control_hetero_residual_id_slopes_mom <-
# MCMCglmm(MeanCorner_hourly_scaled ~ ExpDay + YearBatch +
# Hour, random = ~us(1 + ExpDay):FishId + MomId, rcov =
# ~idh(ExpDay_factor):units, family = 'gaussian', data =
# control_data_hourly, prior =
# prior.fishId.slopes.momId.univariate.idhExpDay, nitt =
# 500000, burnin = 50000, thin = 100)
# saveRDS(model_control_hetero_residual_id_slopes_mom, file
# =
# 'model_MeanCorner_control_hetero_residual_id_slopes_mom_include_hour_8_7_25.rds')
summary(model_control_hetero_residual_id_slopes_mom)  #
```

```
##
##  Iterations = 50001:499901
##  Thinning interval  = 100
##  Sample size  = 4500
##
##  DIC: 35107.38
##
##  G-structure:  ~us(1 + ExpDay):FishId
##
##                                 post.mean   l-95% CI   u-95% CI eff.samp
## (Intercept):(Intercept).FishId  0.2731676  0.1640697  0.3955653     2374
## ExpDay:(Intercept).FishId      -0.0047740 -0.0092527 -0.0012335     4500
## (Intercept):ExpDay.FishId      -0.0047740 -0.0092527 -0.0012335     4500
## ExpDay:ExpDay.FishId            0.0005456  0.0003207  0.0007991     3216
##
##                ~MomId
##
##       post.mean  l-95% CI u-95% CI eff.samp
## MomId   0.02695 6.983e-11  0.09999     1112
##
##  R-structure:  ~idh(ExpDay_factor):units
##
##                       post.mean l-95% CI u-95% CI eff.samp
## ExpDay_factor0.units     1.2341   1.0961   1.3771     4198
## ExpDay_factor1.units     0.7810   0.6953   0.8735     4500
## ExpDay_factor2.units     0.8606   0.7630   0.9608     4500
## ExpDay_factor3.units     0.8155   0.7200   0.9061     4500
## ExpDay_factor4.units     0.7439   0.6590   0.8272     4500
```

```
## ExpDay_factor5.units     0.4147   0.3643   0.4584     4500
## ExpDay_factor6.units     0.4099   0.3614   0.4601     4499
## ExpDay_factor7.units     0.6159   0.5480   0.6878     4500
## ExpDay_factor8.units     0.4322   0.3833   0.4837     4500
## ExpDay_factor9.units     0.5703   0.5040   0.6331     4884
## ExpDay_factor10.units    0.6704   0.5883   0.7573     4500
## ExpDay_factor11.units    0.4981   0.4402   0.5559     4576
## ExpDay_factor12.units    0.4881   0.4309   0.5459     4500
## ExpDay_factor13.units    0.4864   0.4327   0.5413     4500
## ExpDay_factor14.units    0.4787   0.4243   0.5327     4500
## ExpDay_factor15.units    0.6252   0.5552   0.6979     4500
## ExpDay_factor16.units    0.5724   0.5073   0.6378     4500
## ExpDay_factor17.units    0.5398   0.4789   0.6002     4707
## ExpDay_factor18.units    0.5070   0.4480   0.5653     4295
## ExpDay_factor19.units    0.5267   0.4671   0.5865     4500
## ExpDay_factor20.units    0.5952   0.5309   0.6707     5020
## ExpDay_factor21.units    0.5823   0.5149   0.6467     4500
## ExpDay_factor22.units    0.5143   0.4571   0.5739     4500
## ExpDay_factor23.units    0.4323   0.3818   0.4843     5262
## ExpDay_factor24.units    0.4914   0.4347   0.5488     4500
## ExpDay_factor25.units    0.3336   0.2952   0.3733     4500
## ExpDay_factor26.units    0.4700   0.4165   0.5267     4500
## ExpDay_factor27.units    0.4667   0.4119   0.5242     4683
##
##  Location effects: MeanCorner_hourly_scaled ~ ExpDay + YearBatch + Hour
##
##              post.mean   l-95% CI   u-95% CI eff.samp  pMCMC
## (Intercept)  0.2378581 -0.1237675  0.5949707     3943 0.1898
## ExpDay      -0.0060838 -0.0128967  0.0006817     4500 0.0738 .
## YearBatch   -0.0996290 -0.2366600  0.0312532     4500 0.1378
## Hour        -0.0164231 -0.0197410 -0.0130576     4591 <2e-04 ***
## ---
## Signif. codes:  0 '***' 0.001 '**' 0.01 '*' 0.05 '.' 0.1 ' ' 1
```

```r
# FishId slopes + MomId slopes
model_control_hetero_residual_id_slopes_mom_slopes <- readRDS("model_MeanCorner_control_hetero_residual_id_s
# model_control_hetero_residual_id_slopes_mom_slopes <-
# MCMCglmm(MeanCorner_hourly_scaled ~ ExpDay + YearBatch +
# Hour, random = ~us(1 + ExpDay):FishId + us(1 +
# ExpDay):MomId, rcov = ~idh(ExpDay_factor):units, family =
# 'gaussian', data = control_data_hourly, prior =
# prior.fishId.slopes.momId.slopes.univariate.idhExpDay,
# nitt = 500000, burnin = 50000, thin = 100)
# saveRDS(model_control_hetero_residual_id_slopes_mom_slopes,
# file =
# 'model_MeanCorner_control_hetero_residual_id_slopes_mom_slopes_8_22_25.rds')
summary(model_control_hetero_residual_id_slopes_mom_slopes)
```

```
##
##  Iterations = 50001:499901
##  Thinning interval  = 100
##  Sample size  = 4500
##
##  DIC: 35107.31
##
##  G-structure:  ~us(1 + ExpDay):FishId
##
##                                post.mean   l-95% CI   u-95% CI eff.samp
## (Intercept):(Intercept).FishId  0.2710210  0.1585403  0.3904710     2841
## ExpDay:(Intercept).FishId      -0.0045262 -0.0088035 -0.0008954     4238
## (Intercept):ExpDay.FishId      -0.0045262 -0.0088035 -0.0008954     4238
## ExpDay:ExpDay.FishId            0.0005293  0.0003022  0.0007713     3689
```

```
##
##                ~us(1 + ExpDay):MomId
##
##                                post.mean   l-95% CI  u-95% CI eff.samp
## (Intercept):(Intercept).MomId  3.171e-02  4.266e-11 0.1096500     1617
## ExpDay:(Intercept).MomId      -2.741e-04 -1.940e-03 0.0007483     4500
## (Intercept):ExpDay.MomId      -2.741e-04 -1.940e-03 0.0007483     4500
## ExpDay:ExpDay.MomId            4.479e-05  2.362e-12 0.0001689     2648
##
##  R-structure:  ~idh(ExpDay_factor):units
##
##                       post.mean l-95% CI u-95% CI eff.samp
## ExpDay_factor0.units     1.2331   1.0988   1.3798     4500
## ExpDay_factor1.units     0.7807   0.6899   0.8693     4432
## ExpDay_factor2.units     0.8618   0.7704   0.9674     4500
## ExpDay_factor3.units     0.8161   0.7250   0.9135     4500
## ExpDay_factor4.units     0.7431   0.6556   0.8223     4169
## ExpDay_factor5.units     0.4144   0.3671   0.4627     4500
## ExpDay_factor6.units     0.4097   0.3618   0.4596     4757
## ExpDay_factor7.units     0.6155   0.5471   0.6884     4500
## ExpDay_factor8.units     0.4319   0.3803   0.4798     4500
## ExpDay_factor9.units     0.5695   0.5065   0.6356     5221
## ExpDay_factor10.units    0.6708   0.5925   0.7575     4500
## ExpDay_factor11.units    0.4976   0.4432   0.5588     4500
## ExpDay_factor12.units    0.4875   0.4317   0.5461     4500
## ExpDay_factor13.units    0.4874   0.4326   0.5407     4500
## ExpDay_factor14.units    0.4793   0.4225   0.5311     4500
## ExpDay_factor15.units    0.6266   0.5551   0.6958     4500
## ExpDay_factor16.units    0.5731   0.5117   0.6403     4500
## ExpDay_factor17.units    0.5395   0.4768   0.6001     4215
## ExpDay_factor18.units    0.5071   0.4502   0.5696     4819
## ExpDay_factor19.units    0.5272   0.4630   0.5868     4500
## ExpDay_factor20.units    0.5954   0.5262   0.6629     4500
## ExpDay_factor21.units    0.5813   0.5157   0.6523     4500
## ExpDay_factor22.units    0.5139   0.4555   0.5730     4864
## ExpDay_factor23.units    0.4321   0.3839   0.4821     4500
## ExpDay_factor24.units    0.4910   0.4346   0.5482     4500
## ExpDay_factor25.units    0.3332   0.2949   0.3752     4964
## ExpDay_factor26.units    0.4695   0.4143   0.5252     4500
## ExpDay_factor27.units    0.4662   0.4136   0.5262     4635
##
##  Location effects: MeanCorner_hourly_scaled ~ ExpDay + YearBatch + Hour
##
##             post.mean  l-95% CI  u-95% CI eff.samp  pMCMC
## (Intercept)  0.242404 -0.091032  0.621737     4500  0.172
## ExpDay      -0.006098 -0.014223  0.001727     3518  0.120
## YearBatch   -0.101296 -0.234302  0.034689     4500  0.137
## Hour        -0.016365 -0.019839 -0.013124     4500 <2e-04 ***
## ---
## Signif. codes:  0 '***' 0.001 '**' 0.01 '*' 0.05 '.' 0.1 ' ' 1
```

```
# FishId slopes + MomId, but checking with homogeneous
# residual variance
model_control_homo_residual_id_slopes_mom <- readRDS("model_MeanCorner_control_homo_residual_id_slopes_mom_
# model_control_homo_residual_id_slopes_mom <-
# MCMCglmm(MeanCorner_hourly_scaled ~ ExpDay + YearBatch +
# Hour, random = ~us(1 + ExpDay):FishId + MomId, rcov =
# ~units, family = 'gaussian', data = control_data_hourly,
# prior =
# prior.fishId.slopes.momId.univariate.homogemeous_residual,
# nitt = 500000, burnin = 50000, thin = 100)
# saveRDS(model_control_homo_residual_id_slopes_mom, file =
```

```r
# 'model_MeanCorner_control_homo_residual_id_slopes_mom_8_22_25.rds')
summary(model_control_homo_residual_id_slopes_mom)  # Worse fit than heterogeneous residual variance
```

```
##
##  Iterations = 50001:499901
##  Thinning interval  = 100
##  Sample size  = 4500
##
##  DIC: 35652.55
##
##  G-structure:  ~us(1 + ExpDay):FishId
##
##                                 post.mean   l-95% CI   u-95% CI eff.samp
## (Intercept):(Intercept).FishId  0.2713218  0.1685448  0.3944391     2057
## ExpDay:(Intercept).FishId      -0.0045609 -0.0086406 -0.0010377     4500
## (Intercept):ExpDay.FishId      -0.0045609 -0.0086406 -0.0010377     4500
## ExpDay:ExpDay.FishId            0.0005349  0.0003128  0.0007738     3709
##
##                ~MomId
##
##       post.mean  l-95% CI u-95% CI eff.samp
## MomId   0.02629 4.365e-11  0.09808     1693
##
##  R-structure:  ~units
##
##       post.mean l-95% CI u-95% CI eff.samp
## units    0.5795   0.5668    0.593     4500
##
##  Location effects: MeanCorner_hourly_scaled ~ ExpDay + YearBatch + Hour
##
##             post.mean  l-95% CI  u-95% CI eff.samp  pMCMC
## (Intercept)  0.283731 -0.053699  0.646688     4500 0.1004
## ExpDay      -0.008405 -0.015153 -0.001485     4500 0.0178 *
## YearBatch   -0.108608 -0.241424  0.020153     3648 0.0871 .
## Hour        -0.013721 -0.016926 -0.010192     4500 <2e-04 ***
## ---
## Signif. codes:  0 '***' 0.001 '**' 0.01 '*' 0.05 '.' 0.1 ' ' 1
```

```r
# FishId + MomId + no YearBatch
model_control_hetero_residual_id_slopes_mom_noYearBatch <- readRDS("model_MeanCorner_control_hetero_residual
# model_control_hetero_residual_id_slopes_mom_noYearBatch
# <- MCMCglmm(MeanCorner_hourly_scaled ~ ExpDay + Hour,
# random = ~us(1 + ExpDay):FishId + MomId, rcov =
# ~idh(ExpDay_factor):units, family = 'gaussian', data =
# control_data_hourly, prior =
# prior.fishId.slopes.momId.univariate.idhExpDay, nitt =
# 500000, burnin = 50000, thin = 100)
# saveRDS(model_control_hetero_residual_id_slopes_mom_noYearBatch,
# file =
# 'model_MeanCorner_control_hetero_residual_id_slopes_mom_noYearBatch_8_22_25.rds')
summary(model_control_hetero_residual_id_slopes_mom_noYearBatch)  # Including YearBatch doesn't improve the
```

```
##
##  Iterations = 50001:499901
##  Thinning interval  = 100
##  Sample size  = 4500
##
##  DIC: 35108.31
##
##  G-structure:  ~us(1 + ExpDay):FishId
##
##                                 post.mean   l-95% CI   u-95% CI eff.samp
```

```
## (Intercept):(Intercept).FishId  0.2777012  0.1654644  0.4042967     2865
## ExpDay:(Intercept).FishId      -0.0049335 -0.0091496 -0.0013675     4500
## (Intercept):ExpDay.FishId      -0.0049335 -0.0091496 -0.0013675     4500
## ExpDay:ExpDay.FishId            0.0005447  0.0003314  0.0007938     3307
##
##                ~MomId
##
##       post.mean  l-95% CI u-95% CI eff.samp
## MomId    0.0229 5.188e-09  0.08453     1660
##
##  R-structure:  ~idh(ExpDay_factor):units
##
##                       post.mean l-95% CI u-95% CI eff.samp
## ExpDay_factor0.units     1.2365   1.1037   1.3897     4500
## ExpDay_factor1.units     0.7808   0.6901   0.8662     4500
## ExpDay_factor2.units     0.8602   0.7649   0.9593     4500
## ExpDay_factor3.units     0.8142   0.7180   0.9063     4941
## ExpDay_factor4.units     0.7431   0.6605   0.8303     4500
## ExpDay_factor5.units     0.4149   0.3692   0.4635     4500
## ExpDay_factor6.units     0.4095   0.3630   0.4599     4227
## ExpDay_factor7.units     0.6171   0.5489   0.6868     4500
## ExpDay_factor8.units     0.4330   0.3843   0.4842     4500
## ExpDay_factor9.units     0.5709   0.5102   0.6367     5381
## ExpDay_factor10.units    0.6702   0.5838   0.7517     4500
## ExpDay_factor11.units    0.4976   0.4389   0.5583     4157
## ExpDay_factor12.units    0.4884   0.4350   0.5516     4779
## ExpDay_factor13.units    0.4868   0.4309   0.5424     4500
## ExpDay_factor14.units    0.4782   0.4242   0.5344     4500
## ExpDay_factor15.units    0.6255   0.5546   0.6952     4500
## ExpDay_factor16.units    0.5732   0.5029   0.6390     4500
## ExpDay_factor17.units    0.5385   0.4790   0.6006     4699
## ExpDay_factor18.units    0.5076   0.4471   0.5687     4500
## ExpDay_factor19.units    0.5275   0.4619   0.5859     4545
## ExpDay_factor20.units    0.5949   0.5274   0.6665     4255
## ExpDay_factor21.units    0.5816   0.5156   0.6471     4500
## ExpDay_factor22.units    0.5139   0.4540   0.5705     4500
## ExpDay_factor23.units    0.4318   0.3816   0.4826     4959
## ExpDay_factor24.units    0.4908   0.4401   0.5523     4259
## ExpDay_factor25.units    0.3329   0.2941   0.3742     4500
## ExpDay_factor26.units    0.4702   0.4152   0.5258     4500
## ExpDay_factor27.units    0.4667   0.4126   0.5243     4306
##
##  Location effects: MeanCorner_hourly_scaled ~ ExpDay + Hour
##
##             post.mean  l-95% CI  u-95% CI eff.samp  pMCMC
## (Intercept)  0.004320 -0.172855  0.169455     4500 0.9671
## ExpDay      -0.006082 -0.012828  0.001215     4500 0.0853 .
## Hour        -0.016407 -0.019799 -0.013052     4713 <2e-04 ***
## ---
## Signif. codes:  0 '***' 0.001 '**' 0.01 '*' 0.05 '.' 0.1 ' ' 1
```

```
# FishId + MomId + no YearBatch or Hour
model_control_hetero_residual_id_slopes_mom_noHour <- readRDS("model_MeanCorner_control_hetero_residual_id_s
# model_control_hetero_residual_id_slopes_mom_noHour <-
# MCMCglmm(MeanCorner_hourly_scaled ~ ExpDay, random =
# ~us(1 + ExpDay):FishId + MomId, rcov =
# ~idh(ExpDay_factor):units, family = 'gaussian', data =
# control_data_hourly, prior =
# prior.fishId.slopes.momId.univariate.idhExpDay, nitt =
# 500000, burnin = 50000, thin = 100)
# saveRDS(model_control_hetero_residual_id_slopes_mom_noHour,
# file =
```

```r
# 'model_MeanCorner_control_hetero_residual_id_slopes_mom_noHour_8_22_25.rds')
summary(model_control_hetero_residual_id_slopes_mom_noHour)  # Excluding Hour is worse fit
```

```
##
##  Iterations = 50001:499901
##  Thinning interval  = 100
##  Sample size  = 4500
##
##  DIC: 35198.65
##
##  G-structure:  ~us(1 + ExpDay):FishId
##
##                                post.mean   l-95% CI   u-95% CI eff.samp
## (Intercept):(Intercept).FishId  0.2761985  0.1676092  0.3982095     3074
## ExpDay:(Intercept).FishId      -0.0049362 -0.0092677 -0.0011689     4500
## (Intercept):ExpDay.FishId      -0.0049362 -0.0092677 -0.0011689     4500
## ExpDay:ExpDay.FishId            0.0005436  0.0003295  0.0007965     3642
##
##                ~MomId
##
##       post.mean  l-95% CI u-95% CI eff.samp
## MomId   0.02223 5.141e-09  0.08022     1630
##
##  R-structure:  ~idh(ExpDay_factor):units
##
##                         post.mean l-95% CI u-95% CI eff.samp
## ExpDay_factor0.units      1.2246   1.0878   1.3672     4500
## ExpDay_factor1.units      0.7782   0.6928   0.8671     4500
## ExpDay_factor2.units      0.8502   0.7577   0.9560     4500
## ExpDay_factor3.units      0.8002   0.7079   0.8943     4500
## ExpDay_factor4.units      0.7404   0.6618   0.8278     4500
## ExpDay_factor5.units      0.4177   0.3704   0.4665     4300
## ExpDay_factor6.units      0.4098   0.3579   0.4571     4500
## ExpDay_factor7.units      0.6086   0.5416   0.6801     4500
## ExpDay_factor8.units      0.4348   0.3875   0.4869     4500
## ExpDay_factor9.units      0.5679   0.5000   0.6304     4500
## ExpDay_factor10.units     0.6610   0.5799   0.7456     4500
## ExpDay_factor11.units     0.5079   0.4475   0.5682     4500
## ExpDay_factor12.units     0.4868   0.4297   0.5445     4500
## ExpDay_factor13.units     0.4952   0.4396   0.5530     4500
## ExpDay_factor14.units     0.4871   0.4326   0.5450     4775
## ExpDay_factor15.units     0.6411   0.5714   0.7156     4500
## ExpDay_factor16.units     0.5784   0.5143   0.6502     5036
## ExpDay_factor17.units     0.5496   0.4871   0.6112     4500
## ExpDay_factor18.units     0.5125   0.4544   0.5744     4500
## ExpDay_factor19.units     0.5321   0.4702   0.5914     4500
## ExpDay_factor20.units     0.6030   0.5360   0.6756     4500
## ExpDay_factor21.units     0.5877   0.5238   0.6567     4500
## ExpDay_factor22.units     0.5175   0.4571   0.5766     4500
## ExpDay_factor23.units     0.4387   0.3888   0.4937     4217
## ExpDay_factor24.units     0.4868   0.4299   0.5443     4500
## ExpDay_factor25.units     0.3437   0.3045   0.3877     4500
## ExpDay_factor26.units     0.4776   0.4173   0.5310     4292
## ExpDay_factor27.units     0.4728   0.4190   0.5334     4500
##
##  Location effects: MeanCorner_hourly_scaled ~ ExpDay
##
##             post.mean  l-95% CI  u-95% CI eff.samp  pMCMC
## (Intercept) -0.087433 -0.254951  0.082609     4500 0.3129
## ExpDay      -0.006148 -0.012482  0.001316     4500 0.0804 .
## ---
```

```
## Signif. codes:  0 '***' 0.001 '**' 0.01 '*' 0.05 '.' 0.1 ' ' 1
```

```r
# Diagnostics on best fit model
autocorr.diag(model_control_hetero_residual_id_slopes_mom$Sol)
```

```
##           (Intercept)       ExpDay   YearBatch          Hour
## Lag 0     1.000000000  1.000000000 1.000000000  1.000000e+00
## Lag 100   0.014780475 -0.003178578 0.014294379 -3.293027e-02
## Lag 500   0.014614231  0.014336058 0.029036747  3.660581e-05
## Lag 1000  0.007091533 -0.002341523 0.006643051 -2.730940e-04
## Lag 5000 -0.004080740 -0.016872704 0.013801960 -4.133233e-04
```

```r
autocorr.diag(model_control_hetero_residual_id_slopes_mom$VCV)
```

```
##          (Intercept):(Intercept).FishId ExpDay:(Intercept).FishId
## Lag 0                       1.000000000               1.000000000
## Lag 100                     0.070832285              -0.012398718
## Lag 500                     0.015853251              -0.025479399
## Lag 1000                    0.059919046               0.001882291
## Lag 5000                   -0.002927164              -0.026272857
##          (Intercept):ExpDay.FishId ExpDay:ExpDay.FishId      MomId
## Lag 0                  1.000000000          1.000000000 1.00000000
## Lag 100               -0.012398718          0.042108087 0.41582931
## Lag 500               -0.025479399          0.033980826 0.12461297
## Lag 1000               0.001882291          0.003759570 0.01904171
## Lag 5000              -0.026272857         -0.003819706 0.03836322
##          ExpDay_factor0.units ExpDay_factor1.units ExpDay_factor2.units
## Lag 0             1.000000000          1.000000000          1.000000000
## Lag 100           0.034667119         -0.016261548         -0.006424099
## Lag 500          -0.008102153         -0.024635890         -0.004808557
## Lag 1000         -0.009305677          0.009319712          0.012606024
## Lag 5000         -0.014173166         -0.002530095         -0.012287242
##          ExpDay_factor3.units ExpDay_factor4.units ExpDay_factor5.units
## Lag 0              1.00000000          1.000000000          1.000000000
## Lag 100            0.00324835         -0.002122435         -0.010949687
## Lag 500            0.00677824          0.002468282         -0.038296920
## Lag 1000          -0.01333268         -0.008601431         -0.022504229
## Lag 5000           0.01676477         -0.009032723         -0.004910379
##          ExpDay_factor6.units ExpDay_factor7.units ExpDay_factor8.units
## Lag 0             1.000000000          1.000000000          1.000000000
## Lag 100          -0.023340173          0.012341126         -0.018311648
## Lag 500           0.003694409          0.001436300         -0.010590918
## Lag 1000          0.020343894         -0.002462723         -0.018905559
## Lag 5000          0.004659854         -0.005223474          0.002330046
##          ExpDay_factor9.units ExpDay_factor10.units ExpDay_factor11.units
## Lag 0             1.000000000           1.000000000           1.000000000
## Lag 100          -0.014627299           0.000702962           0.018422719
## Lag 500          -0.011777246           0.003294552          -0.010903002
## Lag 1000          0.019591138          -0.001367315           0.009530223
## Lag 5000         -0.005092754          -0.003992787           0.007417775
##          ExpDay_factor12.units ExpDay_factor13.units ExpDay_factor14.units
## Lag 0             1.0000000000           1.000000000           1.000000000
## Lag 100          -0.0203254095          -0.007650041          -0.018435917
## Lag 500          -0.0098056719           0.023970897          -0.016322174
## Lag 1000          0.0009045795           0.011068323          -0.002052446
## Lag 5000          0.0070638598          -0.005221940          -0.001001067
##          ExpDay_factor15.units ExpDay_factor16.units ExpDay_factor17.units
## Lag 0               1.00000000           1.000000000           1.000000000
## Lag 100            -0.02052855          -0.002456624          -0.022598518
## Lag 500            -0.02261023           0.021870368           0.001143011
## Lag 1000           -0.02608239          -0.006308265           0.009686706
## Lag 5000            0.02680034          -0.001126448           0.025794616
##          ExpDay_factor18.units ExpDay_factor19.units ExpDay_factor20.units
```

```
## Lag 0               1.000000000           1.00000000           1.00000000
## Lag 100            -0.008814493          -0.01787173           0.02134467
## Lag 500            -0.015032786          -0.01203229          -0.01772082
## Lag 1000            0.010901107           0.01084569          -0.00198637
## Lag 5000           -0.010976682          -0.00280995           0.01631050
##          ExpDay_factor21.units ExpDay_factor22.units ExpDay_factor23.units
## Lag 0               1.000000000          1.0000000000           1.000000000
## Lag 100             0.001338932          0.0005639873           0.014539318
## Lag 500             0.027008801         -0.0060917033          -0.029298949
## Lag 1000            0.001312724         -0.0199538349          -0.009701497
## Lag 5000            0.016049348         -0.0017982602          -0.023683545
##          ExpDay_factor24.units ExpDay_factor25.units ExpDay_factor26.units
## Lag 0               1.000000000          1.0000000000           1.000000000
## Lag 100             0.016692614          0.0129254948          -0.006718853
## Lag 500            -0.002958982          0.0070799871          -0.014456566
## Lag 1000           -0.020443471         -0.0007872146          -0.010527475
## Lag 5000           -0.016552079          0.0105747891           0.004341107
##          ExpDay_factor27.units
## Lag 0              1.0000000000
## Lag 100            0.0005960182
## Lag 500           -0.0258803906
## Lag 1000           0.0060889700
## Lag 5000          -0.0102446783
```

```r
effectiveSize(model_control_hetero_residual_id_slopes_mom$Sol)
```

```
## (Intercept)      ExpDay   YearBatch        Hour
##    3943.261    4500.000    4500.000    4591.163
```

```r
effectiveSize(model_control_hetero_residual_id_slopes_mom$VCV)  # Numbers are a bit low, so will try the mod
```

```
## (Intercept):(Intercept).FishId      ExpDay:(Intercept).FishId
##                       2373.955                       4500.000
##      (Intercept):ExpDay.FishId           ExpDay:ExpDay.FishId
##                       4500.000                       3216.104
##                          MomId           ExpDay_factor0.units
##                       1111.704                       4197.517
##           ExpDay_factor1.units           ExpDay_factor2.units
##                       4500.000                       4500.000
##           ExpDay_factor3.units           ExpDay_factor4.units
##                       4500.000                       4500.000
##           ExpDay_factor5.units           ExpDay_factor6.units
##                       4500.000                       4498.513
##           ExpDay_factor7.units           ExpDay_factor8.units
##                       4500.000                       4500.000
##           ExpDay_factor9.units          ExpDay_factor10.units
##                       4883.812                       4500.000
##          ExpDay_factor11.units          ExpDay_factor12.units
##                       4575.769                       4500.000
##          ExpDay_factor13.units          ExpDay_factor14.units
##                       4500.000                       4500.000
##          ExpDay_factor15.units          ExpDay_factor16.units
##                       4500.000                       4500.000
##          ExpDay_factor17.units          ExpDay_factor18.units
##                       4707.043                       4294.707
##          ExpDay_factor19.units          ExpDay_factor20.units
##                       4500.000                       5019.971
##          ExpDay_factor21.units          ExpDay_factor22.units
##                       4500.000                       4500.000
##          ExpDay_factor23.units          ExpDay_factor24.units
##                       5261.842                       4500.000
##          ExpDay_factor25.units          ExpDay_factor26.units
##                       4500.000                       4500.000
```

```
##          ExpDay_factor27.units
##                       4682.898
```

```
# FishId slopes + MomId with more iterations
model_control_hetero_residual_id_slopes_mom_more_iterations <- readRDS("model_MeanCorner_control_hetero_res
# model_control_hetero_residual_id_slopes_mom_hour_included
# <- MCMCglmm(MeanCorner_hourly_scaled ~ ExpDay + Hour,
# random = ~us(1 + ExpDay):FishId + MomId, rcov =
# ~idh(ExpDay_factor):units, family = 'gaussian', data =
# control_data_hourly, prior =
# prior.fishId.slopes.momId.univariate.idhExpDay, nitt =
# 1500000, burnin = 150000, thin = 200)
# saveRDS(model_control_hetero_residual_id_slopes_mom_more_iterations,
# file =
# 'model_MeanCorner_control_hetero_residual_id_slopes_mom_hour_included_8_7_25_more_iterations.rds')
summary(model_control_hetero_residual_id_slopes_mom_more_iterations)  #
```

```
##
##  Iterations = 150001:1499801
##  Thinning interval  = 200
##  Sample size  = 6750
##
##  DIC: 35108.26
##
##  G-structure:  ~us(1 + ExpDay):FishId
##
##                                  post.mean   l-95% CI   u-95% CI eff.samp
## (Intercept):(Intercept).FishId  0.2752095  0.1676556  0.3969180     5560
## ExpDay:(Intercept).FishId      -0.0048906 -0.0089720 -0.0012354     6750
## (Intercept):ExpDay.FishId      -0.0048906 -0.0089720 -0.0012354     6750
## ExpDay:ExpDay.FishId            0.0005439  0.0003326  0.0007877     5856
##
##                ~MomId
##
##       post.mean  l-95% CI u-95% CI eff.samp
## MomId   0.02294 1.559e-10  0.08357     3780
##
##  R-structure:  ~idh(ExpDay_factor):units
##
##                       post.mean l-95% CI u-95% CI eff.samp
## ExpDay_factor0.units     1.2359   1.1001   1.3876     6750
## ExpDay_factor1.units     0.7799   0.6915   0.8658     6744
## ExpDay_factor2.units     0.8614   0.7633   0.9591     6750
## ExpDay_factor3.units     0.8159   0.7254   0.9186     6750
## ExpDay_factor4.units     0.7431   0.6562   0.8263     6235
## ExpDay_factor5.units     0.4147   0.3692   0.4633     6750
## ExpDay_factor6.units     0.4101   0.3628   0.4631     6750
## ExpDay_factor7.units     0.6161   0.5452   0.6843     6750
## ExpDay_factor8.units     0.4328   0.3862   0.4837     6750
## ExpDay_factor9.units     0.5714   0.5075   0.6368     6750
## ExpDay_factor10.units    0.6709   0.5877   0.7506     6750
## ExpDay_factor11.units    0.4974   0.4396   0.5579     6941
## ExpDay_factor12.units    0.4876   0.4313   0.5457     6750
## ExpDay_factor13.units    0.4867   0.4327   0.5423     6750
## ExpDay_factor14.units    0.4781   0.4255   0.5319     6750
## ExpDay_factor15.units    0.6259   0.5569   0.6986     6750
## ExpDay_factor16.units    0.5737   0.5119   0.6407     6750
## ExpDay_factor17.units    0.5391   0.4784   0.6002     6750
## ExpDay_factor18.units    0.5073   0.4477   0.5666     6750
## ExpDay_factor19.units    0.5272   0.4641   0.5871     6750
## ExpDay_factor20.units    0.5948   0.5284   0.6659     6673
## ExpDay_factor21.units    0.5819   0.5199   0.6538     6750
```

```
## ExpDay_factor22.units    0.5143   0.4519   0.5698     6996
## ExpDay_factor23.units    0.4327   0.3815   0.4849     6020
## ExpDay_factor24.units    0.4903   0.4340   0.5469     6750
## ExpDay_factor25.units    0.3333   0.2925   0.3726     6750
## ExpDay_factor26.units    0.4693   0.4165   0.5277     6750
## ExpDay_factor27.units    0.4655   0.4126   0.5241     6750
##
##  Location effects: MeanCorner_hourly_scaled ~ ExpDay + Hour
##
##             post.mean   l-95% CI   u-95% CI eff.samp  pMCMC
## (Intercept)  0.0025241 -0.1713306  0.1768594     6750 0.9790
## ExpDay      -0.0060738 -0.0130320  0.0006474     6750 0.0738 .
## Hour        -0.0163804 -0.0197386 -0.0130495     6750 <1e-04 ***
## ---
## Signif. codes:  0 '***' 0.001 '**' 0.01 '*' 0.05 '.' 0.1 ' ' 1
```

```
# Diagnostics on best fit model
autocorr.diag(model_control_hetero_residual_id_slopes_mom_more_iterations$Sol)
```

```
##            (Intercept)       ExpDay          Hour
## Lag 0     1.0000000000  1.000000000  1.000000e+00
## Lag 200   0.0040644762 -0.009596215  3.113204e-03
## Lag 1000  0.0005711312  0.006815619  2.164565e-02
## Lag 2000  0.0086806943 -0.011774665  2.003511e-02
## Lag 10000 0.0063500539 -0.008436682 -5.754622e-05
```

```
autocorr.diag(model_control_hetero_residual_id_slopes_mom_more_iterations$VCV)
```

```
##           (Intercept):(Intercept).FishId ExpDay:(Intercept).FishId
## Lag 0                        1.000000000               1.000000000
## Lag 200                      0.046603291              -0.003365518
## Lag 1000                     0.021894729               0.004529702
## Lag 2000                     0.018237576               0.005970120
## Lag 10000                    0.007707798               0.003921222
##           (Intercept):ExpDay.FishId ExpDay:ExpDay.FishId       MomId
## Lag 0                   1.000000000          1.000000000  1.00000000
## Lag 200                -0.003365518          0.028986571  0.20888682
## Lag 1000                0.004529702          0.013813988  0.02814681
## Lag 2000                0.005970120         -0.012620357  0.02317554
## Lag 10000               0.003921222         -0.005408579 -0.01174122
##           ExpDay_factor0.units ExpDay_factor1.units ExpDay_factor2.units
## Lag 0             1.0000000000          1.000000000         1.0000000000
## Lag 200          -0.0011166532          0.019181999        -0.0003741418
## Lag 1000         -0.0005729098          0.016869181         0.0073961801
## Lag 2000          0.0023833596         -0.001911760        -0.0012638681
## Lag 10000         0.0127746984          0.007300661        -0.0118383614
##           ExpDay_factor3.units ExpDay_factor4.units ExpDay_factor5.units
## Lag 0              1.000000000          1.000000000          1.000000000
## Lag 200            0.008310660          0.026001060         -0.011434516
## Lag 1000           0.005817239          0.018611860         -0.012945379
## Lag 2000          -0.016954987          0.008616641          0.008095227
## Lag 10000          0.017784772          0.037807269         -0.007988676
##           ExpDay_factor6.units ExpDay_factor7.units ExpDay_factor8.units
## Lag 0              1.000000000         1.0000000000          1.000000000
## Lag 200           -0.004740684         0.0009804685         -0.001079387
## Lag 1000           0.008480628        -0.0126215297         -0.013263623
## Lag 2000          -0.001982191         0.0103357130          0.004097904
## Lag 10000          0.016026605         0.0016392976         -0.023051811
##           ExpDay_factor9.units ExpDay_factor10.units ExpDay_factor11.units
## Lag 0              1.000000000           1.000000000           1.000000000
## Lag 200            0.001775443          -0.008103046          -0.016091991
## Lag 1000          -0.030683763           0.007089781          -0.005854648
## Lag 2000          -0.004242909           0.014960547           0.001824130
```

```
## Lag 10000          0.013985988          -0.018223165          -0.013360158
##            ExpDay_factor12.units ExpDay_factor13.units ExpDay_factor14.units
## Lag 0                1.000000000           1.000000000           1.000000000
## Lag 200              0.002030475          -0.008193441           0.012375204
## Lag 1000            -0.002530228           0.000937896          -0.002265242
## Lag 2000            -0.002858256          -0.016257269          -0.010626507
## Lag 10000            0.008070481           0.001028385          -0.010435721
##            ExpDay_factor15.units ExpDay_factor16.units ExpDay_factor17.units
## Lag 0                1.000000000           1.000000000           1.000000000
## Lag 200              0.004350604          -0.003078932           0.007518658
## Lag 1000            -0.004393517          -0.006339500           0.004211933
## Lag 2000             0.020782917           0.019045453           0.002247210
## Lag 10000           -0.034964058          -0.008943081           0.007999807
##            ExpDay_factor18.units ExpDay_factor19.units ExpDay_factor20.units
## Lag 0                1.000000000           1.000000000           1.000000000
## Lag 200             -0.006617371          -0.003086568          -0.019058144
## Lag 1000            -0.006622534          -0.003506475           0.005177668
## Lag 2000             0.003085908           0.002688913           0.003054279
## Lag 10000           -0.003528461          -0.002508260          -0.012119094
##            ExpDay_factor21.units ExpDay_factor22.units ExpDay_factor23.units
## Lag 0                1.000000000           1.000000000           1.000000000
## Lag 200              0.001455486          -0.017973058           0.034793380
## Lag 1000            -0.022305147          -0.008392222           0.004860909
## Lag 2000            -0.003842011          -0.017973797          -0.010598395
## Lag 10000            0.022298404           0.008924457           0.009288213
##            ExpDay_factor24.units ExpDay_factor25.units ExpDay_factor26.units
## Lag 0                1.000000000           1.000000000          1.0000000000
## Lag 200              0.009066123          -0.005268235         -0.0070871664
## Lag 1000            -0.015785205           0.002702150         -0.0150755711
## Lag 2000            -0.028431744          -0.019055160          0.0130235384
## Lag 10000            0.008006083          -0.002460489         -0.0004734303
##            ExpDay_factor27.units
## Lag 0                 1.00000000
## Lag 200               0.01224387
## Lag 1000             -0.01221781
## Lag 2000             -0.01752365
## Lag 10000            -0.02391015
```

```
effectiveSize(model_control_hetero_residual_id_slopes_mom_more_iterations$Sol)
```

```
## (Intercept)      ExpDay        Hour
##        6750        6750        6750
```

```
effectiveSize(model_control_hetero_residual_id_slopes_mom_more_iterations$VCV)
```

```
## (Intercept):(Intercept).FishId      ExpDay:(Intercept).FishId
##                       5559.653                       6750.000
##      (Intercept):ExpDay.FishId           ExpDay:ExpDay.FishId
##                       6750.000                       5856.063
##                          MomId          ExpDay_factor0.units
##                       3779.679                       6750.000
##           ExpDay_factor1.units           ExpDay_factor2.units
##                       6744.267                       6750.000
##           ExpDay_factor3.units           ExpDay_factor4.units
##                       6750.000                       6235.185
##           ExpDay_factor5.units           ExpDay_factor6.units
##                       6750.000                       6750.000
##           ExpDay_factor7.units           ExpDay_factor8.units
##                       6750.000                       6750.000
##           ExpDay_factor9.units          ExpDay_factor10.units
##                       6750.000                       6750.000
##          ExpDay_factor11.units          ExpDay_factor12.units
##                       6941.288                       6750.000
```

```
##          ExpDay_factor13.units          ExpDay_factor14.units
##                       6750.000                       6750.000
##          ExpDay_factor15.units          ExpDay_factor16.units
##                       6750.000                       6750.000
##          ExpDay_factor17.units          ExpDay_factor18.units
##                       6750.000                       6750.000
##          ExpDay_factor19.units          ExpDay_factor20.units
##                       6750.000                       6673.497
##          ExpDay_factor21.units          ExpDay_factor22.units
##                       6750.000                       6996.040
##          ExpDay_factor23.units          ExpDay_factor24.units
##                       6019.623                       6750.000
##          ExpDay_factor25.units          ExpDay_factor26.units
##                       6750.000                       6750.000
##          ExpDay_factor27.units
##                       6750.000
```

Model comparison to determine included random effects for PREDATOR

```r
# Null
model_predator_hetero_residual_null <- readRDS("model_MeanCorner_predator_hetero_residual_null_8_22_25.rds")
# model_predator_hetero_residual_null <-
# MCMCglmm(MeanCorner_hourly_scaled ~ ExpDay + YearBatch +
# Hour, rcov = ~idh(ExpDay_factor):units, family =
# 'gaussian', data = predator_data_hourly, prior =
# prior.null.univariate.idhExpDay, nitt = 500000, burnin =
# 50000, thin = 100)
# saveRDS(model_predator_hetero_residual_null, file =
# 'model_MeanCorner_predator_hetero_residual_null_8_22_25.rds')
summary(model_predator_hetero_residual_null)
```

```
##
##  Iterations = 50001:499901
##  Thinning interval  = 100
##  Sample size  = 4500
##
##  DIC: 49578.58
##
##  R-structure:  ~idh(ExpDay_factor):units
##
##                       post.mean l-95% CI u-95% CI eff.samp
## ExpDay_factor0.units     1.6413   1.4612   1.8320     4500
## ExpDay_factor1.units     1.6272   1.4557   1.8000     4500
## ExpDay_factor2.units     1.6842   1.5035   1.8659     4500
## ExpDay_factor3.units     1.4486   1.3011   1.6077     4500
## ExpDay_factor4.units     1.4006   1.2509   1.5574     4839
## ExpDay_factor5.units     1.0319   0.9177   1.1492     4500
## ExpDay_factor6.units     1.0809   0.9698   1.2077     4500
## ExpDay_factor7.units     0.9566   0.8540   1.0624     4500
## ExpDay_factor8.units     0.8459   0.7536   0.9410     3926
## ExpDay_factor9.units     1.0381   0.9287   1.1538     4500
## ExpDay_factor10.units    0.9532   0.8487   1.0569     4500
## ExpDay_factor11.units    0.8643   0.7663   0.9605     4694
## ExpDay_factor12.units    1.0895   0.9609   1.1994     4500
## ExpDay_factor13.units    0.9257   0.8230   1.0248     4500
## ExpDay_factor14.units    0.7543   0.6716   0.8395     4500
## ExpDay_factor15.units    0.9331   0.8318   1.0376     4500
## ExpDay_factor16.units    1.0789   0.9637   1.2028     4500
## ExpDay_factor17.units    0.9433   0.8419   1.0480     4136
## ExpDay_factor18.units    0.8682   0.7659   0.9607     4500
## ExpDay_factor19.units    0.8541   0.7623   0.9507     4500
```

```
## ExpDay_factor20.units    0.8987   0.8054   0.9971     4500
## ExpDay_factor21.units    1.0292   0.9193   1.1474     4500
## ExpDay_factor22.units    0.9371   0.8405   1.0485     4500
## ExpDay_factor23.units    0.9061   0.8046   1.0007     4500
## ExpDay_factor24.units    0.9988   0.8883   1.1082     4500
## ExpDay_factor25.units    1.0068   0.8946   1.1164     4125
## ExpDay_factor26.units    1.0618   0.9502   1.1841     3781
## ExpDay_factor27.units    0.8946   0.7957   0.9928     4500
##
##  Location effects: MeanCorner_hourly_scaled ~ ExpDay + YearBatch + Hour
##
##               post.mean   l-95% CI   u-95% CI eff.samp  pMCMC
## (Intercept)  0.3054183  0.2535270  0.3580576     4538 <2e-04 ***
## ExpDay      -0.0006238 -0.0025312  0.0013674     4500  0.544
## YearBatch   -0.0265451 -0.0400546 -0.0125808     4500 <2e-04 ***
## Hour        -0.0146360 -0.0192882 -0.0105494     4500 <2e-04 ***
## ---
## Signif. codes:  0 '***' 0.001 '**' 0.01 '*' 0.05 '.' 0.1 ' ' 1
```

```r
# FishId
model_predator_hetero_residual_id <- readRDS("model_MeanCorner_predator_hetero_residual_id_8_22_25.rds")
# model_predator_hetero_residual_id <-
# MCMCglmm(MeanCorner_hourly_scaled ~ ExpDay + YearBatch +
# Hour, random = ~FishId, rcov = ~idh(ExpDay_factor):units,
# family = 'gaussian', data = predator_data_hourly, prior =
# prior.fishId.univariate.idhExpDay, nitt = 500000, burnin
# = 50000, thin = 100)
# saveRDS(model_predator_hetero_residual_id, file =
# 'model_MeanCorner_predator_hetero_residual_id_8_22_25.rds')
summary(model_predator_hetero_residual_id)
```

```
##
##  Iterations = 50001:499901
##  Thinning interval  = 100
##  Sample size  = 4500
##
##  DIC: 45196.2
##
##  G-structure:  ~FishId
##
##         post.mean l-95% CI u-95% CI eff.samp
## FishId    0.2648   0.1687    0.375     3087
##
##  R-structure:  ~idh(ExpDay_factor):units
##
##                        post.mean l-95% CI u-95% CI eff.samp
## ExpDay_factor0.units     1.5936   1.4236   1.7720     4500
## ExpDay_factor1.units     1.4360   1.2885   1.5942     4500
## ExpDay_factor2.units     1.5280   1.3714   1.7089     4500
## ExpDay_factor3.units     1.3572   1.2228   1.5132     4500
## ExpDay_factor4.units     1.3005   1.1669   1.4545     4500
## ExpDay_factor5.units     0.8583   0.7624   0.9508     4500
## ExpDay_factor6.units     0.9893   0.8879   1.1024     4500
## ExpDay_factor7.units     0.8196   0.7307   0.9159     4281
## ExpDay_factor8.units     0.6499   0.5803   0.7226     4500
## ExpDay_factor9.units     0.8412   0.7520   0.9387     4500
## ExpDay_factor10.units    0.6707   0.5999   0.7452     4740
## ExpDay_factor11.units    0.6600   0.5858   0.7299     4500
## ExpDay_factor12.units    0.8442   0.7539   0.9419     4500
## ExpDay_factor13.units    0.6878   0.6159   0.7663     4500
## ExpDay_factor14.units    0.5847   0.5220   0.6506     4500
## ExpDay_factor15.units    0.6732   0.5970   0.7439     4500
```

```
## ExpDay_factor16.units    0.8767   0.7841   0.9782     4482
## ExpDay_factor17.units    0.7087   0.6302   0.7864     4500
## ExpDay_factor18.units    0.6789   0.6058   0.7544     4757
## ExpDay_factor19.units    0.5951   0.5287   0.6596     3601
## ExpDay_factor20.units    0.6527   0.5801   0.7255     4500
## ExpDay_factor21.units    0.7181   0.6428   0.7989     4500
## ExpDay_factor22.units    0.6609   0.5885   0.7334     4846
## ExpDay_factor23.units    0.5731   0.5072   0.6355     4500
## ExpDay_factor24.units    0.6637   0.5943   0.7429     4500
## ExpDay_factor25.units    0.6801   0.6117   0.7585     4799
## ExpDay_factor26.units    0.7685   0.6867   0.8566     4500
## ExpDay_factor27.units    0.6553   0.5782   0.7254     4500
##
##  Location effects: MeanCorner_hourly_scaled ~ ExpDay + YearBatch + Hour
##
##              post.mean   l-95% CI   u-95% CI eff.samp  pMCMC
## (Intercept)  0.2092032 -0.1141020  0.5211358     4500  0.213
## ExpDay      -0.0008083 -0.0025296  0.0010163     4500  0.366
## YearBatch    0.0114293 -0.1071058  0.1329483     4350  0.852
## Hour        -0.0153836 -0.0193843 -0.0116307     4500 <2e-04 ***
## ---
## Signif. codes:  0 '***' 0.001 '**' 0.01 '*' 0.05 '.' 0.1 ' ' 1
```

```r
# MomId
model_predator_hetero_residual_mom <- readRDS("model_MeanCorner_predator_hetero_residual_mom_8_22_25.rds")
# model_predator_hetero_residual_mom <-
# MCMCglmm(MeanCorner_hourly_scaled ~ ExpDay + YearBatch +
# Hour, random = ~MomId, rcov = ~idh(ExpDay_factor):units,
# family = 'gaussian', data = predator_data_hourly, prior =
# prior.momId.univariate.idhExpDay, nitt = 500000, burnin =
# 50000, thin = 100)
# saveRDS(model_predator_hetero_residual_mom, file =
# 'model_MeanCorner_predator_hetero_residual_mom_8_22_25.rds')
summary(model_predator_hetero_residual_mom)
```

```
##
##  Iterations = 50001:499901
##  Thinning interval  = 100
##  Sample size  = 4500
##
##  DIC: 48356.53
##
##  G-structure:  ~MomId
##
##       post.mean l-95% CI u-95% CI eff.samp
## MomId    0.1481   0.0453   0.3093     2454
##
##  R-structure:  ~idh(ExpDay_factor):units
##
##                         post.mean l-95% CI u-95% CI eff.samp
## ExpDay_factor0.units       1.6504   1.4624   1.8219     4500
## ExpDay_factor1.units       1.5396   1.3780   1.7108     4500
## ExpDay_factor2.units       1.5994   1.4347   1.7753     5120
## ExpDay_factor3.units       1.4219   1.2733   1.5840     4500
## ExpDay_factor4.units       1.3476   1.2101   1.5072     4500
## ExpDay_factor5.units       0.9622   0.8552   1.0659     4500
## ExpDay_factor6.units       0.9782   0.8700   1.0894     4500
## ExpDay_factor7.units       0.9058   0.8090   1.0033     4500
## ExpDay_factor8.units       0.7133   0.6414   0.7977     4787
## ExpDay_factor9.units       0.9639   0.8626   1.0692     4500
## ExpDay_factor10.units      0.8380   0.7492   0.9345     4711
## ExpDay_factor11.units      0.8345   0.7442   0.9275     4500
```

```
## ExpDay_factor12.units    1.0061   0.8944   1.1137     4500
## ExpDay_factor13.units    0.8891   0.7952   0.9866     4500
## ExpDay_factor14.units    0.7277   0.6473   0.8114     4758
## ExpDay_factor15.units    0.8510   0.7603   0.9436     4294
## ExpDay_factor16.units    1.0420   0.9346   1.1640     4500
## ExpDay_factor17.units    0.8688   0.7735   0.9629     4753
## ExpDay_factor18.units    0.8424   0.7504   0.9330     4500
## ExpDay_factor19.units    0.7863   0.7058   0.8797     4500
## ExpDay_factor20.units    0.8699   0.7750   0.9645     3913
## ExpDay_factor21.units    0.9576   0.8554   1.0636     5453
## ExpDay_factor22.units    0.8701   0.7741   0.9692     4500
## ExpDay_factor23.units    0.7963   0.7126   0.8853     4500
## ExpDay_factor24.units    0.8848   0.7942   0.9887     4362
## ExpDay_factor25.units    0.9122   0.8128   1.0131     4500
## ExpDay_factor26.units    0.9683   0.8601   1.0771     4500
## ExpDay_factor27.units    0.8283   0.7374   0.9206     4500
##
##  Location effects: MeanCorner_hourly_scaled ~ ExpDay + YearBatch + Hour
##
##              post.mean   l-95% CI   u-95% CI eff.samp  pMCMC
## (Intercept)  0.3875639 -0.0342515  0.8219674     4500 0.0818 .
## ExpDay      -0.0005488 -0.0025170  0.0012155     4500 0.5729
## YearBatch   -0.0417360 -0.2002546  0.1251394     4813 0.5844
## Hour        -0.0145488 -0.0187231 -0.0102696     4500 <2e-04 ***
## ---
## Signif. codes:  0 '***' 0.001 '**' 0.01 '*' 0.05 '.' 0.1 ' ' 1
```

```r
# FishId + MomId
model_predator_hetero_residual_id_mom <- readRDS("model_MeanCorner_predator_hetero_residual_id_mom_8_22_25.r
# model_predator_hetero_residual_id_mom <-
# MCMCglmm(MeanCorner_hourly_scaled ~ ExpDay + YearBatch +
# Hour, random = ~FishId + MomId, rcov =
# ~idh(ExpDay_factor):units, family = 'gaussian', data =
# predator_data_hourly, prior =
# prior.fishId.momId.univariate.idhExpDay, nitt = 500000,
# burnin = 50000, thin = 100)
# saveRDS(model_predator_hetero_residual_id_mom, file =
# 'model_MeanCorner_predator_hetero_residual_id_mom_8_22_25.rds')
summary(model_predator_hetero_residual_id_mom)
```

```
##
##  Iterations = 50001:499901
##  Thinning interval  = 100
##  Sample size  = 4500
##
##  DIC: 45196.38
##
##  G-structure:  ~FishId
##
##        post.mean l-95% CI u-95% CI eff.samp
## FishId    0.2522    0.157   0.3596     4007
##
##                ~MomId
##
##       post.mean l-95% CI u-95% CI eff.samp
## MomId   0.03449 5.83e-09   0.1271     1641
##
##  R-structure:  ~idh(ExpDay_factor):units
##
##                       post.mean l-95% CI u-95% CI eff.samp
## ExpDay_factor0.units     1.5937   1.4304   1.7767     4500
## ExpDay_factor1.units     1.4318   1.2924   1.6007     4500
```

```
## ExpDay_factor2.units     1.5266   1.3603   1.6907     4500
## ExpDay_factor3.units     1.3606   1.2045   1.5091     4648
## ExpDay_factor4.units     1.3031   1.1733   1.4666     4500
## ExpDay_factor5.units     0.8585   0.7631   0.9474     4500
## ExpDay_factor6.units     0.9893   0.8876   1.0985     4500
## ExpDay_factor7.units     0.8214   0.7314   0.9144     4500
## ExpDay_factor8.units     0.6482   0.5765   0.7204     4312
## ExpDay_factor9.units     0.8395   0.7483   0.9328     4500
## ExpDay_factor10.units    0.6704   0.5983   0.7497     4344
## ExpDay_factor11.units    0.6590   0.5854   0.7314     4500
## ExpDay_factor12.units    0.8432   0.7491   0.9344     4500
## ExpDay_factor13.units    0.6874   0.6164   0.7674     4500
## ExpDay_factor14.units    0.5856   0.5204   0.6496     4500
## ExpDay_factor15.units    0.6744   0.6010   0.7487     4500
## ExpDay_factor16.units    0.8751   0.7810   0.9749     4188
## ExpDay_factor17.units    0.7092   0.6301   0.7871     4500
## ExpDay_factor18.units    0.6804   0.6092   0.7577     4500
## ExpDay_factor19.units    0.5943   0.5298   0.6597     4500
## ExpDay_factor20.units    0.6529   0.5863   0.7272     4500
## ExpDay_factor21.units    0.7178   0.6392   0.7959     4500
## ExpDay_factor22.units    0.6621   0.5913   0.7376     4500
## ExpDay_factor23.units    0.5732   0.5121   0.6389     4500
## ExpDay_factor24.units    0.6649   0.5918   0.7403     4500
## ExpDay_factor25.units    0.6793   0.6062   0.7580     4500
## ExpDay_factor26.units    0.7682   0.6829   0.8535     4500
## ExpDay_factor27.units    0.6555   0.5807   0.7313     4500
##
##  Location effects: MeanCorner_hourly_scaled ~ ExpDay + YearBatch + Hour
##
##              post.mean   l-95% CI   u-95% CI eff.samp  pMCMC
## (Intercept)  0.2296928 -0.1355769  0.6062265     5010  0.227
## ExpDay      -0.0008204 -0.0026350  0.0008183     4500  0.360
## YearBatch    0.0066468 -0.1394631  0.1449039     5038  0.931
## Hour        -0.0153998 -0.0191946 -0.0116169     4694 <2e-04 ***
## ---
## Signif. codes:  0 '***' 0.001 '**' 0.01 '*' 0.05 '.' 0.1 ' ' 1
```

```r
# FishId slopes
model_predator_hetero_residual_id_slopes <- readRDS("model_MeanCorner_predator_hetero_residual_id_slopes_8_2
# model_predator_hetero_residual_id_slopes <-
# MCMCglmm(MeanCorner_hourly_scaled ~ ExpDay + YearBatch +
# Hour, random = ~us(1 + ExpDay):FishId, rcov =
# ~idh(ExpDay_factor):units, family = 'gaussian', data =
# predator_data_hourly, prior =
# prior.fishId.slopes.univariate.idhExpDay, nitt = 500000,
# burnin = 50000, thin = 100)
# saveRDS(model_predator_hetero_residual_id_slopes, file =
# 'model_MeanCorner_predator_hetero_residual_id_slopes_8_22_25.rds')
summary(model_predator_hetero_residual_id_slopes)
```

```
##
##  Iterations = 50001:499901
##  Thinning interval  = 100
##  Sample size  = 4500
##
##  DIC: 44386.56
##
##  G-structure:  ~us(1 + ExpDay):FishId
##
##                              post.mean   l-95% CI   u-95% CI eff.samp
## (Intercept):(Intercept).FishId  0.303641  0.1850524  0.426635     3594
## ExpDay:(Intercept).FishId      -0.006296 -0.0111301 -0.001746     4500
```

```
## (Intercept):ExpDay.FishId      -0.006296 -0.0111301 -0.001746     4500
## ExpDay:ExpDay.FishId            0.000747  0.0004634  0.001091     3247
##
##  R-structure:  ~idh(ExpDay_factor):units
##
##                        post.mean l-95% CI u-95% CI eff.samp
## ExpDay_factor0.units     1.4191   1.2739   1.5890     4500
## ExpDay_factor1.units     1.2720   1.1345   1.4153     4500
## ExpDay_factor2.units     1.4102   1.2633   1.5737     4500
## ExpDay_factor3.units     1.2650   1.1285   1.4089     4500
## ExpDay_factor4.units     1.2458   1.1200   1.3912     4500
## ExpDay_factor5.units     0.8319   0.7435   0.9272     4500
## ExpDay_factor6.units     0.8924   0.7989   0.9931     4500
## ExpDay_factor7.units     0.7506   0.6645   0.8280     4500
## ExpDay_factor8.units     0.6006   0.5360   0.6676     4500
## ExpDay_factor9.units     0.8233   0.7366   0.9168     4500
## ExpDay_factor10.units    0.6565   0.5841   0.7296     4500
## ExpDay_factor11.units    0.6503   0.5762   0.7180     6402
## ExpDay_factor12.units    0.8376   0.7487   0.9342     4500
## ExpDay_factor13.units    0.6915   0.6158   0.7708     4500
## ExpDay_factor14.units    0.5860   0.5236   0.6529     4748
## ExpDay_factor15.units    0.6754   0.6032   0.7537     4847
## ExpDay_factor16.units    0.8715   0.7777   0.9681     4500
## ExpDay_factor17.units    0.7008   0.6279   0.7836     4500
## ExpDay_factor18.units    0.6716   0.6009   0.7451     4091
## ExpDay_factor19.units    0.5838   0.5208   0.6490     4500
## ExpDay_factor20.units    0.6429   0.5725   0.7173     4500
## ExpDay_factor21.units    0.6833   0.6133   0.7613     4500
## ExpDay_factor22.units    0.6159   0.5461   0.6841     4710
## ExpDay_factor23.units    0.5140   0.4581   0.5708     4690
## ExpDay_factor24.units    0.5836   0.5209   0.6483     4267
## ExpDay_factor25.units    0.6105   0.5439   0.6759     4823
## ExpDay_factor26.units    0.7439   0.6611   0.8276     4500
## ExpDay_factor27.units    0.6507   0.5772   0.7289     4500
##
##  Location effects: MeanCorner_hourly_scaled ~ ExpDay + YearBatch + Hour
##
##             post.mean  l-95% CI  u-95% CI eff.samp  pMCMC
## (Intercept)  0.145971 -0.173590  0.463009     4500  0.379
## ExpDay      -0.001181 -0.008877  0.006662     4500  0.764
## YearBatch    0.039457 -0.073846  0.163461     4500  0.500
## Hour        -0.015087 -0.018773 -0.011332     4753 <2e-04 ***
## ---
## Signif. codes:  0 '***' 0.001 '**' 0.01 '*' 0.05 '.' 0.1 ' ' 1
```

```r
# FishId slopes + MomId
model_predator_hetero_residual_id_slopes_mom <- readRDS("model_MeanCorner_predator_hetero_residual_id_slopes
# model_predator_hetero_residual_id_slopes_mom <-
# MCMCglmm(MeanCorner_hourly_scaled ~ ExpDay + YearBatch +
# Hour, random = ~us(1 + ExpDay):FishId + MomId, rcov =
# ~idh(ExpDay_factor):units, family = 'gaussian', data =
# predator_data_hourly, prior =
# prior.fishId.slopes.momId.univariate.idhExpDay, nitt =
# 500000, burnin = 50000, thin = 100)
# saveRDS(model_predator_hetero_residual_id_slopes_mom,
# file =
# 'model_MeanCorner_predator_hetero_residual_id_slopes_mom_8_22_25.rds')
summary(model_predator_hetero_residual_id_slopes_mom)  # Best fit that includes MomId
```

```
##
##  Iterations = 50001:499901
##  Thinning interval  = 100
```

```
##  Sample size  = 4500
##
##  DIC: 44386.43
##
##  G-structure:  ~us(1 + ExpDay):FishId
##
##                                post.mean   l-95% CI  u-95% CI eff.samp
## (Intercept):(Intercept).FishId  0.2816862  0.1676355  0.411640     3291
## ExpDay:(Intercept).FishId      -0.0061796 -0.0110112 -0.001250     4500
## (Intercept):ExpDay.FishId      -0.0061796 -0.0110112 -0.001250     4500
## ExpDay:ExpDay.FishId            0.0007591  0.0004686  0.001112     3982
##
##                ~MomId
##
##       post.mean  l-95% CI u-95% CI eff.samp
## MomId   0.04397 1.014e-08    0.144     1622
##
##  R-structure:  ~idh(ExpDay_factor):units
##
##                       post.mean l-95% CI u-95% CI eff.samp
## ExpDay_factor0.units     1.4180   1.2588   1.5678     4882
## ExpDay_factor1.units     1.2734   1.1335   1.4170     4678
## ExpDay_factor2.units     1.4086   1.2668   1.5706     4500
## ExpDay_factor3.units     1.2663   1.1241   1.4009     4500
## ExpDay_factor4.units     1.2463   1.1127   1.3898     4500
## ExpDay_factor5.units     0.8307   0.7435   0.9262     5145
## ExpDay_factor6.units     0.8937   0.7986   0.9993     4500
## ExpDay_factor7.units     0.7502   0.6716   0.8355     4745
## ExpDay_factor8.units     0.6009   0.5368   0.6660     4500
## ExpDay_factor9.units     0.8224   0.7345   0.9127     4500
## ExpDay_factor10.units    0.6556   0.5840   0.7293     4500
## ExpDay_factor11.units    0.6496   0.5785   0.7249     4292
## ExpDay_factor12.units    0.8378   0.7476   0.9314     4500
## ExpDay_factor13.units    0.6924   0.6187   0.7704     4072
## ExpDay_factor14.units    0.5854   0.5242   0.6528     4296
## ExpDay_factor15.units    0.6748   0.6041   0.7523     4500
## ExpDay_factor16.units    0.8745   0.7795   0.9739     4437
## ExpDay_factor17.units    0.7012   0.6269   0.7807     4157
## ExpDay_factor18.units    0.6713   0.5961   0.7430     4500
## ExpDay_factor19.units    0.5831   0.5182   0.6475     4500
## ExpDay_factor20.units    0.6429   0.5759   0.7164     4500
## ExpDay_factor21.units    0.6835   0.6089   0.7633     4500
## ExpDay_factor22.units    0.6152   0.5489   0.6817     4500
## ExpDay_factor23.units    0.5128   0.4591   0.5705     4500
## ExpDay_factor24.units    0.5827   0.5190   0.6500     4500
## ExpDay_factor25.units    0.6117   0.5397   0.6765     4500
## ExpDay_factor26.units    0.7448   0.6635   0.8273     4500
## ExpDay_factor27.units    0.6522   0.5789   0.7260     4500
##
##  Location effects: MeanCorner_hourly_scaled ~ ExpDay + YearBatch + Hour
##
##             post.mean  l-95% CI  u-95% CI eff.samp  pMCMC
## (Intercept)  0.141851 -0.227550  0.548683     4182  0.440
## ExpDay      -0.001082 -0.008885  0.006268     4344  0.788
## YearBatch    0.048221 -0.105374  0.192248     4225  0.507
## Hour        -0.015131 -0.018719 -0.011221     4095 <2e-04 ***
## ---
## Signif. codes:  0 '***' 0.001 '**' 0.01 '*' 0.05 '.' 0.1 ' ' 1
```

```
# FishId slopes + MomId slopes
model_predator_hetero_residual_id_slopes_mom_slopes <- readRDS("model_MeanCorner_predator_hetero_residual_id
# model_predator_hetero_residual_id_slopes_mom_slopes <-
```

```r
# MCMCglmm(MeanCorner_hourly_scaled ~ ExpDay + YearBatch +
# Hour, random = ~us(1 + ExpDay):FishId + us(1 +
# ExpDay):MomId, rcov = ~idh(ExpDay_factor):units, family =
# 'gaussian', data = predator_data_hourly, prior =
# prior.fishId.slopes.momId.slopes.univariate.idhExpDay,
# nitt = 500000, burnin = 50000, thin = 100)
# saveRDS(model_predator_hetero_residual_id_slopes_mom_slopes,
# file =
# 'model_MeanCorner_predator_hetero_residual_id_slopes_mom_slopes_8_22_25.rds')
summary(model_predator_hetero_residual_id_slopes_mom_slopes)
```

```
##
##  Iterations = 50001:499901
##  Thinning interval  = 100
##  Sample size  = 4500
##
##  DIC: 44386.54
##
##  G-structure:  ~us(1 + ExpDay):FishId
##
##                                 post.mean   l-95% CI u-95% CI eff.samp
## (Intercept):(Intercept).FishId  0.2791684  0.1706606  0.40729     3320
## ExpDay:(Intercept).FishId      -0.0059775 -0.0110838 -0.00156     5014
## (Intercept):ExpDay.FishId      -0.0059775 -0.0110838 -0.00156     5014
## ExpDay:ExpDay.FishId            0.0007382  0.0004514  0.00107     4176
##
##               ~us(1 + ExpDay):MomId
##
##                                post.mean   l-95% CI  u-95% CI eff.samp
## (Intercept):(Intercept).MomId  5.064e-02  1.001e-08 0.1707995     1344
## ExpDay:(Intercept).MomId      -2.147e-04 -2.250e-03 0.0013114     4500
## (Intercept):ExpDay.MomId      -2.147e-04 -2.250e-03 0.0013114     4500
## ExpDay:ExpDay.MomId            5.319e-05  2.815e-11 0.0001993     2639
##
##  R-structure:  ~idh(ExpDay_factor):units
##
##                       post.mean l-95% CI u-95% CI eff.samp
## ExpDay_factor0.units     1.4173   1.2653   1.5746     4500
## ExpDay_factor1.units     1.2717   1.1390   1.4112     4500
## ExpDay_factor2.units     1.4091   1.2534   1.5669     4500
## ExpDay_factor3.units     1.2675   1.1278   1.4043     4500
## ExpDay_factor4.units     1.2471   1.1170   1.3939     4500
## ExpDay_factor5.units     0.8300   0.7398   0.9205     4500
## ExpDay_factor6.units     0.8917   0.7975   0.9927     4750
## ExpDay_factor7.units     0.7519   0.6691   0.8327     4500
## ExpDay_factor8.units     0.6005   0.5344   0.6689     4500
## ExpDay_factor9.units     0.8230   0.7345   0.9184     4500
## ExpDay_factor10.units    0.6579   0.5897   0.7333     4500
## ExpDay_factor11.units    0.6505   0.5789   0.7224     4500
## ExpDay_factor12.units    0.8379   0.7505   0.9334     4500
## ExpDay_factor13.units    0.6925   0.6178   0.7704     4500
## ExpDay_factor14.units    0.5845   0.5226   0.6494     4500
## ExpDay_factor15.units    0.6743   0.6047   0.7525     4741
## ExpDay_factor16.units    0.8739   0.7790   0.9716     4500
## ExpDay_factor17.units    0.7002   0.6233   0.7789     4500
## ExpDay_factor18.units    0.6719   0.5983   0.7487     4500
## ExpDay_factor19.units    0.5833   0.5190   0.6476     4500
## ExpDay_factor20.units    0.6424   0.5732   0.7129     4500
## ExpDay_factor21.units    0.6826   0.6101   0.7571     4762
## ExpDay_factor22.units    0.6152   0.5494   0.6845     4500
## ExpDay_factor23.units    0.5138   0.4590   0.5738     4500
```

```
## ExpDay_factor24.units    0.5824   0.5182   0.6482     4500
## ExpDay_factor25.units    0.6119   0.5453   0.6791     4500
## ExpDay_factor26.units    0.7456   0.6596   0.8287     4745
## ExpDay_factor27.units    0.6518   0.5724   0.7239     4500
##
##  Location effects: MeanCorner_hourly_scaled ~ ExpDay + YearBatch + Hour
##
##             post.mean  l-95% CI  u-95% CI eff.samp  pMCMC
## (Intercept)  0.135603 -0.277817  0.502778     4500  0.475
## ExpDay      -0.001122 -0.009716  0.007856     4500  0.773
## YearBatch    0.049976 -0.097472  0.200711     4500  0.501
## Hour        -0.015093 -0.018625 -0.011205     4287 <2e-04 ***
## ---
## Signif. codes:  0 '***' 0.001 '**' 0.01 '*' 0.05 '.' 0.1 ' ' 1
```

```r
# FishId slopes + MomId + no YearBatch
model_predator_hetero_residual_id_slopes_mom_noYearBatch <- readRDS("model_MeanCorner_predator_hetero_residu
# model_predator_hetero_residual_id_slopes_mom_noYearBatch
# <- MCMCglmm(MeanCorner_hourly_scaled ~ ExpDay + Hour,
# random = ~us(1 + ExpDay):FishId + MomId, rcov =
# ~idh(ExpDay_factor):units, family = 'gaussian', data =
# predator_data_hourly, prior =
# prior.fishId.slopes.momId.univariate.idhExpDay, nitt =
# 500000, burnin = 50000, thin = 100)
# saveRDS(model_predator_hetero_residual_id_slopes_mom_noYearBatch,
# file =
# 'model_MeanCorner_predator_hetero_residual_id_slopes_mom_noYearBatch_8_22_25.rds')
summary(model_predator_hetero_residual_id_slopes_mom_noYearBatch)  # Best fit -- simplest within 2 DIC
```

```
##
##  Iterations = 50001:499901
##  Thinning interval  = 100
##  Sample size  = 4500
##
##  DIC: 44387.21
##
##  G-structure:  ~us(1 + ExpDay):FishId
##
##                                post.mean   l-95% CI  u-95% CI eff.samp
## (Intercept):(Intercept).FishId  0.2786315  0.1700669  0.402344     3685
## ExpDay:(Intercept).FishId      -0.0062633 -0.0113353 -0.002067     4500
## (Intercept):ExpDay.FishId      -0.0062633 -0.0113353 -0.002067     4500
## ExpDay:ExpDay.FishId            0.0007512  0.0004567  0.001082     3869
##
##                ~MomId
##
##       post.mean  l-95% CI u-95% CI eff.samp
## MomId   0.03671 6.762e-10    0.122     1239
##
##  R-structure:  ~idh(ExpDay_factor):units
##
##                       post.mean l-95% CI u-95% CI eff.samp
## ExpDay_factor0.units     1.4183   1.2637   1.5754     4500
## ExpDay_factor1.units     1.2724   1.1339   1.4162     4500
## ExpDay_factor2.units     1.4100   1.2591   1.5650     4500
## ExpDay_factor3.units     1.2672   1.1342   1.4160     4500
## ExpDay_factor4.units     1.2465   1.1038   1.3821     4491
## ExpDay_factor5.units     0.8302   0.7425   0.9242     4815
## ExpDay_factor6.units     0.8919   0.7951   0.9895     4748
## ExpDay_factor7.units     0.7514   0.6691   0.8352     4500
## ExpDay_factor8.units     0.6014   0.5336   0.6675     4500
## ExpDay_factor9.units     0.8224   0.7358   0.9124     4500
```

```
## ExpDay_factor10.units    0.6569   0.5884   0.7310     4500
## ExpDay_factor11.units    0.6493   0.5795   0.7215     4500
## ExpDay_factor12.units    0.8382   0.7534   0.9346     4500
## ExpDay_factor13.units    0.6923   0.6154   0.7652     4709
## ExpDay_factor14.units    0.5846   0.5213   0.6484     4500
## ExpDay_factor15.units    0.6748   0.5979   0.7459     4500
## ExpDay_factor16.units    0.8724   0.7841   0.9741     4500
## ExpDay_factor17.units    0.7009   0.6223   0.7794     4500
## ExpDay_factor18.units    0.6715   0.5965   0.7431     4500
## ExpDay_factor19.units    0.5832   0.5194   0.6484     4500
## ExpDay_factor20.units    0.6423   0.5706   0.7118     4500
## ExpDay_factor21.units    0.6826   0.6105   0.7648     4500
## ExpDay_factor22.units    0.6157   0.5483   0.6826     4500
## ExpDay_factor23.units    0.5141   0.4579   0.5692     4727
## ExpDay_factor24.units    0.5838   0.5225   0.6508     4500
## ExpDay_factor25.units    0.6111   0.5430   0.6791     4259
## ExpDay_factor26.units    0.7446   0.6656   0.8366     4877
## ExpDay_factor27.units    0.6510   0.5831   0.7337     4500
##
##  Location effects: MeanCorner_hourly_scaled ~ ExpDay + Hour
##
##             post.mean  l-95% CI  u-95% CI eff.samp  pMCMC
## (Intercept)  0.254158  0.074903  0.437947     4448 0.0102 *
## ExpDay      -0.001113 -0.009103  0.006470     4500 0.7738
## Hour        -0.015125 -0.018900 -0.011434     4500 <2e-04 ***
## ---
## Signif. codes:  0 '***' 0.001 '**' 0.01 '*' 0.05 '.' 0.1 ' ' 1
```

```
# FishId slopes + MomId + no Hour
model_predator_hetero_residual_id_slopes_mom_noHour <- readRDS("model_MeanCorner_predator_hetero_residual_id
# model_predator_hetero_residual_id_slopes_mom_noHour <-
# MCMCglmm(MeanCorner_hourly_scaled ~ ExpDay, random =
# ~us(1 + ExpDay):FishId + MomId, rcov =
# ~idh(ExpDay_factor):units, family = 'gaussian', data =
# predator_data_hourly, prior =
# prior.fishId.slopes.momId.univariate.idhExpDay, nitt =
# 500000, burnin = 50000, thin = 100)
# saveRDS(model_predator_hetero_residual_id_slopes_mom_noHour,
# file =
# 'model_MeanCorner_predator_hetero_residual_id_slopes_mom_noHour_8_22_25.rds')
summary(model_predator_hetero_residual_id_slopes_mom_noHour)
```

```
##
##  Iterations = 50001:499901
##  Thinning interval  = 100
##  Sample size  = 4500
##
##  DIC: 44447.16
##
##  G-structure:  ~us(1 + ExpDay):FishId
##
##                                   post.mean   l-95% CI  u-95% CI eff.samp
## (Intercept):(Intercept).FishId  0.2783473  0.1695921  0.403823     2738
## ExpDay:(Intercept).FishId      -0.0062317 -0.0110963 -0.001901     4500
## (Intercept):ExpDay.FishId      -0.0062317 -0.0110963 -0.001901     4500
## ExpDay:ExpDay.FishId            0.0007457  0.0004603  0.001070     4070
##
##                ~MomId
##
##       post.mean  l-95% CI u-95% CI eff.samp
## MomId    0.0361 1.441e-09   0.1209     1744
##
```

```
##  R-structure:  ~idh(ExpDay_factor):units
##
##                        post.mean l-95% CI u-95% CI eff.samp
## ExpDay_factor0.units      1.4181   1.2689   1.5917     4500
## ExpDay_factor1.units      1.2609   1.1297   1.4021     4500
## ExpDay_factor2.units      1.4039   1.2517   1.5538     4822
## ExpDay_factor3.units      1.2696   1.1369   1.4066     4500
## ExpDay_factor4.units      1.2437   1.1070   1.3797     4500
## ExpDay_factor5.units      0.8304   0.7422   0.9244     4876
## ExpDay_factor6.units      0.8842   0.7914   0.9829     4500
## ExpDay_factor7.units      0.7444   0.6654   0.8301     4991
## ExpDay_factor8.units      0.5975   0.5297   0.6607     4500
## ExpDay_factor9.units      0.8351   0.7462   0.9345     4699
## ExpDay_factor10.units     0.6549   0.5863   0.7301     4262
## ExpDay_factor11.units     0.6513   0.5824   0.7279     4500
## ExpDay_factor12.units     0.8381   0.7451   0.9309     4483
## ExpDay_factor13.units     0.6999   0.6184   0.7741     4500
## ExpDay_factor14.units     0.5928   0.5277   0.6597     4500
## ExpDay_factor15.units     0.6826   0.6080   0.7583     4500
## ExpDay_factor16.units     0.8855   0.7912   0.9871     4500
## ExpDay_factor17.units     0.7015   0.6270   0.7809     4875
## ExpDay_factor18.units     0.6700   0.6001   0.7476     4894
## ExpDay_factor19.units     0.5904   0.5265   0.6592     4500
## ExpDay_factor20.units     0.6498   0.5779   0.7236     4500
## ExpDay_factor21.units     0.6918   0.6149   0.7697     4500
## ExpDay_factor22.units     0.6175   0.5524   0.6857     5110
## ExpDay_factor23.units     0.5143   0.4551   0.5716     4500
## ExpDay_factor24.units     0.5879   0.5240   0.6574     4086
## ExpDay_factor25.units     0.6197   0.5518   0.6882     4500
## ExpDay_factor26.units     0.7493   0.6662   0.8362     4500
## ExpDay_factor27.units     0.6579   0.5870   0.7372     4500
##
##  Location effects: MeanCorner_hourly_scaled ~ ExpDay
##
##             post.mean  l-95% CI  u-95% CI eff.samp  pMCMC
## (Intercept)  0.169532 -0.000164  0.350974     3990 0.0551 .
## ExpDay      -0.001164 -0.009117  0.006397     4500 0.7773
## ---
## Signif. codes:  0 '***' 0.001 '**' 0.01 '*' 0.05 '.' 0.1 ' ' 1
```

```
# Diagnostics on best fit model
autocorr.diag(model_predator_hetero_residual_id_slopes_mom_noYearBatch$Sol)
```

```
##           (Intercept)        ExpDay         Hour
## Lag 0     1.000000000  1.0000000000  1.000000000
## Lag 100  -0.003237861  0.0121838649 -0.013293592
## Lag 500  -0.019394660 -0.0143961187 -0.032282771
## Lag 1000 -0.003547813  0.0084664980 -0.003748410
## Lag 5000 -0.019048862 -0.0007968335 -0.002263434
```

```
autocorr.diag(model_predator_hetero_residual_id_slopes_mom_noYearBatch$VCV)
```

```
##          (Intercept):(Intercept).FishId ExpDay:(Intercept).FishId
## Lag 0                        1.00000000               1.000000000
## Lag 100                      0.03720371              -0.007482604
## Lag 500                      0.02273747              -0.012431161
## Lag 1000                     0.03147317              -0.012244676
## Lag 5000                     0.02043022               0.023299033
##          (Intercept):ExpDay.FishId ExpDay:ExpDay.FishId        MomId
## Lag 0                  1.000000000          1.000000000 1.0000000000
## Lag 100               -0.007482604          0.048614189 0.3865508798
## Lag 500               -0.012431161         -0.005920029 0.1090408280
## Lag 1000              -0.012244676          0.012683984 0.0453539357
```

```
## Lag 5000               0.023299033         0.009959338 0.0003682037
##           ExpDay_factor0.units ExpDay_factor1.units ExpDay_factor2.units
## Lag 0             1.0000000000          1.000000000         1.000000e+00
## Lag 100          -0.0003079472         -0.001858946        -9.656826e-05
## Lag 500           0.0027211866          0.009651988        -2.929223e-04
## Lag 1000         -0.0091683676          0.028178084        -1.235390e-02
## Lag 5000         -0.0130810490          0.016732740        -5.569109e-03
##           ExpDay_factor3.units ExpDay_factor4.units ExpDay_factor5.units
## Lag 0              1.000000000          1.000000000          1.000000000
## Lag 100            0.015374535         -0.015198364         -0.033892342
## Lag 500            0.015065205         -0.006694727          0.012260366
## Lag 1000          -0.002125044          0.023885577         -0.006135299
## Lag 5000           0.014104978          0.021351214          0.012373377
##           ExpDay_factor6.units ExpDay_factor7.units ExpDay_factor8.units
## Lag 0               1.00000000          1.000000000          1.000000000
## Lag 100            -0.02697467          0.015062945         -0.001286830
## Lag 500            -0.02018791          0.002658733          0.005659149
## Lag 1000           -0.02641400         -0.006937347         -0.011226677
## Lag 5000           -0.01073319          0.009987261         -0.009957234
##           ExpDay_factor9.units ExpDay_factor10.units ExpDay_factor11.units
## Lag 0             1.0000000000           1.000000000          1.0000000000
## Lag 100           0.0126627899          -0.010361646         -0.0075351183
## Lag 500          -0.0002318651           0.006146426          0.0004445968
## Lag 1000          0.0052014248           0.005043555          0.0040919408
## Lag 5000         -0.0132460399           0.024684905          0.0090925742
##           ExpDay_factor12.units ExpDay_factor13.units ExpDay_factor14.units
## Lag 0               1.000000000           1.000000000           1.000000000
## Lag 100             0.006724606          -0.022811205           0.016211482
## Lag 500            -0.016558138           0.003223563           0.002351443
## Lag 1000           -0.027308384           0.011017855          -0.023749690
## Lag 5000           -0.001239497          -0.022185978          -0.010179929
##           ExpDay_factor15.units ExpDay_factor16.units ExpDay_factor17.units
## Lag 0               1.000000000           1.000000000           1.000000000
## Lag 100            -0.004636961           0.006623774          -0.011477238
## Lag 500             0.006784598           0.009470000           0.021398193
## Lag 1000           -0.018917404           0.014052397          -0.007803479
## Lag 5000           -0.001682194          -0.014117031          -0.025585922
##           ExpDay_factor18.units ExpDay_factor19.units ExpDay_factor20.units
## Lag 0              1.0000000000           1.000000000          1.0000000000
## Lag 100           -0.0007851742          -0.019296661          0.0127510929
## Lag 500           -0.0072114082          -0.008536392          0.0002924993
## Lag 1000          -0.0158063971           0.016355325          0.0085781967
## Lag 5000          -0.0010785063          -0.025141284         -0.0019544730
##           ExpDay_factor21.units ExpDay_factor22.units ExpDay_factor23.units
## Lag 0               1.000000000          1.0000000000           1.000000000
## Lag 100             0.006790774          0.0093833557          -0.024682586
## Lag 500            -0.001110521         -0.0008244755          -0.008602703
## Lag 1000           -0.020148998         -0.0155370271          -0.009196372
## Lag 5000            0.021259998         -0.0113400506           0.016014255
##           ExpDay_factor24.units ExpDay_factor25.units ExpDay_factor26.units
## Lag 0               1.000000000          1.0000000000           1.000000000
## Lag 100             0.001160295          0.0273801239          -0.040290202
## Lag 500            -0.005836643          0.0003101248           0.018134717
## Lag 1000            0.010311958          0.0262295600           0.021125752
## Lag 5000            0.007363665         -0.0067063222          -0.006490192
##           ExpDay_factor27.units
## Lag 0               1.000000000
## Lag 100            -0.010692916
## Lag 500            -0.002690645
## Lag 1000            0.009017180
## Lag 5000            0.005515951
```

```r
effectiveSize(model_predator_hetero_residual_id_slopes_mom_noYearBatch$Sol)
```

```
## (Intercept)      ExpDay        Hour
##    4447.835    4500.000    4500.000
```

```r
effectiveSize(model_predator_hetero_residual_id_slopes_mom_noYearBatch$VCV)
```

```
## (Intercept):(Intercept).FishId      ExpDay:(Intercept).FishId
##                       3685.496                       4500.000
##      (Intercept):ExpDay.FishId           ExpDay:ExpDay.FishId
##                       4500.000                       3869.275
##                          MomId          ExpDay_factor0.units
##                       1239.046                       4500.000
##           ExpDay_factor1.units           ExpDay_factor2.units
##                       4500.000                       4500.000
##           ExpDay_factor3.units           ExpDay_factor4.units
##                       4500.000                       4490.931
##           ExpDay_factor5.units           ExpDay_factor6.units
##                       4814.662                       4748.447
##           ExpDay_factor7.units           ExpDay_factor8.units
##                       4500.000                       4500.000
##           ExpDay_factor9.units          ExpDay_factor10.units
##                       4500.000                       4500.000
##          ExpDay_factor11.units          ExpDay_factor12.units
##                       4500.000                       4500.000
##          ExpDay_factor13.units          ExpDay_factor14.units
##                       4709.046                       4500.000
##          ExpDay_factor15.units          ExpDay_factor16.units
##                       4500.000                       4500.000
##          ExpDay_factor17.units          ExpDay_factor18.units
##                       4500.000                       4500.000
##          ExpDay_factor19.units          ExpDay_factor20.units
##                       4500.000                       4500.000
##          ExpDay_factor21.units          ExpDay_factor22.units
##                       4500.000                       4500.000
##          ExpDay_factor23.units          ExpDay_factor24.units
##                       4726.714                       4500.000
##          ExpDay_factor25.units          ExpDay_factor26.units
##                       4259.199                       4876.751
##          ExpDay_factor27.units
##                       4500.000
```

```r
# Best fit model that matches control, FishId slopes +
# MomId, with more iterations
model_predator_hetero_residual_id_slopes_more_iterations <- readRDS("model_MeanCorner_predator_hetero_residu
# model_predator_hetero_residual_id_slopes_more_iterations
# <- MCMCglmm(MeanCorner_hourly_scaled ~ ExpDay + Hour,
# random = ~us(1 + ExpDay):FishId + MomId, rcov =
# ~idh(ExpDay_factor):units, family = 'gaussian', data =
# predator_data_hourly, prior =
# prior.fishId.slopes.momId.univariate.idhExpDay, nitt =
# 1500000, burnin = 150000, thin = 200)
# saveRDS(model_predator_hetero_residual_id_slopes_more_iterations,
# file =
# 'model_MeanCorner_predator_hetero_residual_id_slopes_mom_hour_included_8_8_25_more_iterations.rds')
summary(model_predator_hetero_residual_id_slopes_more_iterations)  #
```

```
##
##  Iterations = 150001:1499801
##  Thinning interval  = 200
##  Sample size  = 6750
##
```

```
##  DIC: 44387.19
##
##  G-structure:  ~us(1 + ExpDay):FishId
##
##                                  post.mean   l-95% CI  u-95% CI eff.samp
## (Intercept):(Intercept).FishId  0.2773437  0.1666234  0.401633     5826
## ExpDay:(Intercept).FishId      -0.0062128 -0.0109912 -0.001715     6572
## (Intercept):ExpDay.FishId      -0.0062128 -0.0109912 -0.001715     6572
## ExpDay:ExpDay.FishId            0.0007491  0.0004685  0.001081     5768
##
##                ~MomId
##
##       post.mean  l-95% CI u-95% CI eff.samp
## MomId   0.03634 2.741e-10   0.1213     3446
##
##  R-structure:  ~idh(ExpDay_factor):units
##
##                         post.mean l-95% CI u-95% CI eff.samp
## ExpDay_factor0.units      1.4174   1.2613   1.5768     6750
## ExpDay_factor1.units      1.2715   1.1423   1.4169     7278
## ExpDay_factor2.units      1.4091   1.2573   1.5622     6750
## ExpDay_factor3.units      1.2661   1.1224   1.4006     6750
## ExpDay_factor4.units      1.2468   1.1200   1.3970     6443
## ExpDay_factor5.units      0.8312   0.7428   0.9266     6750
## ExpDay_factor6.units      0.8922   0.7938   0.9920     6750
## ExpDay_factor7.units      0.7508   0.6716   0.8353     6750
## ExpDay_factor8.units      0.6011   0.5369   0.6728     6750
## ExpDay_factor9.units      0.8229   0.7276   0.9090     6750
## ExpDay_factor10.units     0.6567   0.5832   0.7289     6750
## ExpDay_factor11.units     0.6494   0.5752   0.7192     6750
## ExpDay_factor12.units     0.8380   0.7462   0.9299     6380
## ExpDay_factor13.units     0.6929   0.6149   0.7686     6750
## ExpDay_factor14.units     0.5858   0.5231   0.6501     6750
## ExpDay_factor15.units     0.6745   0.5981   0.7494     6397
## ExpDay_factor16.units     0.8733   0.7845   0.9784     6750
## ExpDay_factor17.units     0.7005   0.6202   0.7721     6511
## ExpDay_factor18.units     0.6715   0.5950   0.7441     6750
## ExpDay_factor19.units     0.5837   0.5214   0.6503     6750
## ExpDay_factor20.units     0.6417   0.5689   0.7126     6750
## ExpDay_factor21.units     0.6829   0.6091   0.7572     6432
## ExpDay_factor22.units     0.6152   0.5488   0.6857     6750
## ExpDay_factor23.units     0.5140   0.4570   0.5719     6750
## ExpDay_factor24.units     0.5835   0.5197   0.6473     6750
## ExpDay_factor25.units     0.6109   0.5475   0.6802     6750
## ExpDay_factor26.units     0.7445   0.6646   0.8327     6750
## ExpDay_factor27.units     0.6520   0.5820   0.7296     6750
##
##  Location effects: MeanCorner_hourly_scaled ~ ExpDay + Hour
##
##              post.mean  l-95% CI  u-95% CI eff.samp  pMCMC
## (Intercept)  0.253118  0.071033  0.439169     6407 0.0077 **
## ExpDay      -0.001123 -0.008827  0.006579     6750 0.7647
## Hour        -0.015077 -0.018850 -0.011402     6750 <1e-04 ***
## ---
## Signif. codes:  0 '***' 0.001 '**' 0.01 '*' 0.05 '.' 0.1 ' ' 1
```

```
# Diagnostics on best fit model
autocorr.diag(model_predator_hetero_residual_id_slopes_more_iterations$Sol)
```

```
##             (Intercept)       ExpDay         Hour
## Lag 0       1.000000000  1.000000000  1.000000000
## Lag 200     0.025979948 -0.016158204  0.001575836
```

```
## Lag 1000  -0.009422781 -0.019647428  0.021102185
## Lag 2000  -0.020102860  0.003902826 -0.015068333
## Lag 10000 -0.005413615 -0.019021335  0.002712114
```

```
autocorr.diag(model_predator_hetero_residual_id_slopes_more_iterations$VCV)
```

```
##           (Intercept):(Intercept).FishId ExpDay:(Intercept).FishId
## Lag 0                        1.000000000               1.000000000
## Lag 200                      0.017319641              -0.012762682
## Lag 1000                     0.004302343              -0.016760230
## Lag 2000                    -0.009908455              -0.006743251
## Lag 10000                    0.019537978               0.009772910
##           (Intercept):ExpDay.FishId ExpDay:ExpDay.FishId        MomId
## Lag 0                   1.000000000         1.0000000000  1.000000000
## Lag 200                -0.012762682         0.0181031227  0.259560126
## Lag 1000               -0.016760230        -0.0002779548 -0.006205447
## Lag 2000               -0.006743251         0.0169154239  0.004301705
## Lag 10000               0.009772910         0.0172076830 -0.010398877
##           ExpDay_factor0.units ExpDay_factor1.units ExpDay_factor2.units
## Lag 0              1.000000000          1.000000000         1.0000000000
## Lag 200           -0.002364418          0.003917981        -0.0005456544
## Lag 1000          -0.016493916         -0.008135809        -0.0242019152
## Lag 2000          -0.003345480         -0.005282380         0.0111869025
## Lag 10000         -0.004844576          0.013482315         0.0029622042
##           ExpDay_factor3.units ExpDay_factor4.units ExpDay_factor5.units
## Lag 0              1.000000000          1.000000000          1.000000000
## Lag 200           -0.015339736          0.023188494          0.004773004
## Lag 1000           0.003039125         -0.014332496          0.010108010
## Lag 2000           0.001625000          0.001747833         -0.001892904
## Lag 10000         -0.006759070          0.016788837         -0.026153361
##           ExpDay_factor6.units ExpDay_factor7.units ExpDay_factor8.units
## Lag 0              1.000000000         1.0000000000          1.000000000
## Lag 200           -0.010343779        -0.0009594766         -0.012704392
## Lag 1000          -0.006666905         0.0129863577         -0.002134996
## Lag 2000           0.017647090        -0.0023107506          0.012407374
## Lag 10000         -0.013982922         0.0067863477          0.003273788
##           ExpDay_factor9.units ExpDay_factor10.units ExpDay_factor11.units
## Lag 0             1.0000000000           1.000000000           1.000000000
## Lag 200           0.0047004367          -0.009045465           0.006367125
## Lag 1000         -0.0008611852          -0.012835255           0.004293081
## Lag 2000         -0.0019647761           0.003703858          -0.006031023
## Lag 10000        -0.0315343329          -0.014790528          -0.007011710
##           ExpDay_factor12.units ExpDay_factor13.units ExpDay_factor14.units
## Lag 0              1.000000e+00           1.000000000           1.000000000
## Lag 200            2.810651e-02           0.007219200          -0.008390232
## Lag 1000          -7.764841e-05          -0.017562030           0.013418270
## Lag 2000           1.634694e-03           0.002943571          -0.007000227
## Lag 10000          8.769778e-03           0.023595572           0.011547744
##           ExpDay_factor15.units ExpDay_factor16.units ExpDay_factor17.units
## Lag 0               1.000000000           1.000000000          1.0000000000
## Lag 200             0.026807625           0.004992914          0.0081906132
## Lag 1000            0.015776722           0.002987904          0.0251307652
## Lag 2000           -0.004832740          -0.018085983         -0.0083048461
## Lag 10000          -0.002046702           0.009685834         -0.0004097232
##           ExpDay_factor18.units ExpDay_factor19.units ExpDay_factor20.units
## Lag 0               1.000000000           1.000000000           1.000000000
## Lag 200            -0.002918306           0.014798714          -0.004840615
## Lag 1000           -0.010213150           0.007007101          -0.001069794
## Lag 2000            0.005318004           0.036829460          -0.021625039
## Lag 10000          -0.007345549           0.008312088          -0.013266072
##           ExpDay_factor21.units ExpDay_factor22.units ExpDay_factor23.units
## Lag 0               1.000000000           1.000000000           1.000000000
```

```
## Lag 200             0.007837501          -0.005191668           0.002597932
## Lag 1000           -0.009584850          -0.005558341          -0.012777808
## Lag 2000           -0.003635368          -0.014493246          -0.004428694
## Lag 10000           0.001440588          -0.018539745          -0.012082952
##            ExpDay_factor24.units ExpDay_factor25.units ExpDay_factor26.units
## Lag 0               1.000000000           1.000000000           1.000000000
## Lag 200             0.003373481          -0.015739836          -0.013256645
## Lag 1000           -0.004499340           0.002782380           0.002878213
## Lag 2000            0.017301860           0.003813371          -0.011882663
## Lag 10000          -0.009621836          -0.017502575           0.013574446
##            ExpDay_factor27.units
## Lag 0              1.0000000000
## Lag 200            0.0003271612
## Lag 1000          -0.0045992192
## Lag 2000          -0.0174929480
## Lag 10000         -0.0107003674
```

```
effectiveSize(model_predator_hetero_residual_id_slopes_more_iterations$Sol)
```

```
## (Intercept)      ExpDay        Hour
##    6407.202    6750.000    6750.000
```

```
effectiveSize(model_predator_hetero_residual_id_slopes_more_iterations$VCV)
```

```
## (Intercept):(Intercept).FishId      ExpDay:(Intercept).FishId
##                       5826.369                       6571.895
##      (Intercept):ExpDay.FishId           ExpDay:ExpDay.FishId
##                       6571.895                       5767.740
##                          MomId           ExpDay_factor0.units
##                       3445.802                       6750.000
##          ExpDay_factor1.units           ExpDay_factor2.units
##                       7277.645                       6750.000
##          ExpDay_factor3.units           ExpDay_factor4.units
##                       6750.000                       6443.095
##          ExpDay_factor5.units           ExpDay_factor6.units
##                       6750.000                       6750.000
##          ExpDay_factor7.units           ExpDay_factor8.units
##                       6750.000                       6750.000
##          ExpDay_factor9.units          ExpDay_factor10.units
##                       6750.000                       6750.000
##         ExpDay_factor11.units          ExpDay_factor12.units
##                       6750.000                       6379.990
##         ExpDay_factor13.units          ExpDay_factor14.units
##                       6750.000                       6750.000
##         ExpDay_factor15.units          ExpDay_factor16.units
##                       6396.598                       6750.000
##         ExpDay_factor17.units          ExpDay_factor18.units
##                       6511.132                       6750.000
##         ExpDay_factor19.units          ExpDay_factor20.units
##                       6750.000                       6750.000
##         ExpDay_factor21.units          ExpDay_factor22.units
##                       6432.243                       6750.000
##         ExpDay_factor23.units          ExpDay_factor24.units
##                       6750.000                       6750.000
##         ExpDay_factor25.units          ExpDay_factor26.units
##                       6750.000                       6750.000
##         ExpDay_factor27.units
##                       6750.000
```

Body size does not improve either model, so we can move on without including it.

Our final univariate models include: Fixed effects: ExpDay, Hour
Random effects: FishId with slopes, MomId

```r
model_control <- model_control_hetero_residual_id_slopes_mom_more_iterations
model_predator <- model_predator_hetero_residual_id_slopes_more_iterations
summary(model_control)
```

```
##
##  Iterations = 150001:1499801
##  Thinning interval  = 200
##  Sample size  = 6750
##
##  DIC: 35108.26
##
##  G-structure:  ~us(1 + ExpDay):FishId
##
##                                  post.mean   l-95% CI   u-95% CI eff.samp
## (Intercept):(Intercept).FishId  0.2752095  0.1676556  0.3969180     5560
## ExpDay:(Intercept).FishId      -0.0048906 -0.0089720 -0.0012354     6750
## (Intercept):ExpDay.FishId      -0.0048906 -0.0089720 -0.0012354     6750
## ExpDay:ExpDay.FishId            0.0005439  0.0003326  0.0007877     5856
##
##                ~MomId
##
##       post.mean  l-95% CI u-95% CI eff.samp
## MomId   0.02294 1.559e-10  0.08357     3780
##
##  R-structure:  ~idh(ExpDay_factor):units
##
##                         post.mean l-95% CI u-95% CI eff.samp
## ExpDay_factor0.units      1.2359   1.1001   1.3876     6750
## ExpDay_factor1.units      0.7799   0.6915   0.8658     6744
## ExpDay_factor2.units      0.8614   0.7633   0.9591     6750
## ExpDay_factor3.units      0.8159   0.7254   0.9186     6750
## ExpDay_factor4.units      0.7431   0.6562   0.8263     6235
## ExpDay_factor5.units      0.4147   0.3692   0.4633     6750
## ExpDay_factor6.units      0.4101   0.3628   0.4631     6750
## ExpDay_factor7.units      0.6161   0.5452   0.6843     6750
## ExpDay_factor8.units      0.4328   0.3862   0.4837     6750
## ExpDay_factor9.units      0.5714   0.5075   0.6368     6750
## ExpDay_factor10.units     0.6709   0.5877   0.7506     6750
## ExpDay_factor11.units     0.4974   0.4396   0.5579     6941
## ExpDay_factor12.units     0.4876   0.4313   0.5457     6750
## ExpDay_factor13.units     0.4867   0.4327   0.5423     6750
## ExpDay_factor14.units     0.4781   0.4255   0.5319     6750
## ExpDay_factor15.units     0.6259   0.5569   0.6986     6750
## ExpDay_factor16.units     0.5737   0.5119   0.6407     6750
## ExpDay_factor17.units     0.5391   0.4784   0.6002     6750
## ExpDay_factor18.units     0.5073   0.4477   0.5666     6750
## ExpDay_factor19.units     0.5272   0.4641   0.5871     6750
## ExpDay_factor20.units     0.5948   0.5284   0.6659     6673
## ExpDay_factor21.units     0.5819   0.5199   0.6538     6750
## ExpDay_factor22.units     0.5143   0.4519   0.5698     6996
## ExpDay_factor23.units     0.4327   0.3815   0.4849     6020
## ExpDay_factor24.units     0.4903   0.4340   0.5469     6750
## ExpDay_factor25.units     0.3333   0.2925   0.3726     6750
## ExpDay_factor26.units     0.4693   0.4165   0.5277     6750
## ExpDay_factor27.units     0.4655   0.4126   0.5241     6750
##
##  Location effects: MeanCorner_hourly_scaled ~ ExpDay + Hour
##
##               post.mean   l-95% CI   u-95% CI eff.samp  pMCMC
## (Intercept)  0.0025241 -0.1713306  0.1768594     6750 0.9790
## ExpDay      -0.0060738 -0.0130320  0.0006474     6750 0.0738 .
```

```
## Hour        -0.0163804 -0.0197386 -0.0130495     6750 <1e-04 ***
## ---
## Signif. codes:  0 '***' 0.001 '**' 0.01 '*' 0.05 '.' 0.1 ' ' 1
```

```r
summary(model_predator)
```

```
##
##  Iterations = 150001:1499801
##  Thinning interval  = 200
##  Sample size  = 6750
##
##  DIC: 44387.19
##
##  G-structure:  ~us(1 + ExpDay):FishId
##
##                                  post.mean   l-95% CI  u-95% CI eff.samp
## (Intercept):(Intercept).FishId  0.2773437  0.1666234  0.401633     5826
## ExpDay:(Intercept).FishId      -0.0062128 -0.0109912 -0.001715     6572
## (Intercept):ExpDay.FishId      -0.0062128 -0.0109912 -0.001715     6572
## ExpDay:ExpDay.FishId            0.0007491  0.0004685  0.001081     5768
##
##                ~MomId
##
##       post.mean  l-95% CI u-95% CI eff.samp
## MomId   0.03634 2.741e-10   0.1213     3446
##
##  R-structure:  ~idh(ExpDay_factor):units
##
##                         post.mean l-95% CI u-95% CI eff.samp
## ExpDay_factor0.units       1.4174   1.2613   1.5768     6750
## ExpDay_factor1.units       1.2715   1.1423   1.4169     7278
## ExpDay_factor2.units       1.4091   1.2573   1.5622     6750
## ExpDay_factor3.units       1.2661   1.1224   1.4006     6750
## ExpDay_factor4.units       1.2468   1.1200   1.3970     6443
## ExpDay_factor5.units       0.8312   0.7428   0.9266     6750
## ExpDay_factor6.units       0.8922   0.7938   0.9920     6750
## ExpDay_factor7.units       0.7508   0.6716   0.8353     6750
## ExpDay_factor8.units       0.6011   0.5369   0.6728     6750
## ExpDay_factor9.units       0.8229   0.7276   0.9090     6750
## ExpDay_factor10.units      0.6567   0.5832   0.7289     6750
## ExpDay_factor11.units      0.6494   0.5752   0.7192     6750
## ExpDay_factor12.units      0.8380   0.7462   0.9299     6380
## ExpDay_factor13.units      0.6929   0.6149   0.7686     6750
## ExpDay_factor14.units      0.5858   0.5231   0.6501     6750
## ExpDay_factor15.units      0.6745   0.5981   0.7494     6397
## ExpDay_factor16.units      0.8733   0.7845   0.9784     6750
## ExpDay_factor17.units      0.7005   0.6202   0.7721     6511
## ExpDay_factor18.units      0.6715   0.5950   0.7441     6750
## ExpDay_factor19.units      0.5837   0.5214   0.6503     6750
## ExpDay_factor20.units      0.6417   0.5689   0.7126     6750
## ExpDay_factor21.units      0.6829   0.6091   0.7572     6432
## ExpDay_factor22.units      0.6152   0.5488   0.6857     6750
## ExpDay_factor23.units      0.5140   0.4570   0.5719     6750
## ExpDay_factor24.units      0.5835   0.5197   0.6473     6750
## ExpDay_factor25.units      0.6109   0.5475   0.6802     6750
## ExpDay_factor26.units      0.7445   0.6646   0.8327     6750
## ExpDay_factor27.units      0.6520   0.5820   0.7296     6750
##
##  Location effects: MeanCorner_hourly_scaled ~ ExpDay + Hour
##
##             post.mean  l-95% CI  u-95% CI eff.samp  pMCMC
## (Intercept)  0.253118  0.071033  0.439169     6407 0.0077 **
```

```
## ExpDay      -0.001123 -0.008827  0.006579     6750 0.7647
## Hour        -0.015077 -0.018850 -0.011402     6750 <1e-04 ***
## ---
## Signif. codes:  0 '***' 0.001 '**' 0.01 '*' 0.05 '.' 0.1 ' ' 1
```

```
summary(model_control$Sol)
```

```
##
## Iterations = 150001:1499801
## Thinning interval = 200
## Number of chains = 1
## Sample size per chain = 6750
##
## 1. Empirical mean and standard deviation for each variable,
##    plus standard error of the mean:
##
##                  Mean       SD  Naive SE Time-series SE
## (Intercept)  0.002524 0.087102 1.060e-03      1.060e-03
## ExpDay      -0.006074 0.003458 4.208e-05      4.208e-05
## Hour        -0.016380 0.001714 2.086e-05      2.086e-05
##
## 2. Quantiles for each variable:
##
##                 2.5%       25%       50%      75%      97.5%
## (Intercept) -0.17084 -0.053122  0.002228  0.05726  0.1780650
## ExpDay      -0.01303 -0.008377 -0.006041 -0.00376  0.0006416
## Hour        -0.01974 -0.017538 -0.016378 -0.01523 -0.0130484
```

```
summary(model_predator$Sol)
```

```
##
## Iterations = 150001:1499801
## Thinning interval = 200
## Number of chains = 1
## Sample size per chain = 6750
##
## 1. Empirical mean and standard deviation for each variable,
##    plus standard error of the mean:
##
##                  Mean       SD  Naive SE Time-series SE
## (Intercept)  0.253118 0.093782 1.141e-03      1.172e-03
## ExpDay      -0.001123 0.003952 4.811e-05      4.811e-05
## Hour        -0.015077 0.001909 2.323e-05      2.323e-05
##
## 2. Quantiles for each variable:
##
##                 2.5%       25%      50%       75%     97.5%
## (Intercept)  0.07163  0.190827  0.25145  0.311407  0.441889
## ExpDay      -0.00879 -0.003734 -0.00113  0.001478  0.006652
## Hour        -0.01884 -0.016340 -0.01508 -0.013763 -0.011387
```

```
summary(model_control$VCV)
```

```
##
## Iterations = 150001:1499801
## Thinning interval = 200
## Number of chains = 1
## Sample size per chain = 6750
##
## 1. Empirical mean and standard deviation for each variable,
##    plus standard error of the mean:
##
##                                      Mean        SD  Naive SE Time-series SE
```

```
## (Intercept):(Intercept).FishId  0.2752095 0.0608984 7.412e-04      8.167e-04
## ExpDay:(Intercept).FishId      -0.0048906 0.0019950 2.428e-05      2.428e-05
## (Intercept):ExpDay.FishId      -0.0048906 0.0019950 2.428e-05      2.428e-05
## ExpDay:ExpDay.FishId            0.0005439 0.0001226 1.492e-06      1.602e-06
## MomId                           0.0229431 0.0330616 4.024e-04      5.378e-04
## ExpDay_factor0.units            1.2358548 0.0733765 8.931e-04      8.931e-04
## ExpDay_factor1.units            0.7798947 0.0447463 5.446e-04      5.449e-04
## ExpDay_factor2.units            0.8614361 0.0500230 6.089e-04      6.089e-04
## ExpDay_factor3.units            0.8159421 0.0492220 5.991e-04      5.991e-04
## ExpDay_factor4.units            0.7431493 0.0433654 5.278e-04      5.492e-04
## ExpDay_factor5.units            0.4146712 0.0241173 2.935e-04      2.935e-04
## ExpDay_factor6.units            0.4100896 0.0253983 3.091e-04      3.091e-04
## ExpDay_factor7.units            0.6161276 0.0355222 4.324e-04      4.324e-04
## ExpDay_factor8.units            0.4327796 0.0252766 3.077e-04      3.077e-04
## ExpDay_factor9.units            0.5713562 0.0333633 4.061e-04      4.061e-04
## ExpDay_factor10.units           0.6709354 0.0425607 5.180e-04      5.180e-04
## ExpDay_factor11.units           0.4974369 0.0304599 3.707e-04      3.656e-04
## ExpDay_factor12.units           0.4875683 0.0292329 3.558e-04      3.558e-04
## ExpDay_factor13.units           0.4867065 0.0284731 3.466e-04      3.466e-04
## ExpDay_factor14.units           0.4781194 0.0273708 3.331e-04      3.331e-04
## ExpDay_factor15.units           0.6258749 0.0366341 4.459e-04      4.459e-04
## ExpDay_factor16.units           0.5737243 0.0333606 4.061e-04      4.061e-04
## ExpDay_factor17.units           0.5390785 0.0311930 3.797e-04      3.797e-04
## ExpDay_factor18.units           0.5073486 0.0305742 3.721e-04      3.721e-04
## ExpDay_factor19.units           0.5271524 0.0315297 3.838e-04      3.838e-04
## ExpDay_factor20.units           0.5948167 0.0357078 4.346e-04      4.371e-04
## ExpDay_factor21.units           0.5818668 0.0342010 4.163e-04      4.163e-04
## ExpDay_factor22.units           0.5143016 0.0301258 3.667e-04      3.602e-04
## ExpDay_factor23.units           0.4327134 0.0265812 3.235e-04      3.426e-04
## ExpDay_factor24.units           0.4902908 0.0292136 3.556e-04      3.556e-04
## ExpDay_factor25.units           0.3332952 0.0206918 2.519e-04      2.519e-04
## ExpDay_factor26.units           0.4692723 0.0284853 3.467e-04      3.467e-04
## ExpDay_factor27.units           0.4655188 0.0288124 3.507e-04      3.507e-04
##
## 2. Quantiles for each variable:
##
##                                      2.5%        25%        50%        75%
## (Intercept):(Intercept).FishId  1.788e-01  0.2322010  0.2682329  0.3085555
## ExpDay:(Intercept).FishId      -9.250e-03 -0.0060827 -0.0047451 -0.0034896
## (Intercept):ExpDay.FishId      -9.250e-03 -0.0060827 -0.0047451 -0.0034896
## ExpDay:ExpDay.FishId            3.548e-04  0.0004549  0.0005269  0.0006139
## MomId                           3.196e-05  0.0028696  0.0113682  0.0297547
## ExpDay_factor0.units            1.100e+00  1.1848062  1.2329502  1.2840313
## ExpDay_factor1.units            6.970e-01  0.7489967  0.7783807  0.8084450
## ExpDay_factor2.units            7.682e-01  0.8260951  0.8600956  0.8937331
## ExpDay_factor3.units            7.265e-01  0.7820729  0.8138336  0.8474360
## ExpDay_factor4.units            6.614e-01  0.7137542  0.7418133  0.7707775
## ExpDay_factor5.units            3.698e-01  0.3978334  0.4136593  0.4304041
## ExpDay_factor6.units            3.630e-01  0.3926279  0.4091068  0.4262887
## ExpDay_factor7.units            5.506e-01  0.5915011  0.6143629  0.6389990
## ExpDay_factor8.units            3.863e-01  0.4155786  0.4317884  0.4496312
## ExpDay_factor9.units            5.094e-01  0.5478384  0.5702823  0.5931587
## ExpDay_factor10.units           5.922e-01  0.6411835  0.6694633  0.6983516
## ExpDay_factor11.units           4.419e-01  0.4763353  0.4958849  0.5169646
## ExpDay_factor12.units           4.340e-01  0.4675186  0.4862201  0.5064711
## ExpDay_factor13.units           4.344e-01  0.4670305  0.4855823  0.5055197
## ExpDay_factor14.units           4.279e-01  0.4589597  0.4775282  0.4958818
## ExpDay_factor15.units           5.580e-01  0.5999542  0.6249165  0.6500109
## ExpDay_factor16.units           5.123e-01  0.5503078  0.5724968  0.5959885
## ExpDay_factor17.units           4.811e-01  0.5170670  0.5377838  0.5588688
## ExpDay_factor18.units           4.510e-01  0.4858193  0.5063169  0.5270047
```

```
## ExpDay_factor19.units           4.689e-01  0.5048634  0.5263299  0.5478618
## ExpDay_factor20.units           5.290e-01  0.5703565  0.5934024  0.6181620
## ExpDay_factor21.units           5.196e-01  0.5582946  0.5805846  0.6041440
## ExpDay_factor22.units           4.575e-01  0.4934537  0.5134198  0.5339932
## ExpDay_factor23.units           3.834e-01  0.4138554  0.4321051  0.4499112
## ExpDay_factor24.units           4.360e-01  0.4702013  0.4888946  0.5090983
## ExpDay_factor25.units           2.952e-01  0.3187893  0.3325017  0.3469078
## ExpDay_factor26.units           4.166e-01  0.4497072  0.4678642  0.4880942
## ExpDay_factor27.units           4.133e-01  0.4454260  0.4640827  0.4840665
##                                     97.5%
## (Intercept):(Intercept).FishId  0.4152538
## ExpDay:(Intercept).FishId      -0.0014121
## (Intercept):ExpDay.FishId      -0.0014121
## ExpDay:ExpDay.FishId            0.0008253
## MomId                           0.1148727
## ExpDay_factor0.units            1.3871134
## ExpDay_factor1.units            0.8730008
## ExpDay_factor2.units            0.9660573
## ExpDay_factor3.units            0.9209807
## ExpDay_factor4.units            0.8333242
## ExpDay_factor5.units            0.4641969
## ExpDay_factor6.units            0.4636163
## ExpDay_factor7.units            0.6912030
## ExpDay_factor8.units            0.4840070
## ExpDay_factor9.units            0.6394601
## ExpDay_factor10.units           0.7591770
## ExpDay_factor11.units           0.5611171
## ExpDay_factor12.units           0.5492881
## ExpDay_factor13.units           0.5449180
## ExpDay_factor14.units           0.5346763
## ExpDay_factor15.units           0.7005630
## ExpDay_factor16.units           0.6412559
## ExpDay_factor17.units           0.6036127
## ExpDay_factor18.units           0.5715477
## ExpDay_factor19.units           0.5927006
## ExpDay_factor20.units           0.6667799
## ExpDay_factor21.units           0.6535676
## ExpDay_factor22.units           0.5768581
## ExpDay_factor23.units           0.4868987
## ExpDay_factor24.units           0.5493503
## ExpDay_factor25.units           0.3755613
## ExpDay_factor26.units           0.5280794
## ExpDay_factor27.units           0.5249979
```

```
summary(model_predator$VCV)
```

```
##
## Iterations = 150001:1499801
## Thinning interval = 200
## Number of chains = 1
## Sample size per chain = 6750
##
## 1. Empirical mean and standard deviation for each variable,
##    plus standard error of the mean:
##
##                                      Mean        SD  Naive SE Time-series SE
## (Intercept):(Intercept).FishId  0.2773437 0.0621340 7.563e-04      8.140e-04
## ExpDay:(Intercept).FishId      -0.0062128 0.0023886 2.907e-05      2.946e-05
## (Intercept):ExpDay.FishId      -0.0062128 0.0023886 2.907e-05      2.946e-05
## ExpDay:ExpDay.FishId            0.0007491 0.0001641 1.998e-06      2.161e-06
## MomId                           0.0363356 0.0467167 5.686e-04      7.958e-04
## ExpDay_factor0.units            1.4174065 0.0803915 9.785e-04      9.785e-04
```

```
## ExpDay_factor1.units            1.2715455 0.0705295 8.585e-04      8.268e-04
## ExpDay_factor2.units            1.4091322 0.0787607 9.586e-04      9.586e-04
## ExpDay_factor3.units            1.2660684 0.0710892 8.653e-04      8.653e-04
## ExpDay_factor4.units            1.2467551 0.0710420 8.647e-04      8.851e-04
## ExpDay_factor5.units            0.8312209 0.0476463 5.799e-04      5.799e-04
## ExpDay_factor6.units            0.8922208 0.0505084 6.148e-04      6.148e-04
## ExpDay_factor7.units            0.7508478 0.0419684 5.108e-04      5.108e-04
## ExpDay_factor8.units            0.6010911 0.0345443 4.205e-04      4.205e-04
## ExpDay_factor9.units            0.8229283 0.0472194 5.747e-04      5.747e-04
## ExpDay_factor10.units           0.6566746 0.0373524 4.546e-04      4.546e-04
## ExpDay_factor11.units           0.6493823 0.0374578 4.559e-04      4.559e-04
## ExpDay_factor12.units           0.8379917 0.0472910 5.756e-04      5.921e-04
## ExpDay_factor13.units           0.6928999 0.0387433 4.716e-04      4.716e-04
## ExpDay_factor14.units           0.5857729 0.0329279 4.008e-04      4.008e-04
## ExpDay_factor15.units           0.6744976 0.0388185 4.725e-04      4.854e-04
## ExpDay_factor16.units           0.8732996 0.0497836 6.059e-04      6.059e-04
## ExpDay_factor17.units           0.7004983 0.0392668 4.779e-04      4.866e-04
## ExpDay_factor18.units           0.6714662 0.0381597 4.645e-04      4.645e-04
## ExpDay_factor19.units           0.5836907 0.0330694 4.025e-04      4.025e-04
## ExpDay_factor20.units           0.6417489 0.0365737 4.452e-04      4.452e-04
## ExpDay_factor21.units           0.6829108 0.0382925 4.661e-04      4.775e-04
## ExpDay_factor22.units           0.6152230 0.0349335 4.252e-04      4.252e-04
## ExpDay_factor23.units           0.5139705 0.0291232 3.545e-04      3.545e-04
## ExpDay_factor24.units           0.5835276 0.0331096 4.030e-04      4.030e-04
## ExpDay_factor25.units           0.6109000 0.0341333 4.155e-04      4.155e-04
## ExpDay_factor26.units           0.7445479 0.0425823 5.183e-04      5.183e-04
## ExpDay_factor27.units           0.6519950 0.0381616 4.645e-04      4.645e-04
##
## 2. Quantiles for each variable:
##
##                                      2.5%        25%        50%       75%
## (Intercept):(Intercept).FishId  1.774e-01  0.2329696  0.2697409  0.312444
## ExpDay:(Intercept).FishId      -1.164e-02 -0.0076353 -0.0059963 -0.004549
## (Intercept):ExpDay.FishId      -1.164e-02 -0.0076353 -0.0059963 -0.004549
## ExpDay:ExpDay.FishId            4.937e-04  0.0006321  0.0007272  0.000841
## MomId                           6.746e-05  0.0063038  0.0217191  0.048989
## ExpDay_factor0.units            1.267e+00  1.3626618  1.4140951  1.469829
## ExpDay_factor1.units            1.143e+00  1.2229472  1.2686942  1.317716
## ExpDay_factor2.units            1.264e+00  1.3538394  1.4063509  1.460113
## ExpDay_factor3.units            1.135e+00  1.2177703  1.2622648  1.311517
## ExpDay_factor4.units            1.117e+00  1.1966720  1.2442012  1.292861
## ExpDay_factor5.units            7.432e-01  0.7987881  0.8294923  0.861819
## ExpDay_factor6.units            7.965e-01  0.8577961  0.8910595  0.924698
## ExpDay_factor7.units            6.735e-01  0.7218713  0.7494427  0.778386
## ExpDay_factor8.units            5.373e-01  0.5772722  0.5997254  0.623583
## ExpDay_factor9.units            7.351e-01  0.7905078  0.8215432  0.855059
## ExpDay_factor10.units           5.873e-01  0.6309297  0.6550686  0.680510
## ExpDay_factor11.units           5.804e-01  0.6229382  0.6477811  0.673817
## ExpDay_factor12.units           7.495e-01  0.8065345  0.8357581  0.868547
## ExpDay_factor13.units           6.200e-01  0.6666818  0.6911960  0.717619
## ExpDay_factor14.units           5.256e-01  0.5629309  0.5844907  0.607287
## ExpDay_factor15.units           6.021e-01  0.6471013  0.6730002  0.700364
## ExpDay_factor16.units           7.812e-01  0.8387775  0.8716783  0.905575
## ExpDay_factor17.units           6.277e-01  0.6729692  0.6998557  0.726441
## ExpDay_factor18.units           6.005e-01  0.6448307  0.6699580  0.695990
## ExpDay_factor19.units           5.227e-01  0.5604428  0.5820103  0.605143
## ExpDay_factor20.units           5.731e-01  0.6165058  0.6403045  0.664871
## ExpDay_factor21.units           6.123e-01  0.6561275  0.6812164  0.708271
## ExpDay_factor22.units           5.504e-01  0.5910729  0.6139181  0.638240
## ExpDay_factor23.units           4.591e-01  0.4941043  0.5126366  0.532472
## ExpDay_factor24.units           5.236e-01  0.5604246  0.5827297  0.604824
```

```
## ExpDay_factor25.units          5.482e-01  0.5870590  0.6094768  0.633604
## ExpDay_factor26.units          6.649e-01  0.7147244  0.7430590  0.771912
## ExpDay_factor27.units          5.812e-01  0.6253693  0.6501019  0.676952
##                                   97.5%
## (Intercept):(Intercept).FishId  0.420562
## ExpDay:(Intercept).FishId      -0.002140
## (Intercept):ExpDay.FishId      -0.002140
## ExpDay:ExpDay.FishId            0.001123
## MomId                           0.157441
## ExpDay_factor0.units            1.583157
## ExpDay_factor1.units            1.417832
## ExpDay_factor2.units            1.571862
## ExpDay_factor3.units            1.414912
## ExpDay_factor4.units            1.395314
## ExpDay_factor5.units            0.928002
## ExpDay_factor6.units            0.996254
## ExpDay_factor7.units            0.837620
## ExpDay_factor8.units            0.673591
## ExpDay_factor9.units            0.917467
## ExpDay_factor10.units           0.734576
## ExpDay_factor11.units           0.726076
## ExpDay_factor12.units           0.934845
## ExpDay_factor13.units           0.774462
## ExpDay_factor14.units           0.653682
## ExpDay_factor15.units           0.753946
## ExpDay_factor16.units           0.976431
## ExpDay_factor17.units           0.780505
## ExpDay_factor18.units           0.750117
## ExpDay_factor19.units           0.652866
## ExpDay_factor20.units           0.716952
## ExpDay_factor21.units           0.762516
## ExpDay_factor22.units           0.687796
## ExpDay_factor23.units           0.574747
## ExpDay_factor24.units           0.652636
## ExpDay_factor25.units           0.681118
## ExpDay_factor26.units           0.833305
## ExpDay_factor27.units           0.729384
```

Extracting random slope variance components over time

```
# Control

sigma.a0 <- model_control$VCV[, "(Intercept):(Intercept).FishId"]
sigma.a1 <- model_control$VCV[, "ExpDay:ExpDay.FishId"]
cov <- model_control$VCV[, "(Intercept):ExpDay.FishId"]
mom_intercept <- model_control$VCV[, "MomId"]

# Loop to extract variance components per day
for (day in 0:27) {
    x_i <- day
    sigma.e <- model_control$VCV[, paste0("ExpDay_factor", day,
        ".units")]
    rep <- (sigma.a0 + sigma.a1 * x_i^2 + 2 * cov * x_i)/(sigma.a0 +
        sigma.a1 * x_i^2 + 2 * cov * x_i + sigma.e)
    rep_control <- median(rep)
    rep_control_CI <- HPDinterval(as.mcmc(rep))
    rep_with_mom <- (sigma.a0 + sigma.a1 * x_i^2 + 2 * cov *
        x_i + mom_intercept)/(sigma.a0 + sigma.a1 * x_i^2 + 2 *
        cov * x_i + sigma.e + +mom_intercept)
    rep_with_mom_control <- median(rep_with_mom)
```

```
    rep_with_mom_control_CI <- HPDinterval(as.mcmc(rep_with_mom))
    among_val <- sigma.a0 + (sigma.a1 * x_i^2 + 2 * cov * x_i)
    among_control <- median(among_val)
    among_control_CI <- HPDinterval(as.mcmc(among_val))
    within_control <- median(sigma.e)
    within_control_CI <- HPDinterval(as.mcmc(sigma.e))
    assign(paste0("rep_control_day", day), rep_control)
    assign(paste0("rep_control_CI_day", day), rep_control_CI)
    assign(paste0("rep_with_mom_control_day", day), rep_with_mom_control)
    assign(paste0("rep_with_mom_control_CI_day", day), rep_with_mom_control_CI)
    assign(paste0("among_control_day", day), among_control)
    assign(paste0("among_control_CI_day", day), among_control_CI)
    assign(paste0("within_control_day", day), within_control)
    assign(paste0("within_control_CI_day", day), within_control_CI)
}

# Predator

sigma.a0 <- model_predator$VCV[, "(Intercept):(Intercept).FishId"]
sigma.a1 <- model_predator$VCV[, "ExpDay:ExpDay.FishId"]
cov <- model_predator$VCV[, "(Intercept):ExpDay.FishId"]
mom_intercept <- model_predator$VCV[, "MomId"]

# Loop to extract variance components per day
for (day in 0:27) {
    x_i <- day
    sigma.e <- model_predator$VCV[, paste0("ExpDay_factor", day,
        ".units")]
    rep <- (sigma.a0 + sigma.a1 * x_i^2 + 2 * cov * x_i)/(sigma.a0 +
        sigma.a1 * x_i^2 + 2 * cov * x_i + sigma.e)
    rep_predator <- median(rep)
    rep_predator_CI <- HPDinterval(as.mcmc(rep))
    rep_with_mom <- (sigma.a0 + sigma.a1 * x_i^2 + 2 * cov *
        x_i + mom_intercept)/(sigma.a0 + sigma.a1 * x_i^2 + 2 *
        cov * x_i + sigma.e + +mom_intercept)
    rep_with_mom_predator <- median(rep_with_mom)
    rep_with_mom_predator_CI <- HPDinterval(as.mcmc(rep_with_mom))
    among_val <- sigma.a0 + (sigma.a1 * x_i^2 + 2 * cov * x_i)
    among_predator <- median(among_val)
    among_predator_CI <- HPDinterval(as.mcmc(among_val))
    within_predator <- median(sigma.e)
    within_predator_CI <- HPDinterval(as.mcmc(sigma.e))
    assign(paste0("rep_predator_day", day), rep_predator)
    assign(paste0("rep_predator_CI_day", day), rep_predator_CI)
    assign(paste0("rep_with_mom_predator_day", day), rep_with_mom_predator)
    assign(paste0("rep_with_mom_predator_CI_day", day), rep_with_mom_predator_CI)
    assign(paste0("among_predator_day", day), among_predator)
    assign(paste0("among_predator_CI_day", day), among_predator_CI)
    assign(paste0("within_predator_day", day), within_predator)
    assign(paste0("within_predator_CI_day", day), within_predator_CI)
}

### Plots

## Repeatability - no mom

# Define colors for plotting:
colors <- c(Control = "#1709b5", Predator = "#ed5715")

# Make blank dataframe
variance_components_table <- data.frame(Day = integer(), Repeatability = numeric(),
```

```r
    RepeatabilityLowerCI = numeric(), RepeatabilityUpperCI = numeric(),
    Treatment = character(), stringsAsFactors = FALSE)

# Loop from day 0 through 27
for (day in 0:27) {
    # Control
    rep_ctrl_name <- paste0("rep_control_day", day)
    rep_ctrl_ci_name <- paste0("rep_control_CI_day", day)
    rep_ctrl_val <- get(rep_ctrl_name)
    rep_ctrl_CI <- get(rep_ctrl_ci_name)
    row_ctrl <- data.frame(Day = day, Repeatability = rep_ctrl_val,
        RepeatabilityLowerCI = rep_ctrl_CI[1], RepeatabilityUpperCI = rep_ctrl_CI[2],
        Treatment = "Control")
    variance_components_table <- rbind(variance_components_table,
        row_ctrl)

    # Pred
    rep_pred_name <- paste0("rep_predator_day", day)
    rep_pred_ci_name <- paste0("rep_predator_CI_day", day)
    rep_pred_val <- get(rep_pred_name)
    rep_pred_CI <- get(rep_pred_ci_name)
    row_pred <- data.frame(Day = day, Repeatability = rep_pred_val,
        RepeatabilityLowerCI = rep_pred_CI[1], RepeatabilityUpperCI = rep_pred_CI[2],
        Treatment = "Predator")
    variance_components_table <- rbind(variance_components_table,
        row_pred)
}

plot_name <- ggplot(variance_components_table, aes(x = Day +
    1, y = Repeatability, color = Treatment)) + geom_line(show.legend = FALSE) +
    geom_point(size = 2, show.legend = FALSE) + geom_errorbar(aes(ymin = RepeatabilityLowerCI,
    ymax = RepeatabilityUpperCI), width = 0.5, show.legend = FALSE) +
    scale_x_continuous(limits = c(0.5, 28.5), breaks = seq(1,
        29, by = 3)) + scale_y_continuous(limits = c(0, 0.7),
    breaks = seq(0, 0.7, by = 0.1)) + scale_color_manual(values = colors) +
    labs(x = "Day", y = "Repeatability") + theme_classic()
plot_name
```

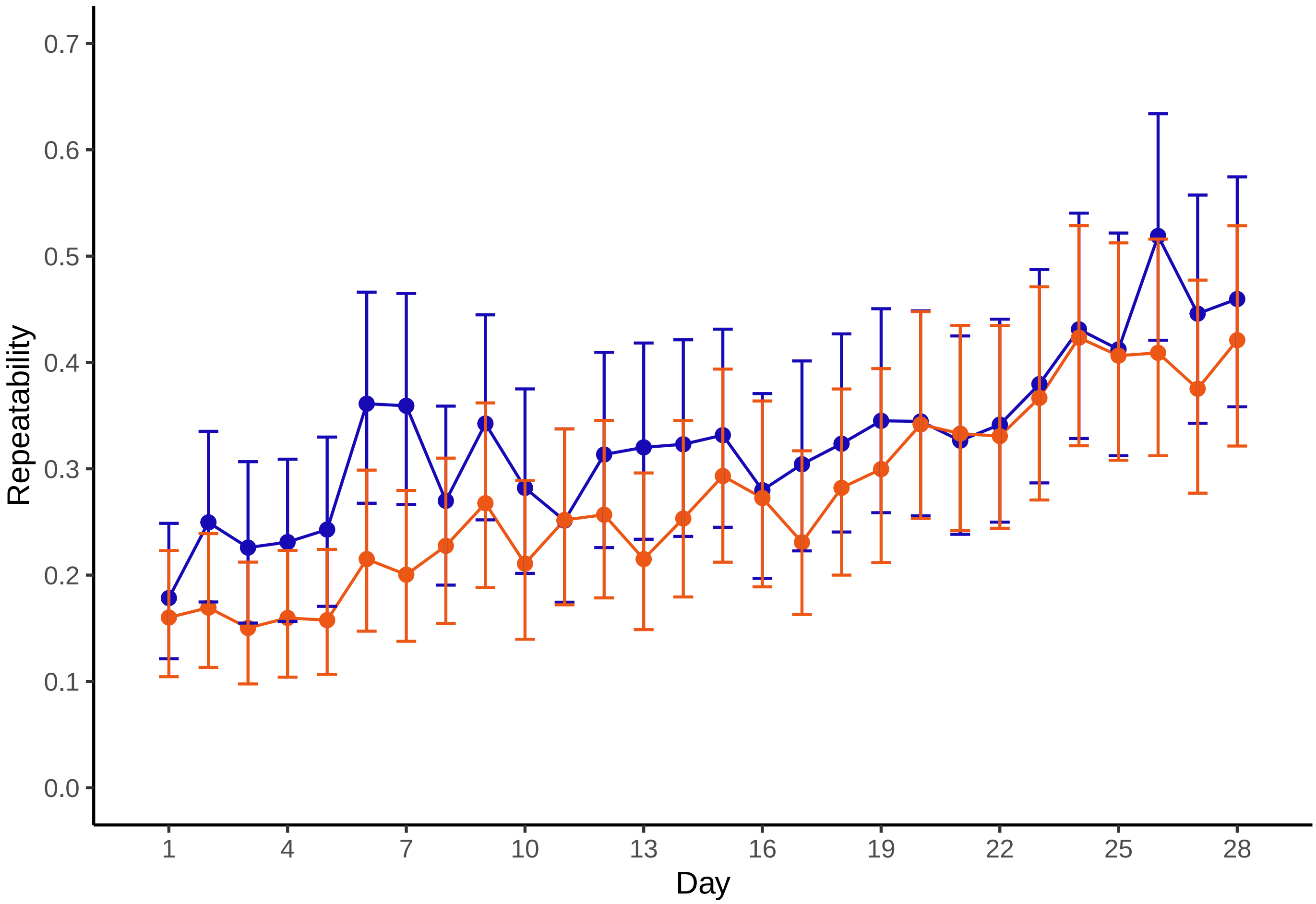


```
ggsave("repeatability_refuge_use_hour.pdf", plot = plot_name,
    dpi = 600)
```

```
## Saving 6.5 x 4.5 in image
```

```
## Repeatability - WITH mom

# Define colors for plotting:
colors <- c(Control = "#1709b5", Predator = "#ed5715")

variance_components_table <- data.frame(Day = integer(), Repeatability = numeric(),
    RepeatabilityLowerCI = numeric(), RepeatabilityUpperCI = numeric(),
    Treatment = character(), stringsAsFactors = FALSE)

# Loop from day 0 through 27
for (day in 0:27) {
    # Control
    rep_ctrl_name <- paste0("rep_with_mom_control_day", day)
    rep_ctrl_ci_name <- paste0("rep_with_mom_control_CI_day",
        day)
    rep_ctrl_val <- get(rep_ctrl_name)
    rep_ctrl_CI <- get(rep_ctrl_ci_name)
    row_ctrl <- data.frame(Day = day, Repeatability = rep_ctrl_val,
        RepeatabilityLowerCI = rep_ctrl_CI[1], RepeatabilityUpperCI = rep_ctrl_CI[2],
        Treatment = "Control")
    variance_components_table <- rbind(variance_components_table,
        row_ctrl)

    # Pred
```

```
    rep_pred_name <- paste0("rep_with_mom_predator_day", day)
    rep_pred_ci_name <- paste0("rep_with_mom_predator_CI_day",
        day)
    rep_pred_val <- get(rep_pred_name)
    rep_pred_CI <- get(rep_pred_ci_name)
    row_pred <- data.frame(Day = day, Repeatability = rep_pred_val,
        RepeatabilityLowerCI = rep_pred_CI[1], RepeatabilityUpperCI = rep_pred_CI[2],
        Treatment = "Predator")
    variance_components_table <- rbind(variance_components_table,
        row_pred)
}

plot_name <- ggplot(variance_components_table, aes(x = Day +
    1, y = Repeatability, color = Treatment)) + geom_line(show.legend = FALSE) +
    geom_point(size = 2, show.legend = FALSE) + geom_errorbar(aes(ymin = RepeatabilityLowerCI,
    ymax = RepeatabilityUpperCI), width = 0.5, show.legend = FALSE) +
    scale_x_continuous(limits = c(0.5, 28.5), breaks = seq(1,
        29, by = 3)) + scale_y_continuous(limits = c(0, 0.7),
    breaks = seq(0, 0.7, by = 0.1)) + scale_color_manual(values = colors) +
    labs(x = "Day", y = "Repeatability") + theme_classic()
plot_name
```

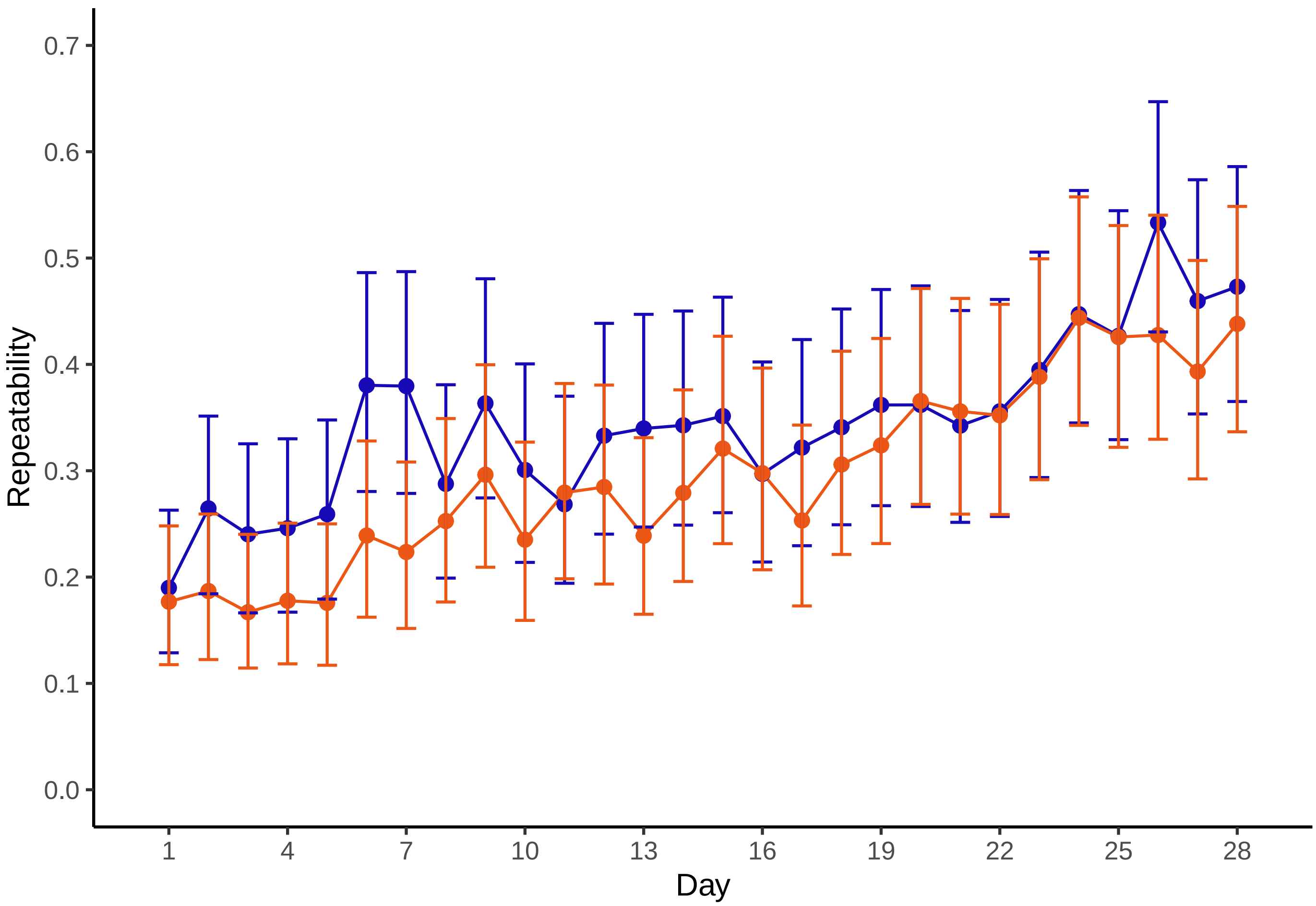


```
ggsave("repeatability_with_mom_refuge_use_hour.pdf", plot = plot_name,
    dpi = 600)
```

```
## Saving 6.5 x 4.5 in image
```

```
## Among-individual variance
```

```r
# Define colors for plotting:
colors <- c(Control = "#1709b5", Predator = "#ed5715")

variance_components_table <- data.frame(Day = integer(), Among = numeric(),
    AmongLowerCI = numeric(), AmongUpperCI = numeric(), Treatment = character(),
    stringsAsFactors = FALSE)

# Loop from day 0 through 27
for (day in 0:27) {
    # Control
    among_ctrl_name <- paste0("among_control_day", day)
    among_ctrl_ci_name <- paste0("among_control_CI_day", day)
    among_ctrl_val <- get(among_ctrl_name)
    among_ctrl_CI <- get(among_ctrl_ci_name)
    row_ctrl <- data.frame(Day = day, Among = among_ctrl_val,
        AmongLowerCI = among_ctrl_CI[1], AmongUpperCI = among_ctrl_CI[2],
        Treatment = "Control")
    variance_components_table <- rbind(variance_components_table,
        row_ctrl)

    # Pred
    among_pred_name <- paste0("among_predator_day", day)
    among_pred_ci_name <- paste0("among_predator_CI_day", day)
    among_pred_val <- get(among_pred_name)
    among_pred_CI <- get(among_pred_ci_name)
    row_pred <- data.frame(Day = day, Among = among_pred_val,
        AmongLowerCI = among_pred_CI[1], AmongUpperCI = among_pred_CI[2],
        Treatment = "Predator")
    variance_components_table <- rbind(variance_components_table,
        row_pred)
}

plot_name <- ggplot(variance_components_table, aes(x = Day +
    1, y = Among, color = Treatment)) + geom_line(show.legend = FALSE) +
    geom_errorbar(aes(ymin = AmongLowerCI, ymax = AmongUpperCI),
        width = 0.5, show.legend = FALSE) + geom_point(size = 2.5,
    shape = 24, fill = "white", show.legend = FALSE) + scale_x_continuous(limits = c(0.5,
    28.5), breaks = seq(1, 29, by = 3)) + scale_y_continuous(limits = c(0,
    1.6), breaks = seq(0, 1.6, by = 0.4)) + scale_color_manual(values = colors) +
    labs(x = "Day", y = "Among-individual variance") + theme_classic()
plot_name
```

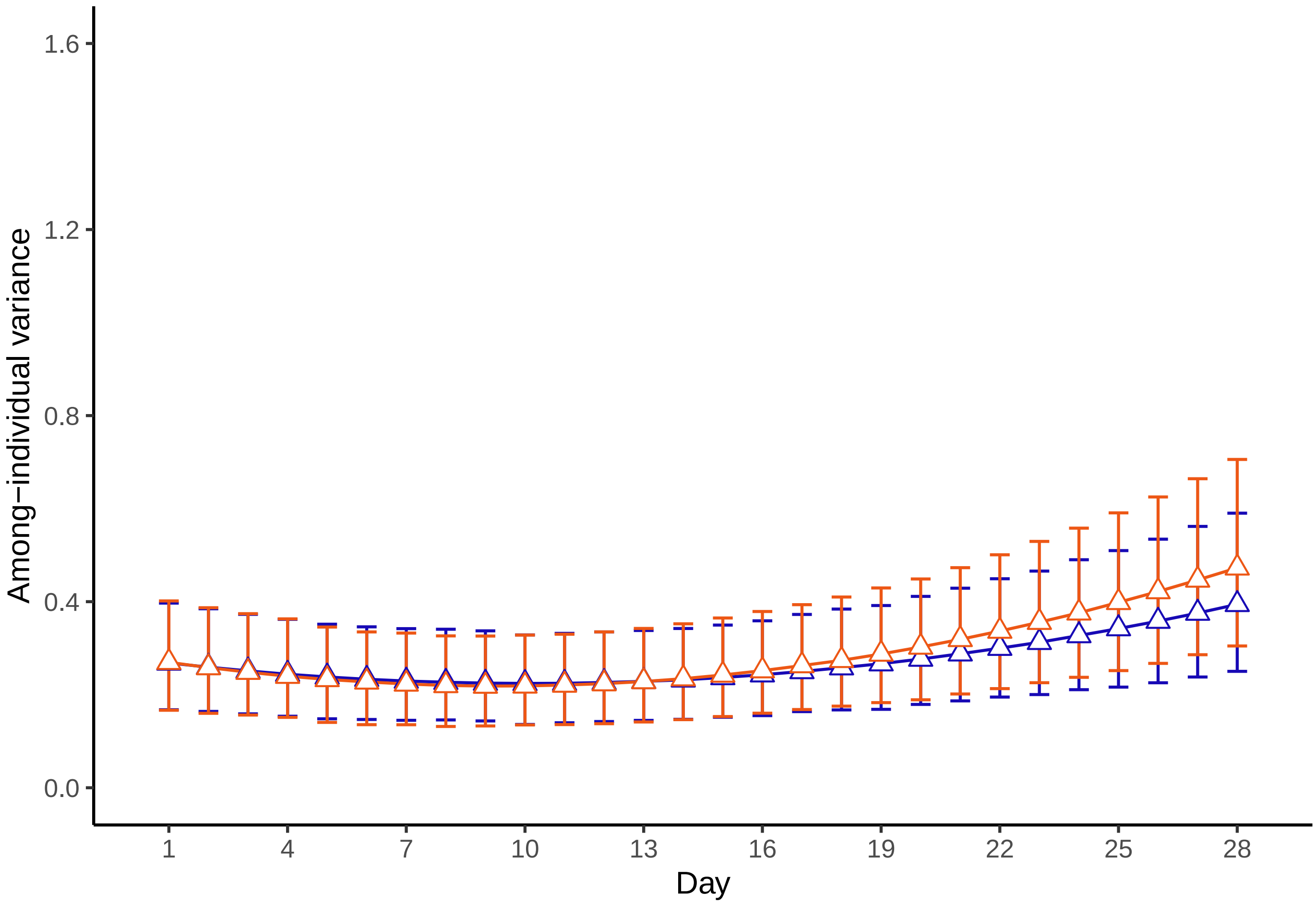


```
ggsave("among_refuge_use_hour.pdf", plot = plot_name, dpi = 600)
```

```
## Saving 6.5 x 4.5 in image
```

```
## Within-individual variance

# Define colors for plotting:
colors <- c(Control = "#1709b5", Predator = "#ed5715")

variance_components_table <- data.frame(Day = integer(), Within = numeric(),
    WithinLowerCI = numeric(), WithinUpperCI = numeric(), Treatment = character(),
    stringsAsFactors = FALSE)

# Loop from day 0 through 27
for (day in 0:27) {
    # Control
    within_ctrl_name <- paste0("within_control_day", day)
    within_ctrl_ci_name <- paste0("within_control_CI_day", day)
    within_ctrl_val <- get(within_ctrl_name)
    within_ctrl_CI <- get(within_ctrl_ci_name)
    row_ctrl <- data.frame(Day = day, Within = within_ctrl_val,
        WithinLowerCI = within_ctrl_CI[1], WithinUpperCI = within_ctrl_CI[2],
        Treatment = "Control")
    variance_components_table <- rbind(variance_components_table,
        row_ctrl)

    # Pred
    within_ctrl_name <- paste0("within_predator_day", day)
    within_ctrl_ci_name <- paste0("within_predator_CI_day", day)
```

```
    within_ctrl_val <- get(within_ctrl_name)
    within_ctrl_CI <- get(within_ctrl_ci_name)
    row_ctrl <- data.frame(Day = day, Within = within_ctrl_val,
        WithinLowerCI = within_ctrl_CI[1], WithinUpperCI = within_ctrl_CI[2],
        Treatment = "Predator")
    variance_components_table <- rbind(variance_components_table,
        row_ctrl)
}

plot_name <- ggplot(variance_components_table, aes(x = Day +
    1, y = Within, color = Treatment)) + geom_line(show.legend = FALSE) +
    geom_point(size = 2, show.legend = FALSE) + geom_errorbar(aes(ymin = WithinLowerCI,
    ymax = WithinUpperCI), width = 0.5, show.legend = FALSE) +
    scale_x_continuous(limits = c(0.5, 28.5), breaks = seq(1,
        29, by = 3)) + scale_y_continuous(limits = c(0, 1.6),
    breaks = seq(0, 1.6, by = 0.4)) + scale_color_manual(values = colors) +
    labs(x = "Day", y = "Within-individual variance") + theme_classic()
plot_name
```

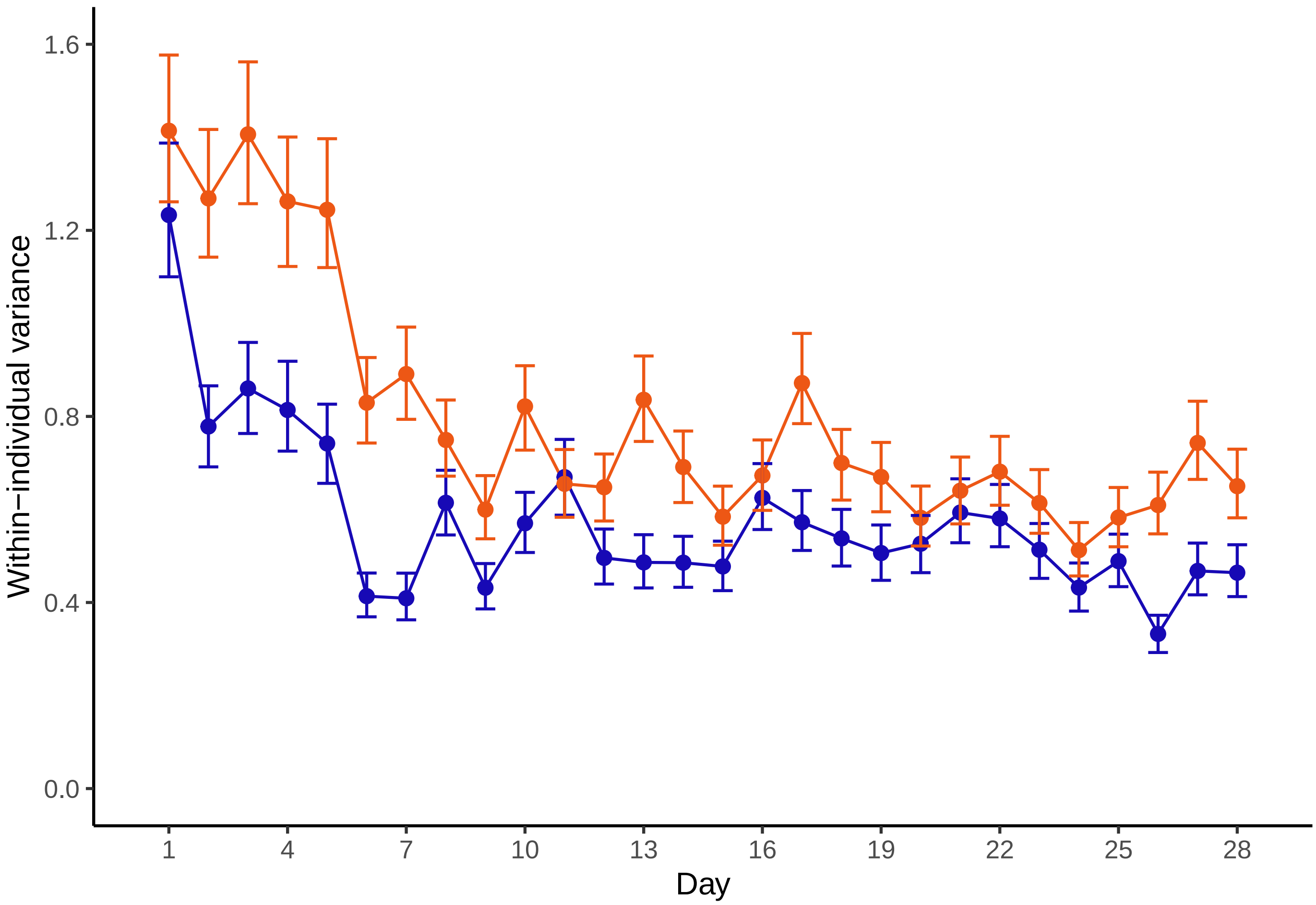


```
ggsave("within_refuge_use_hour.pdf", plot = plot_name, dpi = 600)
```

```
## Saving 6.5 x 4.5 in image
```

Variance components do not appear to change over time in either treatment.
Among-individual differences are stronger in control fish, but not significantly, as there is overlap in confidence intervals.

Within-individual variation is significantly greater in predator fish than control fish.

Relationship between MomId + treatment and body size

```r
# Subset data to include one body size data point per
# individual
bodysize_per_fish_data <- subset(bodysize_only_data, ExpDay ==
    0)

# Length
model_mom_on_bodysize_length_mom_YearBatch <- readRDS("model_bodysize_length_mom_YearBatch_8_18_25.rds")
# model_mom_on_bodysize_length_mom_YearBatch <-
# MCMCglmm(avg_length ~ PredatorCue + YearBatch, random =
# ~MomId, rcov = ~units, family = 'gaussian', data =
# bodysize_per_fish_data, nitt = 50000, burnin = 5000,
# verbose = FALSE)
# saveRDS(model_mom_on_bodysize_length_mom_YearBatch, file
# = 'model_bodysize_length_mom_YearBatch_8_18_25.rds')
summary(model_mom_on_bodysize_length_mom_YearBatch)
```

```
##
##  Iterations = 5001:49991
##  Thinning interval  = 10
##  Sample size  = 4500
##
##  DIC: 488.2316
##
##  G-structure:  ~MomId
##
##       post.mean  l-95% CI u-95% CI eff.samp
## MomId    0.5461 6.152e-17    3.926    145.1
##
##  R-structure:  ~units
##
##       post.mean l-95% CI u-95% CI eff.samp
## units     16.27    11.09    21.31    716.7
##
##  Location effects: avg_length ~ PredatorCue + YearBatch
##
##             post.mean  l-95% CI  u-95% CI eff.samp  pMCMC
## (Intercept) 41.748838 39.261311 44.320335     4223 <2e-04 ***
## PredatorCue -0.056586 -1.711683  1.669424     4500 0.9458
## YearBatch   -0.930918 -1.891342 -0.006529     4251 0.0476 *
## ---
## Signif. codes:  0 '***' 0.001 '**' 0.01 '*' 0.05 '.' 0.1 ' ' 1
```

```r
summary(model_mom_on_bodysize_length_mom_YearBatch$VCV)
```

```
##
## Iterations = 5001:49991
## Thinning interval = 10
## Number of chains = 1
## Sample size per chain = 4500
##
## 1. Empirical mean and standard deviation for each variable,
##    plus standard error of the mean:
##
##          Mean    SD Naive SE Time-series SE
## MomId  0.5461 1.777  0.02649         0.1475
## units 16.2691 2.672  0.03983         0.0998
##
## 2. Quantiles for each variable:
##
##            2.5%       25%       50%       75%  97.5%
```

```
## MomId 3.370e-16 2.133e-12 1.268e-08 4.801e-04  5.827
## units 1.159e+01 1.445e+01 1.608e+01 1.787e+01 22.037
```

```r
HPDinterval(model_mom_on_bodysize_length_mom_YearBatch$VCV)
```

```
##              lower     upper
## MomId 6.152120e-17  3.926158
## units 1.109494e+01 21.313855
## attr(,"Probability")
## [1] 0.95
```

```r
# Width
model_mom_on_bodysize_width_mom_YearBatch <- readRDS("model_bodysize_width_mom_YearBatch_8_18_25.rds")
# model_mom_on_bodysize_width_mom_YearBatch <-
# MCMCglmm(avg_width ~ PredatorCue + YearBatch, random =
# ~MomId, rcov = ~units, family = 'gaussian', data =
# bodysize_per_fish_data, nitt = 50000, burnin = 5000,
# verbose = FALSE)
# saveRDS(model_mom_on_bodysize_width_mom_YearBatch, file =
# 'model_bodysize_width_mom_YearBatch_8_18_25.rds')
summary(model_mom_on_bodysize_width_mom_YearBatch)
```

```
##
##  Iterations = 5001:49991
##  Thinning interval  = 10
##  Sample size  = 4500
##
##  DIC: 170.9134
##
##  G-structure:  ~MomId
##
##       post.mean  l-95% CI u-95% CI eff.samp
## MomId   0.00397 6.492e-17  0.02142    392.3
##
##  R-structure:  ~units
##
##       post.mean l-95% CI u-95% CI eff.samp
## units    0.4109   0.3026   0.5456     4260
##
##  Location effects: avg_width ~ PredatorCue + YearBatch
##
##             post.mean l-95% CI u-95% CI eff.samp  pMCMC
## (Intercept)   9.08201  8.70903  9.46217     4500 <2e-04 ***
## PredatorCue  -0.16035 -0.44645  0.09874     4500  0.242
## YearBatch    -0.08385 -0.22872  0.05560     3567  0.242
## ---
## Signif. codes:  0 '***' 0.001 '**' 0.01 '*' 0.05 '.' 0.1 ' ' 1
```

```r
summary(model_mom_on_bodysize_width_mom_YearBatch$VCV)
```

```
##
## Iterations = 5001:49991
## Thinning interval = 10
## Number of chains = 1
## Sample size per chain = 4500
##
## 1. Empirical mean and standard deviation for each variable,
##    plus standard error of the mean:
##
##          Mean      SD  Naive SE Time-series SE
## MomId 0.00397 0.01900 0.0002832      0.0009590
## units 0.41093 0.06504 0.0009695      0.0009965
##
```

```
## 2. Quantiles for each variable:
##
##            2.5%       25%       50%       75%   97.5%
## MomId 9.047e-16 2.589e-11 3.529e-08 0.0000199 0.05104
## units 3.047e-01 3.656e-01 4.037e-01 0.4493975 0.55169
```

```r
HPDinterval(model_mom_on_bodysize_width_mom_YearBatch$VCV)
```

```
##              lower      upper
## MomId 6.492097e-17 0.02142442
## units 3.025730e-01 0.54564218
## attr(,"Probability")
## [1] 0.95
```

No association between mom or treatment and body size.

Investigating immediate effects of introducing molly alarm cues to predator tracking system

```r
# Load molly alarm cue data
alarm_cue_data <- read.csv("../datasheets/cichlid_feeding_data.csv")

# Prepare for alarm cue data
day_only_data_predator <- subset(day_only_data, PredatorCue ==
    1)
day_only_data_predator$alarm_cue_day <- as.factor(ifelse(day_only_data_predator$Date %in%
    alarm_cue_data$date, 1, 0))
unique(day_only_data_predator$Date[day_only_data_predator$alarm_cue_day ==
    1])  # Worked correctly
```

```
##  [1] "6/16/23"  "5/26/23"  "6/5/23"   "6/12/23"  "6/7/23"   "6/20/23"
##  [7] "6/1/23"   "6/23/23"  "8/1/23"   "8/11/23"  "8/3/23"   "8/18/23"
## [13] "7/28/23"  "8/9/23"   "8/17/23"  "7/26/23"  "7/21/23"  "10/23/23"
## [19] "11/3/23"  "11/14/23" "10/27/23" "11/7/23"  "11/21/23" "12/1/23"
## [25] "10/2/23"  "9/29/23"  "9/27/23"  "10/10/23" "10/13/23" "10/5/23"
## [31] "10/16/23"
```

```r
# Run models

alarm_model_velocity_daily <- readRDS("alarm_model_velocity_daily.rds")
# alarm_model_velocity_daily <- MCMCglmm(MedianVel_scaled ~
# alarm_cue_day + ExpDay + YearBatch, random = ~FishId +
# MomId, rcov = ~idh(alarm_cue_day):units, data =
# day_only_data_predator, family = 'gaussian', nitt =
# 300000, burnin = 30000, thin = 100)
# saveRDS(alarm_model_velocity_daily, file =
# 'alarm_model_velocity_daily.rds')
summary(alarm_model_velocity_daily)
```

```
##
##  Iterations = 30001:299901
##  Thinning interval  = 100
##  Sample size  = 2700
##
##  DIC: 3278.55
##
##  G-structure:  ~FishId
##
##        post.mean l-95% CI u-95% CI eff.samp
## FishId    0.2258   0.1125   0.3505    637.1
##
##               ~MomId
##
```

```
##       post.mean  l-95% CI u-95% CI eff.samp
## MomId    0.1247 1.106e-16   0.3273     1053
##
##  R-structure:  ~idh(alarm_cue_day):units
##
##                      post.mean l-95% CI u-95% CI eff.samp
## alarm_cue_day0.units    0.5533   0.5042   0.5979     2700
## alarm_cue_day1.units    0.5843   0.4922   0.6719     2700
##
##  Location effects: MedianVel_scaled ~ alarm_cue_day + ExpDay + YearBatch
##
##                post.mean  l-95% CI  u-95% CI eff.samp pMCMC
## (Intercept)    -0.317232 -0.861353  0.206434     2700 0.215
## alarm_cue_day1  0.051868 -0.036648  0.147180     2700 0.282
## ExpDay          0.003593 -0.001174  0.008000     2700 0.141
## YearBatch       0.049823 -0.180920  0.234486     2700 0.596
```

```r
summary(alarm_model_velocity_daily$VCV)
```

```
##
## Iterations = 30001:299901
## Thinning interval = 100
## Number of chains = 1
## Sample size per chain = 2700
##
## 1. Empirical mean and standard deviation for each variable,
##    plus standard error of the mean:
##
##                        Mean      SD  Naive SE Time-series SE
## FishId               0.2258 0.06537 0.0012581      0.0025900
## MomId                0.1247 0.11187 0.0021529      0.0034473
## alarm_cue_day0.units 0.5533 0.02438 0.0004692      0.0004692
## alarm_cue_day1.units 0.5843 0.04739 0.0009120      0.0009120
##
## 2. Quantiles for each variable:
##
##                           2.5%     25%    50%    75%  97.5%
## FishId               1.310e-01 0.17883 0.2136 0.2616 0.3800
## MomId                8.213e-12 0.05643 0.1029 0.1663 0.3995
## alarm_cue_day0.units 5.078e-01 0.53605 0.5526 0.5694 0.6032
## alarm_cue_day1.units 5.004e-01 0.55090 0.5810 0.6144 0.6840
```

```r
effectiveSize(alarm_model_velocity_daily$Sol)
```

```
##    (Intercept) alarm_cue_day1         ExpDay      YearBatch
##           2700           2700           2700           2700
```

```r
autocorr.diag(alarm_model_velocity_daily$Sol)
```

```
##           (Intercept) alarm_cue_day1       ExpDay    YearBatch
## Lag 0     1.000000000   1.0000000000  1.000000000  1.000000000
## Lag 100  -0.020339577  -0.0073420173 -0.006030586 -0.001859139
## Lag 500  -0.012967269   0.0117627027  0.045902198 -0.033432150
## Lag 1000 -0.014480923   0.0042913023  0.042550535 -0.015761186
## Lag 5000  0.003516825  -0.0009322602  0.008481495  0.018737568
```

```r
effectiveSize(alarm_model_velocity_daily$VCV)
```

```
##               FishId                MomId alarm_cue_day0.units
##             637.1092            1053.0343            2700.0000
## alarm_cue_day1.units
##            2700.0000
```

```r
autocorr.diag(alarm_model_velocity_daily$VCV)
```

```
##                FishId       MomId alarm_cue_day0.units alarm_cue_day1.units
## Lag 0     1.000000000  1.00000000          1.000000000          1.000000000
## Lag 100   0.157022850  0.11969284          0.013956210         -0.009550955
## Lag 500   0.104167921  0.06979695         -0.020669414          0.014103255
## Lag 1000  0.091405649  0.04958614          0.003167347         -0.048840049
## Lag 5000 -0.002942739 -0.01024998         -0.025454796          0.009683942
```

```r
alarm_model_corner_daily <- readRDS("alarm_model_corner_daily.rds")
# alarm_model_corner_daily <- MCMCglmm(MeanCorner_scaled ~
# alarm_cue_day + ExpDay, random = ~FishId + MomId, rcov =
# ~idh(alarm_cue_day):units, data = day_only_data_predator,
# family = 'gaussian', nitt = 300000, burnin = 30000, thin
# = 100) saveRDS(alarm_model_corner_daily, file =
# 'alarm_model_corner_daily.rds')
summary(alarm_model_corner_daily)
```

```
##
##  Iterations = 30001:299901
##  Thinning interval  = 100
##  Sample size  = 2700
##
##  DIC: 3030.931
##
##  G-structure:  ~FishId
##
##        post.mean l-95% CI u-95% CI eff.samp
## FishId    0.5623   0.3551   0.8094     3070
##
##                ~MomId
##
##       post.mean  l-95% CI u-95% CI eff.samp
## MomId  0.005919 5.829e-17   0.0351    992.6
##
##  R-structure:  ~idh(alarm_cue_day):units
##
##                      post.mean l-95% CI u-95% CI eff.samp
## alarm_cue_day0.units    0.4721   0.4339   0.5133     2877
## alarm_cue_day1.units    0.4623   0.3884   0.5333     2700
##
##  Location effects: MeanCorner_scaled ~ alarm_cue_day + ExpDay
##
##                post.mean  l-95% CI  u-95% CI eff.samp  pMCMC
## (Intercept)     0.264583  0.061340  0.496164     2700 0.0193 *
## alarm_cue_day1  0.024549 -0.059645  0.106762     2700 0.5556
## ExpDay         -0.003604 -0.008098  0.000692     2864 0.1178
## ---
## Signif. codes:  0 '***' 0.001 '**' 0.01 '*' 0.05 '.' 0.1 ' ' 1
```

```r
summary(alarm_model_corner_daily$VCV)
```

```
##
## Iterations = 30001:299901
## Thinning interval = 100
## Number of chains = 1
## Sample size per chain = 2700
##
## 1. Empirical mean and standard deviation for each variable,
##    plus standard error of the mean:
##
##                           Mean      SD  Naive SE Time-series SE
```

```
## FishId               0.562309 0.12063 0.0023215      0.0021770
## MomId                0.005919 0.02616 0.0005034      0.0008303
## alarm_cue_day0.units 0.472074 0.02029 0.0003905      0.0003783
## alarm_cue_day1.units 0.462282 0.03670 0.0007062      0.0007062
##
## 2. Quantiles for each variable:
##
##                           2.5%       25%       50%       75%   97.5%
## FishId               3.745e-01 4.780e-01 5.462e-01 6.291e-01 0.84526
## MomId                4.653e-16 1.739e-12 5.348e-09 2.903e-05 0.07416
## alarm_cue_day0.units 4.340e-01 4.583e-01 4.717e-01 4.854e-01 0.51389
## alarm_cue_day1.units 3.928e-01 4.369e-01 4.606e-01 4.857e-01 0.53969
```

```
effectiveSize(alarm_model_corner_daily$Sol)
```

```
##    (Intercept) alarm_cue_day1         ExpDay
##        2700.00        2700.00        2863.86
```

```
autocorr.diag(alarm_model_corner_daily$Sol)
```

```
##            (Intercept) alarm_cue_day1       ExpDay
## Lag 0     1.0000000000    1.000000000  1.000000000
## Lag 100  -0.0019213995   -0.026220303 -0.029635892
## Lag 500  -0.0147213969    0.012313787 -0.028140854
## Lag 1000 -0.0160888276    0.023882407 -0.005784866
## Lag 5000 -0.0008877686    0.004585938  0.011026918
```

```
effectiveSize(alarm_model_corner_daily$VCV)
```

```
##               FishId                MomId alarm_cue_day0.units
##            3070.2729             992.6044            2877.2246
## alarm_cue_day1.units
##            2700.0000
```

```
autocorr.diag(alarm_model_corner_daily$VCV)
```

```
##                FishId       MomId alarm_cue_day0.units alarm_cue_day1.units
## Lag 0     1.000000000 1.000000000          1.000000000          1.000000000
## Lag 100  -0.025001791 0.167604209         -0.031961580         -0.022432740
## Lag 500   0.002868610 0.060581734          0.024407680         -0.011178694
## Lag 1000 -0.027427796 0.003080629          0.003426285         -0.003628010
## Lag 5000  0.009869094 0.021027899         -0.003473257          0.003605845
```

```
### No notable effects of alarm cue day on behavioral means
### or variance.
```

END OF ANALYSIS
