# Supplementary material for: Born This Way: Individuality Is Seeded Before Birth and Robust to Ecological Stress
Source: Ecol Lett. 2026 Sep 14;29(9):e70454. doi: 10.1111/ele.70454 (PMC13574246; doi:10.1111/ele.70454)
Supplement: Supplementary file 2 [file ELE-29-0-s001.docx]

Supplement for “Born this way: individuality is seeded before birth and robust to ecological stress”

Supplemental Table

Table S1. Outputs for the mixed models showing that body size components did not predict behaviors (velocity, refuge use).

| **Velocity** | | |
| --- | --- | --- |
| **Variable** | **post.mean** | **95% CI** |
| Length | -0.006 | -0.029, 0.017 |
| Width | 0.019 | -0.130, 0.154 |
| **Refuge use** | | |
| **Variable** | **post.mean** | **95% CI** |
| Length | -0.009 | -0.036, 0.020 |
| Width | 0.012 | -0.061, 0.303 |

Supplemental Methods

Maternal behavior tracking experimental setup

Observation tanks were made of white acrylic (Perspex) and had identical environments (dimensions = 15x19x15cm), each containing white gravel along two of the four sides of the tank, as well as a standpipe covered in a sponge filter that fish used as a refuge. All fish experienced the same feeding schedule (fed a standard amount of Tetra-Min flake fish food and brine shrimp twice per day), daily light cycle (12:12 light:dark), water temperature (26±2 °C), and water depth (10cm). The observation tank area was also surrounded by white curtains to reduce potential disturbance from external stimuli.

Tracking behavior

For efficiency and reproducibility, all videos were processed within one of two automated pipelines [(Mölder *et al.* 2021)](https://paperpile.com/c/dxpwif/U28ql), specialized either for adults or for juveniles. All videos were first cropped to exclude adjacent tanks using ffmpeg (version 6.1.1, FFmpeg Developers), based on manually labeled points. We also used labeled center-points to assign the (up to) four tracks in each video to specific individuals. For tracking adults, we used a neural network (efficientNet b3 ; [Tan & Le 2019](https://paperpile.com/c/dxpwif/Vnm2c)), trained and implemented using SLEAP [(Pereira *et al.* 2022)](https://paperpile.com/c/dxpwif/DBl1R). For tracking babies, we similarly began with a neural network (LEAP; [Pereira *et al.* 2019)](https://paperpile.com/c/dxpwif/SAZBG) using SLEAP [(Pereira *et al.* 2022)](https://paperpile.com/c/dxpwif/DBl1R), which was generally accurate, but struggled when small fish were above gravel. To address this, we also used a custom tracker based on a mixture of gaussians background subtractor [(Zivkovic 2004)](https://paperpile.com/c/dxpwif/zfsFS), implemented using openCV [(Bradski & Kaehler 2015)](https://paperpile.com/c/dxpwif/9OXBD) in python. Because this takes advantage of frame-to-frame differences, it was successful tracking fish over gravel. Both sets of tracks were filtered to remove extremely high-density pixels (which likely represent false detections of stationary objects). To combine these two streams, we used a custom implementation of the Viterbi algorithm [(Viterbi 1967)](https://paperpile.com/c/dxpwif/rCyPY), which seeks to estimate the most likely path of some unknown and hidden value (in this case, the location of fish) based on probabilistic observed values (the two parallel detections of fish). From these post-processed tracks we calculated the median velocity, proportion of frames “active” (defined as moving > 5 pixels/second), median velocity when “active,” and proportion of frames detected in the corner with the standpipe (which acts as a refuge). Videos were manually inspected by an expert observer (JHG) for major issues that would result in inaccurate tracking (i.e., missing or moved fish, camera movement). We manually scored 1-minute segments from a randomly selected day within the first and last week for 6 groups (a total of 18,000 frames). The mean false detection rate was only 6.2% (+/- .03% SEM), suggesting for >90% of frames, the fish was either accurately detected or not visible (e.g., under the refuge).

## Measuring body size

To estimate individual body size of offspring at the end of the experiment, we used a custom python script to extract crops of each individual (based on their detected position) on day 27 and manually labeled the head, tail-peduncle, and body width (at its widest point) using Supervisely, a web-based computer vision platform [(Supervisely 2023)](https://paperpile.com/c/dxpwif/9ywOM). Body length was measured as the distance between the head and tail-peduncle label (standard length). The identity and treatment of fish in these crops were blinded during labeling.

## References

[Bradski, G.R. & Kaehler, A. (2015). *Learning OpenCV*. 2nd edn. O’Reilly Media, Sebastopol, CA.](http://paperpile.com/b/dxpwif/9OXBD)

[Mölder, F., Jablonski, K.P., Letcher, B., Hall, M.B., Tomkins-Tinch, C.H., Sochat, V., *et al.* (2021). Sustainable data analysis with Snakemake. *F1000Res.*, 10, 33.](http://paperpile.com/b/dxpwif/U28ql)

[Pereira, T.D., Aldarondo, D.E., Willmore, L., Kislin, M., Wang, S.S.-H., Murthy, M., *et al.* (2019). Fast animal pose estimation using deep neural networks. *Nat. Methods*, 16, 117–125.](http://paperpile.com/b/dxpwif/SAZBG)

[Pereira, T.D., Tabris, N., Matsliah, A., Turner, D.M., Li, J., Ravindranath, S., *et al.* (2022). SLEAP: A deep learning system for multi-animal pose tracking. *Nat. Methods*, 19, 486–495.](http://paperpile.com/b/dxpwif/DBl1R)

[Supervisely. (2023). *Supervisely Computer Vision platform*.](http://paperpile.com/b/dxpwif/9ywOM)

[Tan, M. & Le, Q. (2019). EfficientNet: Rethinking Model Scaling for Convolutional Neural Networks. In: *International Conference on Machine Learning*. Presented at the International Conference on Machine Learning, PMLR, pp. 6105–6114.](http://paperpile.com/b/dxpwif/Vnm2c)

[Viterbi, A. (1967). Error bounds for convolutional codes and an asymptotically optimum decoding algorithm. *IEEE Trans. Inf. Theory*, 13, 260–269.](http://paperpile.com/b/dxpwif/rCyPY)

[Zivkovic, Z. (2004). Improved adaptive Gaussian mixture model for background subtraction. In: *Proceedings of the 17th International Conference on Pattern Recognition, 2004. ICPR 2004*. Presented at the Proceedings of the 17th International Conference on Pattern Recognition, 2004. ICPR 2004., IEEE, pp. 28–31 Vol.2.](http://paperpile.com/b/dxpwif/zfsFS)
